# Comparative Effectiveness of Exercise and Protein-Based Interventions on Muscle Strength, Mass, and Function in Sarcopenia: A Systematic Review and Network Meta-Analysis

**DOI:** 10.1016/j.jnha.2025.100718

**Published:** 2025-11-09

**Authors:** Ruixiang Yan, Shiqi Jia, Di Lu, Wenfeng Song, Wenfeng Zhang, Jian Sun, Duanying Li

**Affiliations:** a School of Athletic Training, Guangzhou Sport University, Guangzhou, Guangdong, China; b Guangdong Provincial Key Laboratory of Human Sports Performance Science, Guangzhou Sport University, Guangzhou, Guangdong, China; c Key Laboratory of Human-Computer Intelligent Interaction for Athletic Performance and Health Promotion, Guangzhou Sport University, Guangzhou, Guangdong, China

**Keywords:** Sarcopenia, Multicomponent exercise, Protein-based nutritional supplementation

## Abstract

**Background:**

Exercise, protein supplementation, and their combination are guideline-recommended strategies for managing sarcopenia. This study aimed to compare the effectiveness of various types of voluntary and simulated exercise on the outcomes of muscle strength, physical function, and muscle mass in individuals with sarcopenia.

**Methods:**

We systematically searched PubMed, Embase, Web of Science, and the Cochrane Central Register of Controlled Trials through December 2024 and conducted a frequentist random-effects network meta-analysis. The certainty of evidence was assessed using the GRADE framework, and interventions were categorized according to their relative effectiveness and certainty ratings.

**Results:**

A total of 96 eligible studies involving 7,596 participants were included. Resistance and balance training combined with protein-based nutritional supplementation (RBT + Nu) was the most effective intervention for improving grip strength (MD = 5.45 kg, 95% CI: 3.58–7.33), gait speed (MD = 0.20 m/s, 95% CI: 0.11 to 0.29), SPPB (MD = 3.59 points, 95% CI: 1.91–5.27), and skeletal muscle index (MD = 0.95 kg/m², 95% CI: 0.16–1.74). Improvements in gait speed and SPPB exceeded the minimal clinically important difference, while gains in grip strength were potentially clinically meaningful. Resistance and balance training (RBT) achieved the most significant improvement in the timed up and go test (MD = −2.29 s, 95% CI: −3.16 to −1.41), whereas aerobic and resistance training combined with nutrition was most effective in the five-times sit-to-stand test (MD = −2.86 s, 95% CI: −4.55 to −1.17); both improvements are potentially clinically meaningful. Resistance training with nutrition showed the largest improvements in knee extension strength (SMD = 0.98, 95% CI: 0.63–1.33) and appendicular skeletal muscle mass (MD = 0.33 kg/m², 95% CI: 0.27 to 0.38). Aerobic, resistance, and balance training produced the greatest benefit for balance performance (SMD = 0.73, 95% CI: 0.13–1.34).

**Conclusion:**

High-certainty evidence supports RBT + Nu as the most effective intervention for improving muscle strength, muscle mass, and physical function in individuals with sarcopenia. We recommend combining RBT with protein and amino acid supplementation as an optimal strategy when feasible.

## Highlights

• Multicomponent exercise plus protein supplementation most effectively improves sarcopenia outcomes.
• Adding amino acids to protein supplementation further enhances muscle strength and function gains.
• Simulated exercise (e.g., vibration, EMS) offers high adherence and valuable alternatives for frail patients.

## 1 Introduction

Sarcopenia is an age-related syndrome characterized by the progressive loss of skeletal muscle mass, strength, and physical performance [1,2]. It is associated with an elevated risk of adverse clinical outcomes, including disability, falls, and mortality [1,2]. Although prevalence estimates vary depending on diagnostic criteria and population characteristics, conservative data suggest that approximately 5–10% of the general population may be affected by sarcopenia [3]. With the ongoing trend of global population aging, the burden of sarcopenia is expected to increase further, placing substantial strain on healthcare systems and driving up related medical costs [4].

Currently, no pharmacological treatment has demonstrated definitive efficacy for sarcopenia in clinical practice [5]. While some agents have shown modest improvements in lean body mass in select populations, consistent benefits in muscle strength and functional outcomes remain unproven [6]. Importantly, the primary therapeutic goal for sarcopenia is to maintain or improve muscle mass and, more critically, to halt or reverse the decline in muscle strength and preserve functional independence and quality of life [3]. Resistance training remains the most evidence-based strategy among the available interventions, having consistently demonstrated efficacy in improving muscle strength, mass, and physical performance in older adults [3]. However, sarcopenia often coexists with age-related comorbidities such as cardiopulmonary impairments and balance deficits, which may limit the feasibility or safety of intensive exercise. As such, current clinical guidelines recommend multimodal exercise interventions, integrating resistance, aerobic, and/or balance training, to address the broader functional limitations in this population [7]. Although tailored and supervised exercise is feasible and beneficial even for older adults with severe frailty or functional limitations, practical barriers such as postoperative recovery, joint pain, and safety concerns may temporarily limit the intensity, frequency, or mode of voluntary training [8]. In this context, simulated exercise modalities such as electrical muscle stimulation (EMS) and whole-body vibration (WBV) have been explored primarily as adjuncts or short-term options for individuals unable to engage in conventional exercise fully. Preliminary evidence suggests that EMS may effectively prevent acute postoperative muscle wasting and promote short-term gains in muscle mass and strength; however, its long-term efficacy in enhancing muscle strength and physical function remains uncertain [9,10]. Although definitions of sarcopenia vary based on criteria such as muscle mass, strength, or physical performance, these differences do not substantially alter the recommended principles for exercise prescription [11]. Future research should focus on optimizing these prescriptions and enhancing adherence to interventions to improve their real-world clinical effectiveness [11,12].

Adequate protein intake is widely accepted as a crucial non-pharmacological strategy for preventing or mitigating the progression of sarcopenia [13,14]. However, the current evidence regarding its efficacy as a standalone intervention remains inconsistent, and a definitive consensus has yet to be established [15,16]. Given the complex and multifactorial pathophysiology of sarcopenia, isolated interventions may be insufficient to elicit clinically meaningful improvements [17]. Evidence suggests that combining protein supplementation with resistance training may further amplify improvements in muscle strength and mass [18,19]. Nevertheless, most available data come from studies involving healthy older adults participating in structured resistance training. There is a lack of systematic evaluation regarding the effects of different exercise modalities combined with various forms of protein supplementation. In particular, there is a need for comparisons between isolated protein supplementation, amino acid supplementation, and their combination on treatment outcomes in individuals with sarcopenia.

To address these gaps, we conducted a network meta-analysis that integrated direct and indirect evidence to compare the effectiveness of voluntary or simulated exercise, protein supplementation, and their combination on muscle strength, muscle mass, and physical performance in individuals with sarcopenia. In addition, subgroup analyses were performed to explore the effects of different forms of protein supplementation. Using a minimally contextualized framework and incorporating adherence data, we aimed to provide a comprehensive synthesis of the evidence to support the development of personalized, evidence-based strategies for sarcopenia management in clinical practice.

## 2 Methods

### 2.1 Protocol and registration

This systematic review and network meta-analysis were registered in the PROSPERO database (Registration number: CRD420251033448). The study was conducted in accordance with the PRISMA 2020 (Preferred Reporting Items for Systematic Reviews and Meta-Analyses) guidelines and the extended statement for network meta-analyses (PRISMA-NMA) [20,21].

### 2.2 Search strategy and study selection

A comprehensive literature search was conducted in four electronic databases: PubMed, Web of Science, Cochrane Central Register of Controlled Trials (CENTRAL), and Embase. The search included studies published from database inception to December 10, 2024, focusing on randomized controlled trials (RCTs) or quasi-experimental studies investigating the effects of different exercise and/or nutritional interventions in individuals with sarcopenia. For eligibility, three reviewers (GS, BW, and LX) independently screened the titles, abstracts, and full texts. Discrepancies were resolved by consulting a fourth reviewer (YE). Additionally, the reference lists of included studies and relevant systematic reviews were manually screened to identify additional eligible studies. The complete search strategies for all databases are provided in Appendix 1.

### 2.3 Eligibility criteria

Eligibility was determined using the PICOS framework (Population, Intervention, Comparator, Outcomes, and Study design) [22]. Studies were included if they met all of the following criteria:

#### 2.3.1 Population

We included participants aged >50 years who were diagnosed with sarcopenia or identified as having possible sarcopenia. No uniform diagnostic criteria were imposed; instead, studies were accepted based on the sarcopenia definitions they employed, including but not limited to those proposed by authoritative bodies such as EWGSOP and AWGS, as well as researcher-defined criteria. All accepted definitions included at least one of the following core domains: low muscle mass, low muscle strength, or impaired physical performance.

#### 2.3.2 Intervention

We included interventions involving voluntary or simulated exercise, protein-based nutritional supplementation, or a combination of both.

#### 2.3.3 Comparator

Eligible comparators included health education, usual care, or placebo interventions.

#### 2.3.4 Outcomes

By the ICSFR recommendations [1], the primary and secondary outcomes were categorized as follows: Primary outcomes: Grip strength, Appendicular skeletal muscle mass index (ASMI), and Gait speed. Secondary outcomes: Knee extension strength, Five-Times Sit-to-Stand Test (5STS), Timed Up and Go (TUG) test, Short Physical Performance Battery (SPPB), Balance test, and Skeletal muscle index (SMI). In line with this categorization, ASMI was defined as appendicular skeletal muscle mass divided by height squared (ASM/height², kg/m²). In contrast, SMI was defined as total skeletal muscle mass divided by height squared (SMM/height², kg/m²). SMI was considered a secondary outcome when studies reported whole-body rather than appendicular skeletal muscle mass [23].

#### 2.3.5 Study design

Only RCTs were included.

The following exclusion criteria were applied: [1] studies focusing on sarcopenic populations with specific comorbidities such as cancer, diabetes, stroke, HIV, chronic obstructive pulmonary disease, chronic kidney disease, liver cirrhosis, other serious illnesses, or recent organ transplantation; [2] studies involving pharmacological interventions [3]; conference abstracts, protocols, or systematic reviews [4]; non-English language publications [5]; studies lacking sufficient outcome data; and [6] studies for which complete reports could not be retrieved through databases or other means.

### 2.4 Data extraction

Two reviewers (GS and BW) independently extracted data from each eligible study using a standardized, predesigned form. Extracted information included: study characteristics (first author, year of publication, country, setting, and diagnostic criteria for sarcopenia), participant characteristics (age, sample size), intervention characteristics (type, duration, frequency, intensity, and dosage), and outcome data (means and standard deviations for continuous outcomes, proportions or event rates for binary outcomes). A third reviewer (LX) verified the data extraction and resolved any discrepancies. For studies with missing or incomplete data, we emailed the corresponding authors up to three times over three weeks.

### 2.5 Measures of treatment effect

In this meta-analysis, treatment effects were evaluated using changes in mean differences (MD) and standard deviations (SD). When SD was not directly reported in the original studies, it was estimated using alternative parameters such as standard error (SE), 95% confidence intervals (CI), p-values, or t-statistics, following established statistical methods [24]. For studies that did not report the standard deviation of the change from baseline, we estimated it using the following formula:

$${SD}_{change}=\sqrt{{SD}_{baseline}^{2}+{SD}_{Post}^{2}-2\times r\times {SD}_{baseline}\times {SD}_{post}}$$

Where r represents the assumed correlation coefficient between baseline and post-intervention measurements. In this analysis, we adopted a conservative estimate of r = 0.5, reflecting a moderate test–retest reliability as widely accepted in the literature. This value was selected to balance potential variability between time points and enhance the pooled estimates' robustness and credibility [24].

### 2.6 Quality assessment of evidence

We assessed the risk of bias for included randomized controlled trials using the Cochrane Risk of Bias 2.0 tool (ROB 2.0), covering five key domains: random sequence generation, allocation concealment, blinding, incomplete outcome data, and selective reporting [25]. Each study was assigned an overall risk of bias score as follows: low risk (score = 1) if all domains were rated as low risk; high risk (score = 3) if at least one domain was rated as high risk; and some concerns (score = 2) for all other scenarios. Two reviewers independently assessed the risk of bias, and disagreements were resolved through discussion.

Furthermore, the confidence in the results of the network meta-analysis was assessed using the CINeMA (Confidence in Network Meta-Analysis) framework [26,27], which evaluates six domains: within-study bias, reporting bias, indirectness, imprecision, heterogeneity, and incoherence. These domains assess systematic errors within individual studies, selective reporting or publication bias, the applicability of the evidence to the research question, the width of confidence intervals, consistency across studies, and discrepancies between direct and indirect evidence.

### 2.7 Minimally contextualized framework

We applied a minimally contextualized framework to assess imprecision, using the control group as the reference and classifying interventions based on the magnitude of effect and certainty of evidence [28]. This method determines whether an intervention achieves a change exceeding the minimally important difference (MID) perceived by individuals [29,30]. The certainty of evidence was graded according to the GRADE framework as high, moderate, low, or very low [28].

For outcomes with established MID including handgrip strength (5.0 kg) [31], usual gait speed (0.10 m/s) [32], five-times sit-to-stand (2.3 seconds) [33], TUG (2.1 seconds) [34], and SPPB (1.0 point) [35], we adopted a partially contextualized framework, classifying effects into four categories: Clinically effective: point estimate and 95% CI both exceed the MID; Possibly clinically important: 95% CI crosses the MID but does not include the null; Effective but not clinically important: 95% CI lies between the null and the MID; Ineffective or uncertain: 95% CI includes the null.

For outcomes without clearly defined MID, a fully minimally contextualized framework was applied, classifying interventions as: Among the most effective, CI excludes the null, and the point estimate shows a strong effect; intermediately effective, CI excludes the null, but the effect size is modest; and among the least effective, CI includes the null, or the point estimate is near zero.

### 2.8 Statistical analysis

All statistical analyses were performed within a frequentist framework using the netmeta package in R. A graph-theoretical approach was employed to construct the treatment network. Effect sizes were estimated using weighted least squares regression and solved using the Moore-Penrose generalized inverse matrix. A random-effects model was adopted to account for between-study heterogeneity [36,37]. For outcomes measured on consistent scales, MD was used as an effect estimate. When outcomes were assessed using different measurement instruments or units, such as knee extension strength or balance performance, standardized mean differences (SMD) with 95% confidence intervals were reported to ensure comparability. Heterogeneity across the network was assessed using the generalized Cochran’s Q statistic globally and locally. To evaluate inconsistency between direct and indirect evidence, we employed the node-splitting method, which tests the difference between the two sources of evidence. A p-value < 0.05 was interpreted as statistically significant inconsistency [38].

The geometry of the network was visualized using network plots, where nodes represent interventions and edges indicate direct comparisons. Forest plots and league tables were generated to present relative effect estimates across treatment comparisons. To facilitate ranking, the Surface Under the Cumulative Ranking curve (SUCRA) values were calculated and displayed using rank-heat plots [39,40]. To assess publication bias, we generated funnel plots and used Egger’s test to evaluate asymmetry.

Finally, we conducted five subgroup analyses, including: Primary subgroup analyses: newly constructed networks examining (1) different exercise modalities, (2) types of protein supplementation (protein alone, amino acids, or combination), and (3) combined exercise and protein supplementation interventions. Secondary subgroup analyses: (1) residential setting (community vs. healthcare institutions), (2) intervention duration (≤12 weeks vs. > 12 weeks), (3) diagnostic criteria (EWGSOP, AWGS, or others), and (4) region (developed vs. developing countries).

## 3 Results

### 3.1 Literature selection and study characteristics

**Fig. 1:**
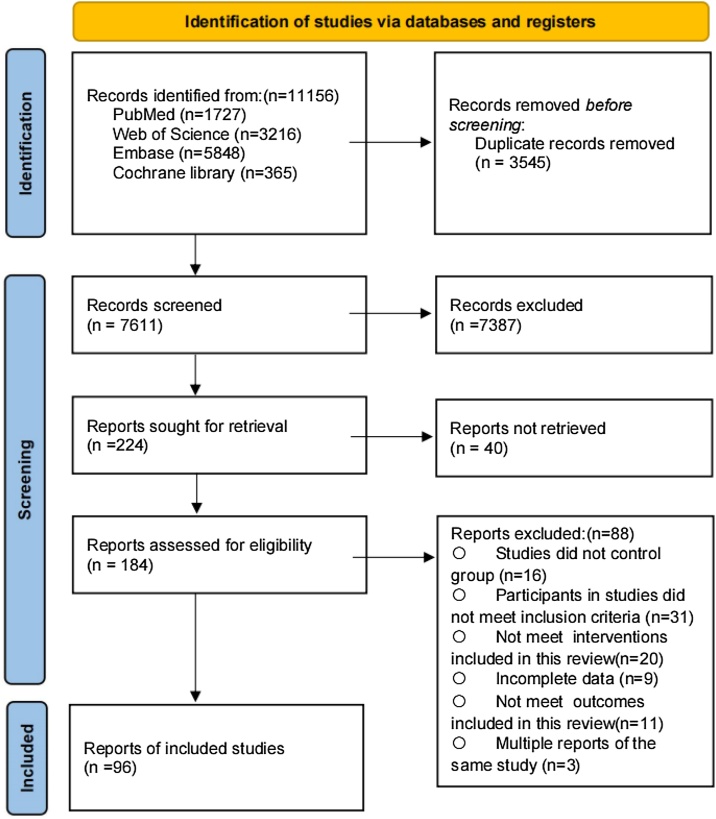
PRISMA Flow diagram of the search process for studies.

A total of 11,156 potentially relevant records were identified through systematic database searches. After removing duplicates, 7,611 records remained for title and abstract screening. Of these, 184 full-text articles were assessed for eligibility. Ultimately, 96 studies comprising 7,596 participants, with a mean age of 74.26 ± 6.54 years, were included in the final systematic review and meta-analysis. Fig. 1 illustrates the complete screening and selection process, and detailed characteristics of the included studies are provided in Table S2.1.

### 3.2 Risk of bias, certainty of evidence, and consistency

**Fig. 2:**
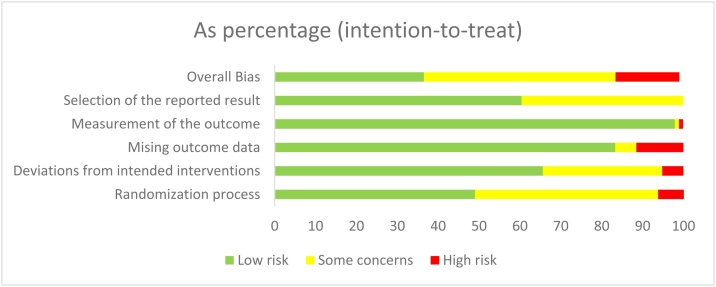
Summary of the risk of bias assessment in the individual domains of the included studies.

Overall, 36 studies (37.5%) were classified as having a low risk of bias, 45 studies (46.9%) as having some concerns, and 15 studies (15.6%) as having a high risk of bias (Fig. 2). The detailed risk of bias assessment for each study is presented in Table S3.1.

In the assessment of consistency, no significant global inconsistency was detected, indicating good agreement between direct and indirect evidence across the network. For the SPPB outcome, a consistency assessment could not be performed because the evidence network lacked closed loops; therefore, the pooled effect estimate should be interpreted with caution, and the certainty of evidence was downgraded by two levels in the “inconsistency” domain of CINeMA.

Further node-splitting analysis identified local inconsistency in only eight comparisons involving grip strength (ART: CG), knee extension strength (Nu: CG, RT: CG), and balance test (RT: CG), accounting for just 2.36% of all comparisons. This very low proportion suggests a high degree of concordance between direct and indirect evidence for most comparisons, supporting the robustness of the network estimates. For these eight comparisons, the certainty of evidence was also downgraded by two levels in the "inconsistency" domain of CINeMA, whereas the certainty ratings for all other outcomes remained unchanged.

Moreover, baseline characteristics, including age and grip strength, were generally comparable across interventions (Table S8.1), and no evidence of violation of the transitivity assumption was identified. Funnel plot analyses also showed no evidence of asymmetry, indicating no apparent publication bias (Appendix 9). Finally, a comprehensive evaluation of evidence certainty using the CINeMA framework indicated that most pairwise comparisons were graded as having very low to moderate certainty (Appendix 8).

### 3.3 Muscle strength

**Fig. 3:**
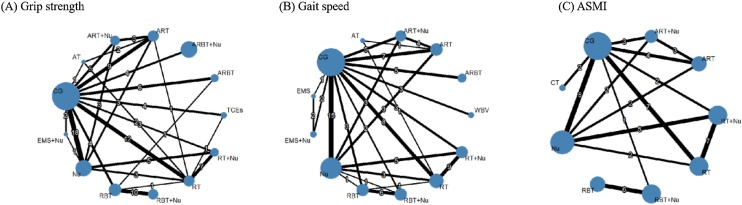
Network plot of interventions for (A) Grip strength, (B) Gait speed, (C) ASMI. Abbreviations: ARBT: Aerobic, resistance, and balance training; ARBT + Nu: Aerobic, resistance, and balance training combined with protein-based nutritional supplementation; ART: Aerobic and resistance training; ART + Nu: Aerobic and resistance training combined with protein-based nutritional supplementation; AT: Aerobic training; CG: Control group; CT: Circuit training; EMS: Electrical muscle stimulation; EMS + Nu: Electrical muscle stimulation combined with protein-based nutritional supplementation; Nu: Protein-based nutritional supplementation; RBT: Resistance and balance training; RBT + Nu: Resistance and balance training combined with protein-based nutritional supplementation; RT: Resistance training; RT + Nu: Resistance training combined with protein-based nutritional supplementation; TCEs: Traditional Chinese exercises; WBV: Whole-body vibration training; TUG:Timed Up and Go; 5STS: Five-times sit-to-stand test; SPPB: Short physical performance battery; ASMI: Appendicular skeletal muscle mass index; SMI: Skeletal muscle index.

**Fig. 4:**
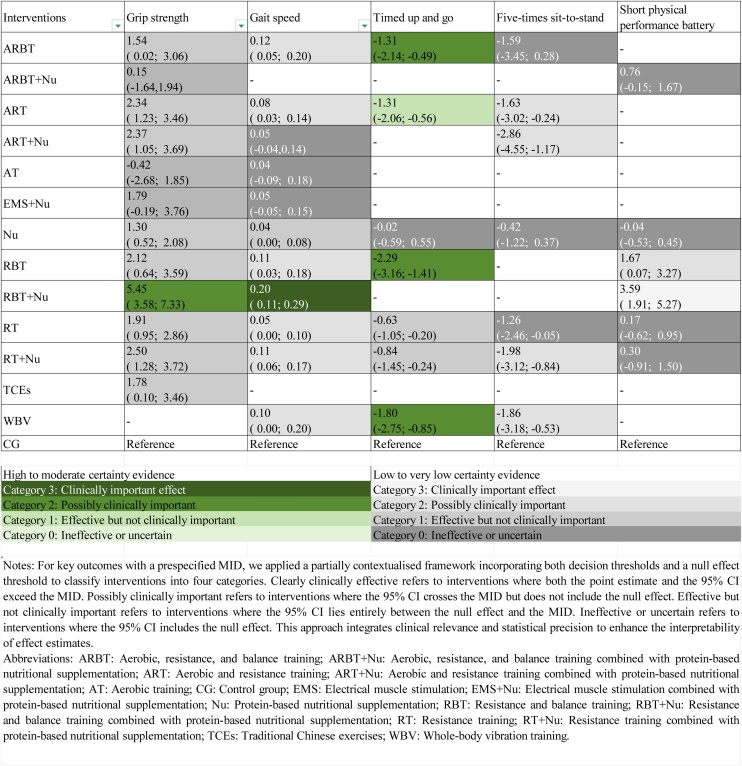
League Table of Pairwise Comparisons between Interventions for Sarcopenia-Related Outcomes, Categorized by Clinical Importance Using a Minimally Contextualized Framework with MID.

Handgrip strength was assessed in 75 trials involving 5,976 participants (Fig. 3). High-certainty evidence indicated that resistance and balance training combined with protein-based nutritional supplementation (RBT + Nu) produced the largest improvement (MD = 5.45 kg, 95% CI: 3.58–7.33; SUCRA 99.9%). However, the 95% CI crossed the prespecified MID, suggesting possible clinical importance. Low-certainty evidence suggested smaller gains with resistance training combined with protein-based nutritional supplementation (RT + Nu), aerobic and resistance training combined with protein-based nutritional supplementation (ART + Nu), aerobic and resistance training, resistance and balance training, resistance training (RT), traditional chinese exercises (TCEs), aerobic, resistance, and balance training (ARBT), and Protein-based nutritional supplementation (Nu). However, none exceeded the MID threshold, indicating that these improvements are unlikely to be clinically important. In contrast, electrical muscle stimulation combined with nutrition (EMS + Nu), aerobic, resistance, and balance training combined with nutrition (ARBT + Nu), and aerobic training (AT) did not demonstrate evidence of benefit (Fig. 4).

**Fig. 5:**
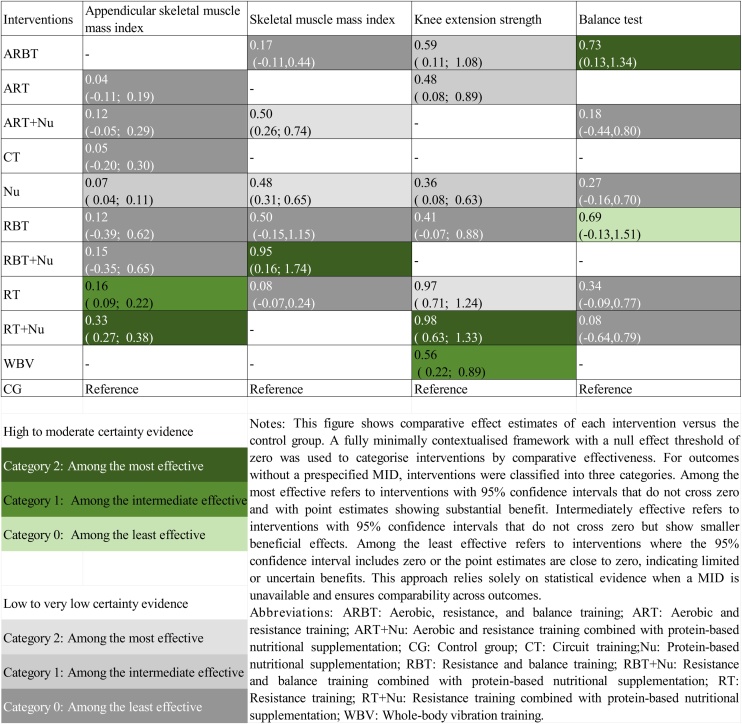
League Table of Pairwise Comparisons between Interventions for Sarcopenia-Related Outcomes, Categorized by Relative Effectiveness Using a Minimally Contextualized Framework without MID.

Knee extension strength was evaluated in 27 trials, including 1,524 participants. High-certainty evidence indicated that RT + Nu was the most effective intervention (SMD = 0.98, 95% CI: 0.63–1.33; SUCRA 90.6%). WBV was also associated with moderate improvements. Low-certainty evidence suggested that RT alone (SMD = 0.97, 95% CI: (0.71–1.24, SUCRA 89.57%) may also be among the most effective options. Moderate improvements were also observed with ARBT, ART, and Nu, whereas RBT appeared to have limited effects (Fig. 5).

### 3.4 Physical performance

Gait speed was evaluated in 58 trials, including 3,776 participants (Fig. 3). High-certainty evidence showed that RBT + Nu yielded the largest improvement (MD = 0.20 m/s, 95% CI: 0.11–0.29; SUCRA 96.9%). Its 95% CI exceeded the prespecified MID, indicating a clinically meaningful effect. Low-certainty evidence indicated improvements with ARBT, RT + Nu, RBT, WBV, ART, and RT, but their 95% CI crossed the MID threshold, suggesting possible clinical importance. Nutrition alone showed a statistically significant effect, but the 95% CI remained below the MID, indicating limited clinical importance. By contrast, EMS, ART + Nu, EMS + Nu, and AT did not demonstrate evidence of benefit (Fig. 4).

The TUG test was evaluated in 23 trials, including 1,151 participants. High-certainty evidence showed that RBT (MD = −2.29 s, 95% CI: −3.16 to −1.41; SUCRA 95.4%) was the most effective intervention in reducing completion time. WBV, ARBT, and ART also reduced completion time, but the 95% confidence intervals for all four interventions crossed the prespecified MID, indicating possible clinical importance. Low-certainty evidence indicated that RT + Nu and RT led to only small reductions that did not reach the MID threshold and are therefore unlikely to be clinically important. Nu alone showed no evidence of benefit (Fig. 4).

5STS was evaluated in 22 trials, including 1,387 participants. Low-certainty evidence showed that ART + Nu (MD = −2.86 s, 95% CI: −4.55 to −1.17; SUCRA 90.0%) may be the most effective intervention for reducing completion time. RT + Nu, WBV, and ART may also reduce completion time; however, the 95% confidence intervals for all four interventions crossed the prespecified MID, suggesting that their clinical importance remains uncertain. In contrast, ARBT, RT, and Nu alone showed no evidence of benefit (Fig. 4).

SPPB was evaluated in 20 trials, including 2,704 participants. Low-certainty evidence showed that RBT + Nu produced the greatest improvement (MD = 3.59 points, 95% CI: 1.91–5.27; SUCRA 99.9%). The 95% CI exceeded the prespecified MID, indicating a clinically meaningful effect. RBT alone may also improve SPPB scores, as its 95% CI crossed the MID threshold, suggesting possible clinical importance. In contrast, ARBT + Nu, RT + Nu, RT, and Nu alone showed no evidence of benefit (Fig. 4).

The balance test was evaluated in 16 trials, including 1,353 participants. High-certainty evidence showed that ARBT significantly improved balance (SMD = 0.73, 95% CI: 0.13–1.34; SUCRA 85.0%) and was the most effective intervention. By contrast, low-certainty evidence suggested that RBT, RT, ART, RT + Nu, and Nu alone were unlikely to produce meaningful improvements (Fig. 5).

### 3.5 Muscle mass

ASMI was evaluated in 35 trials, including 2,009 participants (Fig. 3). High-certainty evidence demonstrated that RT + Nu was the most effective intervention (MD = 0.33 kg/m², 95% CI: 0.27–0.38; SUCRA 93.87%). RT alone showed moderate benefits. Low-certainty evidence indicated that Nu may also confer modest benefits. In contrast, interventions such as RBT + Nu, ART + Nu, RBT, CT, and ART appeared less effective (Fig. 5).

**Fig. 6:**
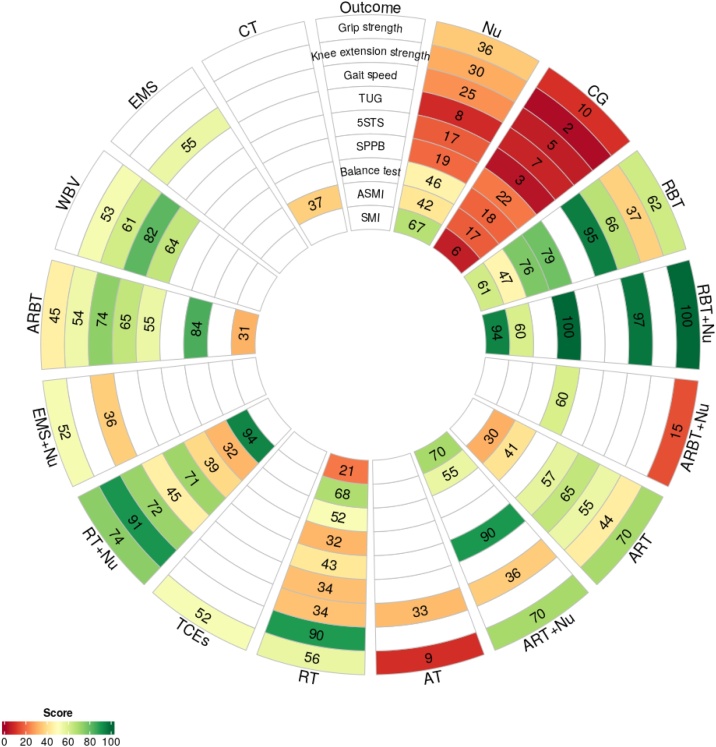
Rank-Heat Plot of Intervention Effectiveness on Sarcopenia-Related Outcomes. Note: The rank heat plot illustrates the relative probability of each intervention achieving higher or lower rankings across multiple outcomes. Darker green cells indicate more favorable rankings, while darker red cells indicate less favorable rankings. The numbers within each cell represent the score assigned to the intervention–outcome pair, with higher scores reflecting better relative performance. Abbreviations: ARBT: Aerobic, resistance, and balance training; ARBT + Nu: Aerobic, resistance, and balance training combined with protein-based nutritional supplementation; ART: Aerobic and resistance training; ART + Nu: Aerobic and resistance training combined with protein-based nutritional supplementation; AT: Aerobic training; CG: Control group; CT: Circuit training; EMS: Electrical muscle stimulation; EMS + Nu: Electrical muscle stimulation combined with protein-based nutritional supplementation; Nu: Protein-based nutritional supplementation; RBT: Resistance and balance training; RBT + Nu: Resistance and balance training combined with protein-based nutritional supplementation; RT: Resistance training; RT + Nu: Resistance training combined with protein-based nutritional supplementation; TCEs: Traditional Chinese exercises; WBV: Whole-body vibration training; TUG:Timed Up and Go; 5STS: Five-times sit-to-stand test; SPPB: Short physical performance battery; ASMI: Appendicular skeletal muscle mass index; SMI: Skeletal muscle index.

SMI was evaluated in 13 trials, including 809 participants. High-certainty evidence showed that RBT + Nu was the most effective intervention (MD = 0.95 kg/m², 95% CI: 0.16–1.74; SUCRA 94.15%). Low-certainty evidence suggested that ART + Nu and Nu may be moderately effective. In contrast, RBT, ARBT, and RT had limited effects (Fig. 5). The SUCRA values for all outcomes are presented in Fig. 6.

### 3.6 Adherence

A total of 83 studies involving 4847 participants reported data on adherence. No significant differences in adherence were found between intervention and control groups. Simulated exercise modalities (EMS, EMS + Nu, WBV) and Nu alone exhibited the highest adherence rates: EMS(OR = 1.79; 95% CI: 0.46–6.94), EMS + Nu(OR = 1.65, 95% CI: 0.52–5.23), WBV(OR = 1.05, 95% CI: 0.38–2.91, Nu(OR = 0.99, 95% CI: 0.73–1.36) (Appendix 5).

### 3.7 Subgroup analysis

Primary subgroup analyses indicated that combining exercise with protein and amino acid supplementation potentially yields greater benefits than exercise, protein supplementation, or amino acid supplementation alone. Specifically, resistance and balance training combined with protein and amino acid supplementation (RBT + Pro + AA) significantly outperformed resistance and balance training combined with protein supplementation alone (RBT + Pro) in improving grip strength, gait speed, SPPB, and ASMI, while also showing greater improvements across other assessed outcomes. Detailed subgroup analyses are presented in (Appendix 10).

### 3.8 Sensitivity analysis

We performed four sensitivity analyses to assess the robustness of the network meta-analysis findings. These analyses excluded studies with [1] high risk of bias [2], small sample sizes (<15 participants per group), [3], inclusion of individuals with possible sarcopenia, and [4] non-standard definitions of sarcopenia. Results remained consistent with the primary analysis, with minimal changes in effect estimates and no substantial shift in effect direction or magnitude, thereby supporting the overall robustness of the conclusions (Appendix 11).

## 4 Discussion

### 4.1 Principal findings

This study systematically evaluated, through network meta-analysis, the effectiveness of exercise interventions, protein supplementation, and their combination in improving muscle mass, muscle strength, and physical function in individuals with sarcopenia. High-certainty evidence indicated that RBT + Nu was the most effective intervention for enhancing handgrip strength and gait speed, with the improvement in gait speed showing clear clinical significance. Low-certainty evidence suggested that RT + Nu was most effective in improving ASMI. Subgroup analysis further revealed that combining exercise with protein and amino acid supplementation may be more advantageous than combining it alone.

### 4.2 Comparison with other studies

The ICFSR recommends resistance training as the first-line intervention for sarcopenia, with high-certainty evidence supporting its effectiveness in improving muscle mass, strength, and physical function [17]. Nevertheless, there is no unified consensus on the optimal training duration, frequency, or intensity [11]. When the aim is to induce greater muscle hypertrophy or adaptive responses, training intensity may need to reach 70–85% of one repetition maximum, performed two to three times per week [41]. For frail or functionally limited older adults, however, such programs are often challenging to implement and may pose increased safety risks [42]. More importantly, strategies that rely on resistance training alone to maintain or improve physical function show limited applicability and cost-effectiveness in this population [[43], [44], [45]]. Functional performance in later life depends not only on muscular strength [46] but also on the coordinated engagement of multiple systems, including balance control, postural stability, and movement efficiency [47]. Although resistance training can substantially enhance strength, its effects on balance and neuromuscular coordination are limited, and the resulting strength gains may transfer only partially to complex functional tasks [48]. Accordingly, optimizing training for people with sarcopenia should look beyond load intensity and emphasize structural diversity and alignment with functional goals to achieve integrated improvements in strength, balance, and daily mobility.

Our study further supports this perspective. Compared with RT alone, RT-based multicomponent interventions produced greater improvements in functional performance. These programs are typically designed to mirror real-world tasks such as walking, turning, and standing up, thereby improving the transfer of training adaptations to everyday functions. Consistent with our observations, a network meta-analysis by Shen et al. [49] reported that multicomponent programs built on resistance training yield more pronounced functional benefits. Physiologically, different training components act through distinct mechanisms on muscular strength, neuromuscular coordination, and metabolic efficiency, creating complementary effects that jointly promote functional improvement. Specifically, resistance training increases muscular strength and power, providing the mechanical foundation for recovery [50]. In contrast, balance training (BT) enhances proprioception, postural control, and neuromuscular coordination, allowing strength gains to be expressed more effectively during complex and dynamic tasks [51]. In our results, this synergy was reflected in significant improvements on the TUG test. TUG evaluates composite functional mobility across sequential sit-to-stand, walking, turning, and sitting phases and demands lower-limb strength, dynamic balance, and transitional control [52]. These task demands align closely with the characteristics of RBT, which simultaneously strengthens the lower limbs and stabilizes posture, enabling more efficient force production with better control during rapid postural transitions. Accordingly, RBT outperformed other interventions on TUG and produced significant gains in gait speed and the SPPB. Notably, when combined with protein supplementation, RBT + Nu achieved clinically meaningful improvements in gait speed and SPPB that exceeded those of other interventions, highlighting the importance of protein support in reinforcing training adaptations and accelerating functional recovery.

AT improves mitochondrial activity and capillary density, enhances oxidative metabolism and energy utilization, and stabilizes energy supply during exercise, thereby sustaining force output during dynamic tasks [52]. Consequently, ART or ART + Nu may optimize performance in repetitive lower-limb tasks, particularly those requiring sustained power and fatigue resistance, such as the 5-STS test. Several studies have also reported that AT alone can effectively improve both dynamic and static balance in older adults [53,54], with static balance gains comparable to those of RT [55]. Moreover, ART has been shown to improve dynamic balance and mobility outcomes, including TUG and gait speed, more effectively than RT alone [1,2]. Collectively, these findings suggest that integrating aerobic components into RT, particularly within RBT frameworks, can induce broader physiological and neural adaptations. This multidimensional adaptation enhances neuromuscular control, metabolic efficiency, and mechanical output, thereby providing additional functional benefits for balance performance [43,44]. However, this integrative approach may introduce trade-offs at the muscular level. Evidence suggests that concurrent aerobic and resistance training can produce an “interference effect” [56,57]. Aerobic exercise activates the AMPK signaling pathway and promotes metabolic adaptation, whereas RT stimulates the mTOR pathway to enhance muscle protein synthesis and hypertrophy. When these stimuli occur in proximity, AMPK activation may inhibit mTOR activity, thereby attenuating RT-induced hypertrophy and strength development [58]. This mechanism may explain why multicomponent interventions based on RT showed smaller gains in ASMI compared with RT or RT + Nu. Muscle hypertrophy requires sufficient mechanical tension and metabolic stress to trigger anabolic signaling and protein synthesis [59]. When BT or AT components are incorporated into RT programs, the overall training intensity is typically lower, and the mechanical and metabolic stimuli are distributed across multiple domains, thereby reducing the anabolic drive and limiting ASMI improvements [60]. Despite this limitation, RT-based multicomponent interventions remain more effective for restoring functional performance [[43], [44], [45]]. Therefore, in clinical practice, exercise prescriptions should be tailored to individual functional deficits and rehabilitation goals, integrating RT, BT, and AT in an evidence-based and proportionally optimized manner. By coordinating neural control, metabolic adaptation, and mechanical loading within a strength-centered framework, clinicians can promote synergistic recovery of balance and mobility and maximize the overall therapeutic benefits of sarcopenia interventions.

Besides exercise interventions, protein supplementation has also been included in the comprehensive management strategies for sarcopenia. International guidelines conditionally recommend protein supplementation and its combination with exercise as supportive measures for nutritional management in older adults with sarcopenia, although the overall certainty of evidence remains low [17]. Our findings are consistent with this view. Although protein supplementation is highly accessible and low-cost, its benefits are modest. It produces only minor improvements in muscle strength and mass, a limited and clinically insignificant effect on gait speed, and no measurable impact on other functional outcomes. It is worth noting that current literature shows some inconsistencies regarding these outcomes [16,[61], [62], [63]], which may be attributed to differences in protein supplement types, dosages, or frequencies. Due to age-related anabolic resistance, older adults may require higher protein intake (≥1.2 g/kg/day) to maintain muscle mass [1,13,64]. Notably, protein supplementation is more appropriately positioned as a nutritional adjunct to resistance training, optimizing the anabolic environment to enhance training adaptations rather than serving as a standalone intervention. [65,66]. Importantly, the effect of protein supplementation combined with exercise is superior to protein or exercise alone [18,67]. For example, Cuyul-Vásquez et al. found that RT combined with protein supplementation significantly improved ASMI and handgrip strength compared to RT alone [18]. In our study, we observed similar advantages. RBT + Nu yielded significantly greater improvements in handgrip strength, gait speed, SPPB, and SMI compared with RBT alone. At the same time, RT combined with protein supplementation showed superior benefits over RT alone in gait speed and ASMI. Even after concurrent training, protein supplementation can promote myofibrillar protein synthesis, accelerate recovery, and, to some extent, mitigate the adverse effects of aerobic exercise on resistance adaptations [68,69]. This nutritional regulation provides a more favorable anabolic environment during concurrent training, potentially reducing the inhibitory signaling caused by metabolic competition [68]. Therefore, compared with ART, ART + Nu can achieve a more balanced adaptation between strength maintenance and fatigue resistance, leading to better performance in repetitive lower-limb tasks such as the 5-STS test. Furthermore, a recent systematic review and meta-analysis evaluated the effect of multicomponent protein supplementation combined with exercise, showing that supplementation with whey protein, leucine, and vitamin D significantly enhanced appendicular muscle mass. Especially when combined with exercise, the intervention showed highly significant effects on grip strength (SMD = 1.52) and SPPB score (SMD = 1.97) [15]. In our subgroup analysis, we further confirmed the added value of combining protein and amino acid supplementation with exercise. Specifically, RBT combined with protein and amino acids resulted in significantly greater improvements in grip strength, gait speed, SPPB, and ASMI compared with RBT combined with protein supplementation alone, thereby supporting the synergistic role of amino acids in nutritional interventions.

Notably, the current evidence is still insufficient to support EMS or WBV as substitutes for conventional exercise. For individuals who are unable to participate in voluntary training due to postoperative recovery, joint pain, or severe frailty, these approaches may serve as short-term or adjunctive strategies to help preserve muscle status and slow further decline. In our analysis, the observed effects of EMS or WBV were generally small, and their clinical relevance remains uncertain. Wu et al. [[8]] reported that WBV improved knee extension strength, TUG, and 5STS performance in older adults with sarcopenia, but its effects on muscle mass were limited. They further emphasized that small sample sizes and heterogeneous interventions limited the existing evidence. Moreover, several meta-analyses have suggested that EMS, either alone or in combination with protein supplementation, may improve muscle mass, strength, and physical function [9,70]. However, our analysis did not confirm these findings, which may be related to variations in stimulation parameters across studies. Importantly, we observed that EMS/EMS + Nu, WBV, and nutritional interventions achieved the highest adherence among all strategies. In the studies including these interventions, most participants were community-dwelling older adults with sarcopenia who were able to perform voluntary exercise. In contrast, a few studies involved institutionalized or functionally limited individuals. This may indicate that such low-burden interventions are feasible and potentially acceptable for older adults with sarcopenia. Nevertheless, the implementation of EMS and WBV remains constrained by reliance on specialized equipment and technical expertise [71]. Wu et al. [8] also emphasized that future studies should directly compare these modalities with conventional exercise. Our network meta-analysis provides indirect comparative evidence addressing this issue to some extent; however, high-quality randomized controlled trials with standardized diagnostic and intervention protocols are still needed to confirm their long-term feasibility, safety, and relative effectiveness.

Beyond structured exercise and nutritional interventions, spontaneous physical activity and sedentary behavior are increasingly recognized as important determinants of sarcopenia. Sánchez-Sánchez et al. [72] conducted a systematic review and meta-analysis. They reported that higher overall activity levels and greater participation in light-to-moderate activities were consistently associated with a reduced risk of sarcopenia and its key components, including muscle strength, muscle mass, and physical performance. Furthermore, sedentary behavior was independently associated with an increased risk of sarcopenia, even after adjustment for physical activity levels. In a separate systematic review, Mo et al. [73] focused on sedentary behavior. They found that prolonged sedentary time was associated with a higher risk of sarcopenia in older adults, whereas frequent interruptions of sedentary periods were associated with a lower risk. These factors may also contribute to the heterogeneity observed across intervention studies, as differences in baseline activity levels and sedentary behavior could affect responsiveness to exercise- and nutrition-based strategies. Future trials should therefore systematically assess spontaneous activity and sedentary behavior and, where feasible, adjust for these factors when evaluating intervention effectiveness.

### 4.3 Clinical implications

In this network meta-analysis, we found that multicomponent exercise interventions combined with protein supplementation were the most effective strategies for improving key outcomes in individuals with sarcopenia. Among the evaluated endpoints, RBT + Nu produced the most clinically meaningful gains in gait speed and SPPB. The increase in gait speed of 0.20 m/s exceeded the established MID of 0.10 m/s. It may allow some individuals to shift from a slow gait (<0.8 m/s, associated with disability and mortality) to a normal gait (≥1.0 m/s), thereby substantially improving functional status. Large cohort data suggest that such an improvement could translate into an approximately 24% reduction in all-cause mortality [74]. Similarly, the 3.6-point improvement in SPPB was sufficient for some individuals to progress from a score of 7–9 or 4–6 to ≥10, marking a transition from “frail/limited” to “good functional” status. Prior evidence indicates that individuals scoring ≤6 have nearly double the risk of all-cause mortality compared with those scoring ≥10, underscoring the prognostic importance of this gain [75]. These findings suggest that the observed improvements are not only statistically robust but also clinically meaningful, with the potential to enhance independence, improve quality of life, and reduce mortality risk. Furthermore, compared with conventional resistance training (RT or RT + Nu), RBT or RBT + Nu demonstrated broader benefits for physical function and should be considered the preferred approach when feasible.

For individuals unable to participate in conventional exercise, protein supplementation or WBV may represent adherence-friendly and practically feasible maintenance or transitional strategies. Existing low-certainty evidence suggests that WBV may provide clinically meaningful improvements in specific measures of physical function. In contrast, the impact of protein supplementation on most functional outcomes appears limited, although it may still contribute to modest gains in muscle strength and muscle mass. Overall, while the observed effect sizes are small and their clinical relevance remains uncertain, these interventions may help preserve muscle status and delay further decline. Future high-quality, standardized randomized controlled trials are required to establish their long-term efficacy and safety, and to define more clearly their role within clinical practice. It is noteworthy that the effectiveness of protein supplementation appears to be context-dependent. At the national level, benefits are more pronounced in developing countries, likely because lower baseline dietary protein intake elicits a stronger physiological response [76]. Protein supplementation should therefore be prioritized as a basic intervention strategy in resource-limited or nutritionally inadequate settings. At the care-setting level, marked differences in intervention effects have also been observed. While protein supplementation improved muscle strength, mass, and function among community-dwelling older adults, little benefit was seen in institutionalized populations, possibly due to more severe comorbidities, absorption disorders, or anabolic resistance. Accordingly, protein supplementation alone may be insufficient in long-term care institutions. A comprehensive approach combining exercise with protein supplementation should be considered to enhance efficacy and adapt to practical care contexts.

### 4.4 Strengths and limitations

The main strength of this systematic review lies in its comprehensive synthesis of the latest evidence regarding the effectiveness of exercise and protein supplementation for sarcopenia. Unlike previous reviews, this study included traditional voluntary exercise interventions (e.g., RT, AT) and systematically integrated simulated modalities (e.g., EMS, WBV), better reflecting the diversity of current intervention practices. Given the lack of unified international diagnostic criteria for sarcopenia, most existing studies are based on consensus definitions proposed by organizations such as EWGSOP2, AWGS, and FNIH. To enhance representativeness and generalizability, we included studies using any international consensus definition and those using non-consensus criteria. This inclusive strategy more accurately reflects the diversity of intervention populations in clinical practice and is supported by recent literature. Smith et al. [11] noted that variations in diagnostic criteria should not be a barrier to evaluating intervention effects in sarcopenia; instead, research should focus on intervention efficacy, implementation strategies, and patient adherence. To test the influence of our inclusion strategy, we conducted sensitivity analyses, which supported the robustness of our findings. Methodologically, we adopted a minimally contextualized framework within the GRADE system, using MID thresholds to classify the clinical relevance and certainty of the evidence. Additionally, we assessed adherence across interventions, helping to identify strategies with greater implementation potential when efficacy was comparable, thereby enhancing the clinical utility of our results.

Nevertheless, this study has several limitations. First, most of the evidence included was of low to very low certainty. Several RCTs did not adequately report allocation concealment or blinding, increasing uncertainty in risk of bias assessments. In addition, some trials had small sample sizes, potentially affecting the stability of the effect estimate. To assess the impact of these issues, we conducted sensitivity analyses excluding high-risk studies and those with fewer than 15 participants per group. The results supported the robustness of our findings. Second, due to the limited number of studies, we did not account for specific types of protein or amino acid supplements in our analysis, which may have contributed to heterogeneity. Future research should distinguish among supplement types and clarify their differential effects on muscle mass, strength, and physical performance to provide more targeted clinical guidance. Third, baseline levels of spontaneous physical activity and sedentary behavior were seldom assessed or adjusted for in the included trials, although they are recognized as potential determinants of sarcopenia. The absence of such data may have influenced intervention effect estimates and partly explains the heterogeneity observed across studies. This suggests that future trials should incorporate these factors into their design and analysis.

## 5 Conclusion

This network meta-analysis found that exercise interventions, protein supplementation, and their combination had beneficial effects on muscle strength, muscle mass, and physical performance in individuals with sarcopenia. Among them, multicomponent exercise combined with protein supplementation was the most effective strategy for improving key outcomes. In particular, RBT + Nu showed clinically meaningful improvements in gait speed, supported by high-certainty evidence. We therefore recommend RBT or RBT + Nu as a preferred option over traditional resistance training (RT or RT + Nu) to achieve broader and clinically meaningful functional improvements.

## CRediT authorship contribution statement

RX and SQ participated in the conception or design, acquisition, analysis, or interpretation of the data, and drafting and revising the manuscript. LD and WF participated in the acquisition, analysis, or interpretation of the data. WF participated in revising the manuscript and supervision. WF, SJ, and DY participated in the acquisition, analysis, or interpretation of the data. All authors have read and approved the final version of the manuscript and agree with the authorship order.

## Consent for publication

Not applicable.

## Ethics approval and consent to participate

Not applicable.

## Funding

Not applicable.

## Data availability statement

All data generated or analyzed during this study are included in this published article (and its supplementary files).

## Declaration of competing interest

The authors declare that they have no competing interests.
