# Supplementary material for: Comparative Effectiveness of Exercise and Protein-Based Interventions on Muscle Strength, Mass, and Function in Sarcopenia: A Systematic Review and Network Meta-Analysis
Source: J Nutr Health Aging. 2025 Nov 9;29(12):100718. doi: 10.1016/j.jnha.2025.100718 (PMC12639618; doi:10.1016/j.jnha.2025.100718)

Supplementary Appendix

**Table of contents**

*[Appendix 1: Search strategy 2](#_Toc1533)*

*[Appendix 2: Characteristics of included studies 9](#_Toc20599)*

*[Appendix 3: Risk of bias of randomized clinical trials 41](#_Toc14548)*

*[Appendix 4: Evaluation of inconsistency and heterogeneity 44](#_Toc18576)*

*[Appendix 5: Network maps and forest plots of secondary outcomes 49](#_Toc7769)*

*[Appendix 6: Minimally contextualized framework 59](#_Toc12215)*

*[Appendix 7: league table 62](#_Toc7773)*

*[Appendix 8: CINeMA Assessment 66](#_Toc6486)*

*[Appendix 9: Funnel Plots with Egger’s Test for Publication Bias 86](#_Toc1565)*

*[Appendix 10: Subgroup Analysis 91](#_Toc20320)*

*[Appendix11: Sensitivity analysis 113](#_Toc2749)*

# Appendix 1: Search strategy

**Table S1.1** Search strategy of Pubmed

| **#** | **Searches** |
| --- | --- |
| 1 | (((((((((((((((((((((((((Exercises[Title/Abstract]) OR (Exercise,Physical[Title/Abstract])) OR (Exercises, Physical[Title/Abstract])) OR (Physical Exercise[Title/Abstract])) OR (Physical Exercises[Title/Abstract])) OR (Physical Activity[Title/Abstract])) OR (Activities, Physical[Title/Abstract])) OR (Activity, Physical[Title/Abstract])) OR (Physical Activities[Title/Abstract])) OR (Exercise, Aerobic[Title/Abstract])) OR (Aerobic Exercise[Title/Abstract])) OR (Aerobic Exercises[Title/Abstract])) OR (Exercises, Aerobic[Title/Abstract])) OR (Exercise, Isometric[Title/Abstract])) OR (Exercises, Isometric[Title/Abstract])) OR (Isometric Exercises[Title/Abstract])) OR (Isometric Exercise[Title/Abstract])) OR (Acute Exercise[Title/Abstract])) OR (Acute Exercises[Title/Abstract])) OR (Exercise, Acute[Title/Abstract])) OR (Exercises, Acute[Title/Abstract])) OR (Exercise Training[Title/Abstract])) OR (Exercise Trainings[Title/Abstract])) OR (Training, Exercise[Title/Abstract])) OR (Trainings, Exercise[Title/Abstract])) OR (Exercise[MeSH Terms]) |
| 2 | (((((((((((((((((((((((Training, Resistance[Title/Abstract]) OR (Strength Training[Title/Abstract])) OR (Training, Strength[Title/Abstract])) OR (Weight-Lifting Strengthening Program[Title/Abstract])) OR (Strengthening Programs, Weight-Lifting[Title/Abstract])) OR (Strengthening Program, Weight-Lifting[Title/Abstract])) OR (Weight Lifting Strengthening Program[Title/Abstract])) OR (Weight-Lifting Strengthening Programs[Title/Abstract])) OR (Weight-Lifting Exercise Program[Title/Abstract])) OR (Exercise Programs, Weight-Lifting[Title/Abstract])) OR (Exercise Program, Weight-Lifting[Title/Abstract])) OR (Weight Lifting Exercise Program[Title/Abstract])) OR (Weight-Lifting Exercise Programs[Title/Abstract])) OR (Weight-Bearing Strengthening Program[Title/Abstract])) OR (Strengthening Programs, Weight-Bearing[Title/Abstract])) OR (Strengthening Program, Weight-Bearing[Title/Abstract])) OR (Weight Bearing Strengthening Program[Title/Abstract])) OR (Weight-Bearing Strengthening Programs[Title/Abstract])) OR (Weight-Bearing Exercise Program[Title/Abstract])) OR (Exercise Programs, Weight-Bearing[Title/Abstract])) OR (Exercise Program, Weight-Bearing[Title/Abstract])) OR (Weight Bearing Exercise Program[Title/Abstract])) OR (Weight-Bearing Exercise Programs[Title/Abstract])) OR (Resistance training[MeSH Terms]) |
| 3 | ((((((((Cardiovascular Exercise[Title/Abstract]) OR (Endurance Training[Title/Abstract])) OR (Walking[Title/Abstract])) OR (Running[Title/Abstract])) OR (Cycling[Title/Abstract])) OR (Swimming[Title/Abstract])) OR (Dancing[Title/Abstract])) OR (combined training[Title/Abstract])) OR (Concurrent Training[Title/Abstract]) |
| 4 | ((((((((((((High Intensity Interval Training[Title/Abstract]) OR (High-Intensity Interval Trainings[Title/Abstract])) OR (Interval Training, High-Intensity[Title/Abstract])) OR (Interval Trainings, High-Intensity[Title/Abstract])) OR (Training, High-Intensity Interval[Title/Abstract])) OR (Trainings, High-Intensity Interval[Title/Abstract])) OR (High-Intensity Intermittent Exercise[Title/Abstract])) OR (Exercise, High-Intensity Intermittent[Title/Abstract])) OR (Exercises, High-Intensity Intermittent[Title/Abstract])) OR (High-Intensity Intermittent Exercises[Title/Abstract])) OR (Sprint Interval Training[Title/Abstract])) OR (Sprint Interval Trainings[Title/Abstract])) OR (High-Intensity Interval Training[MeSH Terms]) |
| 5 | ((((((Circuit-Based Exercise[MeSH Terms]) OR (Circuit Based Exercise[Title/Abstract])) OR (Circuit-Based Exercises[Title/Abstract])) OR (Exercise, Circuit-Based[Title/Abstract])) OR (Exercises, Circuit-Based[Title/Abstract])) OR (Circuit Training[Title/Abstract])) OR (Training, Circuit[Title/Abstract]) |
| 6 | (((((((((((((((((Chinese exercise[Title/Abstract]) OR (traditional exercise[Title/Abstract])) OR (traditional chinese medicine[Title/Abstract])) OR (chinese traditional exercise[Title/Abstract])) OR (traditional chinese exercise[Title/Abstract])) OR (traditional exercise[Title/Abstract])) OR (traditional Kungfu[Title/Abstract])) OR (Kungfu[Title/Abstract])) OR (Shadowboxing[Title/Abstract])) OR (Five-animal exercises[Title/Abstract])) OR (Wuqinxi[Title/Abstract])) OR (Five animal frolics[Title/Abstract])) OR (Five-animal boxing[Title/Abstract])) OR (Baduanjin[Title/Abstract])) OR (Yijinjing[Title/Abstract])) OR (liuzijue[Title/Abstract])) OR (((Qigong[MeSH Terms]) OR (Ch'i Kung[Title/Abstract])) OR (Qi Gong[Title/Abstract]))) OR ((((((((((taiji[MeSH Terms]) OR (Tai-ji[Title/Abstract])) OR (Tai Chi[Title/Abstract])) OR (Chi, Tai[Title/Abstract])) OR (Tai Chi Chuan[Title/Abstract])) OR (Taijiquan[Title/Abstract])) OR (T'ai Chi[Title/Abstract])) OR (Tai Ji Quan[Title/Abstract])) OR (Ji Quan, Tai[Title/Abstract])) OR (Quan, Tai Ji[Title/Abstract])) |
| 7 | (((Mind-body exercises[Title/Abstract]) OR (Yoga[MeSH Terms])) OR (dance[Title/Abstract])) OR (Pilates[Title/Abstract]) |
| 8 | ((((((Balance training[Title/Abstract]) OR (Balance exercise[Title/Abstract])) OR (Balance intervention[Title/Abstract])) OR (Postural control[Title/Abstract])) OR (Stability training[Title/Abstract])) OR (Proprioception training[Title/Abstract])) OR (Motor control training[Title/Abstract]) |
| 9 | (((((Blood Flow Restriction Therapy[MeSH Terms]) OR (Blood Flow Restriction Training[Title/Abstract])) OR (Blood Flow Restriction Exercise[Title/Abstract])) OR (BFR Therapy[Title/Abstract])) OR (BFR Therapies[Title/Abstract])) OR (Therapy, BFR[Title/Abstract]) |
| 10 | (((((vibration[Title/Abstract]) OR (whole body vibration[Title/Abstract])) OR (whole body vibration training[Title/Abstract])) OR (Vibration exercise[Title/Abstract])) OR (whole-body electromyostimulation[Title/Abstract])) OR (Electrical Muscle Stimulation[Title/Abstract]) |
| 11 | (((Sarcopenia[MeSH Terms]) OR (sarcopenia[Title/Abstract])) OR (Muscle loss[Title/Abstract])) OR (Sarcopenias[Title/Abstract]) |
| 12 | ((((((((((((Whey Proteins[MeSH Terms]) OR (Proteins, Whey[Title/Abstract])) OR (Protein, Whey[Title/Abstract])) OR (Whey Protein[Title/Abstract])) OR (whey supplementation[Title/Abstract])) OR (whey protein supplementation[Title/Abstract])) OR (whey intake[Title/Abstract])) OR (whey protein isolate[Title/Abstract])) OR (whey protein concentrate[Title/Abstract])) OR (milk proteins[Title/Abstract])) OR (milk protein[Title/Abstract])) OR (dairy proteins[Title/Abstract])) OR (dairy protein[Title/Abstract]) |
| 13 | (((((((Protein[MeSH Terms]) OR (Dietary Supplements[MeSH Terms])) OR (Amino Acids[MeSH Terms])) OR (Dietary Supplement[Title/Abstract])) OR (Protein supplement[Title/Abstract])) OR (Supplements, Dietary[Title/Abstract])) OR (Dietary Supplementations[Title/Abstract])) OR (Supplementations, Dietary[Title/Abstract]) |
| 14 | ((((((beta-hydroxyisovaleric acid[MeSH Terms]) OR (beta hydroxy beta methylbutyrate[Title/Abstract])) OR (beta-hydroxy beta-methylbutyrate[Title/Abstract])) OR (3-hydroxyisovaleric acid[Title/Abstract])) OR (beta-hydroxy-beta-methylbutyrate[Title/Abstract])) OR (HMB-d6[Title/Abstract])) OR (HMB[Title/Abstract]) |
| 15 | ((((((Amino Acids, Essential[MeSH Terms]) OR (Acids, Essential Amino[Title/Abstract])) OR (Essential Amino Acid[Title/Abstract])) OR (Acid, Essential Amino[Title/Abstract])) OR (Amino Acid, Essential[Title/Abstract])) OR (Essential Amino Acids[Title/Abstract])) OR (EAA[Title/Abstract]) |
| 16 | ((((Leucine[MeSH Terms]) OR (Leucine, L-Isomer[Title/Abstract])) OR (Leucine, L Isomer[Title/Abstract])) OR (L-Isomer Leucine[Title/Abstract])) OR (L-Leucine[Title/Abstract]) |
| 17 | ((((((((Amino Acids, Branched-Chain[MeSH Terms]) OR (Acids, Branched-Chain Amino[Title/Abstract])) OR (Branched-Chain Amino Acids[Title/Abstract])) OR (Branched-Chain Amino Acid[Title/Abstract])) OR (Acid, Branched-Chain Amino[Title/Abstract])) OR (Amino Acid, Branched-Chain[Title/Abstract])) OR (Branched Chain Amino Acid[Title/Abstract])) OR (Amino Acids, Branched Chain[Title/Abstract])) OR (BCAA[Title/Abstract]) |
| 18 | (((((((((((((Therapy, Nutrition[MeSH Terms]) OR (Therapy, Nutrition[Title/Abstract])) OR (Medical Nutrition Therapy[Title/Abstract])) OR (Nutrition Therapy, Medical[Title/Abstract])) OR (Therapy, Medical Nutrition[Title/Abstract])) OR (Diet Therapy[MeSH Terms])) OR (Diet Therapies[Title/Abstract])) OR (Therapy, Diet[Title/Abstract])) OR (Dietary Modification[Title/Abstract])) OR (Dietary Modifications[Title/Abstract])) OR (Diet Modification[Title/Abstract])) OR (Diet Modifications[Title/Abstract])) OR (Modification, Diet[Title/Abstract])) OR (Diet, Food, and Nutrition[MeSH Terms]) |
| 19 | #1 OR #2 OR #3 OR #4 OR #5 OR #6 OR #7 OR #8 OR #9 OR #10 OR #12 OR #13 OR #14 OR #15 OR #16 OR #17 OR #18 |
| 20 | #11 AND #19 AND #20 |

**Table S1.2** Search strategy of Web of Science

| **#** | **Searches** |
| --- | --- |
| 1 | TS=("Sarcopenia" OR "Sarcopenias" OR "Muscle loss") |
| 2 | TS=("Exercise" OR "Exercises" OR "Exercise, Physical" OR "Exercises, Physical" OR "Physical Exercise" OR "Physical Exercises" OR "Physical Activity" OR "Activities, Physical" OR "Activity, Physical" OR "Physical Activities" OR "Exercise, Aerobic" OR "Aerobic Exercise" OR "Aerobic Exercises" OR "Exercises, Aerobic" OR "Exercise, Isometric" OR "Exercises, Isometric" OR "Isometric Exercises" OR "Isometric Exercise" OR "Acute Exercise" OR "Acute Exercises" OR "Exercise, Acute" OR "Exercises, Acute" OR "Exercise Training" OR "Exercise Trainings" OR "Training, Exercise" OR "Trainings, Exercise") |
| 3 | TS=("Resistance training" OR "Training, Resistance" OR "Strength Training" OR "Training, Strength" OR "Weight-Lifting Strengthening Program" OR "Strengthening Programs, Weight-Lifting" OR "Strengthening Program, Weight-Lifting" OR "Weight Lifting Strengthening Program" OR "Weight-Lifting Strengthening Programs" OR "Weight-Lifting Exercise Program" OR "Exercise Programs, Weight-Lifting" OR "Exercise Program, Weight-Lifting" OR "Weight Lifting Exercise Program" OR "Weight-Lifting Exercise Programs" OR "Weight-Bearing Strengthening Program" OR "Strengthening Programs, Weight-Bearing" OR "Strengthening Program, Weight-Bearing" OR "Weight Bearing Strengthening Program" OR "Weight-Bearing Strengthening Programs" OR "Weight-Bearing Exercise Program" OR "Exercise Programs, Weight-Bearing" OR "Exercise Program, Weight-Bearing" OR "Weight Bearing Exercise Program" OR "Weight-Bearing Exercise Programs") |
| 4 | TS=("Cardiovascular Exercise" OR "Endurance Training" OR "Walking" OR "Running" OR "Cycling" OR "Swimming" OR "Dancing" OR "combined training" OR "Concurrent Training") |
| 5 | TS=("High-Intensity Interval Training" OR "High Intensity Interval Training" OR "High-Intensity Interval Trainings" OR "Interval Training, High-Intensity" OR "Interval Trainings, High-Intensity" OR "Training, High-Intensity Interval" OR "Trainings, High-Intensity Interval" OR "High-Intensity Intermittent Exercise" OR "Exercise, High-Intensity Intermittent" OR "Exercises, High-Intensity Intermittent" OR "High-Intensity Intermittent Exercises" OR "Sprint Interval Training" OR "Sprint Interval Trainings") |
| 6 | TS=("Circuit-Based Exercise" OR "Circuit Based Exercise" OR "Circuit-Based Exercises" OR "Exercise, Circuit-Based" OR "Exercises, Circuit-Based" OR "Circuit Training" OR "Training, Circuit") |
| 7 | TS=("Chinese exercise" OR "traditional exercise" OR "traditional chinese medicine" OR "chinese traditional exercise" OR "traditional chinese exercise" OR "traditional Kungfu" OR "Kungfu" OR "Shadowboxing" OR "Five-animal exercises" OR "Wuqinxi" OR "Five animal frolics" OR "Five-animal boxing" OR "Baduanjin" OR "Yijinjing" OR "liuzijue" OR "Qigong" OR "Ch'i Kung" OR "Qi Gong" OR "taiji" OR "Tai-ji" OR "Tai Chi" OR "Chi, Tai" OR "Tai Chi Chuan" OR "Taijiquan" OR "T'ai Chi" OR "Tai Ji Quan" OR "Ji Quan, Tai" OR "Quan, Tai Ji") |
| 8 | TS=("Mind-body exercises" OR "yoga" OR "dance" OR "Pilates") |
| 9 | TS=("Balance training" OR "Balance exercise" OR "Balance intervention" OR "Postural control" OR "Stability training" OR "Proprioception training" OR "Motor control training") |
| 10 | TS=("Blood Flow Restriction Therapy" OR "Blood Flow Restriction Training" OR "Blood Flow Restriction Exercise" OR "BFR Therapy" OR "BFR Therapies" OR "Therapy, BFR") |
| 11 | TS=(vibration OR "whole body vibration" OR "whole body vibration training" OR "vibration exercise" OR "whole-body electromyostimulation" OR "electrical muscle stimulation") |
| 12 | TS=("Protein" OR "Dietary Supplements" OR "Amino Acids" OR "Dietary Supplement" OR "Protein supplement" OR "Supplements, Dietary" OR "Dietary Supplementations" OR "Supplementations, Dietary") |
| 13 | TS=("Whey Proteins" OR "Proteins, Whey" OR "Protein, Whey" OR "Whey Protein" OR "whey supplementation" OR "whey protein supplementation" OR "whey intake" OR "whey protein isolate" OR "whey protein concentrate" OR "milk proteins" OR "milk protein" OR "dairy proteins" OR "dairy protein") |
| 14 | TS=("Amino Acids, Essential" OR "Acids, Essential Amino" OR "Essential Amino Acid" OR "Acid, Essential Amino" OR "Amino Acid, Essential" OR "Essential Amino Acids" OR "EAA") |
| 15 | TS=("Leucine" OR "Leucine, L-Isomer" OR "Leucine, L Isomer" OR "L-Isomer Leucine" OR "L-Leucine") |
| 16 | TS=("Therapy, Nutrition" OR "Medical Nutrition Therapy" OR "Nutrition Therapy, Medical" OR "Therapy, Medical Nutrition" OR "Diet Therapy" OR "Diet Therapies" OR "Therapy, Diet" OR "Dietary Modification" OR "Dietary Modifications" OR "Diet Modification" OR "Diet Modifications" OR "Modification, Diet" OR "Diet, Food, and Nutrition") |
| 17 | TS=("beta-hydroxyisovaleric acid" OR "beta hydroxy beta methylbutyrate" OR "beta-hydroxy beta-methylbutyrate" OR "3-hydroxyisovaleric acid" OR "beta-hydroxy-beta-methylbutyrate" OR "HMB-d6" OR "HMB") |
| 18 | TS=("Amino Acids, Branched-Chain" OR "Acids, Branched-Chain Amino" OR "Branched-Chain Amino Acids" OR "Branched-Chain Amino Acid" OR "Acid, Branched-Chain Amino" OR "Amino Acid, Branched-Chain" OR "Branched Chain Amino Acid" OR "Amino Acids, Branched Chain" OR "BCAA") |
| 19 | #2 OR #3 OR #4 OR #5 OR #6 OR #7 OR #8 OR #9 OR #10 OR #11 OR #12 OR #13 OR #14 OR #15 OR #16 OR #17 OR #18 |
| 20 | #1 AND #19 |

**Table S1.3** Search strategy of Cochrane Central Register of Controlled Trials

| **#** | **Searches** |
| --- | --- |
| 1 | MeSH descriptor: [Sarcopenia] explode all trees |
| 2 | MeSH descriptor: [High-Intensity Interval Training] explode all trees |
| 3 | MeSH descriptor: [Exercise] explode all trees |
| 4 | MeSH descriptor: [Circuit-Based Exercise] explode all trees |
| 5 | MeSH descriptor: [Resistance Training] explode all trees |
| 6 | MeSH descriptor: [Tai Ji] explode all trees |
| 7 | MeSH descriptor: [Qigong] explode all trees |
| 8 | MeSH descriptor: [Yoga] explode all trees |
| 9 | MeSH descriptor: [Exercise Movement Techniques] explode all trees |
| 10 | MeSH descriptor: [Blood Flow Restriction Therapy] explode all trees |
| 11 | ("Balance training":ti,ab,kw OR "Balance exercise":ti,ab,kw OR "Balance intervention":ti,ab,kw OR "Postural control":ti,ab,kw OR "Stability training":ti,ab,kw OR "Proprioception training":ti,ab,kw OR "Motor control training":ti,ab,kw) |
| 12 | ("vibration":ti,ab,kw OR "whole body vibration":ti,ab,kw OR "whole body vibration training":ti,ab,kw OR "vibration exercise":ti,ab,kw OR "whole-body electromyostimulation":ti,ab,kw OR "electrical muscle stimulation":ti,ab,kw OR "aerobic exercise":ti,ab,kw OR "aerobic training":ti,ab,kw OR "cardiovascular exercise":ti,ab,kw OR "endurance training":ti,ab,kw OR "walking":ti,ab,kw OR "running":ti,ab,kw OR "cycling":ti,ab,kw OR "swimming":ti,ab,kw OR "dancing":ti,ab,kw OR "combined training":ti,ab,kw OR "concurrent training":ti,ab,kw OR "mind-body exercises":ti,ab,kw OR "yoga":ti,ab,kw OR "dance":ti,ab,kw OR "pilates":ti,ab,kw OR "traditional chinese medicine":ti,ab,kw OR "chinese traditional exercise":ti,ab,kw OR "traditional exercise":ti,ab,kw OR "traditional kungfu":ti,ab,kw OR "kungfu":ti,ab,kw OR "shadowboxing":ti,ab,kw OR "wuqinxi":ti,ab,kw OR "five-animal exercises":ti,ab,kw OR "five animal frolics":ti,ab,kw OR "five-animal boxing":ti,ab,kw OR "baduanjin":ti,ab,kw OR "yijinjing":ti,ab,kw OR "liuzijue":ti,ab,kw) |
| 13 | MeSH descriptor: [Proteins] explode all trees |
| 14 | MeSH descriptor: [Amino Acids] explode all trees |
| 15 | MeSH descriptor: [Whey Proteins] explode all trees |
| 16 | MeSH descriptor: [beta-hydroxyisovaleric acid] explode all trees |
| 17 | MeSH descriptor: [Amino Acids, Essential] explode all trees |
| 18 | MeSH descriptor: [Leucine] explode all trees |
| 19 | MeSH descriptor: [Therapy, Nutrition] explode all trees |
| 20 | MeSH descriptor: [Diet Therapy] explode all trees |
| 21 | MeSH descriptor: [Diet, Food, and Nutrition] explode all trees |
| 22 | MeSH descriptor: [Amino Acids, Branched-Chain] explode all trees |
| 23 | #2 OR #3 OR #4 OR #5 OR #6 OR #7 OR #8 OR #9 OR #10 OR #11 OR #12 OR #13 OR #14 OR #15 OR #16 OR #17 OR #18 OR #19 OR #20 OR #21 OR #22 |
| 24 | #1 AND #23 |

**Table 1.4** Search strategy of Embase

| **#** | **Searches** |
| --- | --- |
| 1 | 'Sarcopenia'/exp OR sarcopenia:ab,ti OR 'Muscle loss':ab,ti OR sarcopenias:ab,ti |
| 2 | 'Exercise'/exp OR 'Exercise':ab,ti OR 'Exercises':ab,ti OR 'Exercise, Physical':ab,ti OR 'Exercises, Physical':ab,ti OR 'Physical Exercise':ab,ti OR 'Physical Exercises':ab,ti OR 'Physical Activity':ab,ti OR 'Activities, Physical':ab,ti OR 'Activity, Physical':ab,ti OR 'Physical Activities':ab,ti OR 'Exercise, Aerobic':ab,ti OR 'Aerobic Exercise':ab,ti OR 'Aerobic Exercises':ab,ti OR 'Exercises, Aerobic':ab,ti OR 'Exercise, Isometric':ab,ti OR 'Exercises, Isometric':ab,ti OR 'Isometric Exercises':ab,ti OR 'Isometric Exercise':ab,ti OR 'Acute Exercise':ab,ti OR 'Acute Exercises':ab,ti OR 'Exercise, Acute':ab,ti OR 'Exercises, Acute':ab,ti OR 'Exercise Training':ab,ti OR 'Exercise Trainings':ab,ti OR 'Training, Exercise':ab,ti OR 'Trainings, Exercise':ab,ti |
| 3 | 'Resistance training'/exp OR 'Resistance training':ab,ti OR 'Training, Resistance':ab,ti OR 'Strength Training':ab,ti OR 'Training, Strength':ab,ti OR 'Weight-Lifting Strengthening Program':ab,ti OR 'Strengthening Programs, Weight-Lifting':ab,ti OR 'Strengthening Program, Weight-Lifting':ab,ti OR 'Weight Lifting Strengthening Program':ab,ti OR 'Weight-Lifting Strengthening Programs':ab,ti OR 'Weight-Lifting Exercise Program':ab,ti OR 'Exercise Programs, Weight-Lifting':ab,ti OR 'Exercise Program, Weight-Lifting':ab,ti OR 'Weight Lifting Exercise Program':ab,ti OR 'Weight-Lifting Exercise Programs':ab,ti OR 'Weight-Bearing Strengthening Program':ab,ti OR 'Strengthening Programs, Weight-Bearing':ab,ti OR 'Strengthening Program, Weight-Bearing':ab,ti OR 'Weight Bearing Strengthening Program':ab,ti OR 'Weight-Bearing Strengthening Programs':ab,ti OR 'Weight-Bearing Exercise Program':ab,ti OR 'Exercise Programs, Weight-Bearing':ab,ti OR 'Exercise Program, Weight-Bearing':ab,ti OR 'Weight Bearing Exercise Program':ab,ti OR 'Weight-Bearing Exercise Programs':ab,ti |
| 4 | 'Cardiovascular Exercise':ab,ti OR 'Endurance Training':ab,ti OR 'Walking':ab,ti OR 'Running':ab,ti OR 'Cycling':ab,ti OR 'Swimming':ab,ti OR 'Dancing':ab,ti OR 'combined training':ab,ti OR 'Concurrent Training':ab,ti |
| 5 | 'High-Intensity Interval Training'/exp OR 'High Intensity Interval Training':ab,ti OR 'High-Intensity Interval Trainings':ab,ti OR 'Interval Training, High-Intensity':ab,ti OR 'Interval Trainings, High-Intensity':ab,ti OR 'Training, High-Intensity Interval':ab,ti OR 'Trainings, High-Intensity Interval':ab,ti OR 'High-Intensity Intermittent Exercise':ab,ti OR 'Exercise, High-Intensity Intermittent':ab,ti OR 'Exercises, High-Intensity Intermittent':ab,ti OR 'High-Intensity Intermittent Exercises':ab,ti OR 'Sprint Interval Training':ab,ti OR 'Sprint Interval Trainings':ab,ti |
| 6 | 'Circuit-Based Exercise'/exp OR 'Circuit Based Exercise':ab,ti OR 'Circuit-Based Exercises':ab,ti OR 'Exercise, Circuit-Based':ab,ti OR 'Exercises, Circuit-Based':ab,ti OR 'Circuit Training'/exp OR 'Training, Circuit':ab,ti |
| 7 | 'chinese exercise':ab,ti OR 'traditional exercise':ab,ti OR 'traditional chinese medicine':ab,ti OR 'chinese traditional exercise':ab,ti OR 'traditional chinese exercise':ab,ti OR 'traditional kungfu':ab,ti OR 'kungfu':ab,ti OR 'shadowboxing':ab,ti OR 'five-animal exercises':ab,ti OR 'wuqinxi':ab,ti OR 'five animal frolics':ab,ti OR 'five-animal boxing':ab,ti OR 'baduanjin':ab,ti OR 'yijinjing':ab,ti OR 'liuzijue':ab,ti OR 'qigong'/exp OR 'chi kung':ab,ti OR 'qi gong':ab,ti OR 'taiji' OR 'tai-ji':ab,ti OR 'chi, tai':ab,ti OR 'tai chi chuan':ab,ti OR 'taijiquan':ab,ti OR 'tai chi':ab,ti OR 'tai ji quan':ab,ti OR 'ji quan, tai':ab,ti OR 'quan, tai ji':ab,ti |
| 8 | 'Mind-body exercises':ab,ti OR 'yoga'/exp OR 'dance':ab,ti OR 'Pilates'/exp |
| 9 | ('Balance training':ab,ti OR 'Balance exercise':ab,ti OR 'Balance intervention':ab,ti OR 'Postural control':ab,ti OR 'Stability training':ab,ti OR 'Proprioception training':ab,ti OR 'Motor control training':ab,ti) |
| 10 | ('Blood Flow Restriction Therapy'/exp OR 'Blood Flow Restriction Training':ab,ti OR 'Blood Flow Restriction Exercise':ab,ti OR 'BFR Therapy':ab,ti OR 'BFR Therapies':ab,ti OR 'Therapy, BFR':ab,ti) |
| 11 | ("vibration":ab,ti OR "whole body vibration":ab,ti OR "whole body vibration training":ab,ti OR "vibration exercise":ab,ti OR "whole-body electromyostimulation":ab,ti OR "electrical muscle stimulation":ab,ti) |
| 12 | 'Whey Proteins'/exp OR 'Proteins, Whey':ab,ti OR 'Protein, Whey':ab,ti OR 'Whey Protein':ab,ti OR 'whey supplementation':ab,ti OR 'whey protein supplementation':ab,ti OR 'whey intake':ab,ti OR 'whey protein isolate':ab,ti OR 'whey protein concentrate':ab,ti OR 'milk proteins':ab,ti OR 'milk protein':ab,ti OR 'dairy proteins':ab,ti OR 'dairy protein':ab,ti |
| 13 | 'beta-hydroxyisovaleric acid'/exp OR 'beta hydroxy beta methylbutyrate':ab,ti OR 'beta-hydroxy beta-methylbutyrate':ab,ti OR '3-hydroxyisovaleric acid':ab,ti OR 'beta-hydroxy-beta-methylbutyrate':ab,ti OR 'HMB-d6':ab,ti OR 'HMB':ab,ti |
| 14 | 'Amino Acids, Essential'/exp OR 'Acids, Essential Amino':ab,ti OR 'Essential Amino Acid':ab,ti OR 'Acid, Essential Amino':ab,ti OR 'Amino Acid, Essential':ab,ti OR 'Essential Amino Acids':ab,ti OR 'EAA':ab,ti |
| 15 | 'Leucine'/exp OR 'Leucine, L-Isomer':ab,ti OR 'Leucine, L Isomer':ab,ti OR 'L-Isomer Leucine':ab,ti OR 'L-Leucine':ab,ti |
| 16 | 'Amino Acids, Branched-Chain'/exp OR 'Acids, Branched-Chain Amino':ab,ti OR 'Branched-Chain Amino Acids':ab,ti OR 'Branched-Chain Amino Acid':ab,ti OR 'Acid, Branched-Chain Amino':ab,ti OR 'Amino Acid, Branched-Chain':ab,ti OR 'Branched Chain Amino Acid':ab,ti OR 'Amino Acids, Branched Chain':ab,ti OR 'BCAA':ab,ti |
| 17 | 'Therapy, Nutrition'/exp OR 'Therapy, Nutrition':ab,ti OR 'Medical Nutrition Therapy':ab,ti OR 'Nutrition Therapy, Medical':ab,ti OR 'Therapy, Medical Nutrition':ab,ti OR 'Diet Therapy'/exp OR 'Diet Therapies':ab,ti OR 'Therapy, Diet':ab,ti OR 'Dietary Modification':ab,ti OR 'Dietary Modifications':ab,ti OR 'Diet Modification':ab,ti OR 'Diet Modifications':ab,ti OR 'Modification, Diet':ab,ti OR 'Diet, Food, and Nutrition'/exp |
| 18 | 'dietary supplement'/exp OR 'dietary supplement':ab,ti OR 'protein supplement':ab,ti OR 'supplements, dietary':ab,ti OR 'dietary supplementations':ab,ti OR 'supplementations, dietary':ab,ti OR 'protein'/exp OR 'protein':ab,ti OR 'amino acid'/exp OR 'amino acid':ab,ti |
| 19 | #2 OR #3 OR #4 OR #5 OR #6 OR #7 OR #8 OR #9 OR #10 OR #11 OR #12 OR #13 OR #14 OR #15 OR #16 OR #17 OR #18 |
| 20 | #1 AND #19 |

# Appendix 2: Characteristics of included studies

**Table S2.1:** Baseline of characteristics of included studies

| Study ID | Group | Age (Mean±SD) | Sample Size | Period (weeks) | Exercise Frequency (times/week) | Exercise Duration (min) | Exercise  Intensity | Nutritional Intervention Details | Diagnostic Criteria | Country | Setting |
| --- | --- | --- | --- | --- | --- | --- | --- | --- | --- | --- | --- |
| Achison et al 2022^1^ | Nu | 78.3±5.9 | 72 | 52 |  |  |  | Oral leucine powder at a dose of 2.5 g per serving, three times daily (total 7.5 g/day) | EWGSOP 2010 | UK | Institution |
|  | CG | 79.3±6.1 | 73 | 52 |  |  |  | Oral lactose placebo powder, three times daily (total 7.5 g/day) | EWGSOP 2010 | UK | Institution |
| Alemán-Mateo et al2012^2^ | Nu | 75.4±5.0 | 20 | 12 |  |  |  | Daily intake of 21 g whey cheese with meals, providing 15.7 g of protein and 6 g of essential amino acids | Study-defined criteria | Sonora | Community |
|  | CG | 76.7±5.8 | 20 | 12 |  |  |  | Maintained usual dietary habits | Study-defined criteria | Sonora | Community |
| Amasene et al 2019^3^ | RBT+Nu | 82.9±5.67 | 21 | 12 | 2 | 60 | 50%-70% 1-RM | Leucine-enriched whey protein supplement (21 g protein + 3 g leucine) consumed post-resistance training (2 times/week) | EWGSOP2 | Spain | Institution |
|  | RBT | 81.2±6.14 | 20 | 12 | 2 | 60 | 50%-70% 1RM | Isoenergetic placebo (no protein) consumed post-resistance training (2 times/week) | EWGSOP2 | Spain | Institution |
| Amasene et al 2021^4^ | RBT | 81.2±6.14 | 13 | 12 | 2 | 60 | 50%-70% 1RM | Whey protein supplement providing 21 g protein and 6 g leucine consumed immediately after resistance training (2 times/week) | EWGSOP2 | Spain | Institution |
|  | RBT+Nu | 82.9±5.67 | 15 | 12 | 2 | 60 | 50%-70% 1RM | Isoenergetic placebo | EWGSOP2 | Spain | Institution |
| Bagheri et al2020^5^ | ART | 63.8±3.6 | 10 | 8 | 3 | 45-80 | RT: 40% 1RM-75% 1RM AT: 55% HRmax-70% HRmax |  | Study-defined criteria | Iran | Institution |
|  | ART | 61.1±3.8 | 10 | 8 |  | 45-80 | RT: 40% 1RM-75% 1RM AT: 55% HRmax-70% HRmax |  | Study-defined criteria | Iran | Institution |
|  | CG | 65±3.9 | 10 | 8 |  |  |  | Daily activity maintained | Study-defined criteria | Iran | Institution |
| Balachandran et al2014^6^ | CT | 71.6±7.8 | 8 | 15 | 2 | 45 | 50%-80% 1RM |  | EWGSOP 2010 | USA | Community |
|  | RT | 71±8.2 | 9 | 15 | 2 | 60 | 70% 1RM |  | EWGSOP 2010 | USA | Community |
| Bauer et al 2015^7^ | Nu | 77.3±6.7 | 172 | 13 |  |  |  | Daily oral intake of vitamin D (800 IU) and leucine-enriched whey protein (20 g protein + 3 g leucine) | EWGSOP 2010 | Germany | Institution |
|  | CG | 78.1±7 | 158 | 13 |  |  |  | Isoenergetic, isovolumetric placebo without vitamin D or protein | EWGSOP 2010 | Germany | Institution |
| Bellomo et al2013^8^ | RT | 70.9±5.2 | 10 | 12 | 2 | 30 | 60%-85% 1RM |  | CDC | Italy | Institution |
|  | WBV | 70.9±5.2 | 10 | 12 | 2 | 15 | 300 Hz |  | CDC | Italy | Institution |
|  | CG | 70.9±5.2 | 10 | 12 |  |  |  |  | CDC | Italy | Institution |
| Bernabei et al2022^9^ | ARBT+Nu | 73.9±5.9 | 605 | 78 | 6 | 45-60 | AT:RPE 13 (20) RT：RPE 15-16 (20) | Daily protein intake ≥1.0-1.2/kg/day | Study-defined criteria | Italy | Institution |
|  | CG | 79.2±5.8 | 600 | 78 | 6 |  |  | Received health education only | Study-defined criteria | Italy | Institution |
|  | ARBT+Nu | 78.3±5.7 | 155 | 78 |  | 45-60 |  | Daily protein intake ≥1.0-1.2/kg/day | Study-defined criteria | Italy | Institution |
|  | CG | 77.1±5.4 | 159 | 78 |  |  |  | Received health education only | Study-defined criteria | Italy | Institution |
| Björkman et al2019^10^ | ART | 84.0±3.9 | 73 | 52 |  |  |  | Received health education only | EWGSOP 2010 | Finland | Community |
|  | ART+Nu | 83.6±4.7 | 73 | 52 |  |  |  | Daily intake of 20 g whey protein twice daily | EWGSOP 2010 | Finland | Community |
| Bo Yacong et al2019^11^ | CG | 74.83±5.94 | 30 | 26 |  |  |  | Isoenergetic placebo supplement | AWGS2014 | China | Institution |
|  | Nu | 73.23±6.52 | 30 | 26 |  |  |  | nutritional supplement twice daily (40 g per serving reconstituted in 100–150 mL water), providing a total of 44 g protein (57.5% of energy, primarily whey), 1404 IU vitamin D, and 218 mg vitamin E per day | AWGS2014 | China | Institution |
| Chalé et al2013^12^ | RT+Nu | 78.0±4.0 | 42 | 26 | 3 |  | 80% 1RM | Daily intake of 40 g whey protein | Study-defined criteria | Boston | Community |
|  | RT | 77.3±3.28 | 38 | 26 | 3 |  | 80% 1RM | Isoenergetic placebo | Study-defined criteria | Boston | Community |
| Chen et al 2017^13^ | CG | 68.6±3.1 | 15 | 8 |  |  |  |  | Study-defined criteria | China | Community |
|  | RT | 68.9±4.4 | 15 | 8 | 2 | 60 | 60-70% 1RM |  | Study-defined criteria | China | Community |
|  | AT | 69.3±3.0 | 15 | 8 | 2 | 60 | moderately intense |  | Study-defined criteria | China | Community |
|  | ART | 68.5±2.12 | 15 | 8 | 2 | 60 | RT: 60-70% 1RM AT: moderately intense |  | Study-defined criteria | China | Community |
| Chen et al 2018^14^ | RT | 66.7±5.3 | 17 | 8 | 2 | 60 | 60-70% 1RM |  | AWGS2014 | China | Community |
|  | CG | 68.3±2.8 | 16 | 8 |  |  |  |  | AWGS2014 | China | Community |
| Chen et al 2023^15^ | TCEs | 65.68±2.5 | 25 | 8 | 3 | 60 |  |  | AWGS 2019 | China | Community |
|  | CG | 65.21±2.6 | 24 | 8 | 3 | 60 |  |  | AWGS 2019 | China | Community |
| Chen et al 2024^16^ | Nu | 71.9±4.57 | 22 | 12 |  |  |  | consumed 4 slices of fortified cheese daily, providing 12.7 g of protein | AWGS 2019 | China | Community |
|  | Nu | 71.7±5.07 | 25 | 12 |  |  |  | consumed 4 slices of non-fortified cheese daily, providing 9.0 g of protein | AWGS 2019 | China | Community |
|  | CG | 72.3±5.3 | 21 | 12 |  |  |  | Maintained usual dietary habits | AWGS 2019 | China | Community |
| Chiang et al2021^17^ | RBT+Nu | 85±5.62 | 11 | 12 | 3 | 30 |  | 200 mL soy milk after breakfast and post-exercise | AWGS 2014 | China | Institution |
|  | RBT+Nu | 85.25±5.38 | 12 | 12 | 3 | 30 |  | 200 mL milk consumed after breakfast and post-exercise | AWGS 2014 | China | Institution |
|  | RBT | 84.67±7.5 | 12 | 12 |  |  |  |  | AWGS 2014 | China | Institution |
| Dieli-Conwright et al 2018^18^ | ART | 52.8±10.6 | 50 | 16 | 3 | 50–80 | 65%–85% HRmax |  | AWGS 2014 | USA | Institution |
|  | CG | 53.6±10.1 | 50 | 16 |  |  |  |  | AWGS 2014 | USA | Institution |
| El-Hak et al2021^19^ | AT | 58.15±3.06 | 20 | 12 | 3 | 30 |  |  | EWGSOP 2010 | Egypt | Institution |
|  | ART | 58.30±2.81 | 20 | 12 | 3 |  |  |  | EWGSOP 2010 | Egypt | Institution |
| Ferhi et al 2023^20^ | CG | 76.6±5.6 | 20 | 24 | 2 | 60 |  | Maintained daily physical activity without structured training | AWGS 2019 | Tunisia | Institution |
|  | RBT | 74.1±3.7 | 20 | 24 | 2 | 60 | RPE 5-7 (10) |  | AWGS 2019 | Tunisia | Institution |
| Hamaguchi2017^21^ | RT | 60.4±2.7 | 7 | 6 | 2 | 60 | 35% 1RM |  | EWGSOP 2010 | Japan | Community |
|  | CG | 60.6±2.3 | 8 | 6 | 2 | 60 |  | Maintained baseline activity levels | EWGSOP 2010 | Japan | Community |
| Hassan et al2016^22^ | RBT | 85.7±7.0 | 21 | 26 | 2 | 60 | 65-75% 1RM |  | EWGSOP 2010 | Australia | Institution |
|  | CG | 86.1±8.2 | 21 | 26 |  |  |  |  | EWGSOP 2010 | Australia | Institution |
| Huang et al 2017^23^ | RT | 68.89±4.91 | 18 | 12 | 3 | 55 | RPE≥13 (20) |  | Study-defined criteria | China | Community |
|  | CG | 68.89±4.91 | 17 | 12 |  |  |  |  | Study-defined criteria | China | Community |
| Huang et al 2023^24^ | TCEs | 69.7±5.05 | 27 | 12 | 3 | 40 | RPE＜4(10) |  | AWGS 2014 | China | Community |
|  | CG | 72.14±4.79 | 29 | 12 |  |  |  |  | AWGS 2014 | China | Community |
| Iranzo et al 2018^25^ | CG | 81.2±5.4 | 17 | 12 | 3 | 45 |  |  | Study-defined criteria | Spain | Institution |
|  | RT | 82.6±9.1 | 11 | 12 |  |  | 40–60% of maximal isometric muscle strength |  | Study-defined criteria | Spain | Institution |
| Jung et al 2019^26^ | CT | 75±3.9 | 13 | 12 | 3 | 40 | 60–80% HRR |  | AWGS2014 | Korea | Community |
|  | CG | 74.9±5.2 | 13 |  |  |  |  |  | AWGS2014 | Korea | Community |
| Jung et al 2024^27^ | CT | 78.14±3.72 | 14 | 12 | 3 | 40 | 60–80% HRR |  | AWGS2014 | Korea | Community |
|  | CG | 78.21±3.72 | 14 |  |  |  |  |  | AWGS2014 | Korea | Community |
| Kemmler et al 2018^27^ | EMS+Nu | 77.1±4.3 | 33 | 16 | 1.5 | 20 | RPE 6-7 (10) | whey protein supplementation to achieve 1.8 g/kg/day protein intake | FNIH | Germany | Community |
|  | CG | 76.9±5.1 | 34 | 16 |  |  |  | Maintained regular daily activities | FNIH | Germany | Community |
| Kemmler et al 2020^28,29^ | Nu | 77.8±3.6 | 22 | 52 |  |  |  | Daily whey protein intake of 1.2 g/kg body weight, with calcium (1000 mg/day) and vitamin D (800 IU/day) supplementation | EWGSOP 2010 | Germany | Community |
|  | RT+Nu | 79.2±4.7 | 21 | 52 | 2 | 47 |  | Whey protein supplementation targeting 1.5-1.6 g/kg/day protein intake, with additional calcium (1000 mg/day) and vitamin D (800 IU/day) | EWGSOP 2010 | Germany | Community |
| Kemmler et al2016^30^ | EMS | 77.3±4.9 | 23 | 26 | 1 | 20 | RPE 5–6 (10) |  | EWGSOP 2010 | Germany | Community |
|  | EMS+Nu | 76.4±2.9 | 23 | 26 | 1 | 20 | RPE 5–6 (10) | Daily protein supplement (150 kcal, 56% protein, ~21 g protein/day) | EWGSOP 2010 | Germany | Community |
|  | CG | 77.4±4.9 | 22 | 26 |  |  |  | Maintained usual physical activity | EWGSOP 2010 | Germany | Community |
| Kemmler et al2017^31^ | EMS+Nu | 77.1±4.3 | 33 | 16 | 1.5 | 20 | RPE 6-7 (10) | High-protein diet providing 1.7–1.8 g/kg/day protein | FNIH | Germany | Community |
|  | Nu | 78.1±5.1 | 33 | 16 |  |  |  | High-protein diet providing 1.7–1.8 g/kg/day protein intake | FNIH | Germany | Community |
|  | CG | 76.9±5.1 | 34 | 16 |  |  |  | Maintained daily activities | FNIH | Germany | Community |
| Kim et al 2012^32^ | RBT+Nu | 79.5±2.9 | 38 | 12 | 2 | 60 | RPE 12-14 (20) | Daily supplementation of 3 g leucine-rich essential amino acids (6 g/day total) | Study-defined criteria | Japan | Community |
|  | Nu | 79.2±2.8 | 39 | 12 |  |  |  | Supplementation with 3 g leucine-rich essential amino acids, twice daily (6 g/day total) | Study-defined criteria | Japan | Community |
|  | RBT | 79.0±2.9 | 39 | 12 | 2 | 60 | RPE 12-14 (20) |  | Study-defined criteria | Japan | Community |
|  | CG | 78.7±2.8 | 39 | 12 |  |  |  | Received health education only | Study-defined criteria | Japan | Community |
| Kim et al 2013^33^ | RBT | 79.6±4.2 | 32 | 12 | 2 | 60 | RPE 12-14 (20) |  | Study-defined criteria | Japan | Community |
|  | CG | 80.2±5.6 | 32 | 12 |  |  |  | Received health education only | Study-defined criteria | Japan | Community |
| Kim et al 2016^34^ | ART+Nu | 80.9±4.2 | 36 | 12 | 2 | 60 |  | Essential amino acid supplementation (3 g/dose, twice daily, 6 g/day total) | Study-defined criteria | Japan | Community |
|  | ART | 81.4±4.3 | 35 | 12 |  |  |  |  | Study-defined criteria | Japan | Community |
|  | Nu | 81.2±4.9 | 34 | 12 |  |  |  | Supplementation with essential amino acids (3 g/dose, twice daily, 6 g/day total) | Study-defined criteria | Japan | Community |
|  | CG | 81.1±5.1 | 34 | 12 |  |  |  | Received health education only | Study-defined criteria | Japan | Community |
| Kyun et al2020^35^ | ARBT | 76.94±9.43 | 19 | 2 | 5 | 50 | 50%–80% of body weight |  | AWGS 2014 | Korea | Institution |
|  | RBT | 81.15±4.9 | 19 | 2 | 5 | 50 |  |  | AWGS 2014 | Korea | Institution |
| Lee et al 2021^36^ | RT | 70.13±4.51 | 15 | 12 | 3 | 55 | RPE 13 (20) |  | EWGSOP2010 | China | Community |
|  | CG | 71.82±5.33 | 12 | 12 |  |  |  |  | EWGSOP2010 | China | Community |
| Li et al 2022^37^ | TCEs | 80.57±8.93 | 35 | 24 |  |  |  |  | AWGS 2014 | China | Community |
|  | ART | 77.89±10.38 | 35 | 24 |  |  |  |  | AWGS 2014 | China | Community |
| Li et al 2020^38^ | Nu | 70.04±3.98 | 51 | 12 |  |  |  | Nutrition supplementation groups consumed 10 g of whey protein powder with each main meal (total 30 g/day), along with 2 daily doses of fish oil capsules containing 300 mg EPA and 200 mg DHA, and 500 IU of vitamin D₃ (250 IU per dose) | AWGS 2014 | China | Community |
|  | ART | 73.73±5.69 | 37 | 12 | 3 | 90 | RT: 80% 1RM AT: 800 steps per 10 minutes |  | AWGS 2014 | China | Community |
|  | ART+Nu | 71.52±5.28 | 48 | 12 | 3 | 90 | RT: 80% 1RM AT: 800 steps per 10 minutes | Nutrition supplementation groups consumed 10 g of whey protein powder with each main meal (total 30 g/day), along with 2 daily doses of fish oil capsules containing 300 mg EPA and 200 mg DHA, and 500 IU of vitamin D₃ (250 IU per dose) | AWGS 2014 | China | Community |
|  | CG | 72.91±6.29 | 33 | 12 |  |  |  | Received general health counseling | AWGS 2014 | China | Community |
| Liang et al 2020^39^ | RBT | 87.3±6 | 30 | 12 | 2 | 55 | 70–80% 1RM |  | AWGS 2014 | China | Institution |
|  | RT | 86.8±4.7 | 30 | 12 | 2 | 55 | 70–80% 1RM |  | AWGS 2014 | China | Institution |
| Liao et al 2017^40^ | RT | 68.42±5.86 | 25 | 12 | 3 | 60 | RPE 13 (20) |  | EWGSOP2010 | China | Institution |
|  | CG | 66.39±4.49 | 21 | 12 |  |  |  |  | EWGSOP2010 | China | Institution |
| Liao et al 2018^41^ | RT | 66.67±4.54 | 33 | 12 | 3 | 60 | 60%-70% 1RM |  | Study-defined criteria | China | Institution |
|  | CG | 68.32±6.05 | 23 | 12 |  |  |  |  | Study-defined criteria | China | Institution |
| Liao et al 2021^42^ | RT | 69.81±7.24 | 36 | 12 | 2 | 45 | RPE 13-15 (20) |  | AWGS 2019 | China | Institution |
|  | RT+Nu | 68.64±7.42 | 36 | 12 | 2 | 45 | RPE 13-15 (20) | Daily protein supplement (24.2 g/day: 11 g plant oligopeptides, 4 g casein peptides, 5 g BCAA), taken in two divided doses | AWGS 2019 | China | Institution |
| Liao et al2024^43^ | CG | 73.21±4.98 | 46 | 16 |  |  |  | Received nutrition education only | AWGS2019 | China | Community |
|  | ART | 72.04±5.02 | 35 | 16 | 5 | 60 |  | oral peptide-based supplement (185 kcal/day, 24.2 g protein: 11 g plant peptides, 4 g casein peptides, 2.5 g CaHMB) | AWGS2019 | China | Community |
|  | Nu | 72.68±5.59 | 48 | 16 |  |  |  | oral peptide-based supplement (185 kcal/day, 24.2 g protein: 11 g plant peptides, 4 g casein peptides, 2.5 g CaHMB). | AWGS2019 | China | Community |
|  | ART+Nu | 70.52±3.3 | 30 | 16 | 5 | 60 |  | oral peptide-based supplement (185 kcal/day, 24.2 g protein: 11 g plant peptides, 4 g casein peptides, 2.5 g CaHMB). | AWGS2019 | China | Community |
| Lichtenberg et al2019^44^ | Nu | 79.2±4.7 | 22 | 28 |  |  |  | Dietary protein supplementation (1.2 g/kg/day) combined with vitamin D (800 IU/day) | EWGSOP2010 | Germany | Community |
|  | RT+Nu | 77.8±3.6 | 21 | 28 | 2 | 60 | 70%–85% 1RM | Protein supplementation (1.5 g/kg/day) and vitamin D (800 IU/day) | EWGSOP2010 | Germany | Community |
| Lin et al 2020^45^ | Nu | 73.8±8.11 | 28 | 12 |  |  |  | Daily intake from supplements was 25.6 g protein, 2.4 g leucine, and 240 IU vitamin D. | AWGS2014 | China | Institution |
|  | CG | 72.5±5.57 | 28 | 12 |  |  |  | Calorie-controlled diet | AWGS2014 | China | Institution |
| Lu et al2019^46^ | RBT+Nu | 69.76±4.31 | 78 | 12 | 2 |  | moderate and gradually increasing intensity | Combined resistance and balance training with daily nutritional supplementation, including Fortisip Multi Fibre (Nutricia), iron and folate (Sangobion), vitamins B6 and B12 (Neuroforte), and calcium plus vitamin D (Caltrate), administered for 24 weeks to increase caloric intake by ~20% and meet one-third of recommended micronutrient intake | AWGS 2014 | Singapore | Community |
|  | CG | 71±6.65 | 14 | 12 |  |  |  | Standard care | AWGS 2014 | Singapore | Community |
| Liu et al2024^47^ | CG | 75.6±6.35 | 45 | 12 |  |  |  | Daily activities maintained | AWGS2019 | China | Community |
|  | ART | 74.2±4.67 | 41 | 12 | 3 | 60 | RPE 12-14 (20) |  | AWGS2019 | China | Community |
| Mafi et al2018^48^ | RT | 69±2.44 | 14 | 8 | 3 | 55 | 60%~80% 1RM |  | Study-defined criteria | Iran | Institution |
|  | CG | 68±3.04 | 16 | 8 |  |  |  | Received placebo | Study-defined criteria | Iran | Institution |
| Magtouf et al2023^49^ | CG | 75.9±5.4 | 25 | 16 |  |  |  |  | Study-defined criteria | Tunisia | Institution |
|  | RBT | 76.1±3.5 | 25 | 16 | 3 | 60 |  |  | Study-defined criteria | Tunisia | Institution |
| Makizako et al 2020^50^ | CG | 75.8±7.3 | 36 | 12 |  |  |  |  | AWGS 2014 | Japan | Community |
|  | ARBT | 74.1±6.6 | 36 | 12 | 1 | 60 | RPE 12-14 (20) |  | AWGS 2014 | Japan | Community |
| Maltais et al 2016^51,52^ | RT | 64±4.5 | 10 | 16 | 3 | 60 | 80% 1RM |  | Study-defined criteria | Canada | Institution |
|  | RT+Nu | 64±4.8 | 8 | 16 | 3 | 60 | 80% 1RM | the amount of protein, milk powder was added to 1% chocolate cow milk (375 ml, containing 13.53 g protein, 7 g EAA, 37.5 g carbohydrate,3.8 g fat, 375 mg calcium; 270 calories). | Study-defined criteria | Canada | Institution |
| Martínez-Arnau et al2020^53^ | Nu | 78.4±8.4 | 23 | 13 |  |  |  | Daily oral supplementation with 6 g L-leucine | EWGSOP2010 | Spain | Institution |
|  | CG | 79.0±7.6 | 19 | 13 |  |  |  | Daily placebo supplementation with 6 g lactose | EWGSOP2010 | Spain | Institution |
| Maruya et al 2016^54^ | ARBT | 69.2±5.6 | 34 | 26 | 7 | 30 |  |  | AWGS 2014 | Japan | Community |
|  | CG | 68.5±6.2 | 18 | 26 |  |  |  |  | AWGS 2014 | Japan | Community |
| Meza-Valderrama et al 2024^55^ | RBT+Nu | 81.8±8.8 | 17 | 12 | 3 | 50 |  | Received daily supplementation with 3 g Ca-HMB | EWGSOP2 | Spain | Institution |
|  | RBT | 81.3±10.2 | 15 | 12 | 3 | 50 |  |  | EWGSOP2 | Spain | Institution |
| Molnár et al2016^56^ | RT | 66.35±1.79 | 17 | 12 | 2 | 30 |  |  | EWGSOP2010 | USA | Institution |
|  | RT+Nu | 66.59±1.63 | 17 | 12 | 2 | 30 |  | Received twice-daily supplementation with FortiFit (Nutricia), providing a total of 40 g whey protein, 20 g EAA (including 3 g leucine), 18 g carbohydrates, 6 g fat, and 1600 IU vitamin D | EWGSOP2010 | USA | Institution |
| Mori et al 2022^57^ | RT | 77.6±5.2 | 23 | 24 | 2 | 40 | 50–70% 1RM |  | AWGS 2014 | Japan | Community |
|  | NU | 77.8±4.5 | 24 | 24 |  |  |  | Leucine-enriched whey protein supplementation (11 g protein + 2300 mg leucine per dose) taken twice weekly | AWGS 2014 | Japan | Community |
|  | RT+Nu | 77.7±3.3 | 23 | 24 | 2 | 40 | 50–70% 1RM | Leucine-enriched whey protein supplementation (11 g protein + 2300 mg leucine per dose) taken twice weekly | AWGS 2014 | Japan | Community |
| Murphy et al2021^58^ | CG | 73±7 | 31 | 24 |  |  |  | Received placebo | Study-defined criteria | Ireland | Community |
|  | Nu | 70±5 | 38 | 24 |  |  |  | Leucine-enriched protein supplementation twice daily (each serving: 10 g protein + 3 g leucine) | Study-defined criteria | Ireland | Community |
| Nabuco et al 2019^59^ | RT+Nu | 68.0±4.2 | 13 | 12 | 3 |  |  | Daily supplementation with 35 g whey protein | Study-defined criteria | Brazil | Community |
|  | RT | 70.1±3.9 | 13 | 12 | 3 |  |  | Daily placebo supplementation | Study-defined criteria | Brazil | Community |
| Nasimi et al 2021^60^ | Nu | 71.0±6.35 | 33 | 12 |  |  |  | Daily intake of 300 g yogurt containing 3 g HMB, 1000 IU vitamin D, and 500 mg vitamin C | AWGS 2014 | Iran | Community |
|  | CG | 69.0±13.4 | 33 | 12 |  |  |  | Daily intake of 300 g plain yogurt | AWGS 2014 | Iran | Community |
| Nie et al 2023^61^ | ART+Nu | 66.76±5.15 | 50 | 12 | 5 | 30 |  | Daily supplementation of 400 kcal nutritional formula plus 0.6–1.0 g/kg whey protein powder | Study-defined criteria | China | Institution |
|  | CG | 67.28±5.13 | 50 | 12 |  |  |  | Usual care provided | Study-defined criteria | China | Institution |
| Osuka et al 2021^62^ | RT+Nu | 73.5±4.2 | 36 | 12 | 2 | 60 | RPE 12-14 (20) | Daily 1500 mg Ca-HMB supplementation | AWGS 2014 | Japan | Institution |
|  | RT | 71.8±4.1 | 38 | 12 | 2 | 60 | RPE 12-14 (20) |  | AWGS 2014 | Japan | Institution |
|  | Nu | 71.5±4.5 | 37 | 12 |  |  |  | Daily supplementation with 1500 mg Ca-HMB | AWGS 2014 | Japan | Institution |
|  | CG | 71.6±4.2 | 38 | 12 |  |  |  | Health education plus placebo supplementation | AWGS 2014 | Japan | Institution |
| Park et al 2017^63^ | CG | 74.7±5.1 | 25 | 26 |  |  |  |  | Study-defined criteria | Korea | Community |
|  | ART | 73.5±7.1 | 25 | 26 | 5 | 50 | RPE 13-17 (20) |  | Study-defined criteria | Korea | Community |
| Regard et al 2020^64^ | EMS | 78±2 | 12 | 12 | 2 | 20 | Frequency: 20 Hz, pulse width: 250 μs |  | Study-defined criteria | Switzerland | Institution |
|  | EMS+Nu | 78±1 | 15 | 12 | 2 | 20 | Frequency: 20 Hz, pulse width: 250 μs | Received an isocaloric carbohydrate beverage (containing 20 g carbohydrates) and placebo capsules (containing maltodextrin and medium-chain triglycerides) | Study-defined criteria | Switzerland | Institution |
| Rezaei et al2024^65^ | CG | 76.5±3.53 | 9 | 8 | 3 | 60 |  | Maintained daily activities | Study-defined criteria | Iran | Community |
|  | RT | 72.5±4.22 | 10 | 8 |  |  |  |  | Study-defined criteria | Iran | Community |
| Rondanelli et al 2024^66^ | RBT | 79.7±4.8 | 29 | 16 | 5 | 30 | RPE 12-14 (20) |  | EWGSOP2 | Italy | Institution |
|  | RBT+Nu | 79.7±4.8 | 30 | 16 | 5 | 30 |  | Received daily supplementation with 1.5 g Ca-HMB, 125 mg L-carnosine, 50 mg lactoferrin, 250 mg sodium butyrate, and 150 mg magnesium | EWGSOP2 | Italy | Institution |
| Rondanelli et al2016^67^ | RBT+Nu | 80.77±6.29 | 69 | 12 | 5 | 20 | RPE 12-14 (20) | Daily supplementation with 22 g whey protein, 10.9 g essential amino acids (including 4 g leucine), and 100 IU vitamin D | Study-defined criteria | Italy | Institution |
|  | RBT | 80.21±8.54 | 61 | 12 | 5 | 20 | RPE 12-14 (20) |  | Study-defined criteria | Italy | Institution |
| Rondanelli et al2018^68^ | RBT | 81.86±6.43 | 44 | 8 | 6 | 20 |  |  | Study-defined criteria | Italy | Institution |
|  | RBT+Nu | 81.42±8.02 | 33 | 8 | 6 | 20 |  | Daily oral essential amino acids (4 g/day, including 1.6 g leucine) | Study-defined criteria | Italy | Institution |
| Rondanelli et al2020^69^ | RBT | 82±5 | 63 | 8 | 5 | 30 | RPE 12-14 (20) |  | EWGSOP 2010 | Italy | Institution |
|  | RBT+Nu | 81±7 | 64 | 8 | 5 | 30 |  | Nutrition supplement (twice daily: 20 g whey protein + 2.5 g leucine + 800 IU vitamin D) | EWGSOP 2010 | Italy | Institution |
| Rondanelli et al2022^70^ | RBT+Nu | 78.84±5.80 | 22 | 8 | 5 | 30 | RPE 12-14 (20) | Daily supplementation of 2.5 g leucine, 500 mg omega-3 (EPA+DHA), and 1×10⁹ CFU Lactobacillus paracasei PS23 | EWGSOP 2 | Italy | Institution |
|  | RBT | 80.50±3.74 | 28 | 8 | 5 | 30 | RPE 12-14 (20) |  | EWGSOP 2 | Italy | Institution |
| Rufino et al2023^71^ | RT | 79.9±7.2 | 20 | 26 | 2 | 65 | ≥70% 1RM |  | EWGSOP2010 | Spain | Community |
|  | CG | 79.6±7.7 | 18 | 26 |  |  |  |  | EWGSOP2010 | Spain | Community |
| Sammarco et al 2017^72^ | CG | 58±10 | 9 | 16 |  |  |  | Low-calorie placebo supplement | Study-defined criteria | Italy | Institution |
|  | Nu | 53±8.9 | 9 | 16 |  |  |  | Low-calorie, high-protein diet providing 1.2–1.4 g/kg/day of protein | Study-defined criteria | Italy | Institution |
| Sen et al2020^73^ | ARBT | 73.0±4.8 | 46 | 12 | 3 | 60 | RPE 10-12 (20) |  | EWGSOP 2 | Turkey | Community |
|  | CG | 72.7±5.0 | 44 | 12 |  |  |  | Received health education only | EWGSOP 2 | Turkey | Community |
| Seo et al 2021^74^ | RT | 70.3±5.38 | 12 | 16 | 3 | 60 | OMNI-RES AM 7-8 |  | EWGSOP2010 | Korea | Institution |
|  | CG | 72.9±4.75 | 10 | 16 |  |  |  |  | EWGSOP2010 | Korea | Institution |
| Takeuchi et al 2019^75^ | RBT+Nu | 78.8±5.1 | 32 | 8 | 7 |  | 20–30% 1RM | Daily (≤3 h/day) + BCAA and vitamin D supplementation | AWGS 2014 | Japan | Institution |
|  | RBT | 80.9±7.3 | 31 | 8 | 7 |  |  |  | AWGS 2014 | Japan | Institution |
| Tokuda et al2021^76^ | RT | 79±2.2 | 15 | 24 | 2 | 40 |  |  | AWGS2019 | Japan | Community |
|  | RT+Nu | 78±1.48 | 15 | 24 | 2 | 40 |  | Received daily supplementation with 3 g EAA | AWGS2019 | Japan | Community |
| Tsekoura et al2018^77^ | ARBT | 74.56±6.04 | 18 | 12 | 5 | 32 | RPE 10-12 (20) |  | EWGSOP 2010 | Greece | Institution |
|  | ARBT | 71.17±6.47 | 18 | 12 | 6 | 39 | RPE 10-12 (20) |  | EWGSOP 2010 | Greece | Institution |
|  | CG | 72.89±8.31 | 18 | 12 |  |  |  |  | EWGSOP 2010 | Greece | Institution |
| Tuan et al 2024^78^ | ARBT | 79.73±6.82 | 30 | 12 | 2 | 50 | RPE 13 (20) |  | AWGS 2019 | China | Institution |
|  | CG | 79.73±6.82 | 30 | 12 |  |  |  |  | AWGS 2019 | China | Institution |
| Valdés-Badilla et al 2023^79^ | RT | 73.91±8.27 | 21 | 12 |  |  | OMNI-RES 5-8 and ≥75% 1RM |  | Study-defined criteria | Chile | Community |
|  | AT | 72.85±8.67 | 19 | 12 |  |  | Heart rate < 120 beats per minute. |  | Study-defined criteria | Chile | Community |
| Vasconcelos et al 2016^80^ | RT | 72±4.6 | 14 | 10 | 2 | 60 | 40-75% 1RM |  | Study-defined criteria | Brazil | Community |
|  | CG | 72±3.6 | 14 | 10 |  |  |  |  | Study-defined criteria | Brazil | Community |
| Vezzoli et al2019^81^ | CG | 71.7±3.4 | 15 | 12 | 3 |  |  | Maintained daily activities | Study-defined criteria | Italy | Community |
|  | RT | 73.0±5.5 | 20 | 12 | 3 |  | 60% 1RM |  | Study-defined criteria | Italy | Community |
| Vijayakumaran et al2023^82^ | RT+Nu | 66.6±4.0 | 8 | 12 | 3 |  | 80% 1-RM | Day supplementation of 15.1 g whey protein | AWGS 2019 | Malaysia | Community |
|  | RT | 65.5±1.9 | 8 | 12 |  |  |  |  | AWGS 2019 | Malaysia | Community |
| Vikberg et al2019^83^ | CG | 70.0±0.29 | 34 | 10 |  |  |  | Maintained daily activity | EWGSOP 2010 | Sweden | Community |
|  | RT+Nu | 70.9±0.28 | 36 | 10 | 3 | 45 | RPE 6–7 (10) | 250 mL whey drink providing 21 g protein | EWGSOP 2010 | Sweden | Community |
| Wang et al2022^84^ | CG | 69.88±3.29 | 51 | 12 | 10 |  |  | Health education only | EWGSOP2 | China | Community |
|  | ART+Nu | 70.16±4.32 | 50 | 12 | 10 | 45 |  | 1.2–1.5 g/kg/day protein (≥50% high-quality protein) and 600–800 IU/day vitamin D | EWGSOP2 | China | Community |
|  | Nu | 68.18±3.93 | 50 | 12 | 10 |  |  | 1.2–1.5 g/kg/day protein (≥50% high-quality protein) and 600–800 IU/day vitamin D | EWGSOP2 | China | Community |
|  | ART | 69.72±3.60 | 50 | 12 | 10 | 45 |  |  | EWGSOP2 | China | Community |
| Wei et al2016^85^ | WBV | 75±6 | 20 | 12 | 3 | 6 | 40 Hz |  | Study-defined criteria | China | Community |
|  | CG | 76±12 | 20 | 12 |  |  |  |  | Study-defined criteria | China | Community |
| Wei et al2017^86,87^ | WBV | 78±4 | 20 | 12 | 3 | 12 | 20 Hz |  | Study-defined criteria | China | Community |
|  | WBV | 75±6 | 20 | 12 | 3 | 6 | 40 Hz |  | Study-defined criteria | China | Community |
|  | WBV | 74±5 | 20 | 12 | 3 | 4 | 60 Hz |  | Study-defined criteria | China | Community |
|  | CG | 76±6 | 20 | 12 |  |  | Maintained daily activities |  | Study-defined criteria | China | Community |
| Wei et al2022^88^ | TCEs | 66.70±4.10 | 30 | 24 | 3 | 80 |  |  | AWGS 2016 | China | Community |
|  | RT | 66.87±3.84 | 30 | 24 | 3 | 80 | 40-85% 1RM |  | AWGS 2016 | China | Community |
|  | CG | 65.42±3.100 | 30 | 24 |  |  |  |  | AWGS 2016 | China | Community |
| Yamada et al2019^89^ | RT+Nu | 84.9±5.6 | 28 | 12 | 2 | 30 |  | Daily intake of 10 g whey protein and 800 IU vitamin D | AWGS 2014 | Japan | Community |
|  | RT | 84.7±5.1 | 28 | 12 | 2 | 30 |  |  | AWGS 2014 | Japan | Community |
|  | Nu | 83.2±5.7 | 28 | 12 |  |  |  | Daily intake of 10 g whey protein and 800 IU vitamin D | AWGS 2014 | Japan | Community |
|  | CG | 83.9±5.7 | 28 | 12 |  |  |  | Maintained usual lifestyle | AWGS 2014 | Japan | Community |
| Yang et al 2023^90^ | RT+Nu | 72.89±7.02 | 18 | 12 | 2 | 40 |  | Received twice-daily supplementation with 1.5 g Ca-HMB (3 g/day total) | AWGS2019 | China | Community |
|  | RT | 71.44±5.22 | 16 | 12 | 2 | 40 |  | isoenergetic placebo | AWGS2019 | China | Community |
| Yuenyongchaiwat et al 2022^91^ | ART | 69.23±6.71 | 30 | 12 | 7 |  |  |  | AWGS 2019 | Thailand | Community |
|  | CG | 71.93±5.19 | 30 | 12 |  |  |  |  | AWGS 2019 | Thailand | Community |
| Yun et al 2021^92^ | RT | 78.3±5.3 | 13 | 12 | 5 | 30 |  |  | Study-defined criteria | Korea | Institution |
|  | CG | 78.2±4.8 | 13 | 12 |  |  |  | Usual care | Study-defined criteria | Korea | Institution |
| Zdzieblik et al2015^93^ | RT+Nu | 72.3±3.7 | 27 | 12 | 3 | 60 | 65%–80% HRmax | Daily collagen peptide supplementation (15 g) | EWGSOP 2010 | Germany | Community |
|  | RT | 72.1±5.5 | 27 | 12 | 3 | 60 | 65%–80% HRmax | Supplemented with an equivalent amount of silica | EWGSOP 2010 | Germany | Community |
| Zhu et al 2017^94^ | TCEs | 65.6±11.4 | 32 | 12 | 1 | 40 |  |  | Study-defined criteria | China | Community |
|  | CG | 66.3±10.8 | 31 | 12 |  |  |  |  | Study-defined criteria | China | Community |
| Zhu et al 2018^95^ | CG | 72.2±6.6 | 37 | 12 |  |  |  | Maintained usual activities | AWGS2014 | China | Community |
|  | ART | 74.5±7.1 | 40 | 12 | 3 | 90 |  |  | AWGS2014 | China | Community |
|  | ART+Nu | 74.8±6.9 | 36 | 12 | 3 | 90 |  | NutriVigor twice daily (8.61 g protein, 1.21 g HMB, 130 IU vitamin D per sachet) | AWGS2014 | China | Community |
| Zhu et al2019^96^ | TCEs | 88.8±3.7 | 24 | 8 | 5 | 40 |  |  | AWGS 2014 | China | Institution |
|  | WBV | 89.5±4.4 | 28 | 8 | 5 | 40 | 14 Hz |  | AWGS 2014 | China | Institution |
|  | CG | 87.5±3.0 | 27 | 8 |  |  |  |  | AWGS 2014 | China | Institution |

**ARBT**: Aerobic, resistance, and balance training; **ARBT+Nu**: Aerobic, resistance, and balance training combined with protein-based nutritional supplementation; **ART**: Aerobic and resistance training; **ART+Nu**: Aerobic and resistance training combined with protein-based nutritional supplementation; **AT**: Aerobic training; **CG**: Control group; **CT**: Circuit training; **EMS**: Electrical muscle stimulation; **EMS+Nu**: Electrical muscle stimulation combined with protein-based nutritional supplementation; **Nu**: Protein-based nutritional supplementation; **RBT**: Resistance and balance training; **RBT+Nu**: Resistance and balance training combined with protein-based nutritional supplementation; **RT**: Resistance training; **RT+Nu**: Resistance training combined with protein-based nutritional supplementation; **TCEs**: Traditional Chinese exercises (e.g., Tai Chi, Yi Jin Jing); **WBV**: Whole-body vibration training

**Reference**

1. The LACE study group *et al.* Effect of perindopril or leucine on physical performance in older people with sarcopenia: The LACE randomized controlled trial. *J. Cachexia Sarcopenia Muscle* **13**, 858–871 (2022).

2. Aleman-Mateo, H., Macias, L., Esparza-Romero, J., Astiazaran-Garcia, H. & Blancas, A. L. Physiological effects beyond the significant gain in muscle mass in sarcopenic elderly men: evidence from a randomized clinical trial using a protein-rich food. *Clin. Interv. Aging* 225 (2012) doi:10.2147/CIA.S32356.

3. Amasene, M. *et al.* Effects of Leucine-Enriched Whey Protein Supplementation on Physical Function in Post-Hospitalized Older Adults Participating in 12-Weeks of Resistance Training Program: A Randomized Controlled Trial. *Nutrients* **11**, 2337 (2019).

4. Amasene, M. *et al.* Effects of resistance training intervention along with leucine-enriched whey protein supplementation on sarcopenia and frailty in post-hospitalized older adults: Preliminary findings of a randomized controlled trial. *J. Clin. Med.* **11**, 97 (2021).

5. Bagheri, R. *et al.* The effects of concurrent training order on body composition and serum concentrations of follistatin, myostatin and GDF11 in sarcopenic elderly men. *Exp. Gerontol.* **133**, 110869 (2020).

6. Balachandran, A., Krawczyk, S. N., Potiaumpai, M. & Signorile, J. F. High-speed circuit training vs hypertrophy training to improve physical function in sarcopenic obese adults: A randomized controlled trial. *Exp. Gerontol.* **60**, 64–71 (2014).

7. Bauer, J. M. *et al.* Effects of a vitamin D and leucine-enriched whey protein nutritional supplement on measures of sarcopenia in older adults, the PROVIDE study: A randomized, double-blind, placebo-controlled trial. *J. Am. Med. Dir. Assoc.* **16**, 740–747 (2015).

8. Bellomo, R. G. *et al.* Muscle strength and balance training in sarcopenic elderly: A pilot study with randomized controlled trial. *Eur. J. Inflamm.* **11**, 193–201 (2013).

9. Bernabei, R. *et al.* Multicomponent intervention to prevent mobility disability in frail older adults: Randomised controlled trial (SPRINTT project). *BMJ* **377**, e068788 (2022).

10. Björkman, M. P. *et al.* Effect of protein supplementation on physical performance in older people with sarcopenia-a randomized controlled trial. *J. Am. Med. Dir. Assoc.* **21**, 226-232.e1 (2020).

11. Bo, Y. *et al.* A high whey protein, vitamin D and E supplement preserves muscle mass, strength, and quality of life in sarcopenic older adults: A double-blind randomized controlled trial. *Clin. Nutr.* **38**, 159–164 (2019).

12. Chalé, A. *et al.* Efficacy of whey protein supplementation on resistance exercise–induced changes in lean mass, muscle strength, and physical function in mobility-limited older adults. *J. Gerontol. Ser. A* **68**, 682–690 (2013).

13. Chen, H., Chung, Y., Chen, Y., Ho, S. & Wu, H. Effects of different types of exercise on body composition, muscle strength, and IGF‐1 in the elderly with sarcopenic obesity. *J. Am. Geriatr. Soc.* **65**, 827–832 (2017).

14. Chen, H.-T., Wu, H.-J., Chen, Y.-J., Ho, S.-Y. & Chung, Y.-C. Effects of 8-week kettlebell training on body composition, muscle strength, pulmonary function, and chronic low-grade inflammation in elderly women with sarcopenia. *Exp. Gerontol.* **112**, 112–118 (2018).

15. Chen, B. *et al.* Effect of a moderate-intensity comprehensive exercise program on body composition, muscle strength, and physical performance in elderly females with sarcopenia. *Heliyon* **9**, e18951 (2023).

16. Chen, J. *et al.* Effects of cheese ingestion on muscle mass and strength in possible sarcopenia women: An open-label, parallel-group study. *Nutr. Metab.* **21**, 64 (2024).

17. Chiang, F.-Y., Chen, J.-R., Lee, W.-J. & Yang, S.-C. Effects of milk or soy milk combined with mild resistance exercise on the muscle mass and muscle strength in very old nursing home residents with sarcopenia. *Foods* **10**, 2581 (2021).

18. Dieli-Conwright, C. M. *et al.* Effects of aerobic and resistance exercise on metabolic syndrome, sarcopenic obesity, and circulating biomarkers in overweight or obese survivors of breast cancer: A randomized controlled trial. *J. Clin. Oncol.* **36**, 875–883 (2018).

19. Gad, M. G. E.-H. I., El-Hak, S. E.-B., El-SayedEl-Mekawy, H. & Moustafa, M. H. Efficacy of aerobic and core exercise training on improving muscle mass and physical performance in postmenopausal women with sarcopenic obesity. *J. Cardiovasc. Dis. Res.* **12**, 802–813 (2021).

20. Ferhi, H. *et al.* Effects of physical activity program on body composition, physical performance, and neuromuscular strategies during walking in older adults with sarcopenic obesity: Randomized controlled trial. *Healthcare* **11**, 2294 (2023).

21. Hamaguchi, K. *et al.* The effects of low-repetition and light-load power training on bone mineral density in postmenopausal women with sarcopenia: a pilot study. *BMC Geriatr.* **17**, 102 (2017).

22. Hassan, B. H. *et al.* Impact of resistance training on sarcopenia in nursing care facilities: A pilot study. *Geriatr. Nur. (Lond.)* **37**, 116–121 (2016).

23. Huang, S.-W. *et al.* Body composition influenced by progressive elastic band resistance exercise of sarcopenic obesity elderly women: A pilot randomized controlled trial. *Eur. J. Phys. Rehabil. Med.* **53**, (2017).

24. Huang, D. *et al.* Effects of 12 weeks of tai chi on neuromuscular responses and postural control in elderly patients with sarcopenia: A randomized controlled trial. *Front. Neurol.* **14**, 1167957 (2023).

25. Cebrià I Iranzo, M. À., Balasch-Bernat, M., Tortosa-Chuliá, M. Á. & Balasch-Parisi, S. Effects of resistance training of peripheral muscles versus respiratory muscles in older adults with sarcopenia who are institutionalized: A randomized controlled trial. *J. Aging Phys. Act.* **26**, 637–646 (2018).

26. Jung, W.-S., Kim, Y.-Y. & Park, H.-Y. Circuit training improvements in korean women with sarcopenia. *Percept. Mot. Skills* **126**, 828–842 (2019).

27. Jung, W.-S., Ahn, H., Kim, S.-W. & Park, H.-Y. Effects of 12-week circuit exercise intervention on blood pressure, vascular function, and inflammatory cytokines in obese older women with sarcopenia. *Rev. Cardiovasc. Med.* **25**, 185 (2024).

28. Kemmler, W., Kohl, M., Jakob, F., Engelke, K. & Von Stengel, S. Effects of High Intensity Dynamic Resistance Exercise and Whey Protein Supplements on Osteosarcopenia in Older Men with Low Bone and Muscle Mass. Final Results of the Randomized Controlled FrOST Study. *Nutrients* **12**, 2341 (2020).

29. Kemmler, W. *et al.* Effects of high-intensity resistance training on fitness and fatness in older men with osteosarcopenia. *Front. Physiol.* **11**, 1014 (2020).

30. Kemmler, W. *et al.* Whole-body electromyostimulation to fight sarcopenic obesity in community-dwelling older women at risk. Resultsof the randomized controlled FORMOsA-sarcopenic obesity study. *Osteoporos. Int.* **27**, 3261–3270 (2016).

31. Kemmler, W. *et al.* Whole-body electromyostimulation and protein supplementation favorably affect sarcopenic obesity in community-dwelling older men at risk: The randomized controlled FranSO study. *Clin. Interv. Aging* **Volume 12**, 1503–1513 (2017).

32. Kim, H. K. *et al.* Effects of exercise and amino acid supplementation on body composition and physical function in community‐dwelling elderly japanese sarcopenic women: A randomized controlled trial. *J. Am. Geriatr. Soc.* **60**, 16–23 (2012).

33. Kim, H. *et al.* Effects of exercise and tea catechins on muscle mass, strength and walking ability in community‐dwelling elderly japanese sarcopenic women: A randomized controlled trial. *Geriatr. Gerontol. Int.* **13**, 458–465 (2013).

34. Kim, H. *et al.* Exercise and nutritional supplementation on community-dwelling elderly japanese women with sarcopenic obesity: A randomized controlled trial. *J. Am. Med. Dir. Assoc.* **17**, 1011–1019 (2016).

35. Oh, M.-K. *et al.* Efficacy of combined antigravity treadmill and conventional rehabilitation after hip fracture in patients with sarcopenia. *J. Gerontol. Ser. A* **75**, e173–e181 (2020).

36. Lee, Y.-H. *et al.* Effects of progressive elastic band resistance exercise for aged osteosarcopenic adiposity women. *Exp. Gerontol.* **147**, 111272 (2021).

37. Li, Z. *et al.* Ditangquan exercises based on safe-landing strategies prevent falls and injury among older individuals with sarcopenia. *Front. Med.* **9**, 936314 (2022).

38. Li, Z. *et al.* Effects of nutrition supplementation and physical exercise on muscle mass, muscle strength and fat mass among sarcopenic elderly: A randomized controlled trial. *Appl. Physiol. Nutr. Metab.* **46**, 494–500 (2021).

39. Liang, Y., Wang, R., Jiang, J., Tan, L. & Yang, M. A randomized controlled trial of resistance and balance exercise for sarcopenic patients aged 80–99 years. *Sci. Rep.* **10**, 18756 (2020).

40. Liao, C.-D. *et al.* Effects of elastic resistance exercise on body composition and physical capacity in older women with sarcopenic obesity: A CONSORT-compliant prospective randomized controlled trial. *Medicine (Baltimore)* **96**, e7115 (2017).

41. Liao, C.-D. *et al.* Effects of elastic band exercise on lean mass and physical capacity in older women with sarcopenic obesity: A randomized controlled trial. *Sci. Rep.* **8**, 2317 (2018).

42. Liao, C.-D. *et al.* Effects of protein-rich nutritional composition supplementation on sarcopenia indices and physical activity during resistance exercise training in older women with knee osteoarthritis. *Nutrients* **13**, 2487 (2021).

43. Liao, X. *et al.* Effects of oral oligopeptide preparation and exercise intervention in older people with sarcopenia: A randomized controlled trial. *BMC Geriatr.* **24**, 260 (2024).

44. Lichtenberg, T., Von Stengel, S., Sieber, C. & Kemmler, W. The favorable effects of a high-intensity resistance training on sarcopenia in older community-dwelling men with osteosarcopenia: The randomized controlled FrOST study. *Clin. Interv. Aging* **Volume 14**, 2173–2186 (2019).

45. Lin, C.-C., Shih, M.-H., Chen, C.-D. & Yeh, S.-L. Effects of adequate dietary protein with whey protein, leucine, and vitamin D supplementation on sarcopenia in older adults: An open-label, parallel-group study. *Clin. Nutr.* **40**, 1323–1329 (2021).

46. Lu, Y. *et al.* Assessment of sarcopenia among community-dwelling at-risk frail adults aged 65 years and older who received multidomain lifestyle interventions: A secondary analysis of a randomized clinical trial. *JAMA Netw. Open* **2**, e1913346 (2019).

47. Liu, M. *et al.* Graded progressive home-based resistance combined with aerobic exercise in community-dwelling older adults with sarcopenia: A randomized controlled trial. *Clin. Interv. Aging* **Volume 19**, 1581–1595 (2024).

48. Mafi, F., Biglari, S., Ghardashi Afousi, A. & Gaeini, A. A. Improvement in skeletal muscle strength and plasma levels of follistatin and myostatin induced by an 8-week resistance training and epicatechin supplementation in sarcopenic older adults. *J. Aging Phys. Act.* **27**, 384–391 (2019).

49. Magtouf, E. *et al.* Influence of concurrent exercise training on ankle muscle activation during static and proactive postural control on older adults with sarcopenic obesity: A multicenter, randomized, and controlled trial. *Eur. J. Investig. Health Psychol. Educ.* **13**, 2779–2794 (2023).

50. Makizako, H. *et al.* Effects of a multicomponent exercise program in physical function and muscle mass in sarcopenic/pre-sarcopenic adults. *J. Clin. Med.* **9**, 1386 (2020).

51. Maltais, M. L., Ladouceur, J. P. & Dionne, I. J. The effect of resistance training and different sources of postexercise protein supplementation on muscle mass and physical capacity in sarcopenic elderly men. *J. Strength Cond. Res.* **30**, 1680–1687 (2016).

52. Maltais, M. L. *et al.* Effect of resistance training and various sources of protein supplementation on body fat mass and metabolic profile in sarcopenic overweight older adult men: A pilot study. *Int. J. Sport Nutr. Exerc. Metab.* **26**, 71–77 (2016).

53. Martínez-Arnau, F. M. *et al.* Effects of leucine administration in sarcopenia: A randomized and placebo-controlled clinical trial. *Nutrients* **12**, 932 (2020).

54. Maruya, K. *et al.* Effect of a simple and adherent home exercise program on the physical function of community dwelling adults sixty years of age and older with pre-sarcopenia or sarcopenia. *J. Phys. Ther. Sci.* **28**, 3183–3188 (2016).

55. Meza-Valderrama, D. *et al.* Supplementation with β-hydroxy-β-methylbutyrate after resistance training in post-acute care patients with sarcopenia: A randomized, double-blind placebo-controlled trial. *Arch. Gerontol. Geriatr.* **119**, 105323 (2024).

56. Molnár, A. *et al.* Special nutrition intervention is required for muscle protective efficacy of physical exercise in elderly people at highest risk of sarcopenia. *Physiol. Int.* **103**, 368–376 (2016).

57. Mori, H. & Tokuda, Y. De-training effects following leucine-enriched whey protein supplementation and resistance training in older adults with sarcopenia: A randomized controlled trial with 24 weeks of follow-up. *J. Nutr. Health Aging* **26**, 994–1002 (2022).

58. Murphy, C. H. *et al.* Does supplementation with leucine-enriched protein alone and in combination with fish-oil-derived n–3 PUFA affect muscle mass, strength, physical performance, and muscle protein synthesis in well-nourished older adults? A randomized, double-blind, placebo-controlled trial. *Am. J. Clin. Nutr.* **113**, 1411–1427 (2021).

59. Nabuco, H. C. G. *et al.* Effect of whey protein supplementation combined with resistance training on body composition, muscular strength, functional capacity, and plasma-metabolism biomarkers in older women with sarcopenic obesity: A randomized, double-blind, placebo-controlled trial. *Clin. Nutr. ESPEN* **32**, 88–95 (2019).

60. Nasimi, N. *et al.* A novel fortified dairy product and sarcopenia measures in sarcopenic older adults: A double-blind randomized controlled trial. *J. Am. Med. Dir. Assoc.* **22**, 809–815 (2021).

61. Nie, N. *et al.* Clinical efficacy of nutritional intervention combined with muscle exercise on sarcopenia patients with femoral fracture: A pilot randomized controlled trial. *BioMed Res. Int.* **2023**, 3222686 (2023).

62. Osuka, Y. *et al.* Effects of exercise and/or β-hydroxy-β-methylbutyrate supplementation on muscle mass, muscle strength, and physical performance in older women with low muscle mass: A randomized, double-blind, placebo-controlled trial. *Am. J. Clin. Nutr.* **114**, 1371–1385 (2021).

63. Park, J., Kwon, Y. & Park, H. Effects of 24-week aerobic and resistance training on carotid artery intima-media thickness and flow velocity in elderly women with sarcopenic obesity. *J. Atheroscler. Thromb.* **24**, 1117–1124 (2017).

64. Boutry-Regard, C., Vinyes-Parés, G., Breuillé, D. & Moritani, T. Supplementation with whey protein, omega-3 fatty acids and polyphenols combined with electrical muscle stimulation increases muscle strength in elderly adults with limited mobility: A randomized controlled trial. *Nutrients* **12**, 1866 (2020).

65. Rezaei, S., Eslami, R. & Tartibian, B. The effects of TRX suspension training on sarcopenic biomarkers and functional abilities in elderlies with sarcopenia: A controlled clinical trial. *BMC Sports Sci. Med. Rehabil.* **16**, 58 (2024).

66. Rondanelli, M. *et al.* A patented dietary supplement (hydroxy-methyl-butyrate, carnosine, magnesium, butyrate, lactoferrin) is a promising therapeutic target for age-related sarcopenia through the regulation of gut permeability: A randomized controlled trial. *Nutrients* **16**, 1369 (2024).

67. Rondanelli, M. *et al.* Whey protein, amino acids, and vitamin D supplementation with physical activity increases fat-free mass and strength, functionality, and quality of life and decreases inflammation in sarcopenic elderly. *Am. J. Clin. Nutr.* **103**, 830–840 (2016).

68. Rondanelli, M. *et al.* Is a combination of melatonin and amino acids useful to sarcopenic elderly patients? A randomized trial. *Geriatrics* **4**, 4 (2018).

69. Rondanelli, M. *et al.* Improving rehabilitation in sarcopenia: A randomized‐controlled trial utilizing a muscle‐targeted food for special medical purposes. *J. Cachexia Sarcopenia Muscle* **11**, 1535–1547 (2020).

70. Rondanelli, M. *et al.* Effectiveness of a novel food composed of leucine, omega-3 fatty acids and probiotic lactobacillus paracasei PS23 for the treatment of sarcopenia in elderly subjects: A 2-month randomized double-blind placebo-controlled trial. *Nutrients* **14**, 4566 (2022).

71. Flor-Rufino, C. *et al.* Fat infiltration and muscle hydration improve after high-intensity resistance training in women with sarcopenia. A randomized clinical trial. *Maturitas* **168**, 29–36 (2023).

72. Sammarco, R. *et al.* Evaluation of hypocaloric diet with protein supplementation in middle-aged sarcopenic obese women: A pilot study. *Obes. Facts* **10**, 160–167 (2017).

73. Sen, E. I. *et al.* Effect of home-based exercise program on physical function and balance in older adults with sarcopenia: A multicenter randomized controlled study. *J. Aging Phys. Act.* **29**, 1010–1017 (2021).

74. Seo, M.-W. *et al.* Effects of 16 weeks of resistance training on muscle quality and muscle growth factors in older adult women with sarcopenia: A randomized controlled trial. *Int. J. Environ. Res. Public. Health* **18**, 6762 (2021).

75. Takeuchi, I. *et al.* Effects of branched‐chain amino acids and vitamin D supplementation on physical function, muscle mass and strength, and nutritional status in sarcopenic older adults undergoing hospital‐based rehabilitation: A multicenter randomized controlled trial. *Geriatr. Gerontol. Int.* **19**, 12–17 (2019).

76. Tokuda, Y. & Mori, H. Essential amino acid and tea catechin supplementation after resistance exercise improves skeletal muscle mass in older adults with sarcopenia: An open-label, pilot, randomized controlled trial. *J. Am. Nutr. Assoc.* **42**, 255–262 (2023).

77. Tsekoura, M. *et al.* The effects of group and home-based exercise programs in elderly with sarcopenia: A randomized controlled trial. *J. Clin. Med.* **7**, 480 (2018).

78. Tuan, S.-H. *et al.* Assessing the clinical effectiveness of an exergame-based exercise training program using ring fit adventure to prevent and postpone frailty and sarcopenia among older adults in rural long-term care facilities: Randomized controlled trial. *J. Med. Internet Res.* **26**, e59468 (2024).

79. Valdés-Badilla, P. *et al.* Effectiveness of elastic band training and group-based dance on physical-functional performance in older women with sarcopenia: a pilot study. *BMC Public Health* **23**, 2113 (2023).

80. Vasconcelos, K. S. S. *et al.* Effects of a progressive resistance exercise program with high-speed component on the physical function of older women with sarcopenic obesity: A randomized controlled trial. *Braz. J. Phys. Ther.* **20**, 432–440 (2016).

81. Vezzoli, A. *et al.* Moderate intensity resistive training reduces oxidative stress and improves muscle mass and function in older individuals. *Antioxidants* **8**, 431 (2019).

82. Vijayakumaran, R. K., Daly, R. M. & Tan, V. P. S. “we want more”: Perspectives of sarcopenic older women on the feasibility of high-intensity progressive resistance exercises and a whey-protein nutrition intervention. *Front. Nutr.* **10**, 1176523 (2023).

83. Vikberg, S. *et al.* Effects of resistance training on functional strength and muscle mass in 70-year-old individuals with pre-sarcopenia: A randomized controlled trial. *J. Am. Med. Dir. Assoc.* **20**, 28–34 (2019).

84. Wang, Z. *et al.* Effects of internet-based nutrition and exercise interventions on the prevention and treatment of sarcopenia in the elderly. *Nutrients* **14**, 2458 (2022).

85. Wei, N. *et al.* Whole-body vibration training improves muscle and physical performance in community dwelling with sarcopenia: A randomized controlled trial. *Int. J. Phys. Ther. Rehabil.* **2**, (2016).

86. Wei, N., Pang, M. Y., Ng, S. S. & Ng, G. Y. Optimal frequency/time combination of whole‐body vibration training for improving muscle size and strength of people with age‐related muscle loss (sarcopenia): A randomized controlled trial. *Geriatr. Gerontol. Int.* **17**, 1412–1420 (2017).

87. Wei, N., Pang, M. Y., Ng, S. S. & Ng, G. Y. Optimal frequency/time combination of whole body vibration training for developing physical performance of people with sarcopenia: A randomized controlled trial. *Clin. Rehabil.* **31**, 1313–1321 (2017).

88. Wei, M. *et al.* Hybrid exercise program for sarcopenia in older adults: The effectiveness of explainable artificial intelligence-based clinical assistance in assessing skeletal muscle area. *Int. J. Environ. Res. Public. Health* **19**, 9952 (2022).

89. Yamada, M. *et al.* Synergistic effect of bodyweight resistance exercise and protein supplementation on skeletal muscle in sarcopenic or dynapenic older adults. *Geriatr. Gerontol. Int.* **19**, 429–437 (2019).

90. Yang, C. *et al.* Effects of beta-hydroxy-beta-methylbutyrate supplementation on older adults with sarcopenia: A randomized, double-blind, placebo-controlled study. *J. Nutr. Health Aging* **27**, 329–339 (2023).

91. Yuenyongchaiwat, K. & Akekawatchai, C. Beneficial effects of walking-based home program for improving cardio-respiratory performance and physical activity in sarcopenic older people: A randomized controlled trial. *Eur. J. Phys. Rehabil. Med.* **58**, (2023).

92. Yun, J. H., Kim, D. H. & Chang, M. C. A simple bedside exercise method to enhance lower limb muscle strength in moderate alzheimer’s disease patients with sarcopenia. *Healthcare* **9**, 680 (2021).

93. Zdzieblik, D., Oesser, S., Baumstark, M. W., Gollhofer, A. & König, D. Collagen peptide supplementation in combination with resistance training improves body composition and increases muscle strength in elderly sarcopenic men: A randomised controlled trial. *Br. J. Nutr.* **114**, 1237–1245 (2015).

94. Zhu, G., Shen, Z., Shen, Q., Jin, Y. & Lou, Z. Effect of yi jin jing (sinew-transforming qigong exercises) on skeletal muscle strength in the elderly. *J. Acupunct. Tuina Sci.* **15**, 434–439 (2017).

95. Zhu, L.-Y. *et al.* Effects of exercise and nutrition supplementation in community-dwelling older Chinese people with sarcopenia: A randomized controlled trial. *Age Ageing* **48**, 220–228 (2019).

96. Zhu, Y. *et al.* Tai chi and whole-body vibrating therapy in sarcopenic men in advanced old age: A clinical randomized controlled trial. *Eur. J. Ageing* **16**, 273–282 (2019).

# Appendix 3: Risk of bias of randomized clinical trials

**Table S3.1:** Study level risk of bias assessment using Cochrane risk of bias tool 2.0 for assessing risk of bias of randomized clinical trials.

| Unique ID | Randomization | Deviations from | Missing outcome | Measurement of | Selection of | Overall Bias |
| --- | --- | --- | --- | --- | --- | --- |
|  | process | intended interventions | data | the outcome | the reported result |  |
| Achison et al2022 | Low | Low | Some concerns | Low | Low | Some concerns |
| Alemán-Mateo et al2012 | Some concerns | Low | Low | Low | Some concerns | Some concerns |
| Amasene et al2019 | High | Low | Low | Low | Low | High |
| Amasene et al2021 | Some concerns | Some concerns | High | Low | Some concerns | High |
| Balachandran et al2014 | Some concerns | Some concerns | Low | Low | Some concerns | Some concerns |
| Bauer et al2015 | Low | Low | Low | Low | Low | Low |
| Bellomo et al2013 | Some concerns | Some concerns | Low | Low | Some concerns | Some concerns |
| Bernabei et al2022 | Low | Low | Low | Low | Low | Low |
| Björkman et al2019 | Low | Low | Low | Low | Low | Low |
| Bo et al2019 | Low | Low | Low | Low | Low | Low |
| Chale et al2013 | Some concerns | Some concerns | Low | Low | Low | Some concerns |
| Chen et al 2018 | Some concerns | Low | Low | Low | Some concerns | Some concerns |
| Chen et al2017 | Some concerns | Some concerns | High | Some concerns | Some concerns | High |
| Chen et al2023 | Low | Low | Low | Low | Some concerns | Some concerns |
| Chen et al2024 | Some concerns | Low | Low | Low | Low | Some concerns |
| Chiang et al2021 | Some concerns | Some concerns | Low | Low | Low | Some concerns |
| Cramer et al2016 | Low | Low | Some concerns | Low | Low | Some concerns |
| Dieli-Conwright et al2018 | Low | Low | Low | Low | Low | Low |
| EI-Hak et al2021 | Some concerns | Some concerns | Low | Low | Some concerns | Some concerns |
| Ferhi et al2023 | Low | Some concerns | High | Low | Low | High |
| Hamaguchi et al2017 | High | Some concerns | Low | Low | Low | Some concerns |
| Hassan et al2016 | Some concerns | Low | Low | Low | Some concerns | Some concerns |
| Huang et al2017 | Low | Low | Low | Low | Low | Low |
| Huang et al2023 | Low | Low | Low | Low | Low | Low |
| Iranzo et al2018 | Low | Low | Low | Low | Low | Low |
| Jung et al2019 | Some concerns | Some concerns | Low | Low | Some concerns | Some concerns |
| Jung et al2024 | Some concerns | Some concerns | Low | Low | Some concerns | Some concerns |
| Kemmler et al2016 | Low | Low | Low | Low | Low | Low |
| Kemmler et al2017 | Low | Low | Low | Low | Low | Low |
| Kemmler et al2018 | Low | Low | Low | Low | Low | Low |
| Kemmler et al2020a | Low | Low | Low | Low | Low | Low |
| Kemmler et al2020b | Low | Low | Low | Low | Low | Low |
| Kim et al2012 | Some concerns | Low | Low | Low | Some concerns | Some concerns |
| Kim et al2013 | Low | Low | Low | Low | Low | Low |
| Kim et al2016 | Some concerns | Some concerns | Low | Low | Some concerns | Some concerns |
| Kyun et al2020 | Low | Some concerns | Some concerns | Low | Low | Some concerns |
| Lee et al2021 | Low | Low | Low | Low | Low | Low |
| Li et al2020 | High | High | High | Low | Some concerns | High |
| Li et al2022 | Low | Low | Low | Low | Low | Low |
| Liang et al2020 | Some concerns | Low | Low | Low | Low | Some concerns |
| Liao et al 2017 | Some concerns | Low | Low | Low | Some concerns | Some concerns |
| Liao et al2018 | Low | Low | Low | Low | Low | Low |
| Liao et al2021 | Low | Low | Low | Low | Low | Low |
| Liao et al2024 | Some concerns | High | High | Low | Some concerns | High |
| Lichtenberg et al2019 | Low | Low | Low | Low | Low | Low |
| Lin et al2020 | Some concerns | High | High | Low | Some concerns | High |
| Liu et al2024 | Low | Low | Low | Low | Low | Low |
| Lu et al2019 | Low | Low | Low | Low | Low | Low |
| Mafi et al2018 | Some concerns | Low | Low | Low | Some concerns | Some concerns |
| Magtouf et al2023 | Low | High | High | Low | Some concerns | High |
| Makizako et al2020 | Some concerns | Low | Low | Low | Low | Some concerns |
| Maltais et al2016a | Some concerns | Low | Low | Low | Some concerns | Some concerns |
| Maltais et al2016b | Some concerns | Low | Low | Low | Some concerns | Some concerns |
| Martínez-Arnau et al2020 | Low | Low | Low | Low | Low | Low |
| Maruya et al2016 | Some concerns | Some concerns | High | High | Some concerns | High |
| Meza-Valderrama et al2024 | Low | Low | Low | Low | Low | Low |
| Molnar et al2016 | High | Some concerns | Low | Low | Some concerns | High |
| Mori et al2022 | Low | Some concerns | Low | Low | Some concerns | Some concerns |
| Murphy et al2021 | Low | Low | Some concerns | Low | Low | Some concerns |
| Nabuco et al2019 | Some concerns | Low | Low | Low | Low | Some concerns |
| Nasimi et al2021 | Low | Low | Low | Low | Low | Low |
| Nie et al2023 | Some concerns | Some concerns | Low | Low | Some concerns | Some concerns |
| Osuka et al2021 | Low | Low | Low | Low | Low | Low |
| Park et al2017 | Some concerns | Low | Low | Low | Some concerns | Some concerns |
| Regard et al2020 | Low | High | High | Low | Low | High |
| Rezaei et al2024 | Some concerns | Some concerns | Low | Low | Low | Some concerns |
| Rondanelli et al2016 | Low | Low | Low | Low | Low | Low |
| Rondanelli et al2018 | Some concerns | Low | Some concerns | Low | Low | Some concerns |
| Rondanelli et al2020 | Low | Low | Low | Low | Low | Low |
| Rondanelli et al2022 | Low | Low | Low | Low | Low | Low |
| Rondanelli et al2024 | Low | Low | Low | Low | Low | Low |
| Rufino et al2023 | Low | Low | Low | Low | Low | Low |
| Sammarco et al2017 | High | Some concerns | Low | Low | Some concerns | High |
| Sen et al2020 | Low | Some concerns | Low | Low | Low | Some concerns |
| Seo et al2021 | Some concerns | Low | Low | Low | Some concerns | Some concerns |
| Takeuchi et al2019 | Some concerns | Low | Low | Low | Low | Some concerns |
| Tokuda et al2021 | Low | Some concerns | Low | Low | Low | Some concerns |
| Tsekoura et al2018 | Low | Low | Low | Low | Low | Low |
| Tuan et al2024 | Some concerns | Low | Low | Low | Low | Some concerns |
| Valdés-Badilla | Low | Low | Low | Low | Low | Low |
| Vasconcelos et al2016 | Low | Low | Low | Low | Low | Low |
| Vezzoli et al2019 | Some concerns | Some concerns | High | Low | Some concerns | High |
| Vijayakumaran et al2023 | Some concerns | Some concerns | Low | Low | Some concerns | Some concerns |
| Vikberg et al2019 | Low | Low | Low | Low | Low | Low |
| Wang et al2022 | High | Some concerns | Low | Low | Low | High |
| Wei et al2016 | Some concerns | Low | Low | Low | Some concerns | Some concerns |
| Wei et al2017 | Some concerns | Low | Low | Low | Low | Some concerns |
| Wei et al2022 | Some concerns | Some concerns | Low | Low | Some concerns | Some concerns |
| Yamada et al2019 | Some concerns | Low | Low | Low | Some concerns | Some concerns |
| Yang et al2023 | Low | Low | Low | Low | Low | Low |
| Yuenyongchaiwat et al2022 | Some concerns | Low | Low | Low | Some concerns | Some concerns |
| Yun et al2021 | Some concerns | Low | Low | Low | Some concerns | Some concerns |
| Zdzieblik et al2015 | Some concerns | Some concerns | Low | Low | Some concerns | Some concerns |
| Zhu et al 2017 | Some concerns | Some concerns | Low | Low | Some concerns | Some concerns |
| Zhu et al 2019 | Some concerns | Some concerns | High | Low | Some concerns | High |
| Zhu et al2018 | Low | Low | Low | Low | Low | Low |

# Appendix 4: Evaluation of inconsistency and heterogeneity

**Table S4.1:** Global consistency and heterogeneity

| **Clinical outcome** | **Chi square** | **P value** | **I^2^** | **τ²** |
| --- | --- | --- | --- | --- |
| Grip strength | 15.28 | 0.8845 | 64.8% | 1.8834 |
| Knee extension strength | 14.326 | 0.3512 | 34.6% | 0.2179 |
| Gait speed | 13.09 | 0.9754 | 72% | 0.0669 |
| Five-Times Sit-to-Stand test | 7.09 | 0.4190 | 59.2% | 0.8767 |
| Timed up and go test | 2.05 | 0.5630 | 62.3% | 0.4248 |
| SPPB | NA | NA | 24.4% | 0.1702 |
| Balance test | 3.31 | 0.7696 | 73.1% | 0.2304 |
| ASMI | 3.21 | 0.9877 | 50.9% | 0.0331 |
| SMI | 0 | 0.9811 | 0% | 0 |

**Table S4.2:** Side-splitting of Grip strength. Inconsistency test between direct and indirect treatment comparisons in mixed treatment comparison.

| comparison | k | prop | nma | direct | indir. | Diff | z | p-value |
| --- | --- | --- | --- | --- | --- | --- | --- | --- |
| CG:ARBT | 6 | 0.85 | -1.5403 | -1.3248 | -2.7255 | 1.4006 | 0.65 | 0.5159 |
| RBT:ARBT | 1 | 0.3 | 0.575 | -0.41 | 0.9906 | -1.4006 | -0.65 | 0.5159 |
| ART:ART+Nu | 5 | 0.8 | -0.0295 | -0.3718 | 1.3152 | -1.687 | -0.98 | 0.3295 |
| ART:AT | 2 | 0.59 | 2.7607 | 2.295 | 3.4404 | -1.1454 | -0.49 | 0.6251 |
| ART:CG | 9 | 0.77 | 2.3441 | 2.8904 | 0.5083 | 2.3821 | 1.76 | 0.0778 |
| ART:Nu | 3 | 0.36 | 1.0458 | -0.3454 | 1.8217 | -2.1671 | -1.62 | 0.1057 |
| ART:RT | 1 | 0.06 | 0.4389 | -5.6 | 0.8075 | -6.4075 | -2.1 | 0.0361 |
| ART+Nu:CG | 5 | 0.72 | 2.3736 | 2.6059 | 1.78 | 0.8259 | 0.55 | 0.5817 |
| ART+Nu:Nu | 3 | 0.42 | 1.0753 | 0.0971 | 1.7934 | -1.6963 | -1.14 | 0.2528 |
| AT:CG | 1 | 0.12 | -0.4167 | -0.4 | -0.419 | 0.019 | 0.01 | 0.9957 |
| AT:RT | 2 | 0.6 | -2.3218 | -3.0264 | -1.2866 | -1.7398 | -0.74 | 0.4572 |
| CG:EMS+Nu | 2 | 0.87 | -1.7875 | -1.6944 | -2.4016 | 0.7071 | 0.24 | 0.8123 |
| CG:Nu | 18 | 0.84 | -1.2983 | -1.2607 | -1.5006 | 0.2398 | 0.22 | 0.8262 |
| CG:RBT | 4 | 0.69 | -2.1152 | -1.7007 | -3.0504 | 1.3497 | 0.83 | 0.4093 |
| CG:RT | 12 | 0.68 | -1.9051 | -2.0208 | -1.6639 | -0.357 | -0.34 | 0.7328 |
| CG:RT+Nu | 3 | 0.34 | -2.5007 | -1.1016 | -3.2318 | 2.1302 | 1.62 | 0.1047 |
| CG:TCEs | 4 | 0.92 | -1.7788 | -1.8585 | -0.8852 | -0.9733 | -0.31 | 0.755 |
| EMS+Nu:Nu | 1 | 0.46 | 0.4892 | 1 | 0.0463 | 0.9537 | 0.46 | 0.6472 |
| Nu:RT | 3 | 0.34 | -0.6068 | -0.1371 | -0.8462 | 0.7091 | 0.6 | 0.5456 |
| Nu:RT+Nu | 5 | 0.71 | -1.2024 | -1.4512 | -0.591 | -0.8602 | -0.64 | 0.5239 |
| RBT:RT | 1 | 0.23 | 0.2101 | 2.8 | -0.5445 | 3.3445 | 1.64 | 0.1007 |
| RT:RT+Nu | 7 | 0.73 | -0.5956 | -0.619 | -0.5316 | -0.0874 | -0.06 | 0.9506 |
| RT:TCEs | 1 | 0.34 | 0.1264 | 0.11 | 0.1349 | -0.0249 | -0.01 | 0.9899 |

**Table S4.3:** Side-splitting of Knee extension strength. Inconsistency test between direct and indirect treatment comparisons in mixed treatment comparison.

| comparison | k | prop | nma | direct | indir. | Diff | z | p-value |
| --- | --- | --- | --- | --- | --- | --- | --- | --- |
| ART:CG | 3 | 0.86 | 0.4858 | 0.4423 | 0.7449 | -0.3027 | -0.46 | 0.6435 |
| ART:Nu | 1 | 0.39 | 0.1418 | 0.0923 | 0.1731 | -0.0808 | -0.15 | 0.8776 |
| ART:RT | 1 | 0.27 | -0.5101 | -0.3662 | -0.5627 | 0.1965 | 0.34 | 0.7343 |
| CG:Nu | 6 | 0.81 | -0.344 | -0.2117 | -0.8989 | 0.6871 | 1.74 | 0.0817 |
| CG:RBT | 2 | 0.88 | -0.403 | -0.4191 | -0.2882 | -0.1309 | -0.16 | 0.8753 |
| CG:RT | 10 | 0.79 | -0.996 | -1.1363 | -0.4821 | -0.6542 | -1.82 | 0.0684 |
| CG:RT+Nu | 2 | 0.34 | -1.0184 | -0.9936 | -1.031 | 0.0373 | 0.09 | 0.9288 |
| CG:WBV | 6 | 0.95 | -0.571 | -0.5942 | -0.14 | -0.4542 | -0.53 | 0.5965 |
| Nu:RBT | 1 | 0.51 | -0.0591 | -0.1135 | -0.002 | -0.1116 | -0.19 | 0.848 |
| Nu:RT | 3 | 0.46 | -0.652 | -0.3193 | -0.9298 | 0.6104 | 1.68 | 0.0928 |
| Nu:RT+Nu | 3 | 0.61 | -0.6744 | -0.5797 | -0.8236 | 0.2439 | 0.56 | 0.5727 |
| RT:RT+Nu | 7 | 0.92 | -0.0225 | -0.1074 | 0.9691 | -1.0764 | -1.74 | 0.0824 |
| RT:WBV | 1 | 0.17 | 0.425 | -0.114 | 0.5371 | -0.6511 | -1.06 | 0.2881 |

**Table S4.4:** Side-splitting of Gait speed. Inconsistency test between direct and indirect treatment comparisons in mixed treatment comparison.

| comparison | k | prop | nma | direct | indir. | Diff | z | p-value |
| --- | --- | --- | --- | --- | --- | --- | --- | --- |
| ART:ART+Nu | 4 | 0.8 | 0.0299 | 0.0473 | -0.038 | 0.0853 | 0.9 | 0.3706 |
| ART:AT | 1 | 0.74 | 0.043 | 0.02 | 0.1082 | -0.0882 | -0.59 | 0.5546 |
| ART:CG | 7 | 0.82 | 0.0846 | 0.0738 | 0.1348 | -0.0609 | -0.77 | 0.4403 |
| ART:Nu | 3 | 0.5 | 0.0455 | 0.0431 | 0.048 | -0.0049 | -0.08 | 0.9399 |
| ART+Nu:CG | 5 | 0.84 | 0.0547 | 0.0449 | 0.1071 | -0.0622 | -0.67 | 0.5006 |
| ART+Nu:Nu | 3 | 0.54 | 0.0156 | -0.0188 | 0.0564 | -0.0753 | -1.05 | 0.2941 |
| AT:RT | 1 | 0.33 | -0.0109 | -0.07 | 0.0182 | -0.0882 | -0.59 | 0.5546 |
| CG:EMS | 1 | 0.73 | -0.0897 | -0.11 | -0.0361 | -0.0739 | -0.47 | 0.6377 |
| CG:EMS+Nu | 2 | 0.97 | -0.0532 | -0.0487 | -0.2001 | 0.1514 | 0.5 | 0.6158 |
| CG:Nu | 15 | 0.85 | -0.039 | -0.0296 | -0.0915 | 0.062 | 1.19 | 0.2358 |
| CG:RBT | 3 | 0.53 | -0.1071 | -0.1382 | -0.0725 | -0.0657 | -0.85 | 0.3935 |
| CG:RBT+Nu | 2 | 0.43 | -0.2003 | -0.1259 | -0.2564 | 0.1305 | 1.43 | 0.1516 |
| CG:RT | 8 | 0.62 | -0.0525 | -0.0615 | -0.0375 | -0.024 | -0.45 | 0.6518 |
| CG:RT+Nu | 3 | 0.27 | -0.1148 | -0.1909 | -0.086 | -0.1049 | -1.57 | 0.1153 |
| EMS:EMS+Nu | 2 | 0.89 | 0.0365 | 0.0301 | 0.0881 | -0.058 | -0.28 | 0.7811 |
| Nu:RBT | 1 | 0.24 | -0.0681 | -0.13 | -0.0487 | -0.0813 | -0.86 | 0.3925 |
| Nu:RBT+Nu | 1 | 0.29 | -0.1613 | -0.1 | -0.1863 | 0.0863 | 0.84 | 0.402 |
| Nu:RT | 3 | 0.26 | -0.0135 | -0.0558 | 0.0016 | -0.0573 | -0.9 | 0.3692 |
| Nu:RT+Nu | 5 | 0.64 | -0.0758 | -0.0644 | -0.0961 | 0.0317 | 0.52 | 0.6032 |
| RBT:RBT+Nu | 6 | 0.89 | -0.0932 | -0.1029 | -0.0116 | -0.0913 | -0.79 | 0.4298 |
| RBT:RT | 1 | 0.32 | 0.0546 | 0.07 | 0.0473 | 0.0227 | 0.25 | 0.7998 |
| RT:RT+Nu | 9 | 0.75 | -0.0623 | -0.0696 | -0.04 | -0.0296 | -0.45 | 0.6537 |

**Table S4.5:** Side-splitting of Five chair stand time. Inconsistency test between direct and indirect treatment comparisons in mixed treatment comparison.

| comparison | k | prop | nma | direct | indir. | Diff | z | p-value |
| --- | --- | --- | --- | --- | --- | --- | --- | --- |
| ART:ART+Nu | 2 | 0.89 | 1.236 | 0.8683 | 4.1212 | -3.253 | -1.07 | 0.2826 |
| ART:CG | 3 | 0.91 | -1.6271 | -1.6187 | -1.7102 | 0.0915 | 0.04 | 0.9703 |
| ART:Nu | 1 | 0.4 | -1.2049 | -0.44 | -1.725 | 1.285 | 0.82 | 0.4134 |
| ART+Nu:CG | 2 | 0.86 | -2.8631 | -2.9764 | -2.1544 | -0.822 | -0.33 | 0.7421 |
| ART+Nu:Nu | 1 | 0.5 | -2.4408 | -1.78 | -3.0985 | 1.3185 | 0.73 | 0.4665 |
| CG:Nu | 8 | 0.95 | 0.4222 | 0.3545 | 1.7177 | -1.3633 | -0.73 | 0.4647 |
| CG:RT | 2 | 0.58 | 1.2558 | 1.3406 | 1.1382 | 0.2024 | 0.16 | 0.871 |
| CG:RT+Nu | 3 | 0.73 | 1.9788 | 1.6434 | 2.8975 | -1.2541 | -0.95 | 0.3401 |
| Nu:RT | 2 | 0.58 | 0.8336 | 1.3197 | 0.1624 | 1.1573 | 0.88 | 0.3782 |
| Nu:RT+Nu | 2 | 0.51 | 1.5566 | 1.8357 | 1.2667 | 0.569 | 0.45 | 0.6491 |
| RT:RT+Nu | 7 | 0.97 | 0.723 | 0.8019 | -1.456 | 2.2578 | 0.86 | 0.3895 |

**Table S4.6:** Side-splitting of Timed up and go. Inconsistency test between direct and indirect treatment comparisons in mixed treatment comparison.

| comparison | k | prop | nma | direct | indir. | Diff | z | p-value |
| --- | --- | --- | --- | --- | --- | --- | --- | --- |
| CG:Nu | 3 | 0.89 | 0.022 | -0.078 | 0.8045 | -0.8825 | -0.96 | 0.3385 |
| CG:RBT | 2 | 1 | 2.2861 | 2.3067 | -3.1774 | 5.4841 | 0.75 | 0.4512 |
| CG:RT | 6 | 0.9 | 0.6274 | 0.7045 | -0.0402 | 0.7447 | 1.05 | 0.2937 |
| CG:RT+Nu | 2 | 0.64 | 0.843 | 0.5117 | 1.4196 | -0.9079 | -1.41 | 0.1571 |
| Nu:RT | 1 | 0.51 | 0.6055 | 0.5 | 0.7163 | -0.2163 | -0.33 | 0.7387 |
| Nu:RT+Nu | 1 | 0.63 | 0.821 | 0.6 | 1.2014 | -0.6014 | -0.78 | 0.435 |
| RBT:RT | 1 | 0.01 | -1.6587 | 3.8 | -1.6841 | 5.4841 | 0.75 | 0.4512 |
| RT:RT+Nu | 3 | 0.78 | 0.2155 | 0.4023 | -0.4602 | 0.8625 | 1.17 | 0.2413 |

**Table S4.7:** Side-splitting of Balance test. Inconsistency test between direct and indirect treatment comparisons in mixed treatment comparison.

| Comparison | k | prop | nma | direct | indir. | Diff | z | p-value |
| --- | --- | --- | --- | --- | --- | --- | --- | --- |
| CG:ARBT | 2 | 0.84 | -0.733 | -0.4367 | -2.3046 | 1.8679 | 2.2 | 0.0275 |
| RBT:ARBT | 1 | 0.59 | -0.0424 | -0.8121 | 1.0558 | -1.8679 | -2.2 | 0.0275 |
| ART:CG | 2 | 0.89 | 0.176 | 0.1939 | 0.0298 | 0.1641 | 0.16 | 0.8716 |
| ART:Nu | 1 | 0.54 | -0.0903 | -0.0402 | -0.1499 | 0.1096 | 0.16 | 0.875 |
| CG:Nu | 5 | 0.93 | -0.2663 | -0.2925 | 0.0651 | -0.3576 | -0.43 | 0.6705 |
| CG:RT | 6 | 0.89 | -0.3409 | -0.479 | 0.7393 | -1.2183 | -1.78 | 0.0759 |
| CG:RT+Nu | 2 | 0.86 | -0.0751 | 0.0684 | -0.9769 | 1.0454 | 0.98 | 0.3266 |
| Nu:RT | 1 | 0.21 | -0.0746 | -0.1341 | -0.0591 | -0.075 | -0.1 | 0.9191 |
| Nu:RT+Nu | 1 | 0.39 | 0.1912 | -0.2606 | 0.4747 | -0.7354 | -0.88 | 0.381 |
| RBT:RT | 1 | 0.64 | 0.3498 | 1.019 | -0.8488 | 1.8679 | 2.2 | 0.0275 |
| RT:RT+Nu | 1 | 0.39 | 0.2659 | -0.1265 | 0.5116 | -0.6381 | -0.76 | 0.4466 |

**Table S4.8.:** Side-splitting of ASMI. Inconsistency test between direct and indirect treatment comparisons in mixed treatment comparison.

| comparison | k | prop | nma | direct | indir. | Diff | z | p-value |
| --- | --- | --- | --- | --- | --- | --- | --- | --- |
| ART+Nu:ART | 3 | 0.95 | 0.0837 | 0.079 | 0.1811 | -0.1021 | -0.23 | 0.8173 |
| CG:ART | 4 | 0.78 | -0.0366 | -0.0949 | 0.1737 | -0.2686 | -1.44 | 0.1492 |
| Nu:ART | 2 | 0.52 | 0.0366 | 0.1343 | -0.0705 | 0.2048 | 1.32 | 0.186 |
| ART+Nu:CG | 3 | 0.8 | 0.1203 | 0.1891 | -0.149 | 0.3381 | 1.55 | 0.1205 |
| ART+Nu:Nu | 2 | 0.54 | 0.0471 | -0.0152 | 0.121 | -0.1362 | -0.77 | 0.4419 |
| CG:Nu | 8 | 0.93 | -0.0732 | -0.0699 | -0.1182 | 0.0483 | 0.63 | 0.5283 |
| CG:RT | 7 | 0.71 | -0.1579 | -0.1461 | -0.1862 | 0.0401 | 0.54 | 0.5866 |
| CG:RT+Nu | 2 | 0.38 | -0.3251 | -0.2781 | -0.3545 | 0.0764 | 1.26 | 0.2092 |
| Nu:RT | 2 | 0.49 | -0.0847 | -0.0867 | -0.0828 | -0.0038 | -0.06 | 0.9543 |
| Nu:RT+Nu | 5 | 0.84 | -0.252 | -0.2594 | -0.2117 | -0.0477 | -0.65 | 0.5146 |
| RT:RT+Nu | 7 | 0.78 | -0.1672 | -0.1768 | -0.1332 | -0.0436 | -0.53 | 0.5983 |

**Table S4.9.:** Side-splitting of SMI. Inconsistency test between direct and indirect treatment comparisons in mixed treatment comparison.

| Comparison | k | prop | nma | direct | indir. | Diff | z | p-value |
| --- | --- | --- | --- | --- | --- | --- | --- | --- |
| ART+Nu:CG | 2 | 1 | 0.5028 | 0.5054 | -1.1756 | 1.681 | 0.54 | 0.5878 |
| ART+Nu:Nu | 2 | 1 | 0.0231 | 0.0252 | -0.7616 | 0.7868 | 0.33 | 0.7442 |
| CG:Nu | 3 | 1 | -0.4798 | -0.4776 | -2.1468 | 1.6692 | 0.67 | 0.5047 |

# Appendix 5: Network maps and forest plots

**Figure S5.1:** Network map of the effect on Grip strength, and forest plot of network effect sizes for compared with control. The size of the nodes was proportional to the number of participants included in the trial, and the thickness of lines between the interventions relates to the number of studies for that comparison.


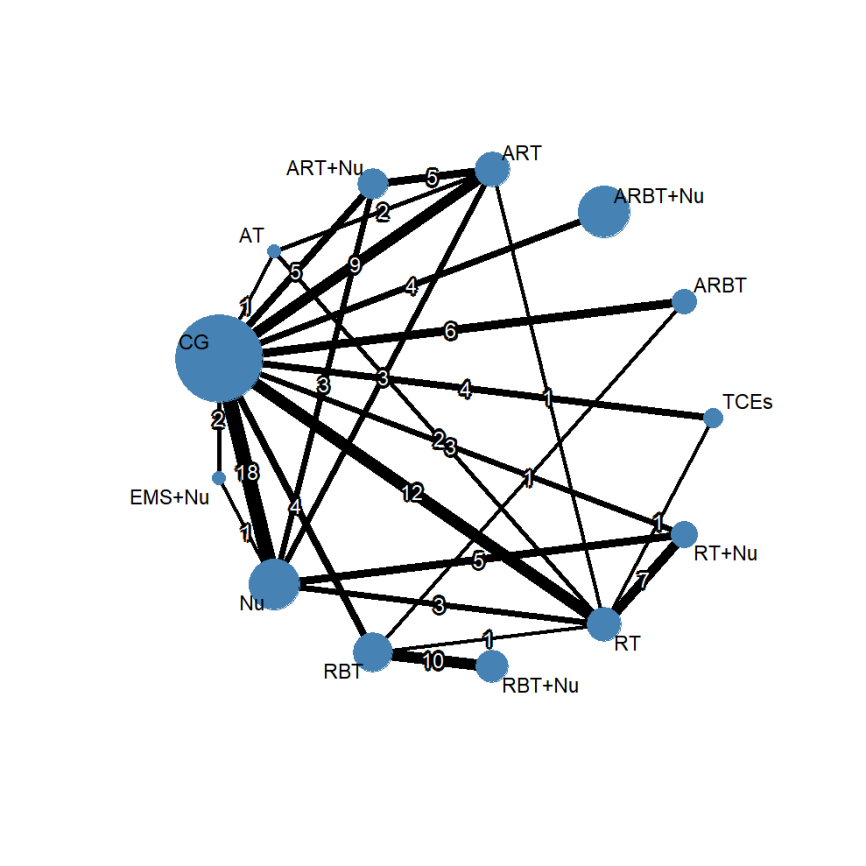

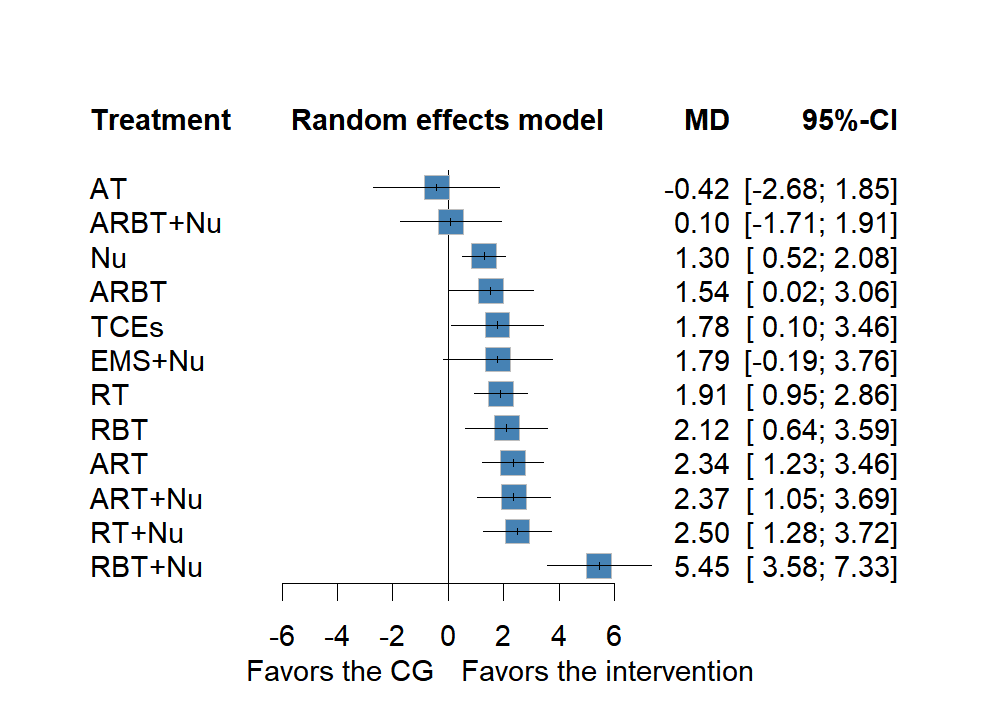


**Figure S5.2:** Network map of the effect on Knee extension strength, and forest plot of network effect sizes for compared with control. The size of the nodes was proportional to the number of participants included in the trial, and the thickness of lines between the interventions relates to the number of studies for that comparison.


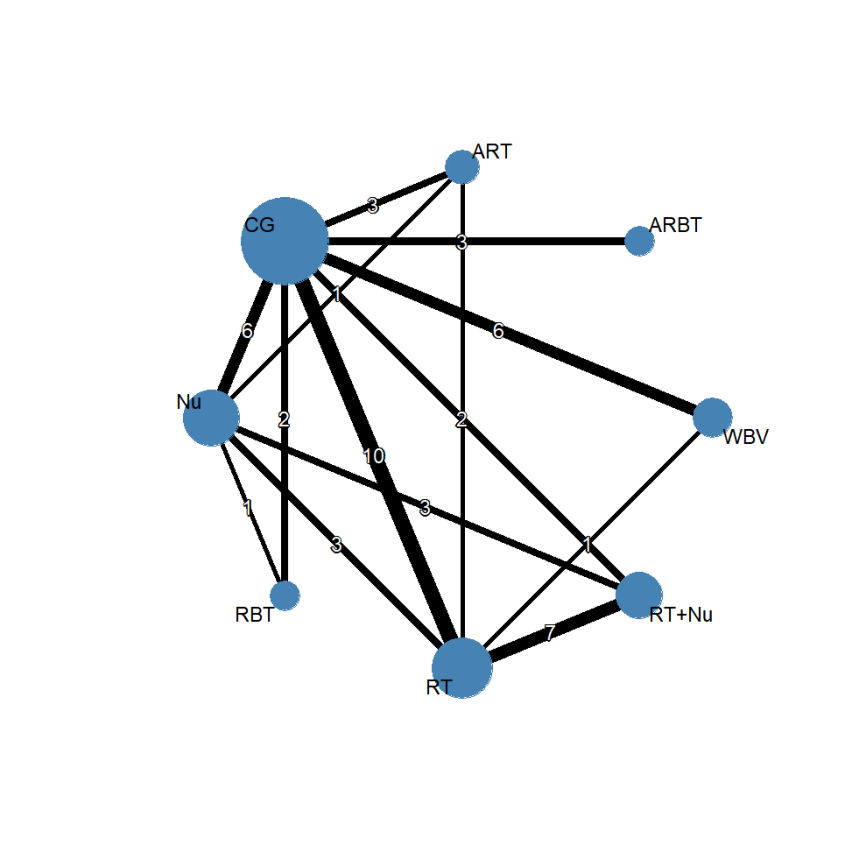


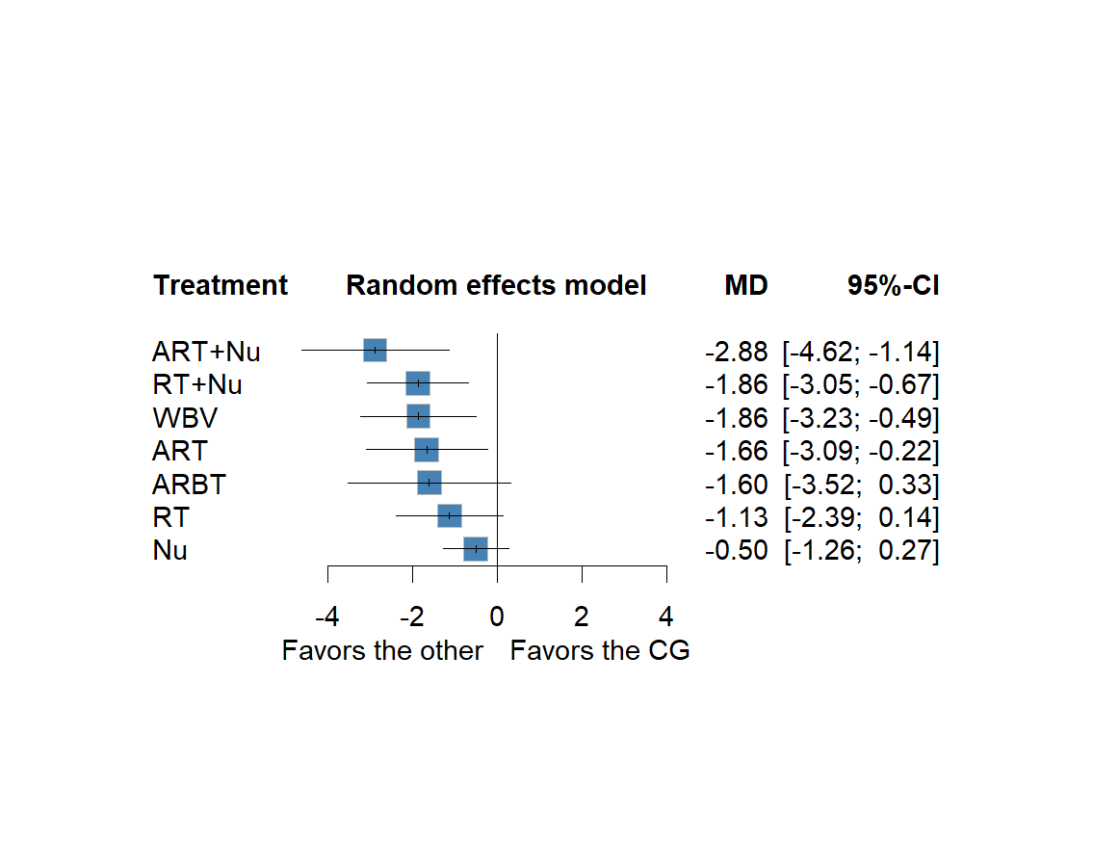


**Figure S5.3:** Network map of the effect on Gait speed, and forest plot of network effect sizes for compared with control. The size of the nodes was proportional to the number of participants included in the trial, and the thickness of lines between the interventions relates to the number of studies for that comparison.

**
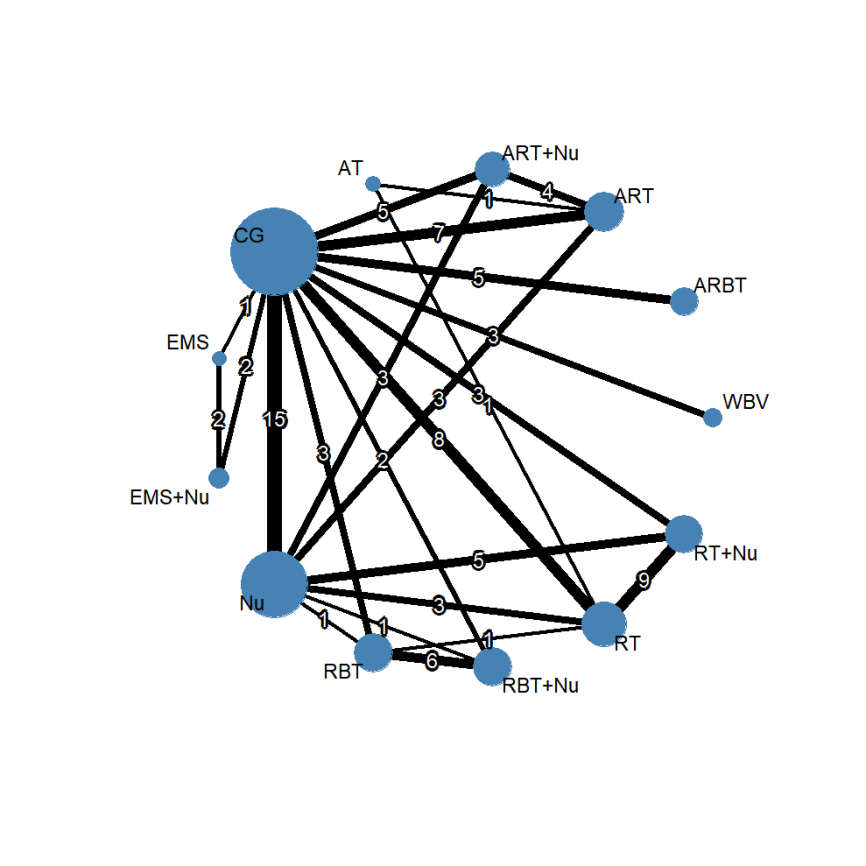
**

**
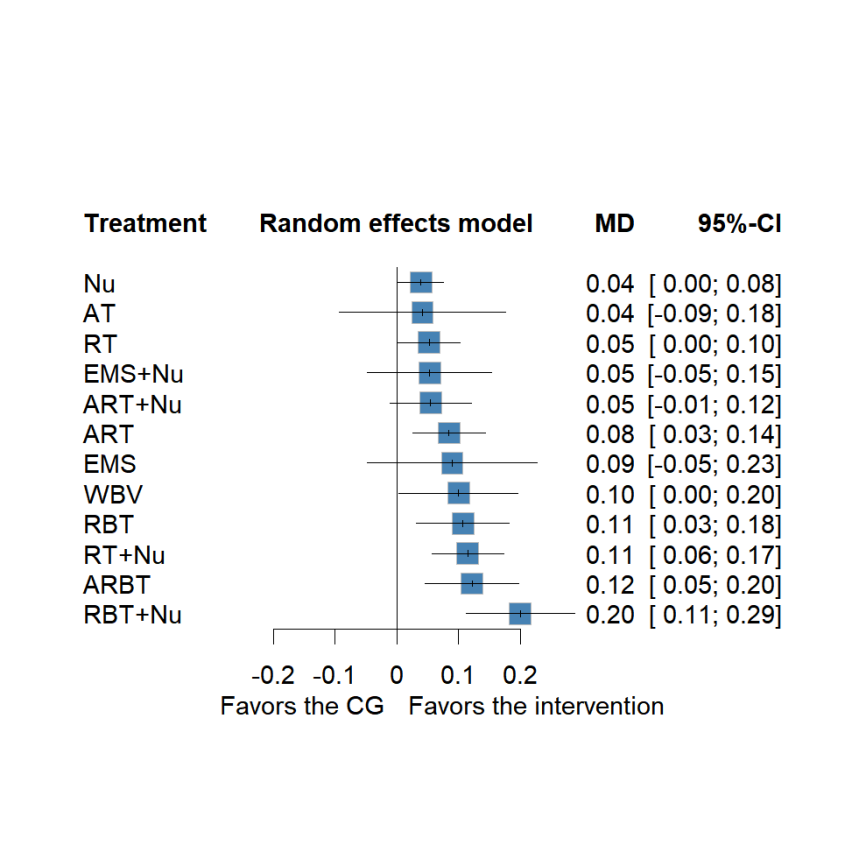
**

**Figure S5.4:** Network map of the effect on Five-Times Sit-to-Stand test, and forest plot of network effect sizes for compared with control. The size of the nodes was proportional to the number of participants included in the trial, and the thickness of lines between the interventions relates to the number of studies for that comparison.

**
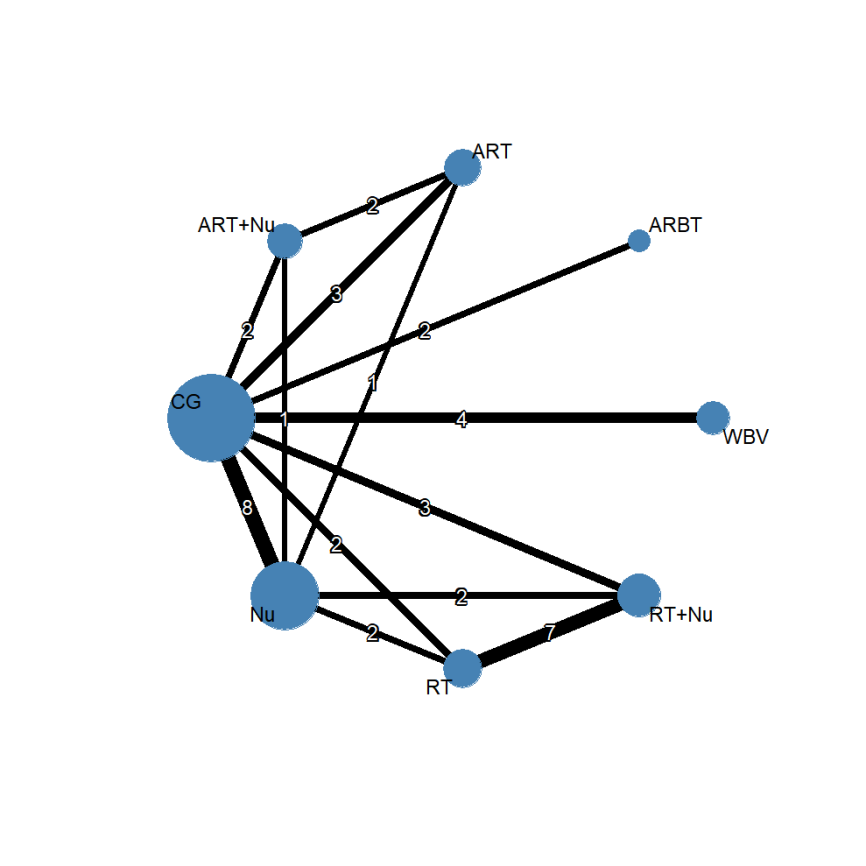

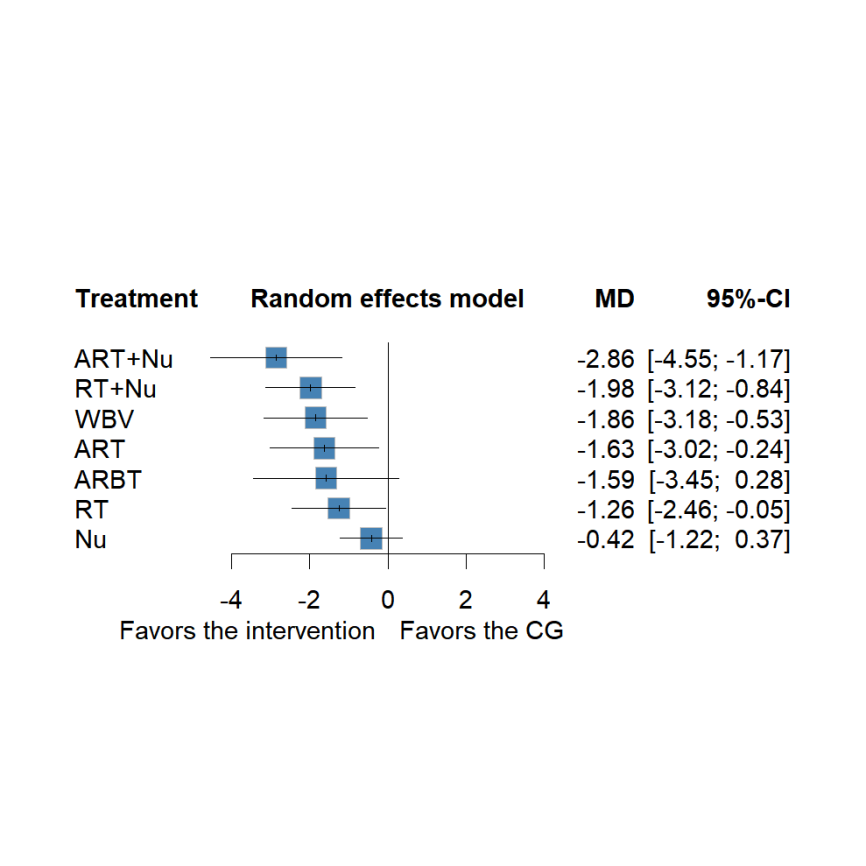
**

**Figure S5.5:** Network map of the effect on Timed up and go, and forest plot of network effect sizes for compared with control. The size of the nodes was proportional to the number of participants included in the trial, and the thickness of lines between the interventions relates to the number of studies for that comparison.


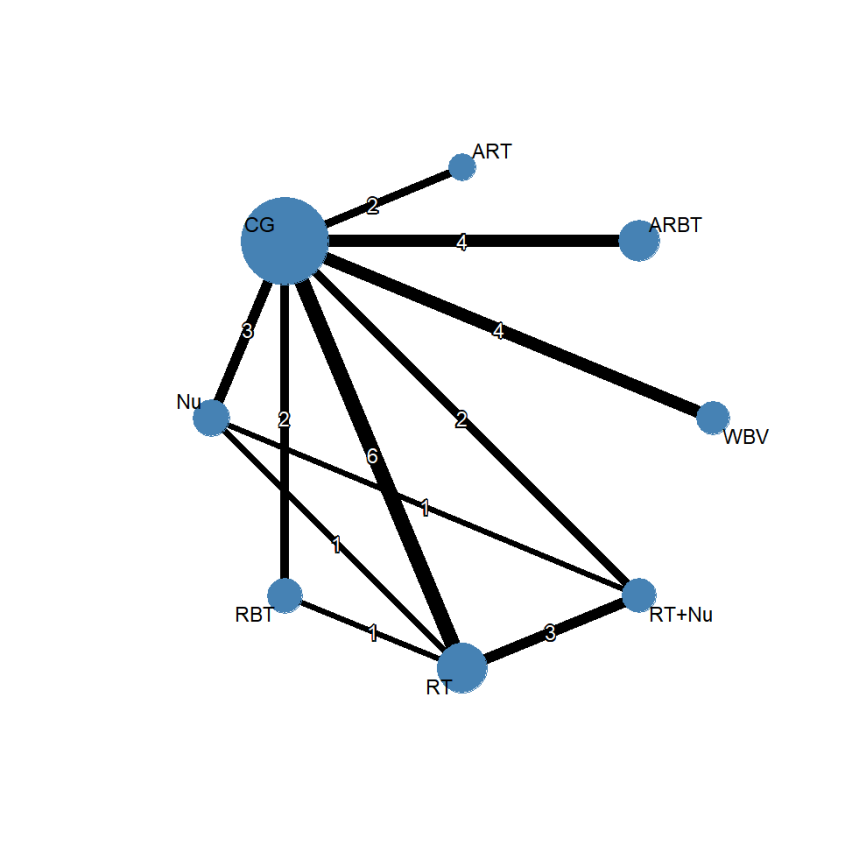


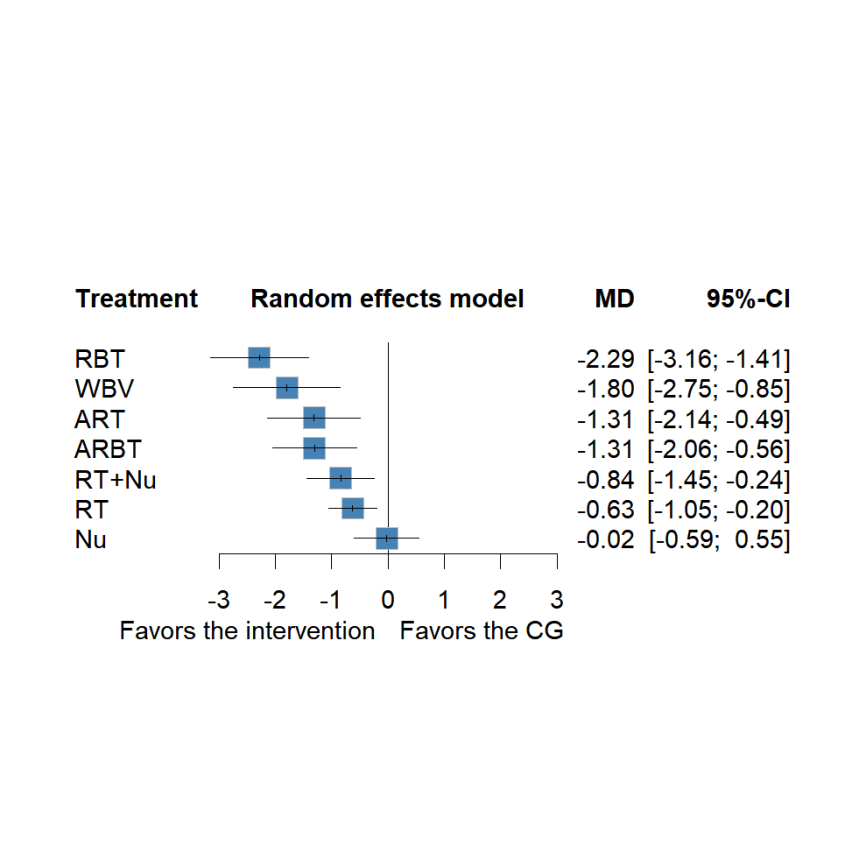


**Figure S5.6:** Network map of the effect on SPPB, and forest plot of network effect sizes for compared with control. The size of the nodes was proportional to the number of participants included in the trial, and the thickness of lines between the interventions relates to the number of studies for that comparison.


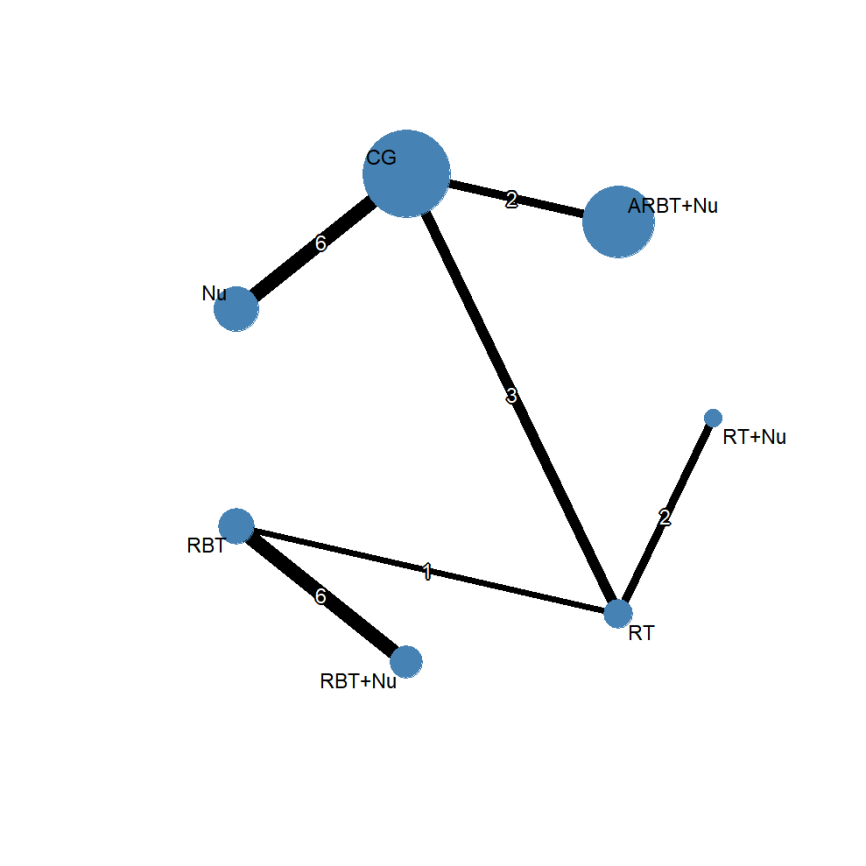


**
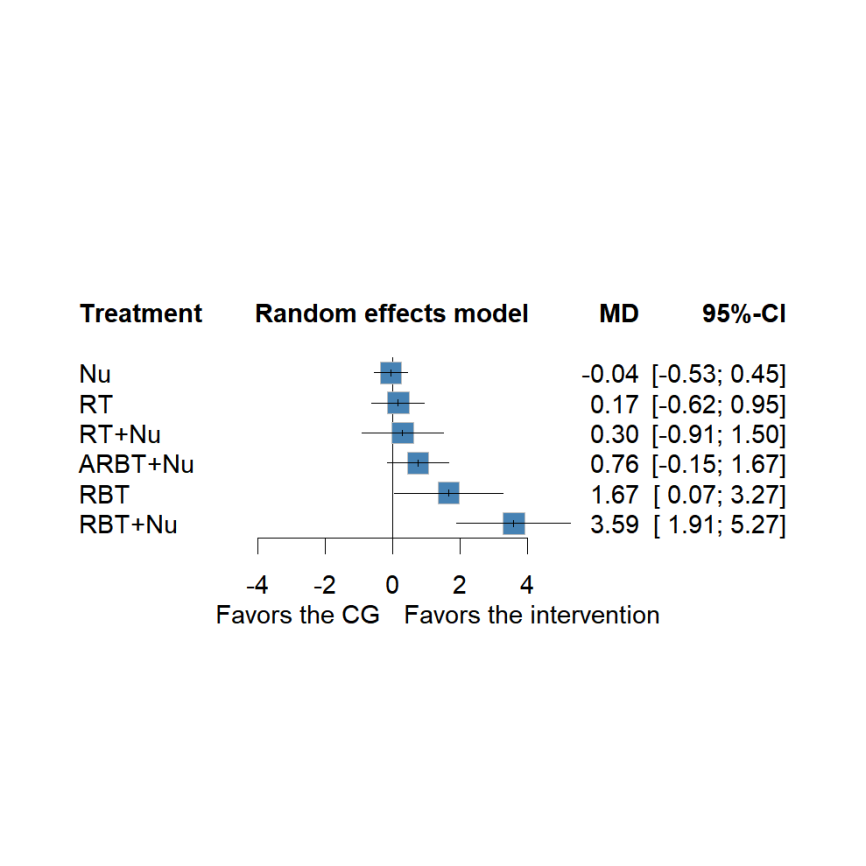
**

**Figure S5.7:** Network map of the effect on Balance test, and forest plot of network effect sizes for compared with control. The size of the nodes was proportional to the number of participants included in the trial, and the thickness of lines between the interventions relates to the number of studies for that comparison.


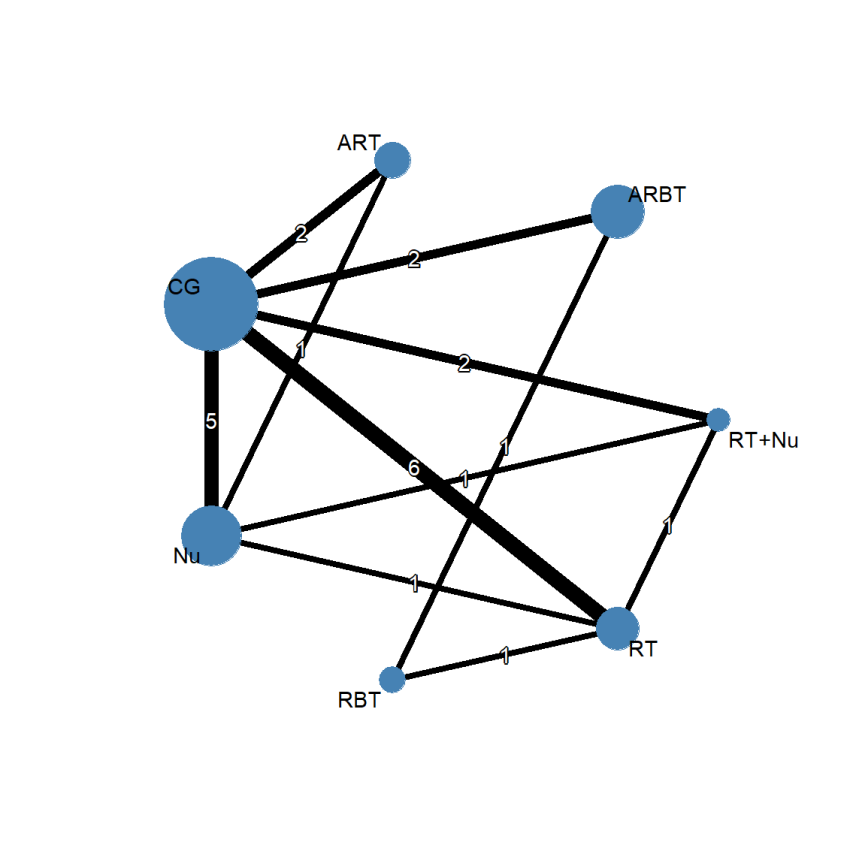


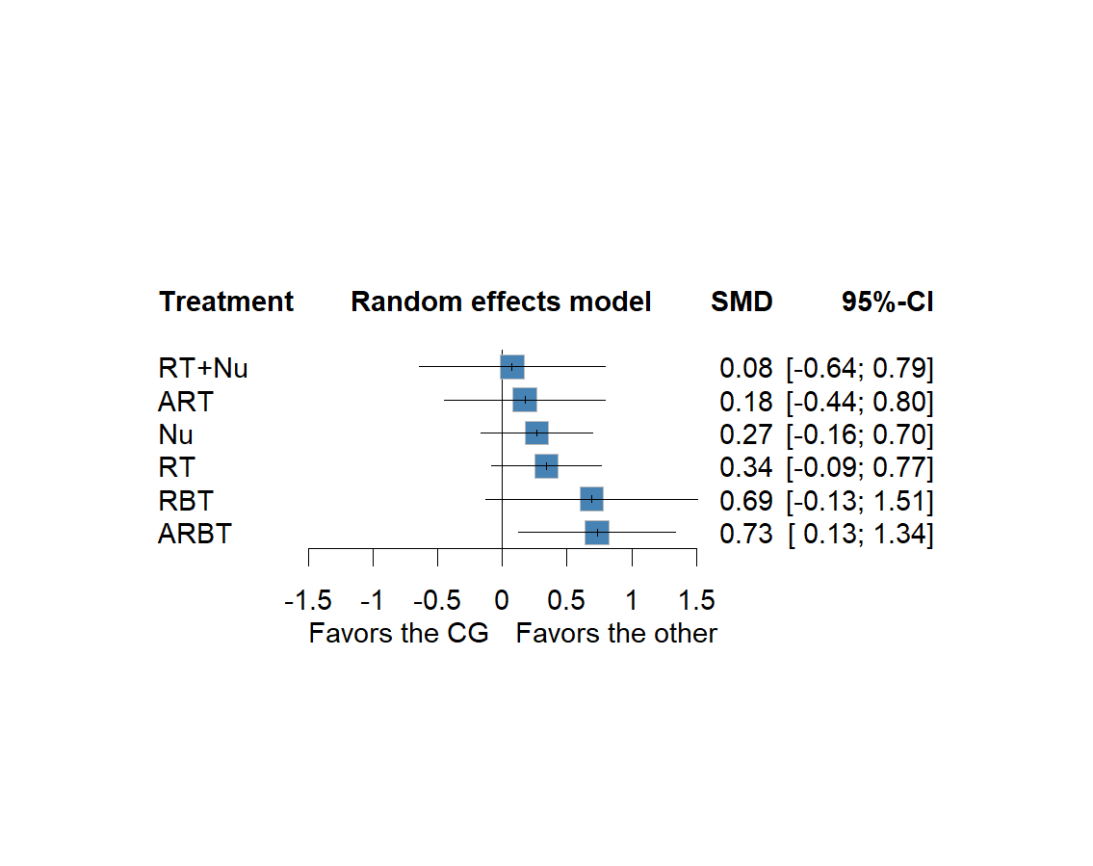


**Figure S5.9:** Network map of the effect on ASMI, and forest plot of network effect sizes for compared with control. The size of the nodes was proportional to the number of participants included in the trial, and the thickness of lines between the interventions relates to the number of studies for that comparison.

**
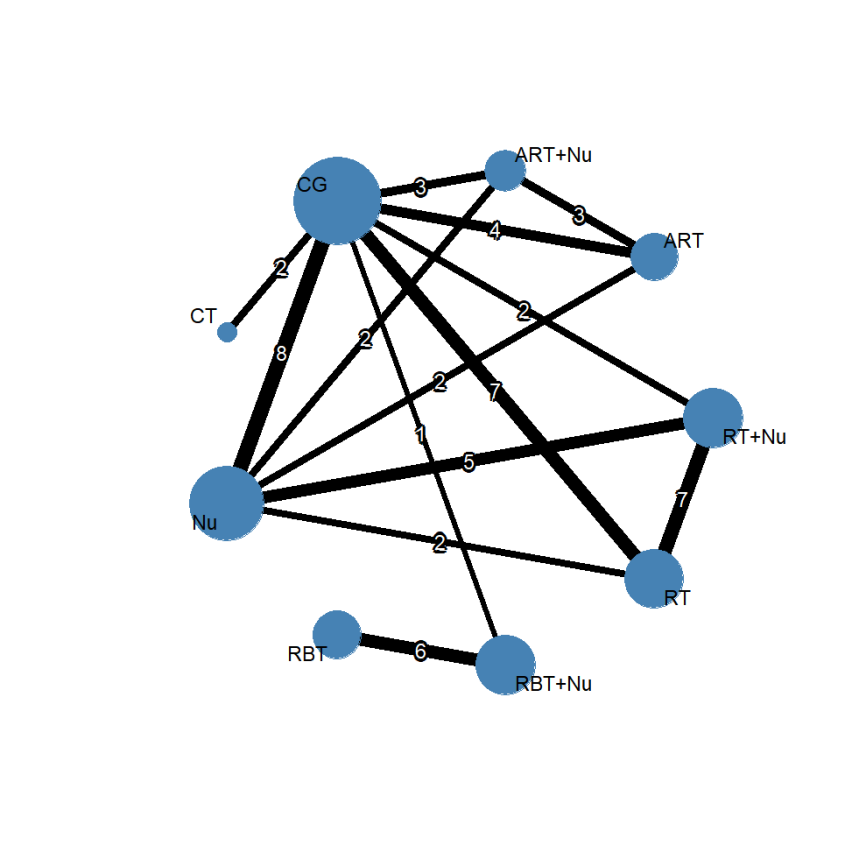

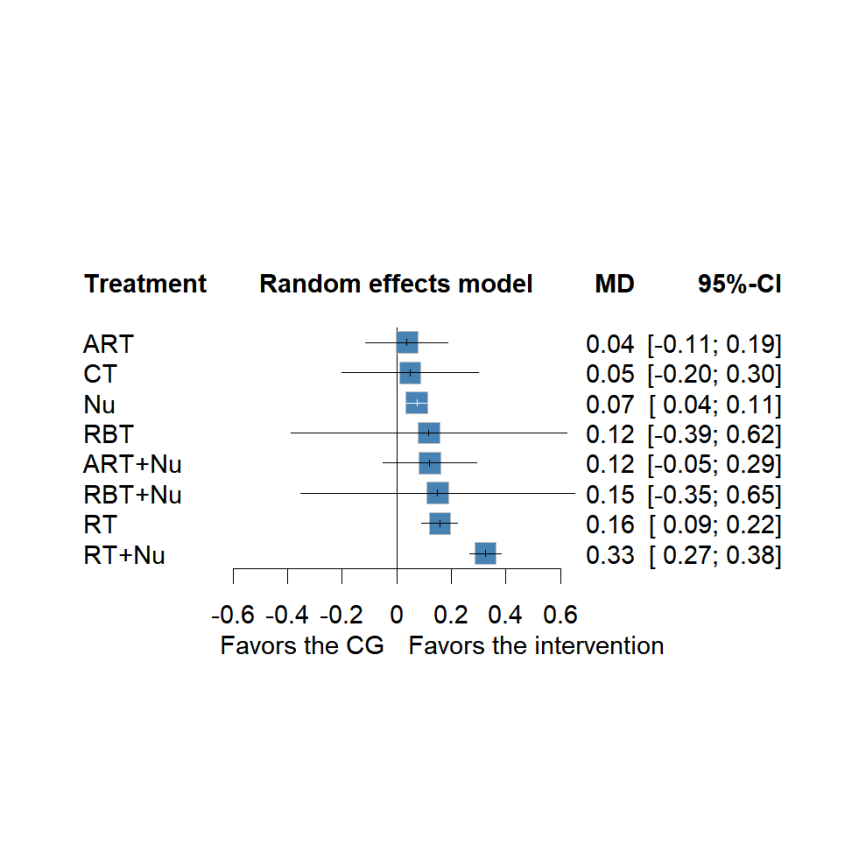
**

**Figure S5.9:** Network map of the effect on SMI, and forest plot of network effect sizes for compared with control. The size of the nodes was proportional to the number of participants included in the trial, and the thickness of lines between the interventions relates to the number of studies for that comparison.


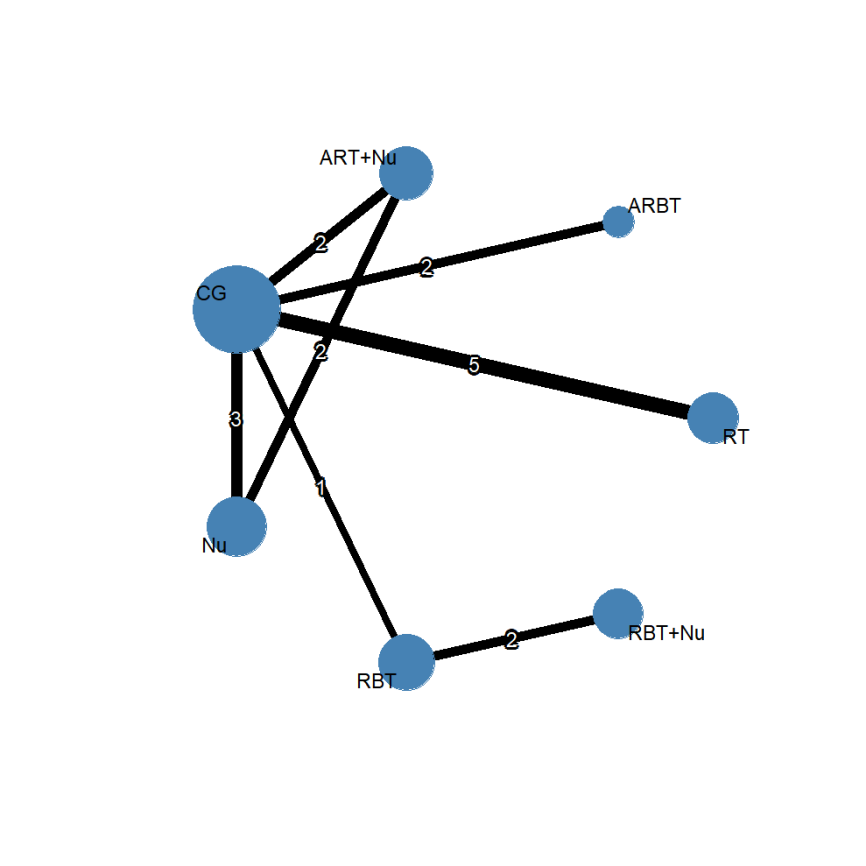


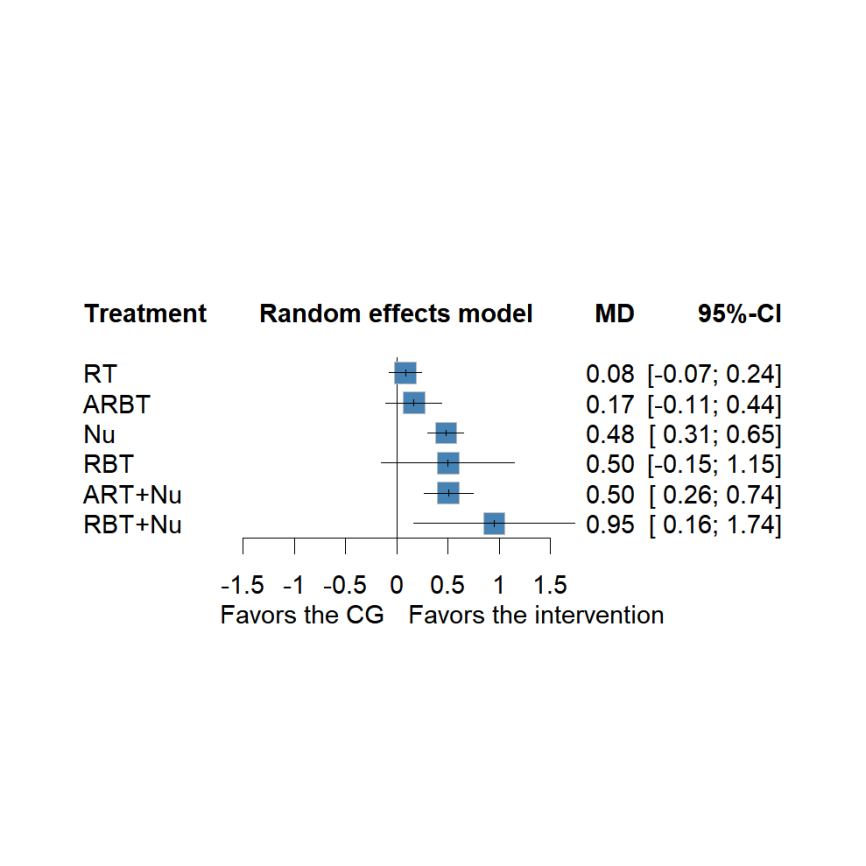


**Figure S5.7:** Network map of the effect on Adherence, and forest plot of network effect sizes for compared with control. The size of the nodes was proportional to the number of participants included in the trial, and the thickness of lines between the interventions relates to the number of studies for that comparison.

**
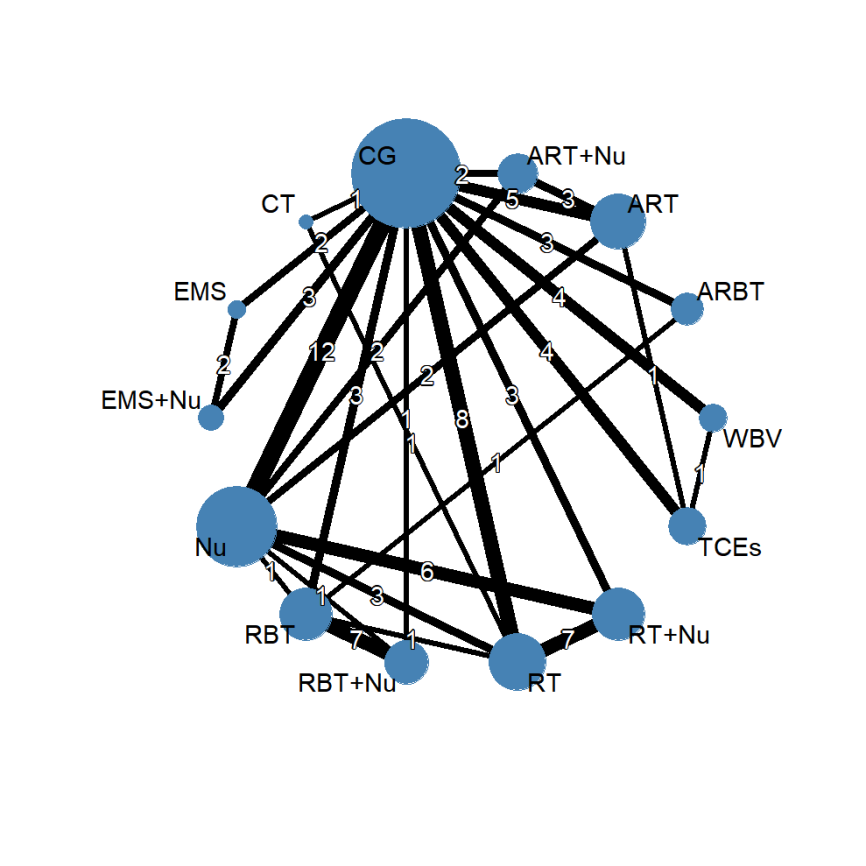

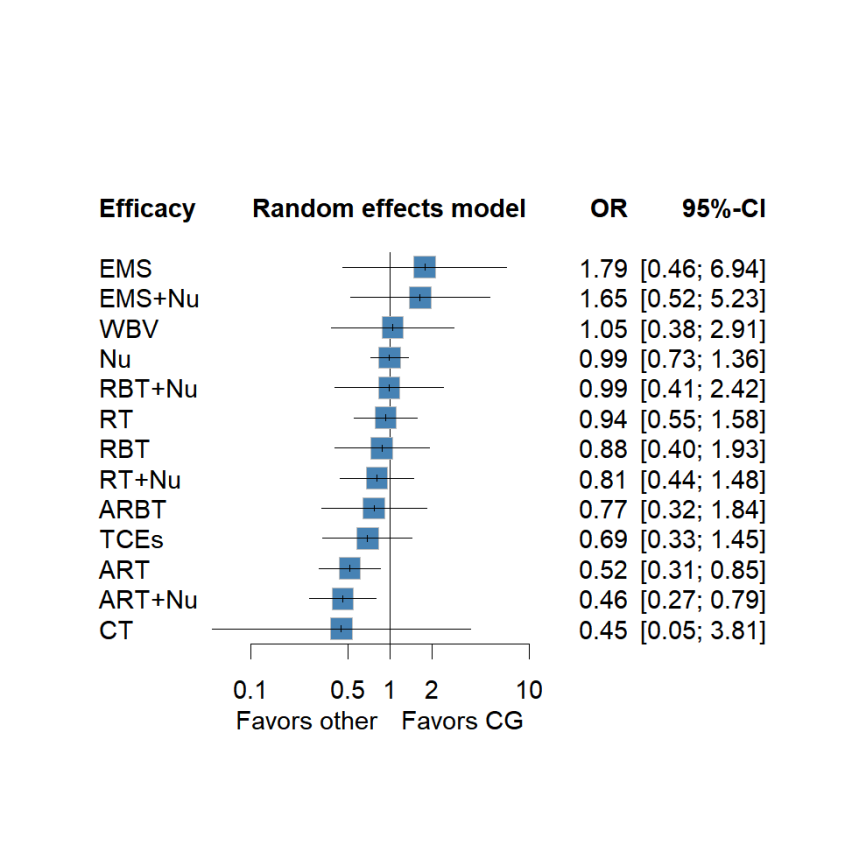
**

**Appendix 6: Minimally contextualized framework**

**Table S6.1 Classification of Interventions Based on Partially Contextualized Framework (Threshold = MID)**

| **Outcome** | **Certainty of Evidence** | **Group** | **Intervention** | **Intervention vs Control** | **Bayesian SUCRA** |
| --- | --- | --- | --- | --- | --- |
| Grip strength | High certainty (moderate to high certainty evidence) | Category 2: Probably clinically important | RBT+Nu | 5.45 ( 3.58; 7.33) | 99.85 |
|  | Low certainty (low to very low certainty evidence) | Category 1: Effective but not clinically important | RT+Nu | 2.50 ( 1.28; 3.72) | 74.11 |
|  |  |  | ART+Nu | 2.37 ( 1.05; 3.69) | 69.67 |
|  |  |  | ART | 2.34 ( 1.23; 3.46) | 69.6 |
|  |  |  | RBT | 2.12 ( 0.64; 3.59) | 61.98 |
|  |  |  | RT | 1.91 ( 0.95; 2.86) | 55.62 |
|  |  |  | TCEs | 1.78 ( 0.10; 3.46) | 52.18 |
|  |  |  | ARBT | 1.54 ( 0.02; 3.06) | 45.27 |
|  |  |  | Nu | 1.30 ( 0.52; 2.08) | 35.6 |
|  |  | Category 0: Ineffective or uncertain | EMS+Nu | 1.79 (-0.19; 3.76) | 52.47 |
|  |  |  | ARBT+Nu | 0.15(-1.64；1.94) | 14.64 |
|  |  |  | AT | -0.42 (-2.68; 1.85) | 9.16 |
| Gait speed | High certainty (moderate to high certainty evidence) | Category 3: Clinically important effect | RBT+Nu | 0.20 ( 0.11; 0.29) | 96.92 |
|  | Low certainty (low to very low certainty evidence) | Category 2: Possibly clinically important | ARBT | 0.12 ( 0.05; 0.20) | 73.7 |
|  |  |  | RT+Nu | 0.11 ( 0.06; 0.17) | 72.07 |
|  |  |  | RBT | 0.11 ( 0.03; 0.18) | 66.22 |
|  |  |  | WBV | 0.10 ( 0.00; 0.20) | 61.42 |
|  |  |  | ART | 0.08 ( 0.03; 0.14) | 54.98 |
|  |  |  | RT | 0.05 ( 0.00; 0.10) | 34.03 |
|  |  | Category 1: Effective but not clinically important | Nu | 0.04 ( 0.00; 0.08) | 25.29 |
|  |  | Category 0: Ineffective or uncertain | EMS | 0.09 (-0.05; 0.23) | 55.19 |
|  |  |  | ART+Nu | 0.05(-0.04,0.14) | 35.98 |
|  |  |  | EMS+Nu | 0.05 (-0.05; 0.15) | 36.29 |
|  |  |  | AT | 0.04 (-0.09; 0.18) | 32.55 |
| Timed up and go | High certainty (moderate to high certainty evidence) | Category 2: Possibly clinically important | RBT | -2.29 (-3.16; -1.41) | 95.4 |
|  |  |  | WBV | -1.80 (-2.75; -0.85) | 81.67 |
|  |  |  | ARBT | -1.31 (-2.14; -0.49) | 64.57 |
|  |  | Category 1: Effective but not clinically important | ART | -1.31 (-2.06; -0.56) | 64.53 |
|  | Low certainty (low to very low certainty evidence) | Category 1: Effective but not clinically important | RT+Nu | -0.84 (-1.45; -0.24) | 45.06 |
|  |  |  | RT | -0.63 (-1.05; -0.20) | 33.58 |
|  |  | Category 0: Ineffective or uncertain | Nu | -0.02 (-0.59; 0.55) | 8.37 |
| 5-times sit-to-stand | Low certainty (low to very low certainty evidence) | Category 2: Possibly clinically effective | ART+Nu | -2.86 (-4.55; -1.17) | 90.04 |
|  |  |  | RT+Nu | -1.98 (-3.12; -0.84) | 70.93 |
|  |  |  | WBV | -1.86 (-3.18; -0.53) | 64.48 |
|  |  |  | ART | -1.63 (-3.02; -0.24) | 56.5 |
|  |  | Category 0: Ineffective or uncertain | ARBT | -1.59 (-3.45; 0.28) | 55.04 |
|  |  |  | RT | -1.26 (-2.46; -0.05) | 42.77 |
|  |  |  | Nu | -0.42 (-1.22; 0.37) | 16.92 |
| SPPB | Low certainty (low to very low certainty evidence) | Category 3: Clearly clinically effective | RBT+Nu | 3.59 ( 1.91; 5.27) | 99.97 |
|  |  | Category 2: Possibly clinically important | RBT | 1.67 ( 0.07; 3.27) | 78.63 |
|  |  | Category 0: Ineffective or uncertain | ARBT+Nu | 0.76 (-0.15; 1.67) | 60.16 |
|  |  |  | RT+Nu | 0.30 (-0.91; 1.50) | 38.75 |
|  |  |  | RT | 0.17 (-0.62; 0.95) | 31.74 |
|  |  |  | Nu | -0.04 (-0.53; 0.45) | 19.22 |

**Table S6.2 Classification of Interventions Based on Minimally Contextualized Framework (Threshold = Null Effect)**

| **Outcome** | **Certainty of Evidence** | **Group** | **Intervention** | **Intervention vs Control** | **Bayesian SUCRA** |
| --- | --- | --- | --- | --- | --- |
| ASMI | High certainty (moderate to high certainty evidence) | Category 2: Among the most effective | RT+Nu | 0.33 ( 0.27; 0.38) | 93.87 |
|  |  | Category 1: Among the intermediate effective | RT | 0.16 ( 0.09; 0.22) | 68.11 |
|  | Low certainty (low to very low certainty evidence) | Category 1: Among the intermediate effective | Nu | 0.07 ( 0.04; 0.11) | 42.15 |
|  |  | Category 0: Among the least effective | RBT+Nu | 0.15 (-0.35; 0.65) | 59.88 |
|  |  |  | ART+Nu | 0.12 (-0.05; 0.29) | 55.36 |
|  |  |  | RBT | 0.12 (-0.39; 0.62) | 46.67 |
|  |  |  | CT | 0.05 (-0.20; 0.30) | 36.55 |
|  |  |  | ART | 0.04 (-0.11; 0.19) | 30.48 |
| SMI | High certainty (moderate to high certainty evidence) | Category 2: Among the most effective | RBT+Nu | 0.95 (0.16; 1.74) | 94.15 |
|  | Low certainty (low to very low certainty evidence) | Category 1: Among the intermediate effective | ART+Nu | 0.50 (0.26; 0.74) | 69.68 |
|  |  |  | Nu | 0.48 (0.31; 0.65) | 66.66 |
|  |  | Category 0: Among the least effective | RBT | 0.50 (-0.15,1.15) | 61.49 |
|  |  |  | ARBT | 0.17 (-0.11,0.44) | 30.97 |
|  |  |  | RT | 0.08 (-0.07,0.24) | 21.42 |
| Knee extension strength | High certainty (moderate to high certainty evidence) | Category 2: Among the most effective | RT+Nu | 0.98 ( 0.63; 1.33) | 90.58 |
|  |  | Category 1: Among the intermediate effective | WBV | 0.56 ( 0.22; 0.89) | 52.63 |
|  | Low certainty (low to very low certainty evidence) | Category 2: Among the most effective | RT | 0.97 ( 0.71; 1.24) | 89.57 |
|  |  | Category 1: Among the intermediate effective | ARBT | 0.59 ( 0.11; 1.08) | 54.42 |
|  |  |  | ART | 0.48 ( 0.08; 0.89) | 44.42 |
|  |  |  | Nu | 0.36 ( 0.08; 0.63) | 29.74 |
|  |  | Category 0: Among the least effective | RBT | 0.41 (-0.07; 0.88) | 36.99 |
| Balance test | High certainty (moderate to high certainty evidence) | Category 2: Among the most effective | ARBT | 0.73(0.13,1.34) | 84.3 |
|  | Low certainty (low to very low certainty evidence) | Category 0: Among the least effective | RBT | 0.69(-0.13,1.51) | 76.48 |
|  |  |  | RT | 0.34(-0.09,0.77) | 51.9 |
|  |  |  | Nu | 0.27(-0.16,0.70) | 46.25 |
|  |  |  | ART | 0.18(-0.44,0.80) | 41.36 |
|  |  |  | RT+Nu | 0.08(-0.64,0.79) | 31.62 |

| **Outcome** | **Certainty of Evidence** | **Group** | **Intervention** | **Intervention vs Control** | **Bayesian SUCRA** |
| --- | --- | --- | --- | --- | --- |
| ASMI | High certainty  (moderate to high certainty evidence) | Category 2: among the most effective | NU | 0.21(0.11,0.32) | 63 |
|  | Low certainty  (low to very low certainty evidence) | Category 2: among the most effective | RT+Nu | 0.47(0.33,0.62) | 98.43 |
|  |  |  | RT | 0.33(0.22,0.45) | 82.17 |
|  |  | Category 0: among the least effective | ART+Nu | 0.15(-0.06,0.36) | 50.18 |
|  |  |  | ART | 0.08(-0.08,0.24) | 35.96 |
|  |  |  | RBT | 0.06(-0.44,0.55) | 37.6 |
|  |  |  | RBT+Nu | 0.05(-0.45,0.55) | 34.16 |
|  |  |  | CT | 0.05(-0.20,0.29) | 30.8 |
| SMI | Low certainty  (low to very low certainty evidence) | Category 2: among the most effective | RBT+Nu | 0.84(0.40,1.29) | 99.84 |
|  |  |  | RBT | 0.50(0.09,0.91) | 83.63 |
|  |  |  | RT+Nu | 0.24(0.17,0.30) | 71.38 |
|  |  | Category 1: intermediately effective | EMS+Nu | 0.09(0.02,0.16) | 40.86 |
|  |  |  | RT | 0.09(0.01,0.17) | 39.53 |
|  |  |  | Nu | 0.05(0.01,0.09) | 25.04 |
|  |  | Category 0: among the least effective | ARBT | 0.14(-0.09,0.37) | 48.83 |
|  |  |  | ART | 0.08(-0.27,0.42) | 34.86 |
| Knee extension strength | High certainty  (moderate to high certainty evidence) | Category 2: among the most effective | RT+Nu | 1.05(0.68,1.42) | 90.38 |
|  | Low certainty  (low to very low certainty evidence) | Category 2: among the most effective | RT | 1.02(0.75,1.30) | 90.34 |
|  |  |  | ARBT | 0.59(0.11,1.08) | 54.52 |
|  |  |  | WBV | 0.56(0.22,0.90) | 51.25 |
|  |  |  | ART | 0.48(0.08,0.89) | 44.18 |
|  |  | Category 1: intermediately effective | Nu | 0.31(0.00,0.62) | 31.42 |
|  |  | Category 0: among the least effective | RBT | 0.39(-0.09,0.88) | 36.84 |
| Balance test | High certainty  (moderate to high certainty evidence) | Category 2: among the most effective | ARBT | 0.73(0.13,1.34) | 84.99 |
|  |  | Category 0: among the least effective | RBT | 0.69(-0.13,1.51) | 78.97 |
|  | Low certainty  (low to very low certainty evidence) | Category 0: among the least effective | RT | 0.34(-0.09,0.77) | 54.67 |
|  |  |  | Nu | 0.27(-0.16,0.70) | 47.72 |
|  |  |  | ART | 0.18(-0.44,0.80) | 38.19 |
|  |  |  | RT+Nu | 0.08(-0.64,0.79) | 29.83 |

#

# Appendix 7: league table

**Table S7.1**: league table of Grip strength

This table presents both direct and network comparisons of intervention effects on Grip strength. All effect estimates are expressed as MD with 95% CI. Results from the network meta-analysis are displayed in the lower triangle, while pairwise meta-analysis results are shown in the upper triangle. Statistically significant results are presented in bold. The shading color indicates the confidence rating of the evidence, as assessed using the CINeMA framework: blue denotes high confidence, green moderate, orange low, and red very low.

**Table S7.1**: league table of Gait speed

This table presents both direct and network comparisons of intervention effects on Gait speed. All effect estimates are expressed as MD with 95% CI. Results from the network meta-analysis are displayed in the lower triangle, while pairwise meta-analysis results are shown in the upper triangle. Statistically significant results are presented in bold. The shading color indicates the confidence rating of the evidence, as assessed using the CINeMA framework: blue denotes high confidence, green moderate, orange low, and red very low.

**Table S7.2**: league table of Knee extension strength

This table presents both direct and network comparisons of intervention effects on Knee extension strength. All effect estimates are expressed as SMD with 95% CI. Results from the network meta-analysis are displayed in the lower triangle, while pairwise meta-analysis results are shown in the upper triangle. Statistically significant results are presented in bold. The shading color indicates the confidence rating of the evidence, as assessed using the CINeMA framework: blue denotes high confidence, green moderate, orange low, and red very low.

**Table S7.3**: league table of Five-Times Sit-to-Stand test

This table presents both direct and network comparisons of intervention effects on Five-Times Sit-to-Stand test. All effect estimates are expressed as MD with 95% CI. Results from the network meta-analysis are displayed in the lower triangle, while pairwise meta-analysis results are shown in the upper triangle. Statistically significant results are presented in bold. The shading color indicates the confidence rating of the evidence, as assessed using the CINeMA framework: blue denotes high confidence, green moderate, orange low, and red very low.

**Table S7.4**: league table of Timed up and go test

This table presents both direct and network comparisons of intervention effects on Timed up and go. All effect estimates are expressed as MD with 95% CI. Results from the network meta-analysis are displayed in the lower triangle, while pairwise meta-analysis results are shown in the upper triangle. Statistically significant results are presented in bold. The shading color indicates the confidence rating of the evidence, as assessed using the CINeMA framework: blue denotes high confidence, green moderate, orange low, and red very low.

**Table S7.8**: league table of SPPB

This table presents both direct and network comparisons of intervention effects on SPPB. All effect estimates are expressed as MD with 95% CI. Results from the network meta-analysis are displayed in the lower triangle, while pairwise meta-analysis results are shown in the upper triangle. Statistically significant results are presented in bold. The shading color indicates the confidence rating of the evidence, as assessed using the CINeMA framework: blue denotes high confidence, green moderate, orange low, and red very low.

**Table S7.5**: league table of Balance test

This table presents both direct and network comparisons of intervention effects on Balance test. All effect estimates are expressed as SMD with 95% CI. Results from the network meta-analysis are displayed in the lower triangle, while pairwise meta-analysis results are shown in the upper triangle. Statistically significant results are presented in bold. The shading color indicates the confidence rating of the evidence, as assessed using the CINeMA framework: blue denotes high confidence, green moderate, orange low, and red very low.

**Table S7.6**: league table of ASMI

This table presents both direct and network comparisons of intervention effects on Balance test. All effect estimates are expressed as MD with 95% CI. Results from the network meta-analysis are displayed in the lower triangle, while pairwise meta-analysis results are shown in the upper triangle. Statistically significant results are presented in bold. The shading color indicates the confidence rating of the evidence, as assessed using the CINeMA framework: blue denotes high confidence, green moderate, orange low, and red very low.

**Table S7.7**: league table of SMI

This table presents both direct and network comparisons of intervention effects on Balance test. All effect estimates are expressed as MD with 95% CI. Results from the network meta-analysis are displayed in the lower triangle, while pairwise meta-analysis results are shown in the upper triangle. Statistically significant results are presented in bold. The shading color indicates the confidence rating of the evidence, as assessed using the CINeMA framework: blue denotes high confidence, green moderate, orange low, and red very low.

# Appendix 8: CINeMA Assessment

We use the CINeMA framework to assess evidence certainty, evaluating each network estimate based on the following criteria:

- **Within study bias:** We classified the overall risk of bias for each study as low risk of bias, the risk of bias as moderate when none of the four assessed risk of bias items were rated as high risk, and the risk of bias as high when one or both items were rated as high risk. See **Appendix 3** for the bias assessment.
- **Reporting bias:** We judged it visually by a funnel plot **(Appendix 9)**.
- **Indirectness:** Transferability assumptions were assessed by reporting baseline glycated hemoglobin levels in the included study population and by comparing age and BMI at baseline concordance between groups.
- **Imprecision:** We use the CINeMA website to grade the accuracy of each comparison.
- **Heterogeneity:** We assessed the degree of worry by comparing clinical reasoning based on 95% confidence intervals (CIs) while applying the same clinical reasoning framework as for inaccuracy. In particular, we judged the consistency of our findings based on the confidence and prediction intervals associated with clinically important effect sizes. And we used the same thresholds of clinical significance as described above and followed the recommendations automatically provided by CINeMA (https://cinema.ispm.unibe.ch/).
- **Inconsistency:** For inconsistency, we looked at the results for node splitting **(Appendix 4)** and we saw major problems when p<0.10, but otherwise no problems.

Table S8.1: Transitivity (Indirectness) Assessment

| Intervention | Baseline variable (Mean ± SD) | |
| --- | --- | --- |
|  | Age | Grip strength |
| ARBT | 74.10±3.51 | 19.87±3.84 |
| ARBT+Nu | 78.80±0.71 | 20.53±0.87 |
| ART | 70.31±8.49 | 19.7±4.43 |
| ART+Nu | 74.04±6.13 | 18.93±2.91 |
| AT | 66.77±7.67 | 16.89±5.27 |
| CT | 74.91±3.27 | - |
| EMS | 77.65±0.49 | - |
| EMS+Nu | 77.15±0.66 | - |
| Nu | 74.22±6.25 | 21.45±6.41 |
| RBT | 80.97±3.14 | 16.99±3.62 |
| RBT+Nu | 80.89±3.87 | 17.73±3.73 |
| RT | 72.10±6.02 | 20.08±4.73 |
| RT+Nu | 73.27±5.89 | 22.45±6.59 |
| TCEs | 72.84±9.65 | 17.10±2.44 |
| WBV | 77.07±6.5 | - |
| CG | 73.12±5.89 | 20.99±5.0 |

**Figure S8.1:** Risk of bias contribution by intervention group in Grip strength

**
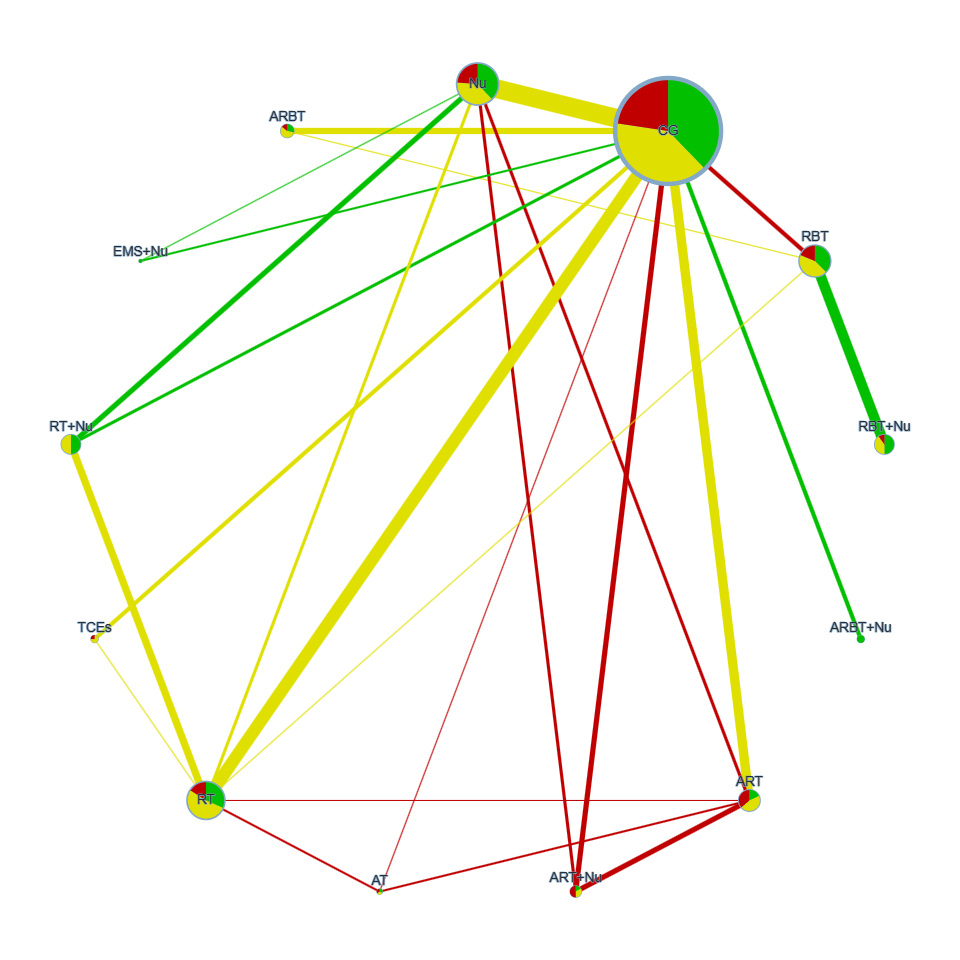
**

**Table S8.2:** CINeMA Results of Grip strength

| Comparison | Within-study  bias | Reporting  bias | Indirectness | Imprecision | Heterogeneity | Incoherence | Confidence rating |
| --- | --- | --- | --- | --- | --- | --- | --- |
| ARBT:CG | Some concerns | Low risk | No concerns | No concerns | Major concerns | No concerns | Very low |
| ARBT:RBT | Some concerns | Low risk | No concerns | Major concerns | No concerns | No concerns | Very low |
| ARBT+Nu:CG | No concerns | Low risk | No concerns | Major concerns | No concerns | No concerns | Low |
| ART:ART+Nu | Major concerns | Low risk | No concerns | Major concerns | No concerns | No concerns | Very low |
| ART:AT | Some concerns | Low risk | No concerns | No concerns | Major concerns | No concerns | Very low |
| ART:CG | Some concerns | Low risk | No concerns | No concerns | Major concerns | Major concerns | Very low |
| ART:Nu | Some concerns | Low risk | No concerns | Major concerns | No concerns | No concerns | Very low |
| ART:RT | Some concerns | Low risk | No concerns | Major concerns | No concerns | No concerns | Very low |
| ART+Nu:CG | Some concerns | Low risk | No concerns | No concerns | Major concerns | No concerns | Very low |
| ART+Nu:Nu | Some concerns | Low risk | No concerns | Major concerns | No concerns | No concerns | Very low |
| AT:CG | Some concerns | Low risk | No concerns | Major concerns | No concerns | No concerns | Very low |
| AT:RT | No concerns | Low risk | No concerns | No concerns | Major concerns | No concerns | Low |
| CG:EMS+Nu | No concerns | Low risk | No concerns | Major concerns | No concerns | No concerns | Low |
| CG:Nu | No concerns | Low risk | No concerns | No concerns | Major concerns | No concerns | Low |
| CG:RBT | Major concerns | Low risk | No concerns | No concerns | Major concerns | No concerns | Very low |
| CG:RT | Some concerns | Low risk | No concerns | No concerns | Major concerns | No concerns | Very low |
| CG:RT+Nu | No concerns | Low risk | No concerns | No concerns | Major concerns | No concerns | Low |
| CG:TCEs | Some concerns | Low risk | No concerns | No concerns | Major concerns | No concerns | Very low |
| EMS+Nu:Nu | No concerns | Low risk | No concerns | Major concerns | No concerns | No concerns | Low |
| Nu:RT | Some concerns | Low risk | No concerns | Major concerns | No concerns | No concerns | Very low |
| Nu:RT+Nu | No concerns | Low risk | No concerns | No concerns | Major concerns | No concerns | Low |
| RBT:RBT+Nu | No concerns | Low risk | No concerns | No concerns | No concerns | No concerns | High |
| RBT:RT | Some concerns | Low risk | No concerns | Major concerns | No concerns | No concerns | Very low |
| RT:RT+Nu | Some concerns | Low risk | No concerns | Major concerns | No concerns | No concerns | Very low |
| RT:TCEs | Some concerns | Low risk | No concerns | Major concerns | No concerns | No concerns | Very low |
| ARBT:ARBT+Nu | No concerns | Low risk | No concerns | Major concerns | No concerns | No concerns | Low |
| ARBT:ART | Some concerns | Low risk | No concerns | Major concerns | No concerns | No concerns | Very low |
| ARBT:ART+Nu | Some concerns | Low risk | No concerns | Major concerns | No concerns | No concerns | Very low |
| ARBT:AT | Some concerns | Low risk | No concerns | Major concerns | No concerns | No concerns | Very low |
| ARBT:EMS+Nu | No concerns | Low risk | No concerns | Major concerns | No concerns | No concerns | Low |
| ARBT:Nu | Some concerns | Low risk | No concerns | Major concerns | No concerns | No concerns | Very low |
| ARBT:RBT+Nu | Some concerns | Low risk | No concerns | No concerns | No concerns | No concerns | Moderate |
| ARBT:RT | Some concerns | Low risk | No concerns | Major concerns | No concerns | No concerns | Very low |
| ARBT:RT+Nu | Some concerns | Low risk | No concerns | Major concerns | No concerns | No concerns | Very low |
| ARBT:TCEs | Some concerns | Low risk | No concerns | Major concerns | No concerns | No concerns | Very low |
| ARBT+Nu:ART | No concerns | Low risk | No concerns | No concerns | Major concerns | No concerns | Low |
| ARBT+Nu:ART+Nu | No concerns | Low risk | No concerns | No concerns | Major concerns | No concerns | Low |
| ARBT+Nu:AT | No concerns | Low risk | No concerns | Major concerns | No concerns | No concerns | Low |
| ARBT+Nu:EMS+Nu | No concerns | Low risk | No concerns | Major concerns | No concerns | No concerns | Low |
| ARBT+Nu:Nu | No concerns | Low risk | No concerns | Major concerns | No concerns | No concerns | Low |
| ARBT+Nu:RBT | No concerns | Low risk | No concerns | Major concerns | No concerns | No concerns | Low |
| ARBT+Nu:RBT+Nu | No concerns | Low risk | No concerns | No concerns | No concerns | No concerns | High |
| ARBT+Nu:RT | No concerns | Low risk | No concerns | Major concerns | No concerns | No concerns | Low |
| ARBT+Nu:RT+Nu | No concerns | Low risk | No concerns | No concerns | Major concerns | No concerns | Low |
| ARBT+Nu:TCEs | No concerns | Low risk | No concerns | Major concerns | No concerns | No concerns | Low |
| ART:EMS+Nu | No concerns | Low risk | No concerns | Major concerns | No concerns | No concerns | Low |
| ART:RBT | Some concerns | Low risk | No concerns | Major concerns | No concerns | No concerns | Very low |
| ART:RBT+Nu | Some concerns | Low risk | No concerns | No concerns | Major concerns | No concerns | Very low |
| ART:RT+Nu | Some concerns | Low risk | No concerns | Major concerns | No concerns | No concerns | Very low |
| ART:TCEs | Some concerns | Low risk | No concerns | Major concerns | No concerns | No concerns | Very low |
| ART+Nu:AT | Some concerns | Low risk | No concerns | No concerns | Major concerns | No concerns | Very low |
| ART+Nu:EMS+Nu | No concerns | Low risk | No concerns | Major concerns | No concerns | No concerns | Low |
| ART+Nu:RBT | Some concerns | Low risk | No concerns | Major concerns | No concerns | No concerns | Very low |
| ART+Nu:RBT+Nu | Some concerns | Low risk | No concerns | No concerns | Major concerns | No concerns | Very low |
| ART+Nu:RT | Some concerns | Low risk | No concerns | Major concerns | No concerns | No concerns | Very low |
| ART+Nu:RT+Nu | Some concerns | Low risk | No concerns | Major concerns | No concerns | No concerns | Very low |
| ART+Nu:TCEs | Some concerns | Low risk | No concerns | Major concerns | No concerns | No concerns | Very low |
| AT:EMS+Nu | No concerns | Low risk | No concerns | Major concerns | No concerns | No concerns | Low |
| AT:Nu | Some concerns | Low risk | No concerns | Major concerns | No concerns | No concerns | Very low |
| AT:RBT | Some concerns | Low risk | No concerns | Major concerns | No concerns | No concerns | Very low |
| AT:RBT+Nu | No concerns | Low risk | No concerns | No concerns | No concerns | No concerns | High |
| AT:RT+Nu | Some concerns | Low risk | No concerns | No concerns | Major concerns | No concerns | Very low |
| AT:TCEs | Some concerns | Low risk | No concerns | Major concerns | No concerns | No concerns | Very low |
| CG:RBT+Nu | No concerns | Low risk | No concerns | No concerns | No concerns | No concerns | High |
| EMS+Nu:RBT | No concerns | Low risk | No concerns | Major concerns | No concerns | No concerns | Low |
| EMS+Nu:RBT+Nu | No concerns | Low risk | No concerns | No concerns | Major concerns | No concerns | Low |
| EMS+Nu:RT | No concerns | Low risk | No concerns | Major concerns | No concerns | No concerns | Low |
| EMS+Nu:RT+Nu | No concerns | Low risk | No concerns | Major concerns | No concerns | No concerns | Low |
| EMS+Nu:TCEs | No concerns | Low risk | No concerns | Major concerns | No concerns | No concerns | Low |
| Nu:RBT | Some concerns | Low risk | No concerns | Major concerns | No concerns | No concerns | Very low |
| Nu:RBT+Nu | No concerns | Low risk | No concerns | No concerns | No concerns | No concerns | High |
| Nu:TCEs | Some concerns | Low risk | No concerns | Major concerns | No concerns | No concerns | Very low |
| RBT:RT+Nu | Some concerns | Low risk | No concerns | Major concerns | No concerns | No concerns | Very low |
| RBT:TCEs | Some concerns | Low risk | No concerns | Major concerns | No concerns | No concerns | Very low |
| RBT+Nu:RT | Some concerns | Low risk | No concerns | No concerns | No concerns | No concerns | Moderate |
| RBT+Nu:RT+Nu | No concerns | Low risk | No concerns | No concerns | Major concerns | No concerns | Low |
| RBT+Nu:TCEs | Some concerns | Low risk | No concerns | No concerns | Major concerns | No concerns | Very low |
| RT+Nu:TCEs | Some concerns | Low risk | No concerns | Major concerns | No concerns | No concerns | Very low |

**Figure S8.2**: Risk of bias contribution by intervention group in Knee extension strength


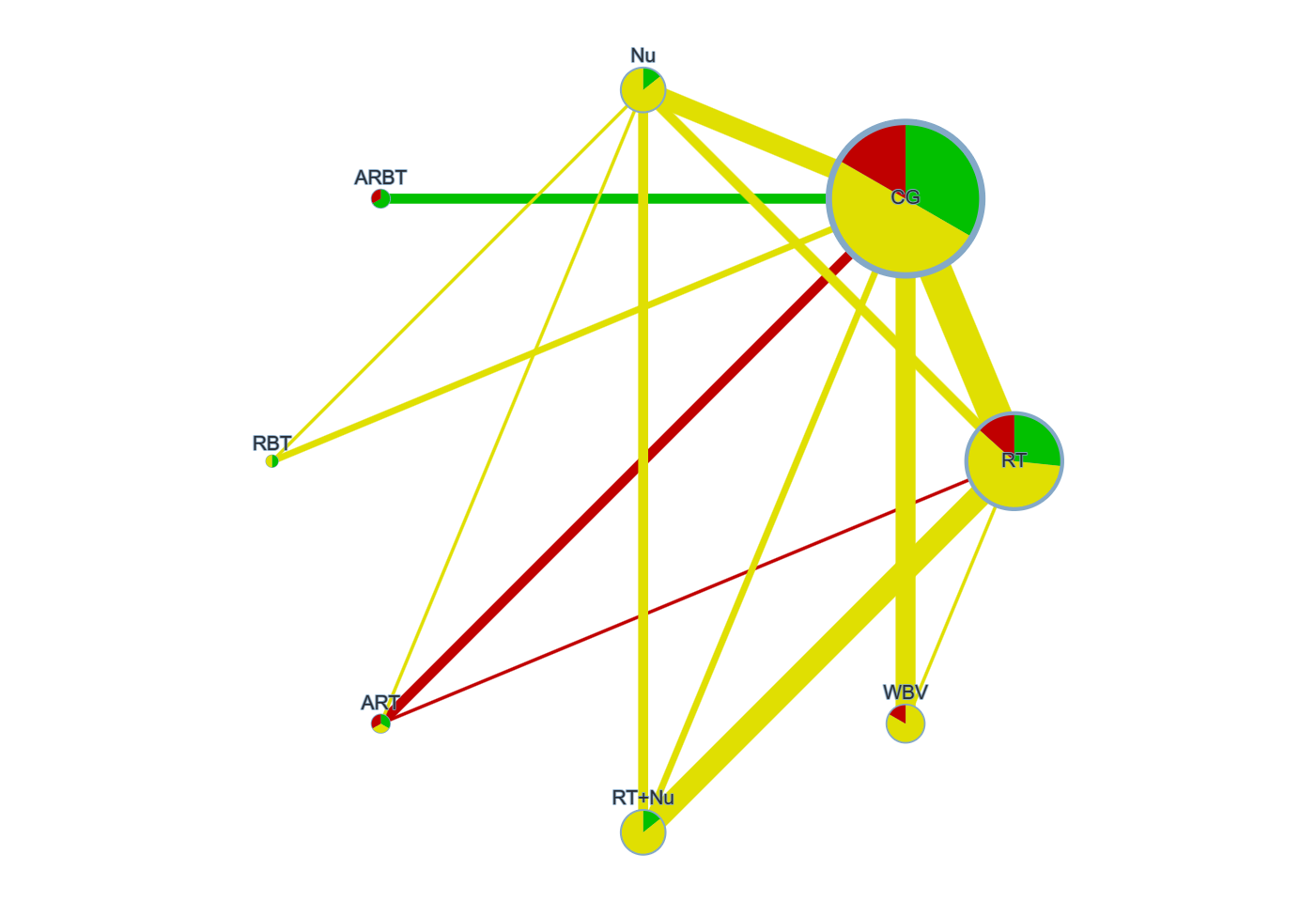


**Table S8.3:** CINeMA Results of Knee extension strength

| Comparison | Within-study  bias | Reporting  bias | Indirectness | Imprecision | Heterogeneity | Incoherence | Confidence  rating |
| --- | --- | --- | --- | --- | --- | --- | --- |
| ARBT:CG | No concerns | Low risk | No concerns | No concerns | Major concerns | Some concerns | Very low |
| ART:CG | Some concerns | Low risk | No concerns | No concerns | Major concerns | No concerns | Very low |
| ART:Nu | Some concerns | Low risk | No concerns | Major concerns | No concerns | No concerns | Low |
| ART:RT | Some concerns | Low risk | No concerns | No concerns | Major concerns | No concerns | Very low |
| CG:Nu | Some concerns | Low risk | No concerns | No concerns | Major concerns | Major concerns | Very low |
| CG:RBT | Some concerns | Low risk | No concerns | Major concerns | No concerns | No concerns | Very low |
| CG:RT | No concerns | Low risk | No concerns | No concerns | No concerns | Major concerns | Low |
| CG:RT+Nu | Some concerns | Low risk | No concerns | No concerns | No concerns | No concerns | Moderate |
| CG:WBV | Some concerns | Low risk | No concerns | No concerns | No concerns | No concerns | Moderate |
| Nu:RBT | Some concerns | Low risk | No concerns | Major concerns | No concerns | No concerns | Very low |
| Nu:RT | Some concerns | Low risk | No concerns | No concerns | Major concerns | No concerns | Very low |
| Nu:RT+Nu | Some concerns | Low risk | No concerns | No concerns | Major concerns | Major concerns | Very low |
| RT:RT+Nu | Some concerns | Low risk | No concerns | Major concerns | No concerns | Major concerns | Very low |
| RT:WBV | Some concerns | Low risk | No concerns | Major concerns | No concerns | No concerns | Very low |
| ARBT:ART | No concerns | Low risk | No concerns | Major concerns | No concerns | Some concerns | Very low |
| ARBT:Nu | No concerns | Low risk | No concerns | Major concerns | No concerns | Some concerns | Very low |
| ARBT:RBT | No concerns | Low risk | No concerns | Major concerns | No concerns | Some concerns | Very low |
| ARBT:RT | No concerns | Low risk | No concerns | Major concerns | No concerns | Some concerns | Very low |
| ARBT:RT+Nu | No concerns | Low risk | No concerns | Major concerns | No concerns | Some concerns | Very low |
| ARBT:WBV | Some concerns | Low risk | No concerns | Major concerns | No concerns | Some concerns | Very low |
| ART:RBT | Some concerns | Low risk | No concerns | Major concerns | No concerns | Some concerns | Very low |
| ART:RT+Nu | Some concerns | Low risk | No concerns | Major concerns | No concerns | Some concerns | Very low |
| ART:WBV | Some concerns | Low risk | No concerns | Major concerns | No concerns | Some concerns | Very low |
| Nu:WBV | Some concerns | Low risk | No concerns | Major concerns | No concerns | Some concerns | Very low |
| RBT:RT | Some concerns | Low risk | No concerns | No concerns | Major concerns | Some concerns | Very low |
| RBT:RT+Nu | Some concerns | Low risk | No concerns | Major concerns | No concerns | Some concerns | Very low |
| RBT:WBV | Some concerns | Low risk | No concerns | Major concerns | No concerns | Some concerns | Very low |
| RT+Nu:WBV | Some concerns | Low risk | No concerns | Major concerns | No concerns | Some concerns | Very low |

**Figure S8.3**: Risk of bias contribution by intervention group in Gait speed


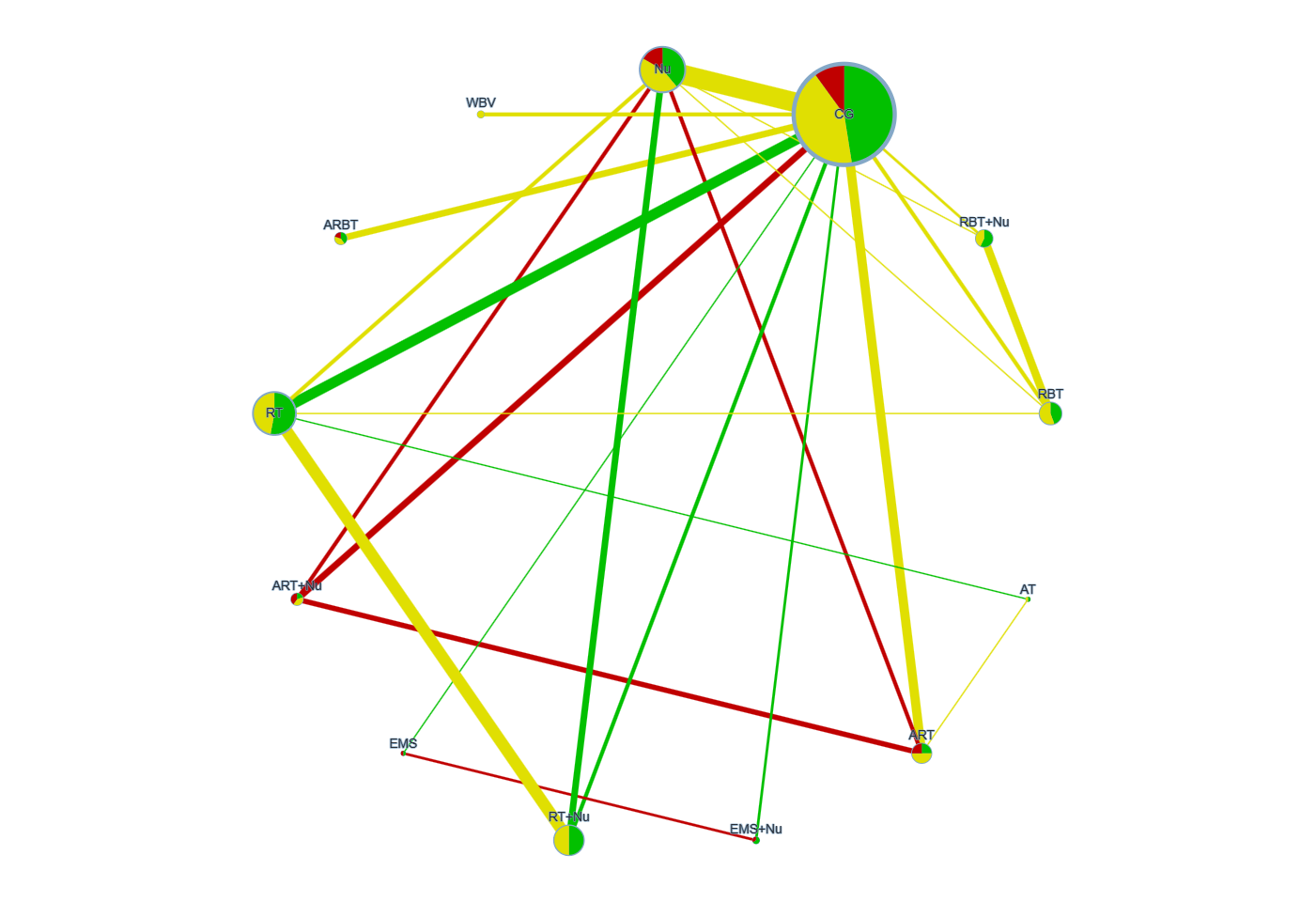


**Table S8.4:** CINeMA Results of Gait speed

| Comparison | Within-study  bias | Reporting  bias | Indirectness | Imprecision | Heterogeneity | Incoherence | Confidence  rating |
| --- | --- | --- | --- | --- | --- | --- | --- |
| ARBT:CG | No concerns | Low risk | No concerns | No concerns | Major concerns | No concerns | Low |
| ART:ART+Nu | Major concerns | Low risk | No concerns | Major concerns | No concerns | No concerns | Very low |
| ART:AT | Some concerns | Low risk | No concerns | Major concerns | No concerns | No concerns | Very low |
| ART:CG | Major concerns | Low risk | No concerns | No concerns | Major concerns | No concerns | Very low |
| ART:Nu | Major concerns | Low risk | No concerns | Major concerns | No concerns | No concerns | Very low |
| ART+Nu:CG | Some concerns | Low risk | No concerns | Major concerns | No concerns | No concerns | Very low |
| ART+Nu:Nu | Major concerns | Low risk | No concerns | Major concerns | No concerns | No concerns | Very low |
| AT:RT | No concerns | Low risk | No concerns | Major concerns | No concerns | No concerns | Low |
| CG:EMS | No concerns | Low risk | No concerns | Major concerns | No concerns | No concerns | Low |
| CG:EMS+Nu | No concerns | Low risk | No concerns | Major concerns | No concerns | No concerns | Low |
| CG:Nu | Some concerns | Low risk | No concerns | No concerns | Major concerns | No concerns | Very low |
| CG:RBT | Some concerns | Low risk | No concerns | No concerns | Major concerns | No concerns | Very low |
| CG:RBT+Nu | No concerns | Low risk | No concerns | No concerns | No concerns | No concerns | High |
| CG:RT | No concerns | Low risk | No concerns | No concerns | Major concerns | No concerns | Low |
| CG:RT+Nu | No concerns | Low risk | No concerns | No concerns | Major concerns | No concerns | Low |
| CG:WBV | Some concerns | Low risk | No concerns | No concerns | Major concerns | No concerns | Very low |
| EMS:EMS+Nu | No concerns | Low risk | No concerns | Major concerns | No concerns | No concerns | Low |
| Nu:RBT | Some concerns | Low risk | No concerns | Major concerns | No concerns | No concerns | Very low |
| Nu:RBT+Nu | Some concerns | Low risk | No concerns | No concerns | Major concerns | No concerns | Very low |
| Nu:RT | No concerns | Low risk | No concerns | Major concerns | No concerns | No concerns | Low |
| Nu:RT+Nu | No concerns | Low risk | No concerns | No concerns | Major concerns | No concerns | Low |
| RBT:RBT+Nu | No concerns | Low risk | No concerns | No concerns | Major concerns | No concerns | Low |
| RBT:RT | Some concerns | Low risk | No concerns | Major concerns | No concerns | No concerns | Very low |
| RT:RT+Nu | No concerns | Low risk | No concerns | No concerns | Major concerns | No concerns | Low |
| ARBT:ART | Some concerns | Low risk | No concerns | Major concerns | No concerns | No concerns | Very low |
| ARBT:ART+Nu | Some concerns | Low risk | No concerns | Major concerns | No concerns | No concerns | Very low |
| ARBT:AT | Some concerns | Low risk | No concerns | Major concerns | No concerns | No concerns | Very low |
| ARBT:EMS | No concerns | Low risk | No concerns | Major concerns | No concerns | No concerns | Low |
| ARBT:EMS+Nu | No concerns | Low risk | No concerns | Major concerns | No concerns | No concerns | Low |
| ARBT:Nu | No concerns | Low risk | No concerns | Major concerns | No concerns | No concerns | Low |
| ARBT:RBT | Some concerns | Low risk | No concerns | Major concerns | No concerns | No concerns | Very low |
| ARBT:RBT+Nu | Some concerns | Low risk | No concerns | Major concerns | No concerns | No concerns | Very low |
| ARBT:RT | No concerns | Low risk | No concerns | Major concerns | No concerns | No concerns | Low |
| ARBT:RT+Nu | No concerns | Low risk | No concerns | Major concerns | No concerns | No concerns | Low |
| ARBT:WBV | Some concerns | Low risk | No concerns | Major concerns | No concerns | No concerns | Very low |
| ART:EMS | No concerns | Low risk | No concerns | Major concerns | No concerns | No concerns | Low |
| ART:EMS+Nu | No concerns | Low risk | No concerns | Major concerns | No concerns | No concerns | Low |
| ART:RBT | Some concerns | Low risk | No concerns | Major concerns | No concerns | No concerns | Very low |
| ART:RBT+Nu | Some concerns | Low risk | No concerns | No concerns | Major concerns | No concerns | Very low |
| ART:RT | No concerns | Low risk | No concerns | Major concerns | No concerns | No concerns | Low |
| ART:RT+Nu | No concerns | Low risk | No concerns | Major concerns | No concerns | No concerns | Low |
| ART:WBV | Some concerns | Low risk | No concerns | Major concerns | No concerns | No concerns | Very low |
| ART+Nu:AT | Some concerns | Low risk | No concerns | Major concerns | No concerns | No concerns | Very low |
| ART+Nu:EMS | No concerns | Low risk | No concerns | Major concerns | No concerns | No concerns | Low |
| ART+Nu:EMS+Nu | No concerns | Low risk | No concerns | Major concerns | No concerns | No concerns | Low |
| ART+Nu:RBT | Some concerns | Low risk | No concerns | Major concerns | No concerns | No concerns | Very low |
| ART+Nu:RBT+Nu | Some concerns | Low risk | No concerns | No concerns | Major concerns | No concerns | Very low |
| ART+Nu:RT | No concerns | Low risk | No concerns | Major concerns | No concerns | No concerns | Low |
| ART+Nu:RT+Nu | No concerns | Low risk | No concerns | Major concerns | No concerns | No concerns | Low |
| ART+Nu:WBV | Some concerns | Low risk | No concerns | Major concerns | No concerns | No concerns | Very low |
| AT:CG | Some concerns | Low risk | No concerns | Major concerns | No concerns | No concerns | Very low |
| AT:EMS | No concerns | Low risk | No concerns | Major concerns | No concerns | No concerns | Low |
| AT:EMS+Nu | No concerns | Low risk | No concerns | Major concerns | No concerns | No concerns | Low |
| AT:Nu | Some concerns | Low risk | No concerns | Major concerns | No concerns | No concerns | Very low |
| AT:RBT | Some concerns | Low risk | No concerns | Major concerns | No concerns | No concerns | Very low |
| AT:RBT+Nu | Some concerns | Low risk | No concerns | Major concerns | No concerns | No concerns | Very low |
| AT:RT+Nu | No concerns | Low risk | No concerns | Major concerns | No concerns | No concerns | Low |
| AT:WBV | Some concerns | Low risk | No concerns | Major concerns | No concerns | No concerns | Very low |
| EMS:Nu | No concerns | Low risk | No concerns | Major concerns | No concerns | No concerns | Low |
| EMS:RBT | No concerns | Low risk | No concerns | Major concerns | No concerns | No concerns | Low |
| EMS:RBT+Nu | No concerns | Low risk | No concerns | Major concerns | No concerns | No concerns | Low |
| EMS:RT | No concerns | Low risk | No concerns | Major concerns | No concerns | No concerns | Low |
| EMS:RT+Nu | No concerns | Low risk | No concerns | Major concerns | No concerns | No concerns | Low |
| EMS:WBV | No concerns | Low risk | No concerns | Major concerns | No concerns | No concerns | Low |
| EMS+Nu:Nu | No concerns | Low risk | No concerns | Major concerns | No concerns | No concerns | Low |
| EMS+Nu:RBT | No concerns | Low risk | No concerns | Major concerns | No concerns | No concerns | Low |
| EMS+Nu:RBT+Nu | No concerns | Low risk | No concerns | No concerns | Major concerns | No concerns | Low |
| EMS+Nu:RT | No concerns | Low risk | No concerns | Major concerns | No concerns | No concerns | Low |
| EMS+Nu:RT+Nu | No concerns | Low risk | No concerns | Major concerns | No concerns | No concerns | Low |
| EMS+Nu:WBV | No concerns | Low risk | No concerns | Major concerns | No concerns | No concerns | Low |
| Nu:WBV | Some concerns | Low risk | No concerns | Major concerns | No concerns | No concerns | Very low |
| RBT:RT+Nu | Some concerns | Low risk | No concerns | Major concerns | No concerns | No concerns | Very low |
| RBT:WBV | Some concerns | Low risk | No concerns | Major concerns | No concerns | No concerns | Very low |
| RBT+Nu:RT | No concerns | Low risk | No concerns | No concerns | Major concerns | No concerns | Low |
| RBT+Nu:RT+Nu | No concerns | Low risk | No concerns | Major concerns | No concerns | No concerns | Low |
| RBT+Nu:WBV | Some concerns | Low risk | No concerns | Major concerns | No concerns | No concerns | Very low |
| RT:WBV | Some concerns | Low risk | No concerns | Major concerns | No concerns | No concerns | Very low |
| RT+Nu:WBV | Some concerns | Low risk | No concerns | Major concerns | No concerns | No concerns | Very low |

**Figure S8.4:** Risk of bias contribution by intervention group in Five-Times Sit-to-Stand test


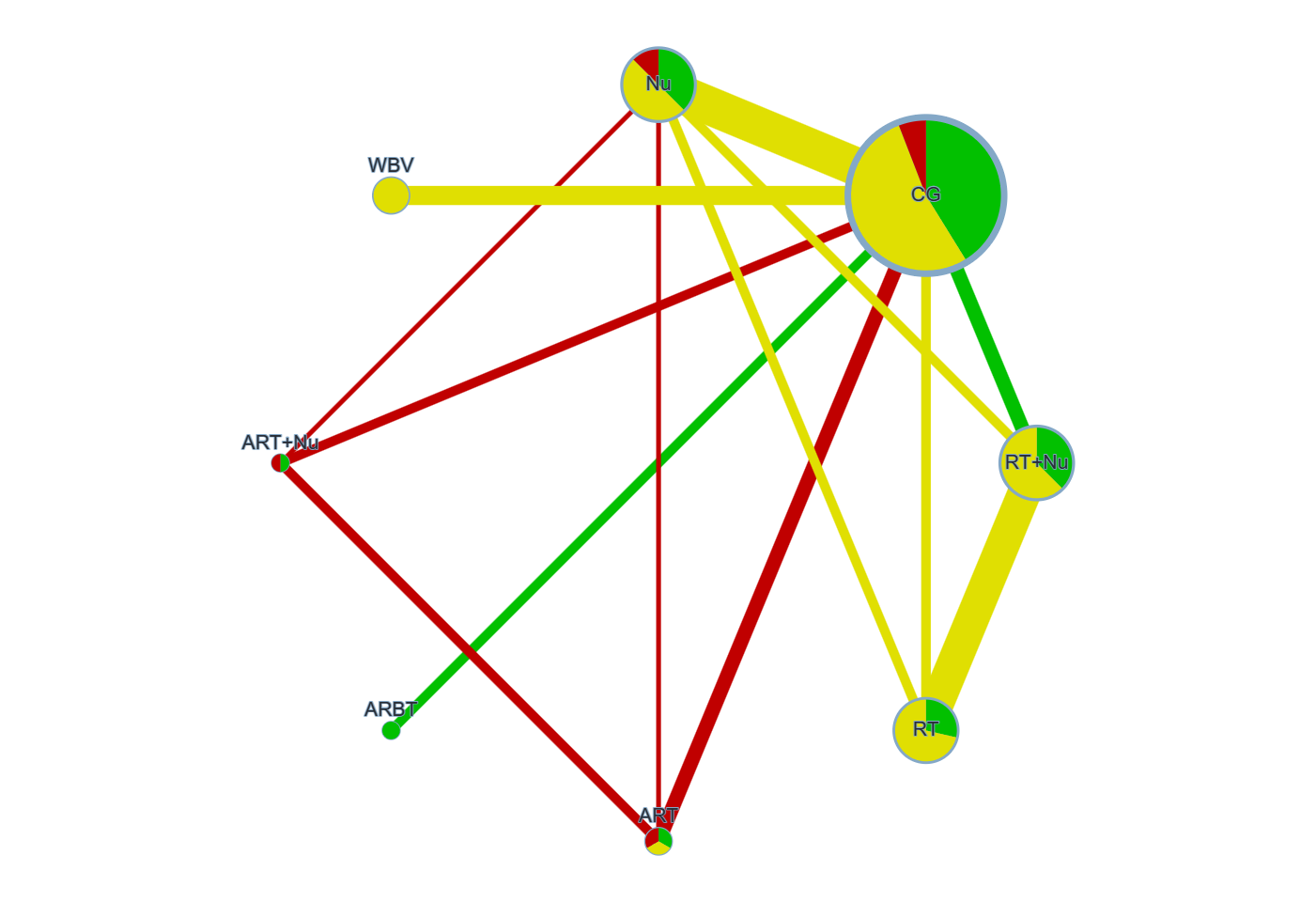


**Table S8.5:** CINeMA Results of Five-Times Sit-to-Stand test

| Comparison | Within-study  bias | Reporting  bias | Indirectness | Imprecision | Heterogeneity | Incoherence | Confidence  rating |
| --- | --- | --- | --- | --- | --- | --- | --- |
| ARBT:CG | No concerns | Low risk | No concerns | Major concerns | No concerns | No concerns | Low |
| ART:ART+Nu | Major concerns | Low risk | No concerns | Major concerns | No concerns | No concerns | Very low |
| ART:CG | Major concerns | Low risk | No concerns | No concerns | Major concerns | No concerns | Very low |
| ART:Nu | Major concerns | Low risk | No concerns | Major concerns | No concerns | No concerns | Very low |
| ART+Nu:CG | Major concerns | Low risk | No concerns | No concerns | No concerns | No concerns | Low |
| ART+Nu:Nu | Major concerns | Low risk | No concerns | No concerns | Major concerns | No concerns | Very low |
| CG:Nu | No concerns | Low risk | No concerns | Major concerns | No concerns | No concerns | Low |
| CG:RT | No concerns | Low risk | No concerns | No concerns | Major concerns | No concerns | Low |
| CG:RT+Nu | No concerns | Low risk | No concerns | No concerns | Major concerns | No concerns | Low |
| CG:WBV | Some concerns | Low risk | No concerns | No concerns | Major concerns | No concerns | Very low |
| Nu:RT | No concerns | Low risk | No concerns | Major concerns | No concerns | No concerns | Low |
| Nu:RT+Nu | No concerns | Low risk | No concerns | No concerns | Major concerns | No concerns | Low |
| RT:RT+Nu | No concerns | Low risk | No concerns | Major concerns | No concerns | No concerns | Low |
| ARBT:ART | No concerns | Low risk | No concerns | Major concerns | No concerns | No concerns | Low |
| ARBT:ART+Nu | No concerns | Low risk | No concerns | Major concerns | No concerns | No concerns | Low |
| ARBT:Nu | No concerns | Low risk | No concerns | Major concerns | No concerns | No concerns | Low |
| ARBT:RT | No concerns | Low risk | No concerns | Major concerns | No concerns | No concerns | Low |
| ARBT:RT+Nu | No concerns | Low risk | No concerns | Major concerns | No concerns | No concerns | Low |
| ARBT:WBV | No concerns | Low risk | No concerns | Major concerns | No concerns | No concerns | Low |
| ART:RT | No concerns | Low risk | No concerns | Major concerns | No concerns | No concerns | Low |
| ART:RT+Nu | No concerns | Low risk | No concerns | Major concerns | No concerns | No concerns | Low |
| ART:WBV | Some concerns | Low risk | No concerns | Major concerns | No concerns | No concerns | Very low |
| ART+Nu:RT | No concerns | Low risk | No concerns | Major concerns | No concerns | No concerns | Low |
| ART+Nu:RT+Nu | No concerns | Low risk | No concerns | Major concerns | No concerns | No concerns | Low |
| ART+Nu:WBV | Some concerns | Low risk | No concerns | Major concerns | No concerns | No concerns | Very low |
| Nu:WBV | Some concerns | Low risk | No concerns | Major concerns | No concerns | No concerns | Very low |
| RT:WBV | Some concerns | Low risk | No concerns | Major concerns | No concerns | No concerns | Very low |
| RT+Nu:WBV | Some concerns | Low risk | No concerns | Major concerns | No concerns | No concerns | Very low |

**Figure S8.5:** Risk of bias contribution by intervention group in TUG


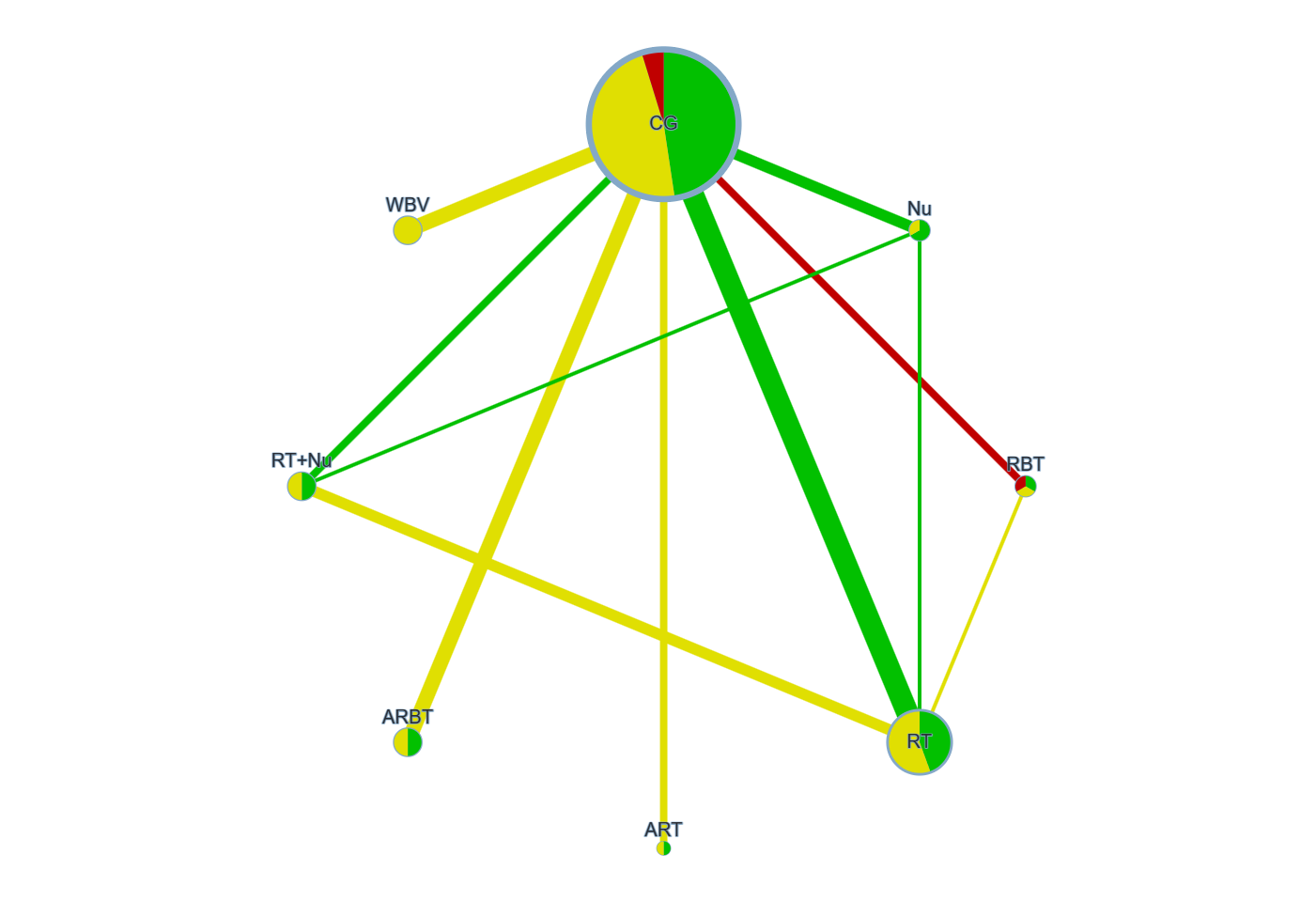


**Table S8.6:** CINeMA Results of TUG

| Comparison | Within-study  bias | Reporting  bias | Indirectness | Imprecision | Heterogeneity | Incoherence | Confidence  rating |
| --- | --- | --- | --- | --- | --- | --- | --- |
| ARBT:CG | No concerns | Low risk | No concerns | No concerns | No concerns | No concerns | High |
| ART:CG | No concerns | Low risk | No concerns | No concerns | No concerns | No concerns | High |
| CG:Nu | No concerns | Low risk | No concerns | Major concerns | No concerns | No concerns | Low |
| CG:RBT | No concerns | Low risk | No concerns | No concerns | No concerns | No concerns | High |
| CG:RT | No concerns | Low risk | No concerns | No concerns | Major concerns | No concerns | Low |
| CG:RT+Nu | No concerns | Low risk | No concerns | No concerns | Major concerns | No concerns | Low |
| CG:WBV | Some concerns | Low risk | No concerns | No concerns | No concerns | No concerns | Moderate |
| Nu:RT | No concerns | Low risk | No concerns | Major concerns | No concerns | No concerns | Low |
| Nu:RT+Nu | No concerns | Low risk | No concerns | No concerns | Major concerns | No concerns | Low |
| RBT:RT | No concerns | Low risk | No concerns | No concerns | No concerns | No concerns | High |
| RT:RT+Nu | No concerns | Low risk | No concerns | Major concerns | No concerns | No concerns | Low |
| ARBT:ART | No concerns | Low risk | No concerns | Major concerns | No concerns | No concerns | Low |
| ARBT:Nu | No concerns | Low risk | No concerns | No concerns | Major concerns | No concerns | Low |
| ARBT:RBT | No concerns | Low risk | No concerns | Major concerns | No concerns | No concerns | Low |
| ARBT:RT | No concerns | Low risk | No concerns | Major concerns | No concerns | No concerns | Low |
| ARBT:RT+Nu | No concerns | Low risk | No concerns | Major concerns | No concerns | No concerns | Low |
| ARBT:WBV | Some concerns | Low risk | No concerns | Major concerns | No concerns | No concerns | Very low |
| ART:Nu | No concerns | Low risk | No concerns | No concerns | Major concerns | No concerns | Low |
| ART:RBT | No concerns | Low risk | No concerns | Major concerns | No concerns | No concerns | Low |
| ART:RT | No concerns | Low risk | No concerns | Major concerns | No concerns | No concerns | Low |
| ART:RT+Nu | No concerns | Low risk | No concerns | Major concerns | No concerns | No concerns | Low |
| ART:WBV | Some concerns | Low risk | No concerns | Major concerns | No concerns | No concerns | Very low |
| Nu:RBT | No concerns | Low risk | No concerns | No concerns | No concerns | No concerns | High |
| Nu:WBV | Some concerns | Low risk | No concerns | No concerns | No concerns | No concerns | Moderate |
| RBT:RT+Nu | No concerns | Low risk | No concerns | No concerns | Major concerns | No concerns | Low |
| RBT:WBV | Some concerns | Low risk | No concerns | Major concerns | No concerns | No concerns | Very low |
| RT:WBV | Some concerns | Low risk | No concerns | No concerns | Major concerns | No concerns | Very low |
| RT+Nu:WBV | Some concerns | Low risk | No concerns | Major concerns | No concerns | No concerns | Very low |

**Figure S8.6:** Risk of bias contribution by intervention group in SPPB


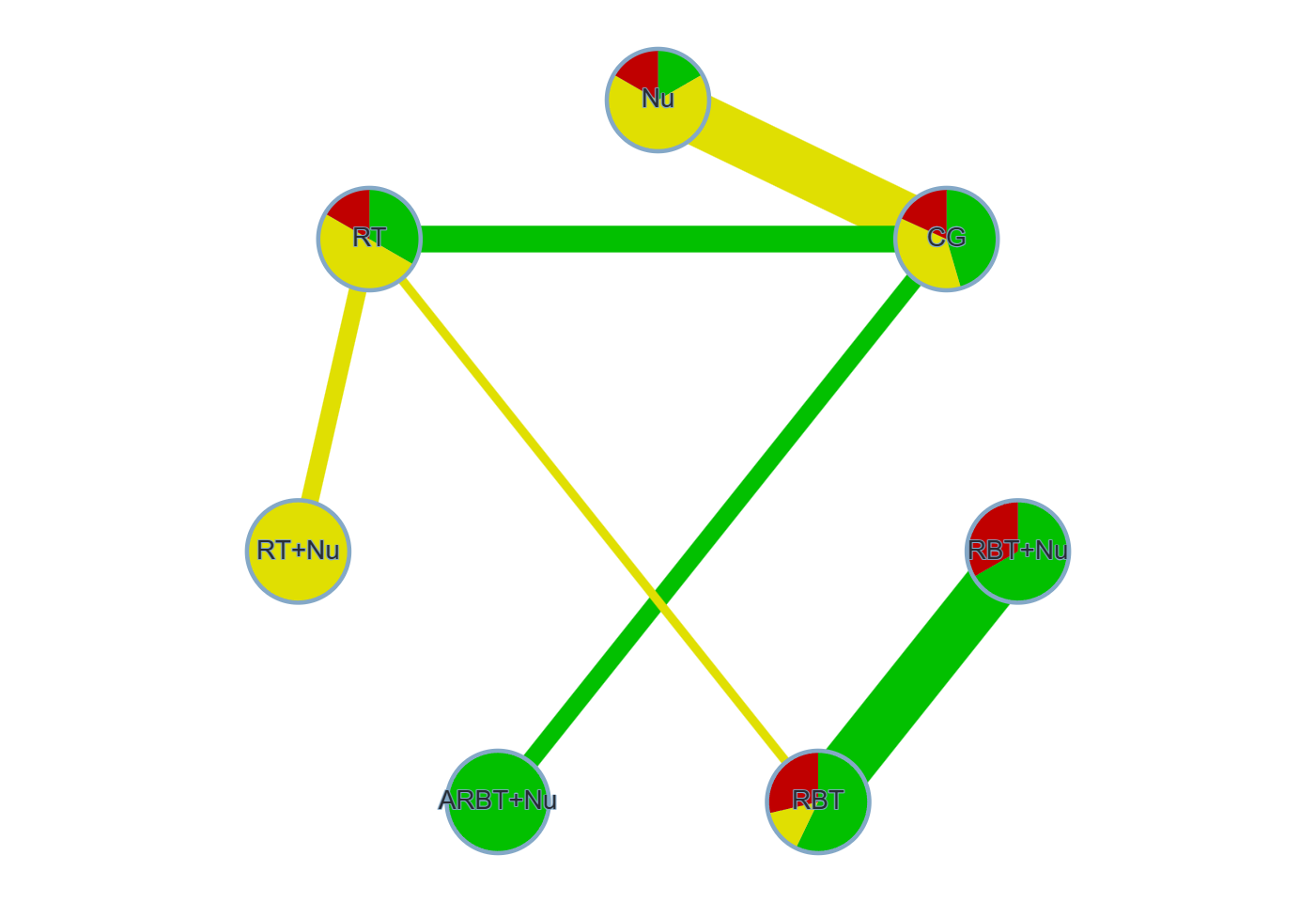


**Table S8.7:** CINeMA Results of SPPB

| Comparison | Within-study  bias | Reporting  bias | Indirectness | Imprecision | Heterogeneity | Incoherence | Confidence  rating |
| --- | --- | --- | --- | --- | --- | --- | --- |
| ARBT+Nu:CG | No concerns | Low risk | No concerns | Major concerns | No concerns | Major concerns | Very low |
| CG:Nu | Some concerns | Low risk | No concerns | Major concerns | No concerns | Major concerns | Very low |
| CG:RT | No concerns | Low risk | No concerns | Major concerns | No concerns | Major concerns | Very low |
| RBT:RBT+Nu | No concerns | Low risk | No concerns | No concerns | No concerns | Major concerns | Low |
| RBT:RT | Some concerns | Low risk | No concerns | No concerns | Major concerns | Major concerns | Very low |
| RT:RT+Nu | Some concerns | Low risk | No concerns | Major concerns | No concerns | Major concerns | Very low |
| ARBT+Nu:Nu | No concerns | Low risk | No concerns | Major concerns | No concerns | Major concerns | Very low |
| ARBT+Nu:RBT | No concerns | Low risk | No concerns | Major concerns | No concerns | Major concerns | Very low |
| ARBT+Nu:RBT+Nu | No concerns | Low risk | No concerns | No concerns | No concerns | Major concerns | Low |
| ARBT+Nu:RT | No concerns | Low risk | No concerns | Major concerns | No concerns | Major concerns | Very low |
| ARBT+Nu:RT+Nu | No concerns | Low risk | No concerns | Major concerns | No concerns | Major concerns | Very low |
| CG:RBT | Some concerns | Low risk | No concerns | No concerns | Major concerns | Major concerns | Very low |
| CG:RBT+Nu | No concerns | Low risk | No concerns | No concerns | No concerns | Major concerns | Low |
| CG:RT+Nu | Some concerns | Low risk | No concerns | Major concerns | No concerns | Major concerns | Very low |
| Nu:RBT | Some concerns | Low risk | No concerns | No concerns | Major concerns | Major concerns | Very low |
| Nu:RBT+Nu | Some concerns | Low risk | No concerns | No concerns | No concerns | Major concerns | Very low |
| Nu:RT | No concerns | Low risk | No concerns | Major concerns | No concerns | Major concerns | Very low |
| Nu:RT+Nu | Some concerns | Low risk | No concerns | Major concerns | No concerns | Major concerns | Very low |
| RBT:RT+Nu | Some concerns | Low risk | No concerns | Major concerns | No concerns | Major concerns | Very low |
| RBT+Nu:RT | Some concerns | Low risk | No concerns | No concerns | No concerns | Major concerns | Very low |
| RBT+Nu:RT+Nu | Some concerns | Low risk | No concerns | No concerns | No concerns | Major concerns | Very low |

**Figure S8.7:** Risk of bias contribution by intervention group in Balance test


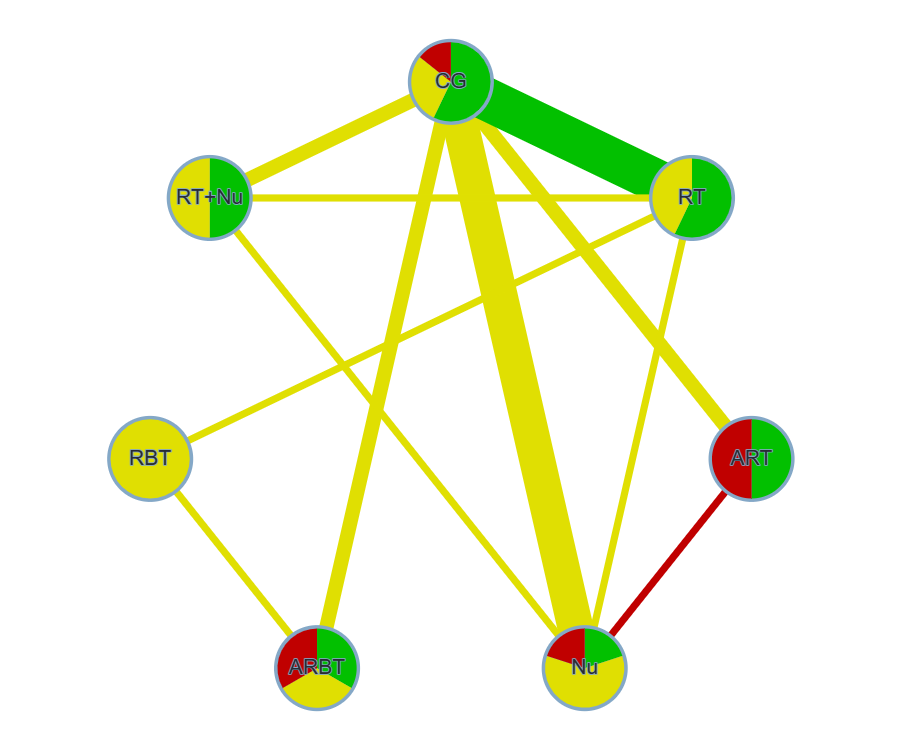


**Table S8.8:** CINeMA Results of Balance test

| Comparison | Within-study  bias | Reporting bias | Indirectness | Imprecision | Heterogeneity | Incoherence | Confidence rating |
| --- | --- | --- | --- | --- | --- | --- | --- |
| ARBT:CG | No concerns | Low risk | No concerns | No concerns | No concerns | No concerns | High |
| ARBT:RBT | Some concerns | Low risk | No concerns | Major concerns | No concerns | Major concerns | Very low |
| ART:CG | Major concerns | Low risk | No concerns | Major concerns | No concerns | No concerns | Very low |
| ART:Nu | Major concerns | Low risk | No concerns | Major concerns | No concerns | No concerns | Very low |
| CG:Nu | Major concerns | Low risk | No concerns | Major concerns | No concerns | No concerns | Very low |
| CG:RT | No concerns | Low risk | No concerns | Major concerns | No concerns | Major concerns | Very low |
| CG:RT+Nu | No concerns | Low risk | No concerns | Major concerns | No concerns | No concerns | Low |
| Nu:RT | No concerns | Low risk | No concerns | Major concerns | No concerns | No concerns | Low |
| Nu:RT+Nu | No concerns | Low risk | No concerns | Major concerns | No concerns | No concerns | Low |
| RBT:RT | Some concerns | Low risk | No concerns | No concerns | No concerns | Major concerns | Very low |
| RT:RT+Nu | No concerns | Low risk | No concerns | Major concerns | No concerns | No concerns | Low |
| ARBT:ART | No concerns | Low risk | No concerns | No concerns | No concerns | No concerns | High |
| ARBT:Nu | No concerns | Low risk | No concerns | No concerns | No concerns | No concerns | High |
| ARBT:RT | No concerns | Low risk | No concerns | No concerns | No concerns | No concerns | High |
| ARBT:RT+Nu | No concerns | Low risk | No concerns | No concerns | No concerns | No concerns | High |
| ART:RBT | No concerns | Low risk | No concerns | No concerns | No concerns | No concerns | High |
| ART:RT | No concerns | Low risk | No concerns | Major concerns | No concerns | No concerns | Low |
| ART:RT+Nu | No concerns | Low risk | No concerns | Major concerns | No concerns | No concerns | Low |
| CG:RBT | Some concerns | Low risk | No concerns | No concerns | No concerns | No concerns | Moderate |
| Nu:RBT | Some concerns | Low risk | No concerns | No concerns | No concerns | No concerns | Moderate |
| RBT:RT+Nu | No concerns | Low risk | No concerns | No concerns | No concerns | No concerns | High |

**Figure S8.8:** Risk of bias contribution by intervention group in ASMI


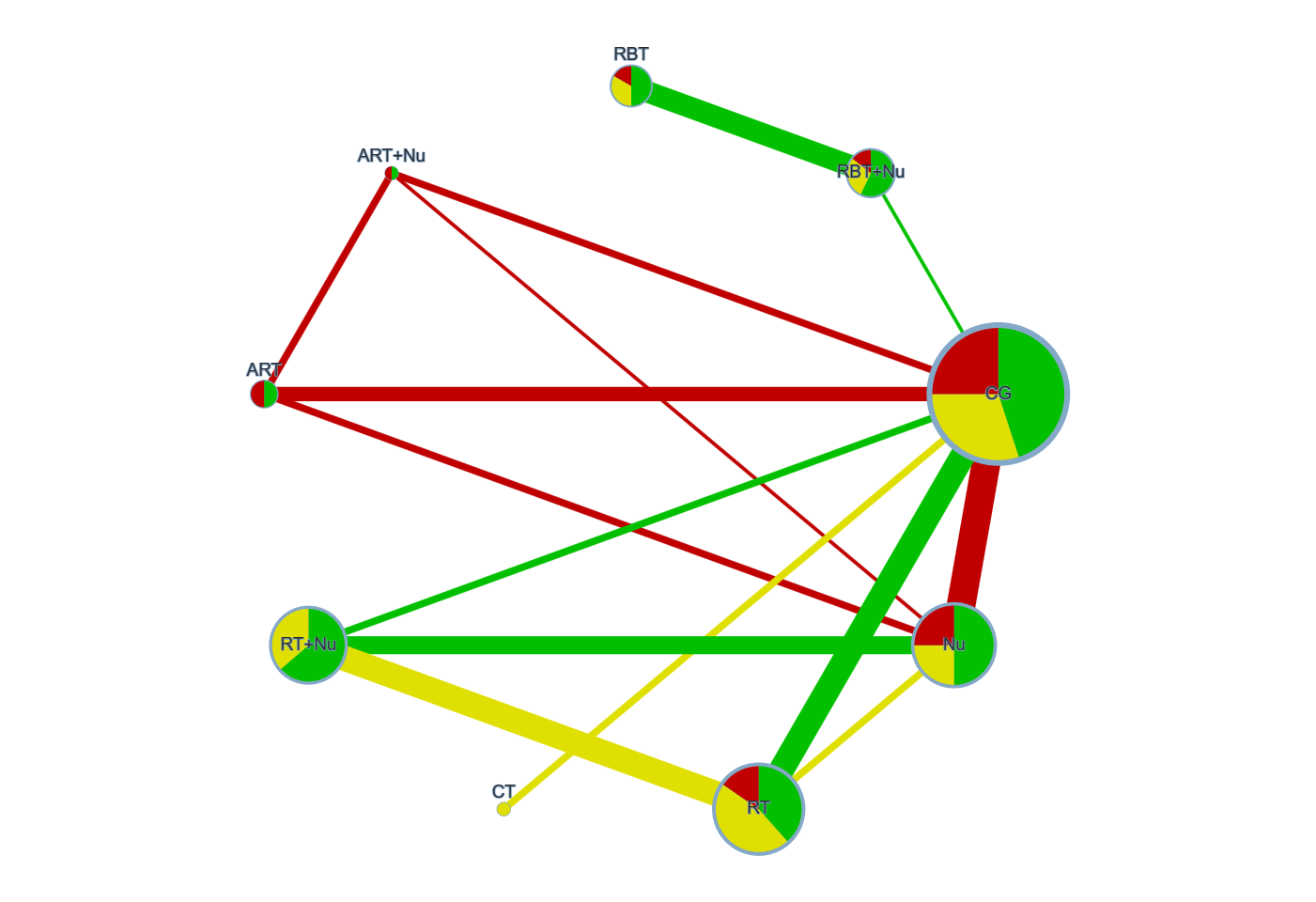


**Table S8.9:** CINeMA Results of ASMI

| Comparison | Within-study  bias | Reporting  bias | Indirectness | Imprecision | Heterogeneity | Incoherence | Confidence  rating |
| --- | --- | --- | --- | --- | --- | --- | --- |
| ART:ART+Nu | Major concerns | Low risk | No concerns | Major concerns | No concerns | No concerns | Very low |
| ART:CG | Major concerns | Low risk | No concerns | Major concerns | No concerns | No concerns | Very low |
| ART:Nu | Major concerns | Low risk | No concerns | Major concerns | No concerns | No concerns | Very low |
| ART+Nu:CG | Major concerns | Low risk | No concerns | Major concerns | No concerns | No concerns | Very low |
| ART+Nu:Nu | Major concerns | Low risk | No concerns | Major concerns | No concerns | No concerns | Very low |
| CG:CT | Some concerns | Low risk | No concerns | Major concerns | No concerns | No concerns | Very low |
| CG:Nu | Some concerns | Low risk | No concerns | No concerns | Major concerns | No concerns | Very low |
| CG:RBT+Nu | No concerns | Low risk | No concerns | Major concerns | No concerns | No concerns | Low |
| CG:RT | No concerns | Low risk | No concerns | No concerns | No concerns | No concerns | High |
| CG:RT+Nu | No concerns | Low risk | No concerns | No concerns | No concerns | No concerns | High |
| Nu:RT | No concerns | Low risk | No concerns | No concerns | Major concerns | No concerns | Low |
| Nu:RT+Nu | No concerns | Low risk | No concerns | No concerns | No concerns | No concerns | High |
| RBT:RBT+Nu | Some concerns | Low risk | No concerns | Major concerns | No concerns | No concerns | Very low |
| RT:RT+Nu | No concerns | Low risk | No concerns | No concerns | No concerns | No concerns | High |
| ART:CT | Some concerns | Low risk | No concerns | Major concerns | No concerns | No concerns | Very low |
| ART:RBT | No concerns | Low risk | No concerns | Major concerns | No concerns | No concerns | Low |
| ART:RBT+Nu | No concerns | Low risk | No concerns | Major concerns | No concerns | No concerns | Low |
| ART:RT | No concerns | Low risk | No concerns | Major concerns | No concerns | No concerns | Low |
| ART:RT+Nu | No concerns | Low risk | No concerns | No concerns | No concerns | No concerns | High |
| ART+Nu:CT | Some concerns | Low risk | No concerns | Major concerns | No concerns | No concerns | Very low |
| ART+Nu:RBT | No concerns | Low risk | No concerns | Major concerns | No concerns | No concerns | Low |
| ART+Nu:RBT+Nu | No concerns | Low risk | No concerns | Major concerns | No concerns | No concerns | Low |
| ART+Nu:RT | No concerns | Low risk | No concerns | Major concerns | No concerns | No concerns | Low |
| ART+Nu:RT+Nu | No concerns | Low risk | No concerns | No concerns | No concerns | No concerns | High |
| CG:RBT | No concerns | Low risk | No concerns | Major concerns | No concerns | No concerns | Low |
| CT:Nu | Some concerns | Low risk | No concerns | Major concerns | No concerns | No concerns | Very low |
| CT:RBT | Some concerns | Low risk | No concerns | Major concerns | No concerns | No concerns | Very low |
| CT:RBT+Nu | No concerns | Low risk | No concerns | Major concerns | No concerns | No concerns | Very low |
| CT:RT | Some concerns | Low risk | No concerns | Major concerns | No concerns | No concerns | Very low |
| CT:RT+Nu | Some concerns | Low risk | No concerns | No concerns | No concerns | No concerns | Moderate |
| Nu:RBT | Some concerns | Low risk | No concerns | Major concerns | No concerns | No concerns | Very low |
| Nu:RBT+Nu | No concerns | Low risk | No concerns | Major concerns | No concerns | No concerns | Low |
| RBT:RT | No concerns | Low risk | No concerns | Major concerns | No concerns | No concerns | Low |
| RBT:RT+Nu | No concerns | Low risk | No concerns | Major concerns | No concerns | No concerns | Low |
| RBT+Nu:RT | No concerns | Low risk | No concerns | Major concerns | No concerns | No concerns | Low |
| RBT+Nu:RT+Nu | No concerns | Low risk | No concerns | Major concerns | No concerns | No concerns | Low |

**Figure S8.8:** Risk of bias contribution by intervention group in SMI

**
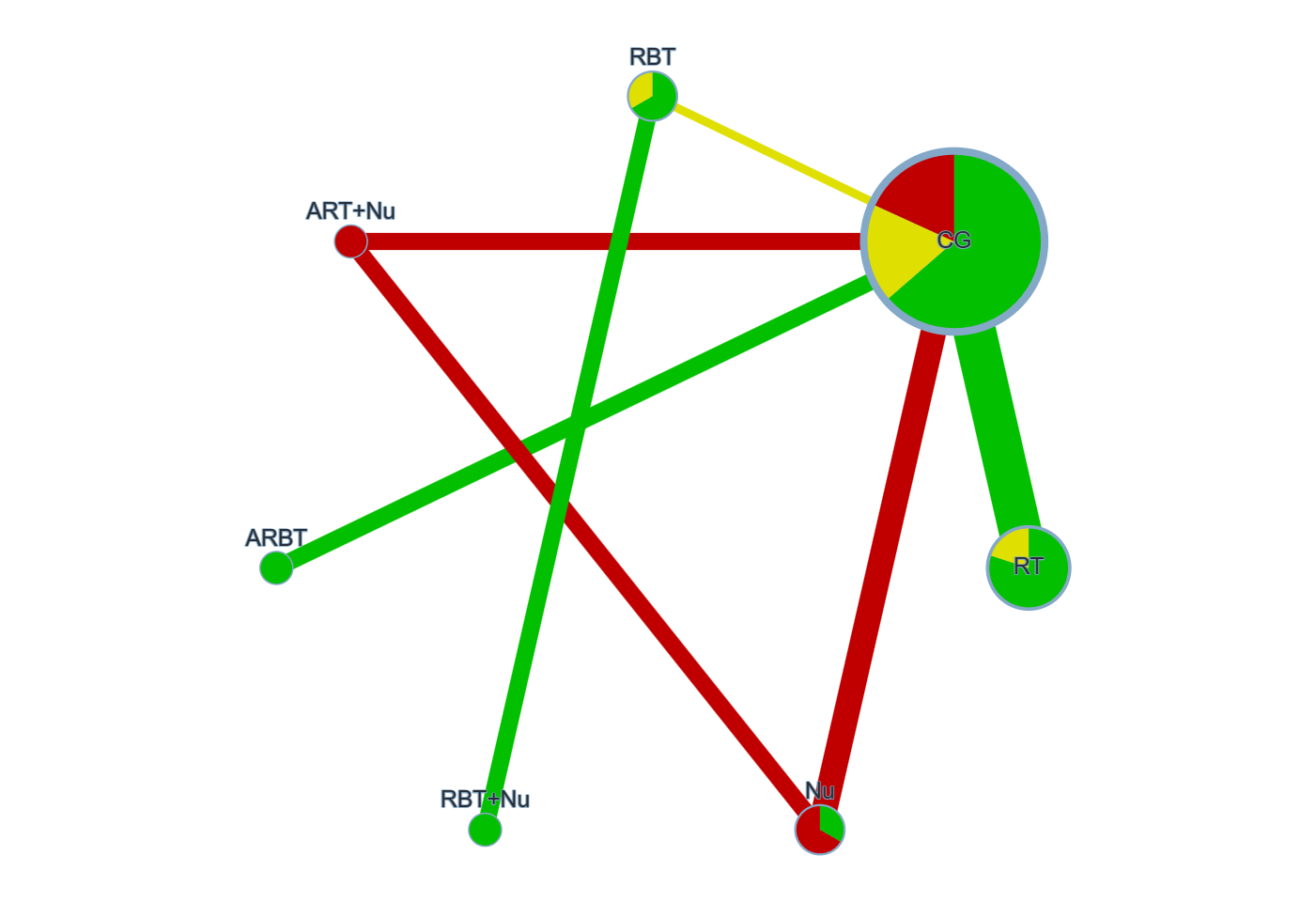
**

**Table S8.10:** CINeMA Results of SMI

| Comparison | Within-study  bias | Reporting  bias | Indirectness | Imprecision | Heterogeneity | Incoherence | Confidence  rating |
| --- | --- | --- | --- | --- | --- | --- | --- |
| ARBT:CG | No concerns | Low risk | No concerns | Major concerns | No concerns | No concerns | Low |
| ART+Nu:CG | Major concerns | Low risk | No concerns | No concerns | No concerns | No concerns | Low |
| ART+Nu:Nu | Major concerns | Low risk | No concerns | Major concerns | No concerns | No concerns | Very low |
| CG:Nu | Major concerns | Low risk | No concerns | No concerns | No concerns | No concerns | Low |
| CG:RBT | Some concerns | Low risk | No concerns | Major concerns | No concerns | No concerns | Very low |
| CG:RT | Some concerns | Low risk | No concerns | Major concerns | No concerns | No concerns | Very low |
| RBT:RBT+Nu | No concerns | Low risk | No concerns | No concerns | Major concerns | No concerns | Low |
| ARBT:ART+Nu | Major concerns | Low risk | No concerns | Major concerns | No concerns | No concerns | Very low |
| ARBT:Nu | Major concerns | Low risk | No concerns | Major concerns | No concerns | No concerns | Very low |
| ARBT:RBT | No concerns | Low risk | No concerns | Major concerns | No concerns | No concerns | Low |
| ARBT:RBT+Nu | No concerns | Low risk | No concerns | Major concerns | No concerns | No concerns | Low |
| ARBT:RT | No concerns | Low risk | No concerns | Major concerns | No concerns | No concerns | Low |
| ART+Nu:RBT | Major concerns | Low risk | No concerns | Major concerns | No concerns | No concerns | Very low |
| ART+Nu:RBT+Nu | Major concerns | Low risk | No concerns | Major concerns | No concerns | No concerns | Very low |
| ART+Nu:RT | Major concerns | Low risk | No concerns | No concerns | No concerns | No concerns | Low |
| CG:RBT+Nu | No concerns | Low risk | No concerns | No concerns | No concerns | No concerns | High |
| Nu:RBT | Major concerns | Low risk | No concerns | Major concerns | No concerns | No concerns | Very low |
| Nu:RBT+Nu | Major concerns | Low risk | No concerns | Major concerns | No concerns | No concerns | Very low |
| Nu:RT | Major concerns | Low risk | No concerns | No concerns | No concerns | No concerns | Low |
| RBT:RT | Some concerns | Low risk | No concerns | Major concerns | No concerns | No concerns | Very low |
| RBT+Nu:RT | Some concerns | Low risk | No concerns | No concerns | Major concerns | No concerns | Very low |

# Appendix 9: Funnel Plots with Egger’s Test for Publication Bias

**Figure S9.1:** Funnel plot of Grip strength

**
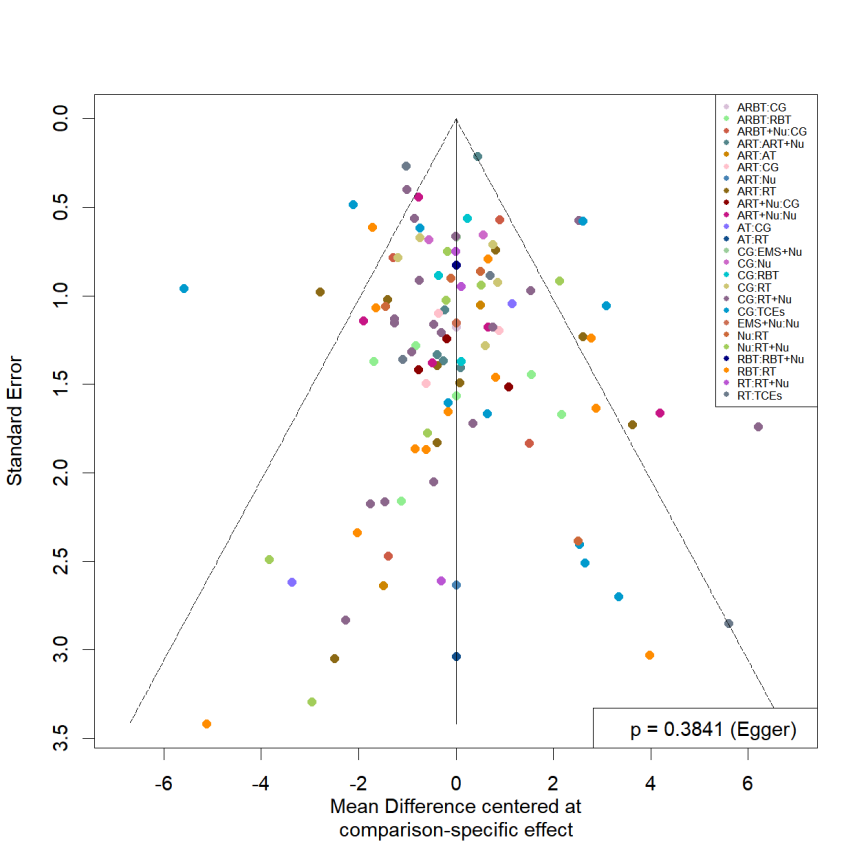
**

**Figure S9.2:** Funnel plot of Knee extension strength


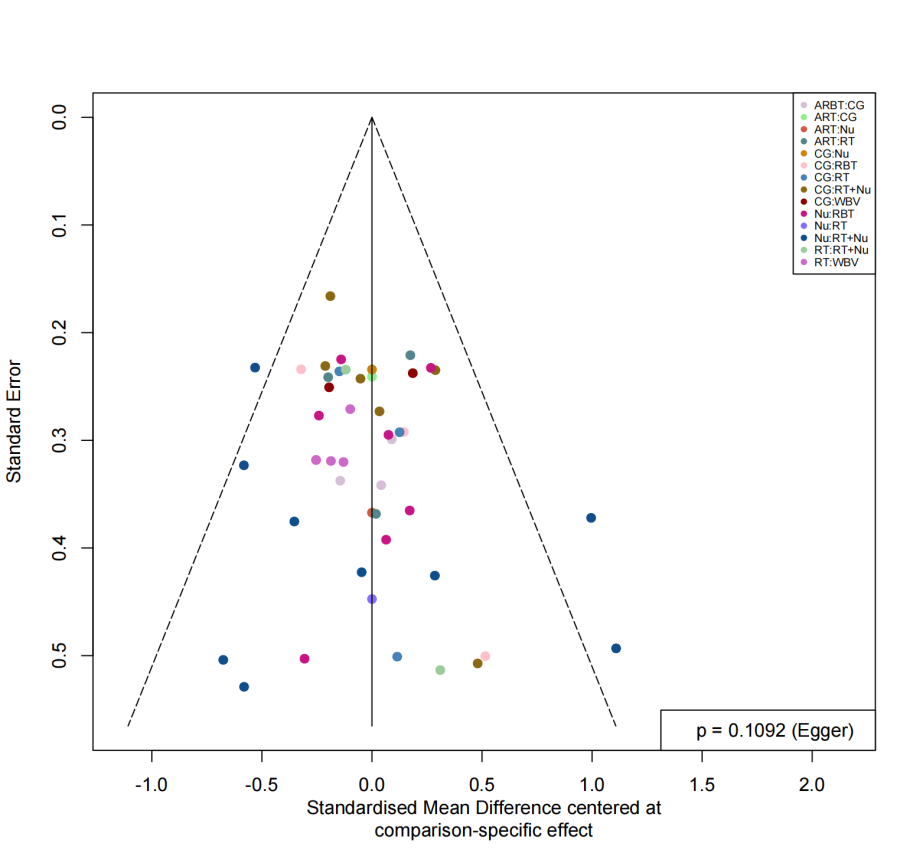


**Figure S9.3:** Funnel plot of Gait speed


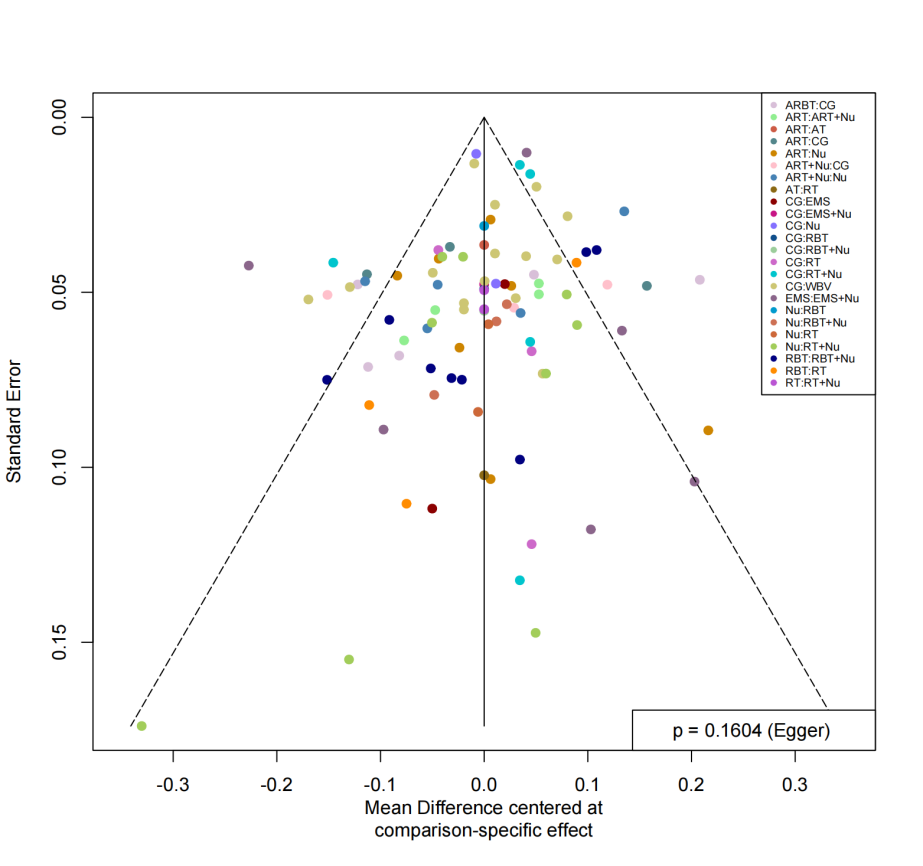


**Figure S9.4:** Funnel plot of Five-Times Sit-to-Stand test


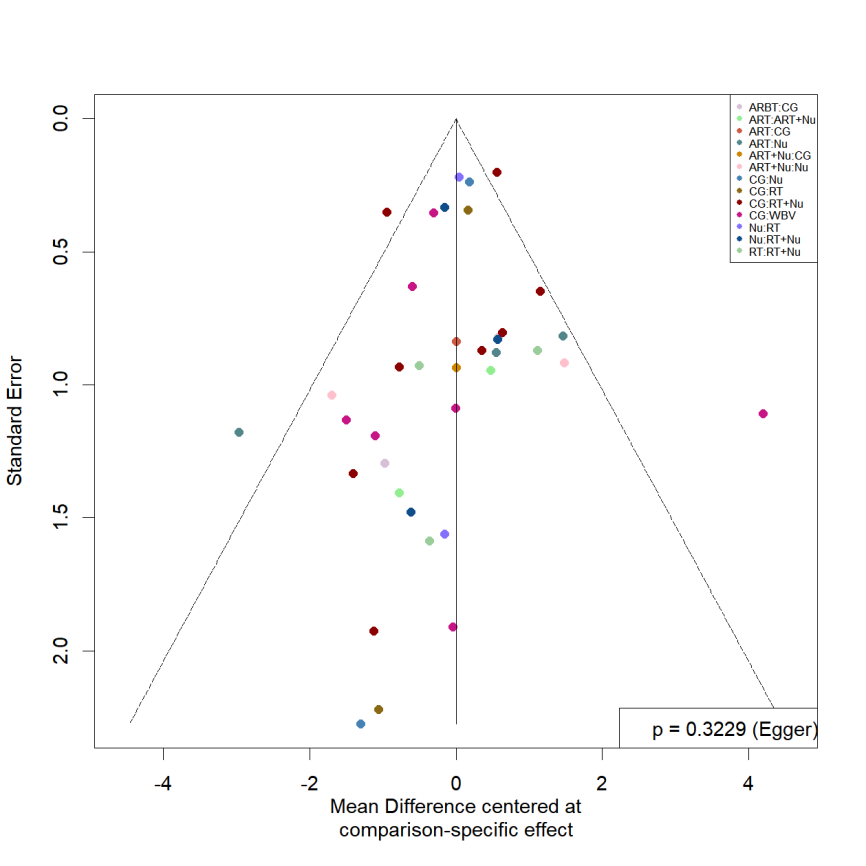


**Figure S9.5:** Funnel plot of Timed up and go


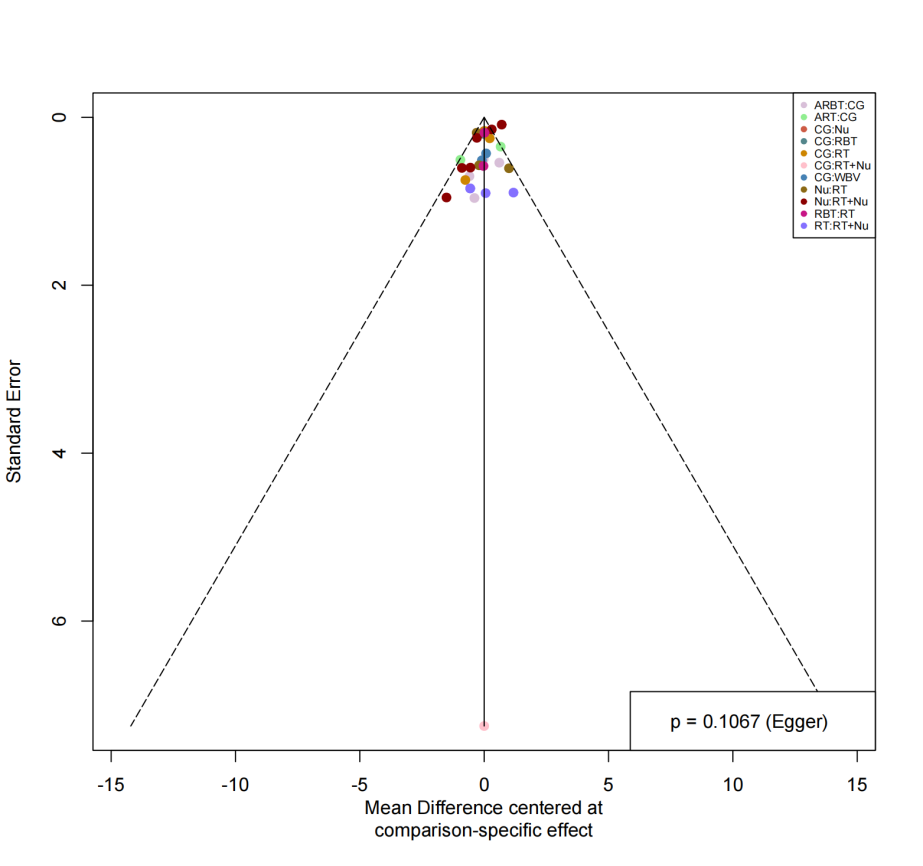


**Figure S9.6:** Funnel plot of SPPB


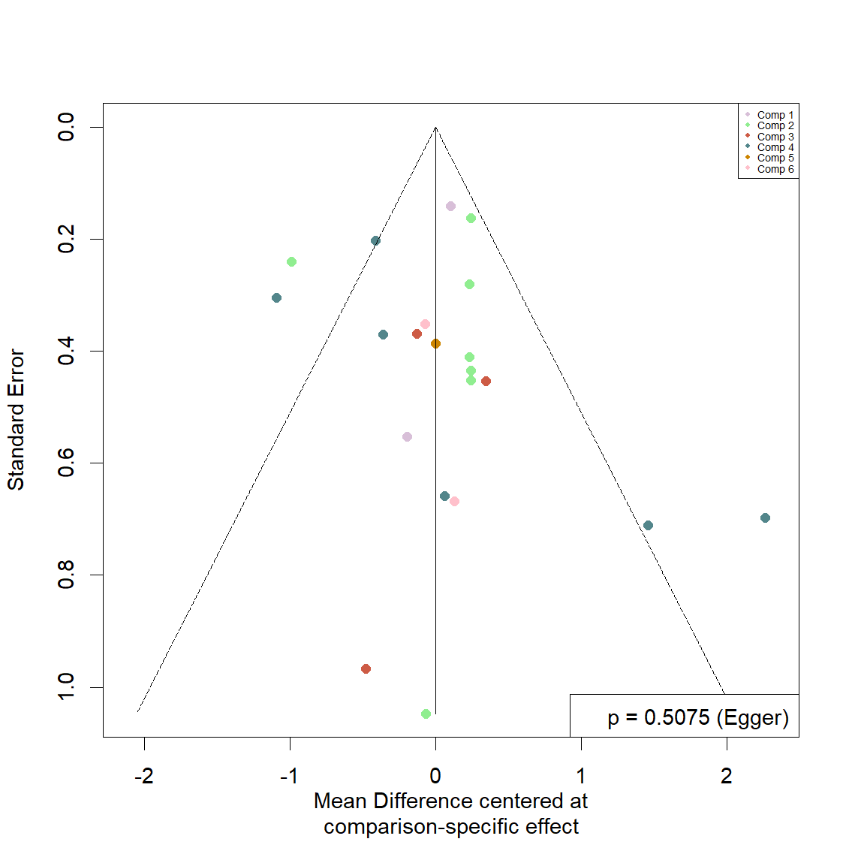


**Figure S9.7:** Funnel plot of Balance test


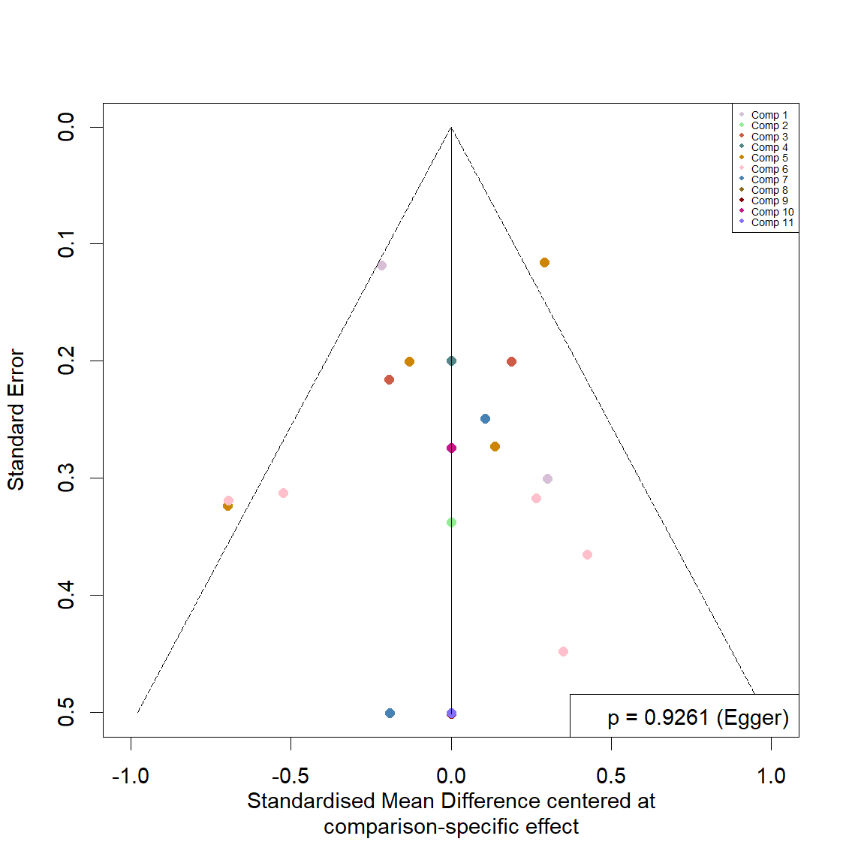


**Figure S9.8:** Funnel plot of ASMI


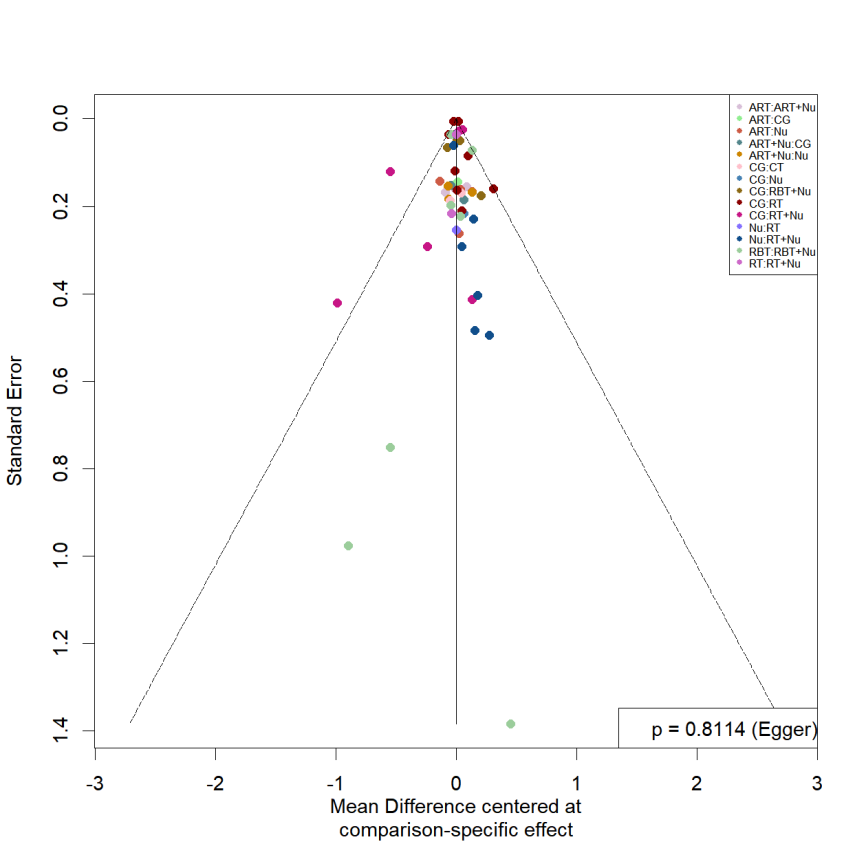


**Figure S9.9:** Funnel plot of SMI


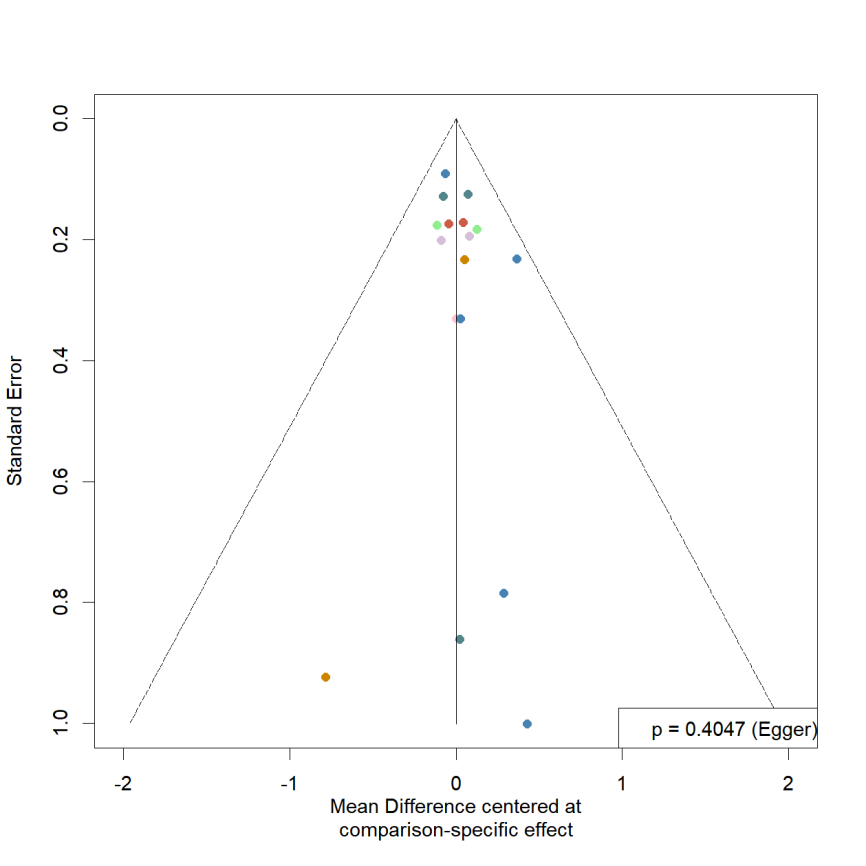


# Appendix 10: Subgroup Analysis

**Appendix 10.1 Main Subgroup Analysis: Subgroup Analysis of Different Protein Supplementation Strategies and Their Combinations with Exercise Interventions**

**Figure S10.1** Network map of Grip Strength


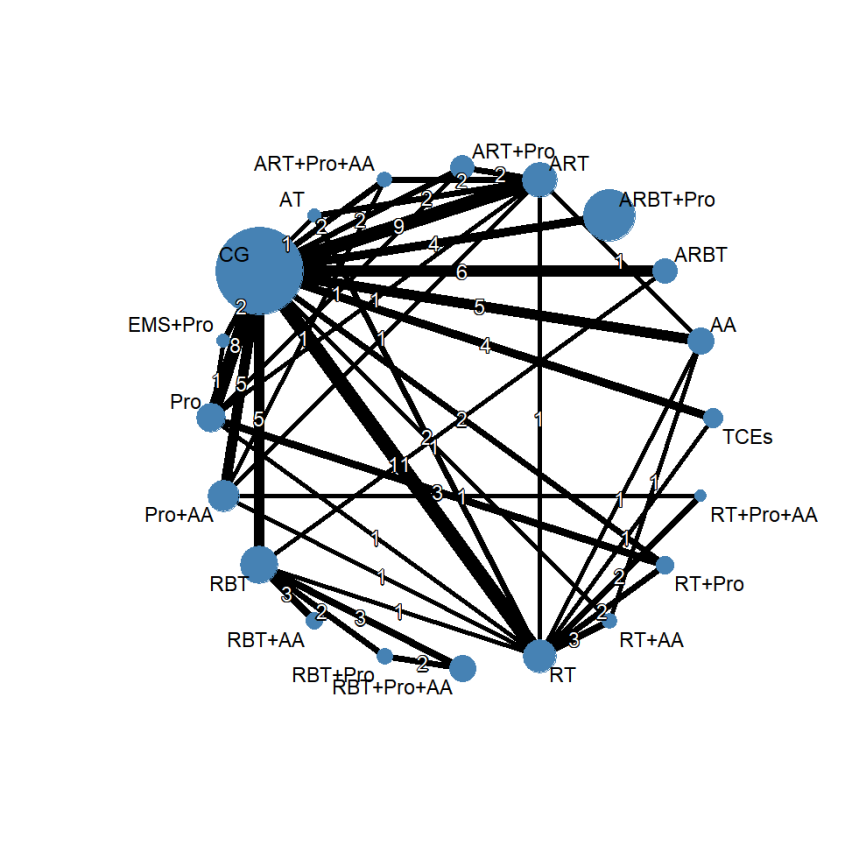


**Figure S10.2** Forest plot of Grip Strength


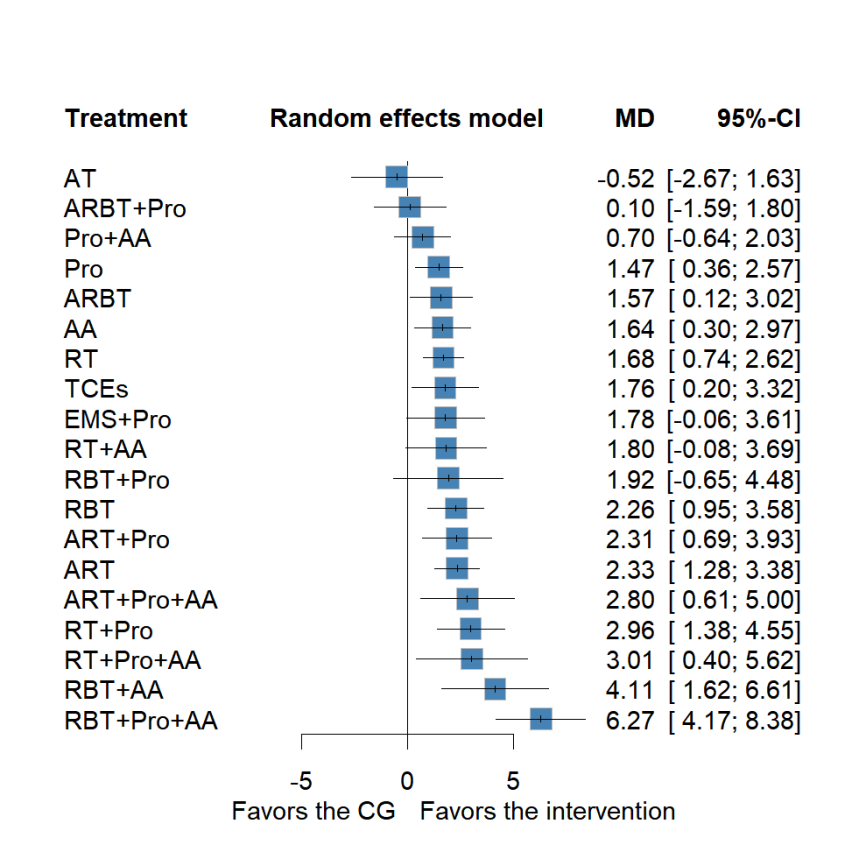


**Table S10.1** League table of Grip strength


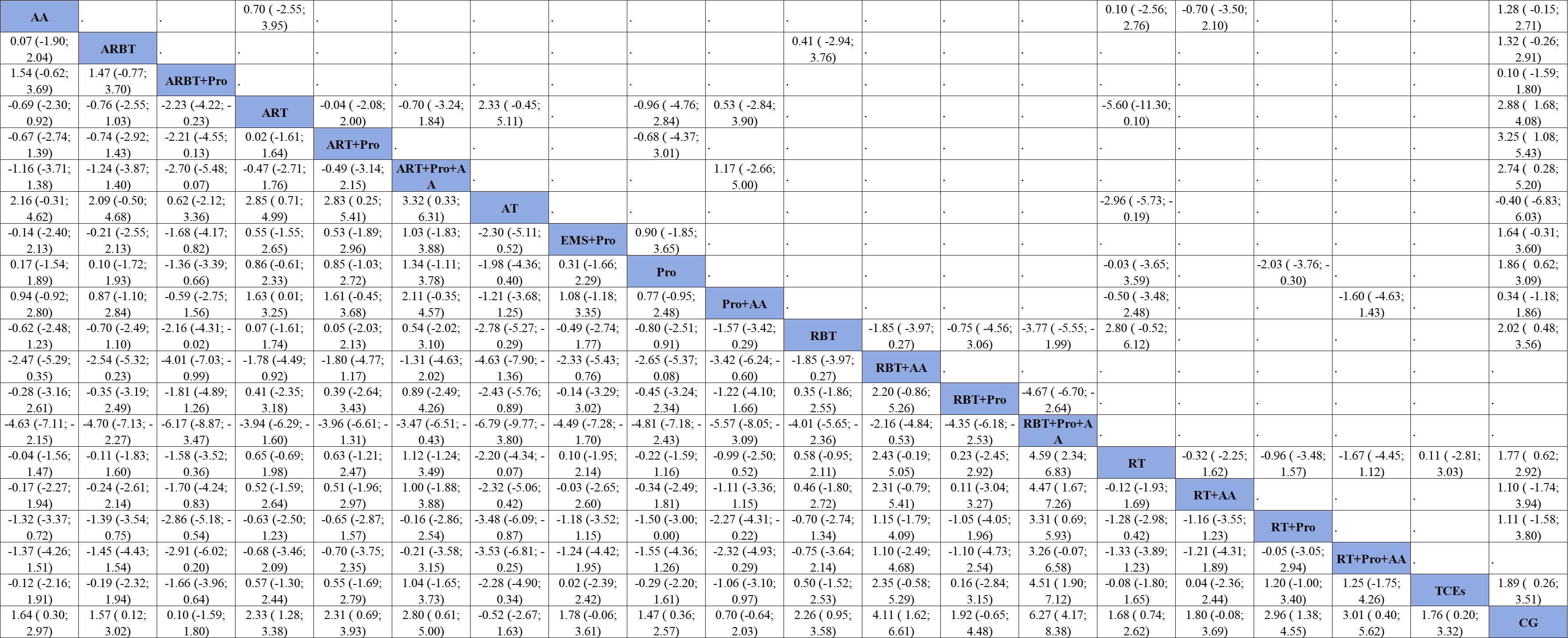


**Figure S10.3** Network map of Knee extension strength


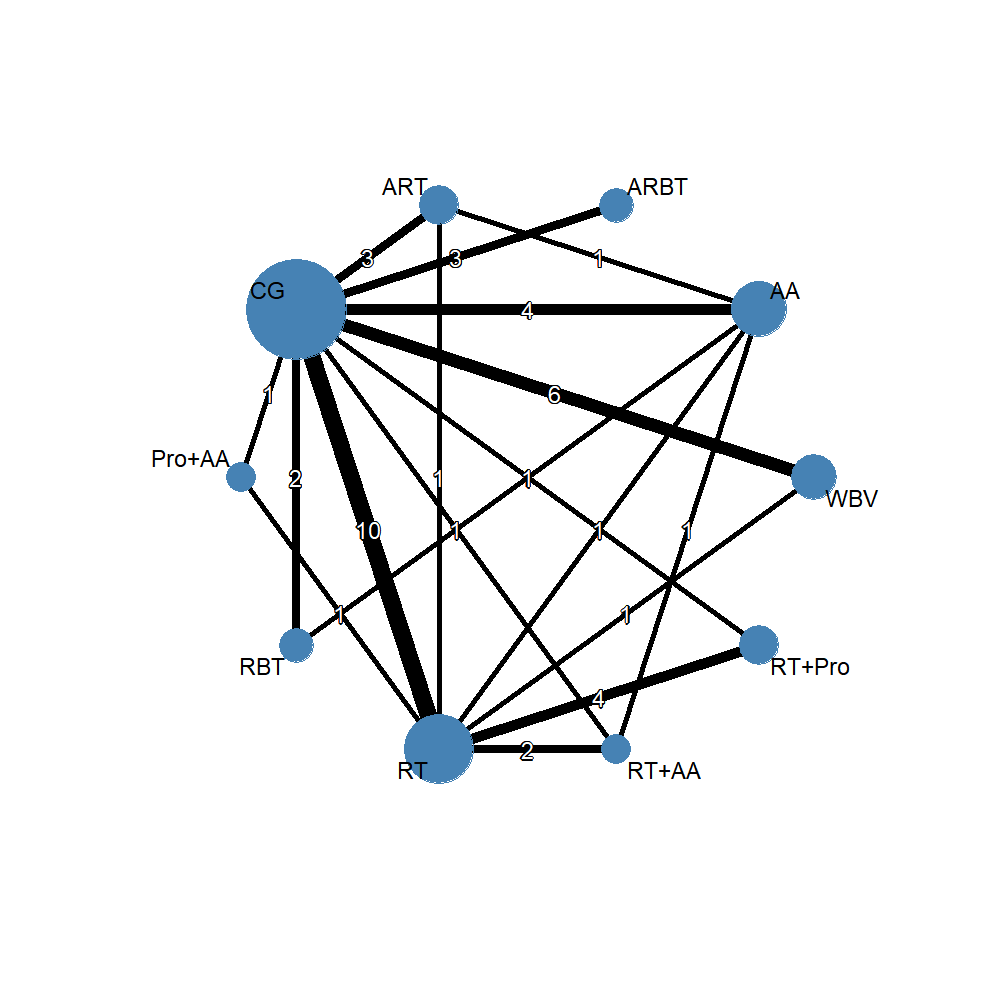


**Figure S10.4** Forest plot of Knee extension strength


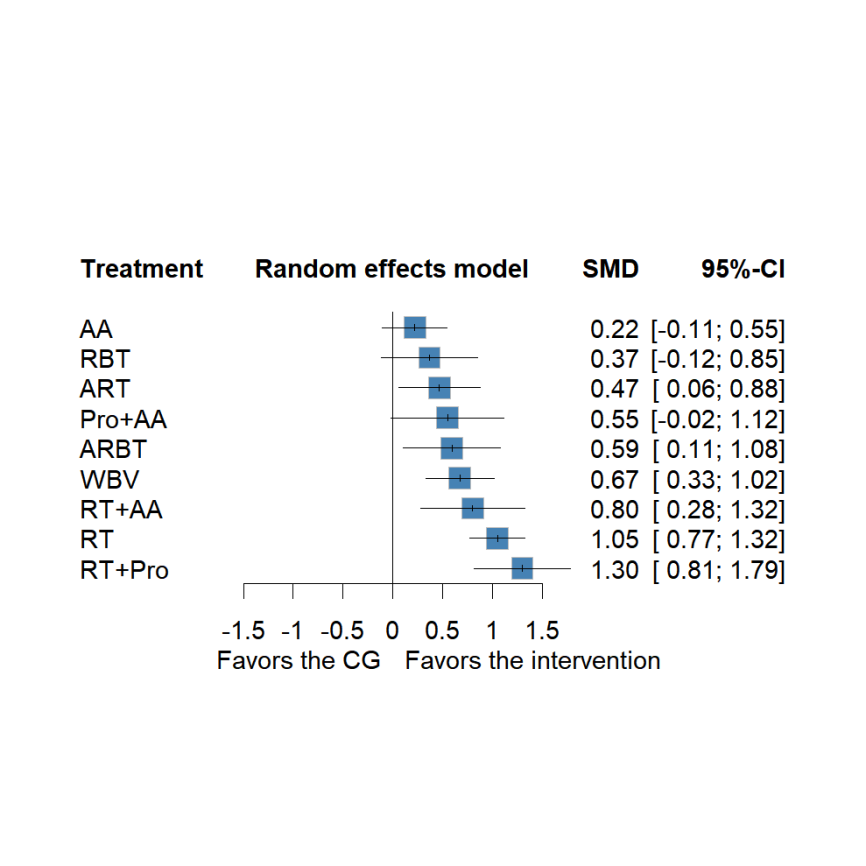


**Table S10.2** League table of Knee extension strength


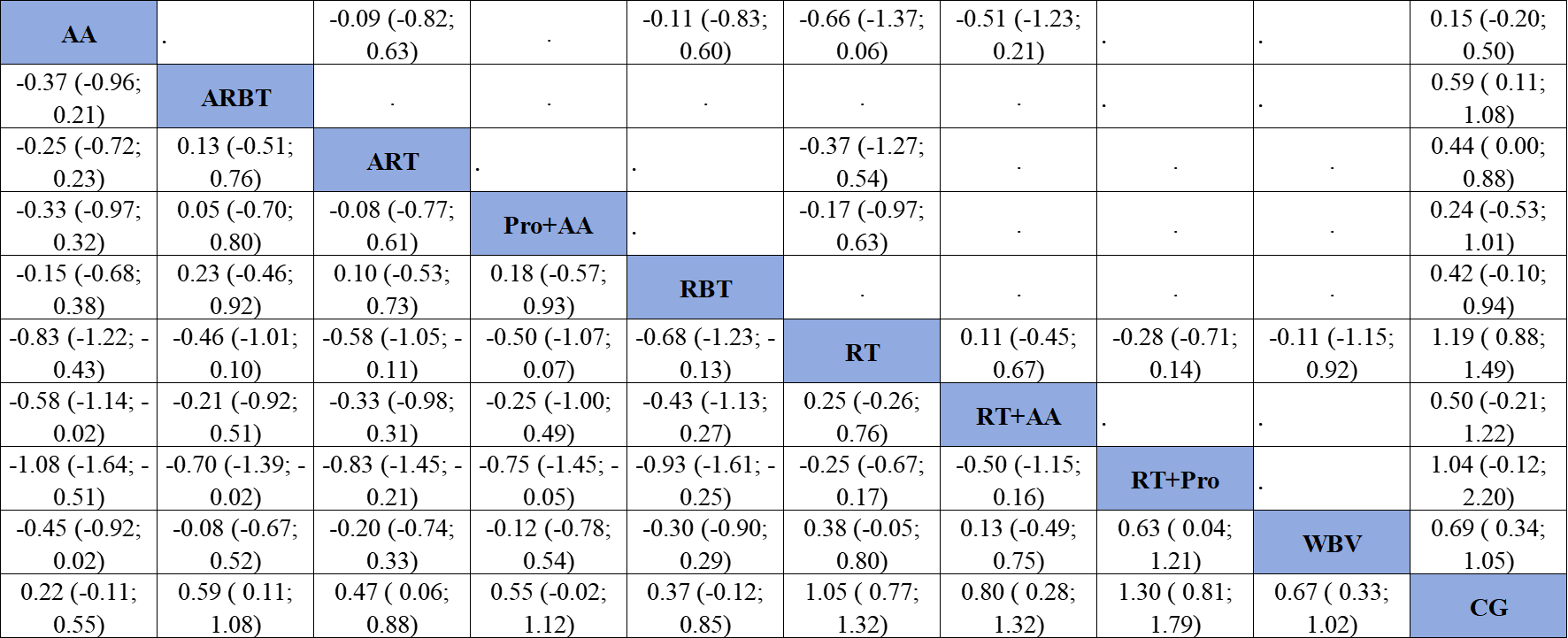


**Figure S10.5** Network map of Gait speed


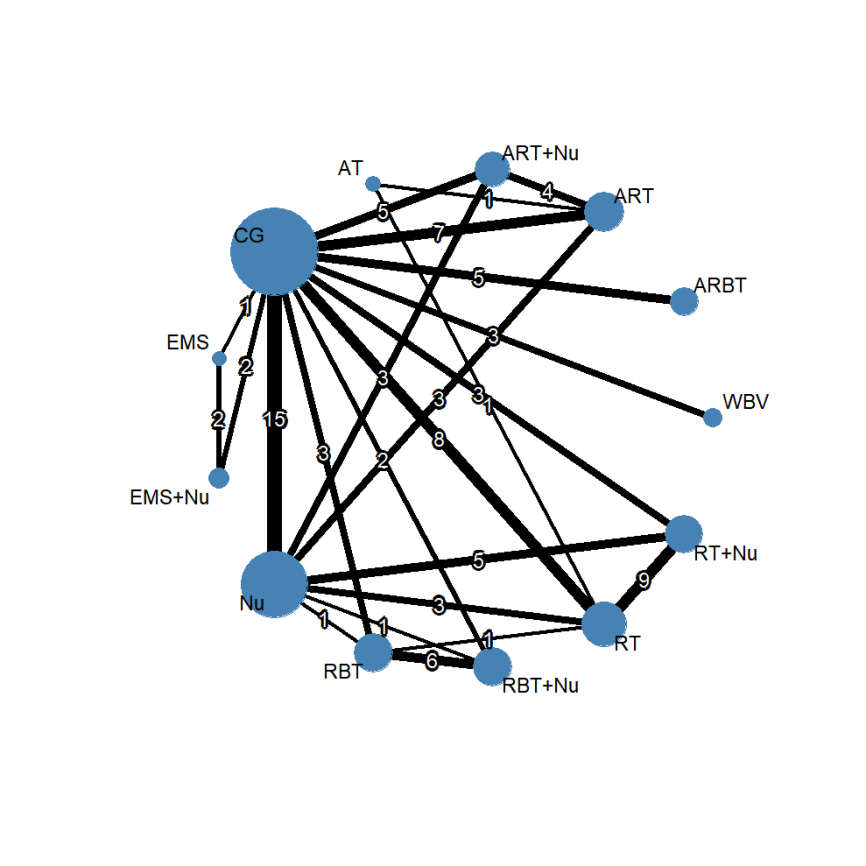


**Figure S10.6** Forest plot of Gait speed


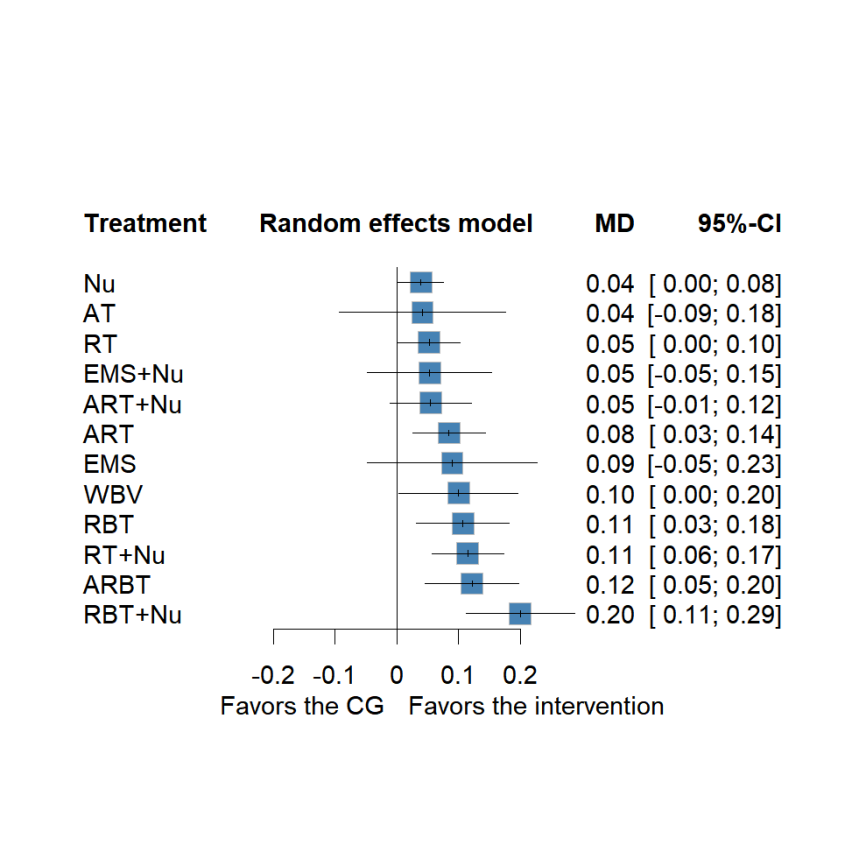


**Table S10.3** League table of Gait speed


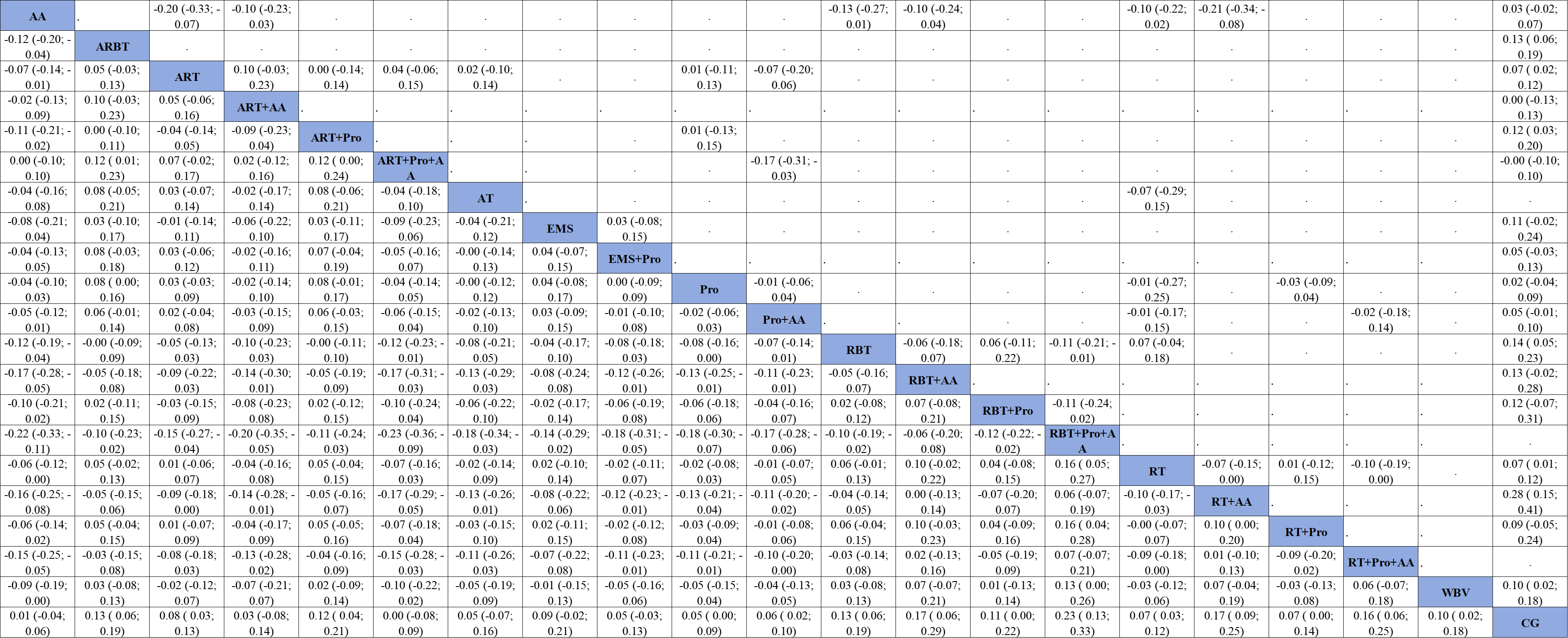


**Figure S10.7** Network map of Five-Times Sit-to-Stand test


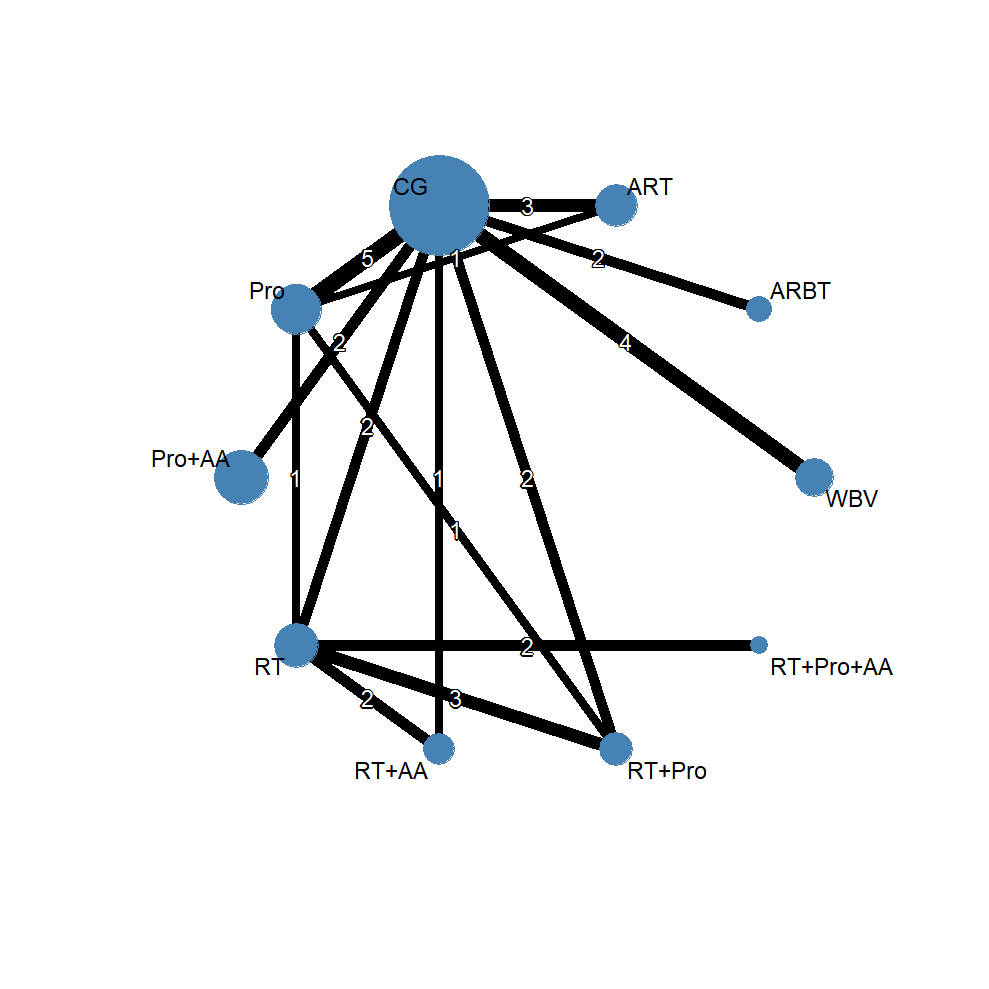


**Figure S10.8** Forest plot of Five-Times Sit-to-Stand test


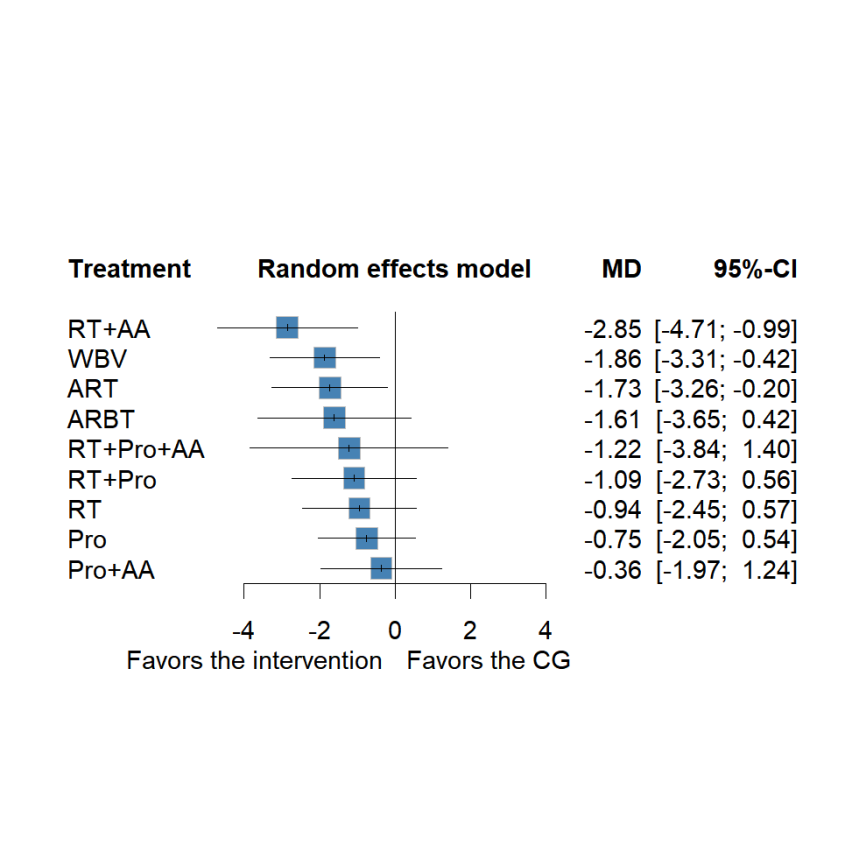


**Table S10.4** League table of Five-Times Sit-to-Stand test


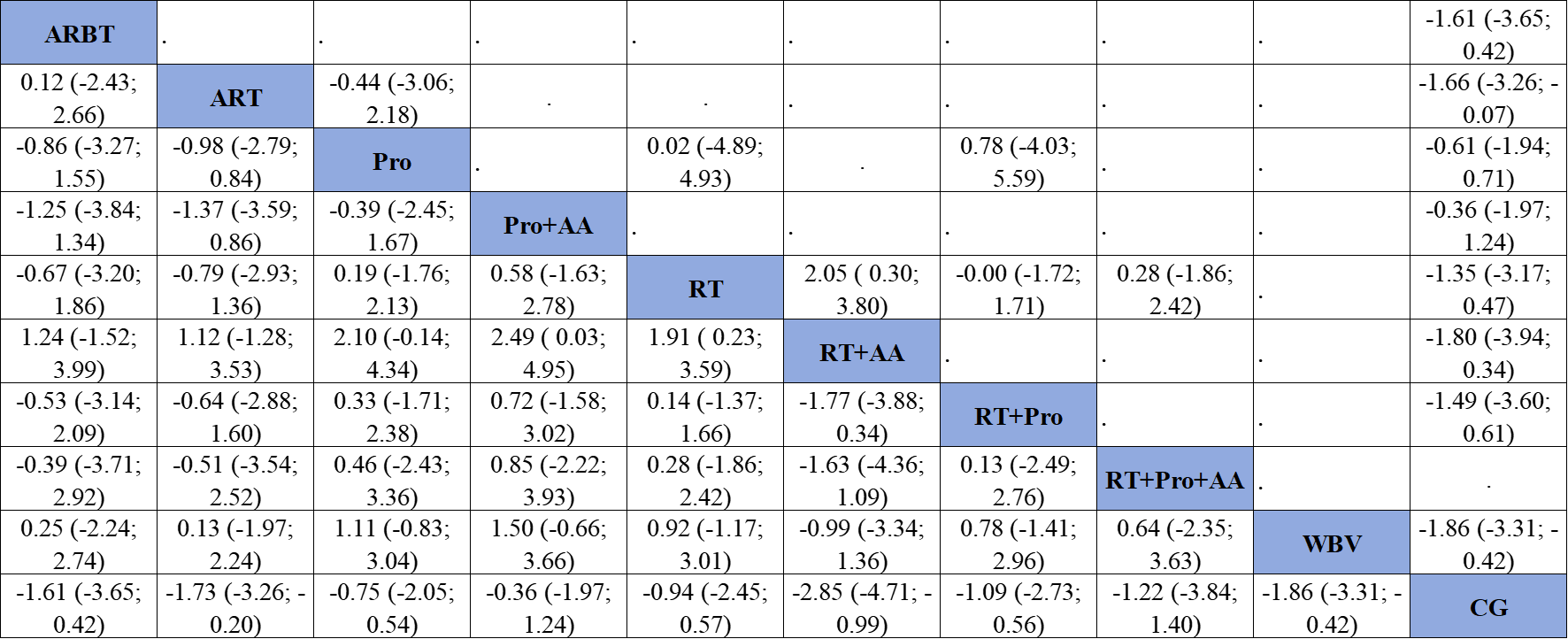


**Figure S10.9** Network map of Timed up and go test.


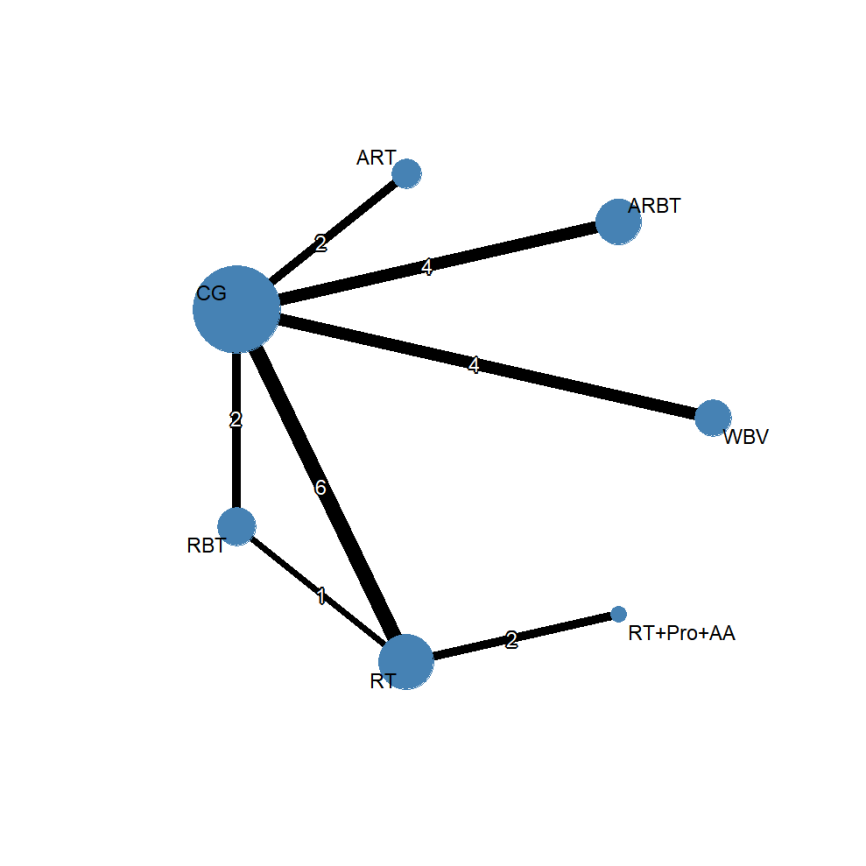


**Figure S10.10** Forest plot of Timed up and go test.


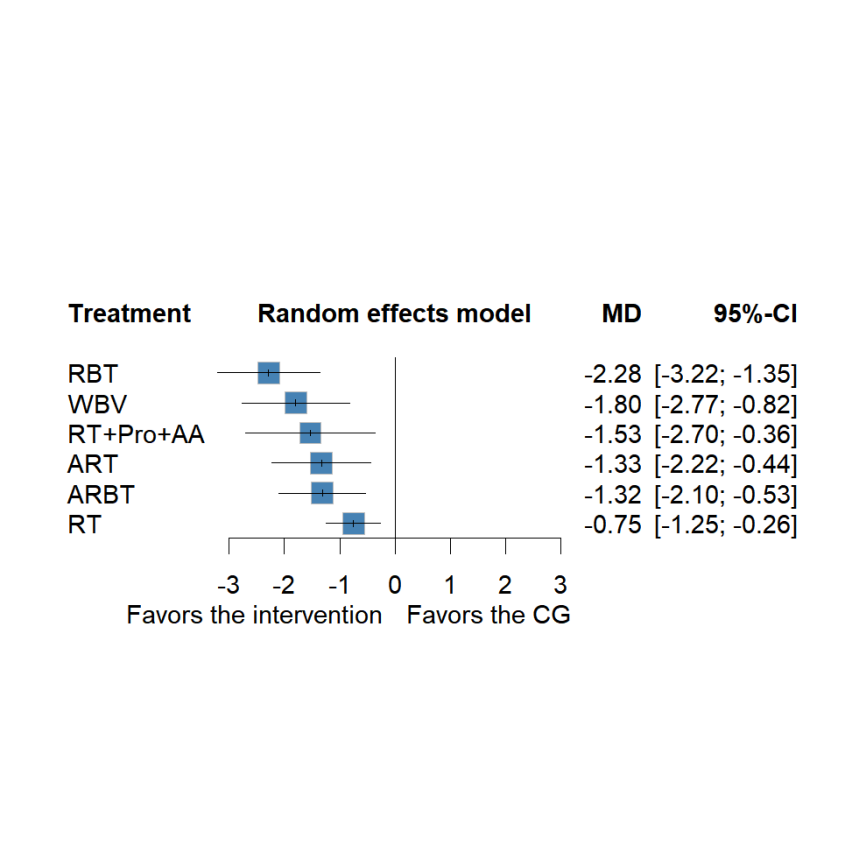


**Table S10.5** League table of Timed up and go test.


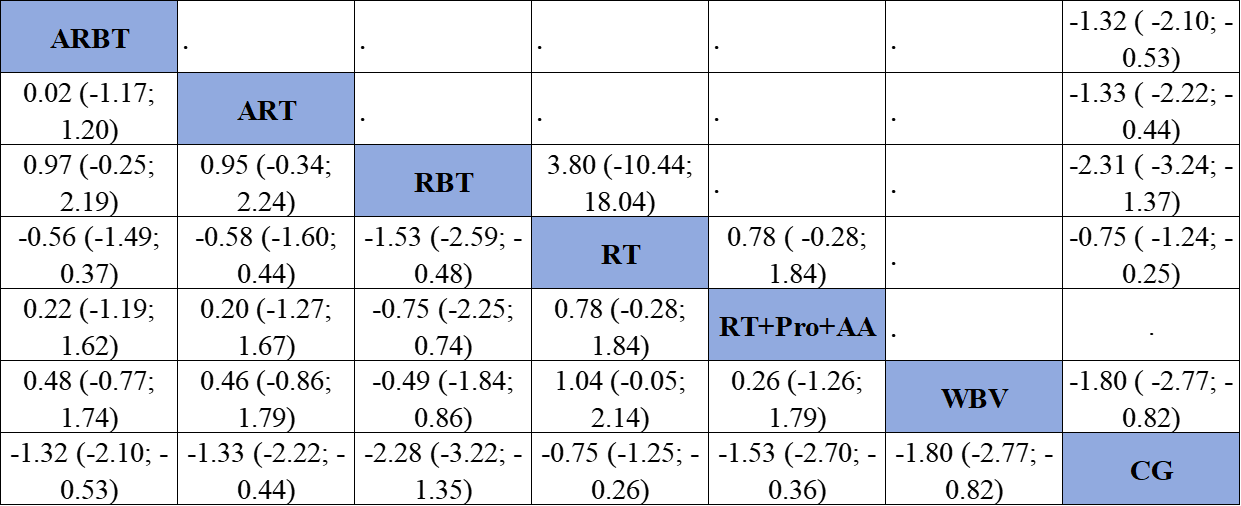


**Figure S10.11** Network map of SPPB


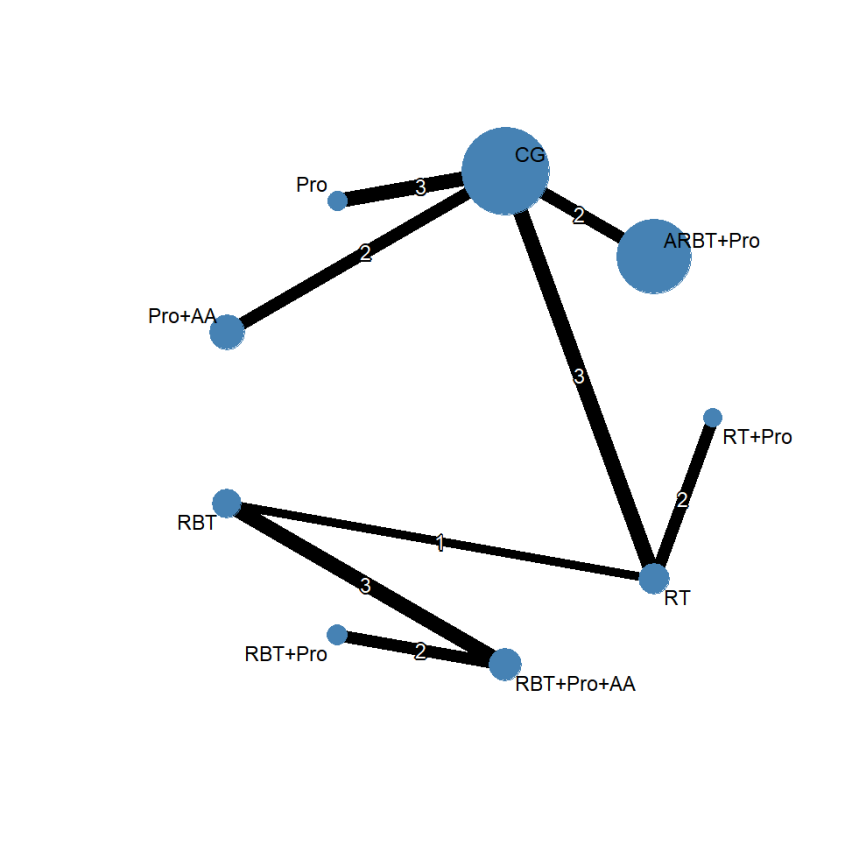


**Figure S10.12** Forest plot of SPPB


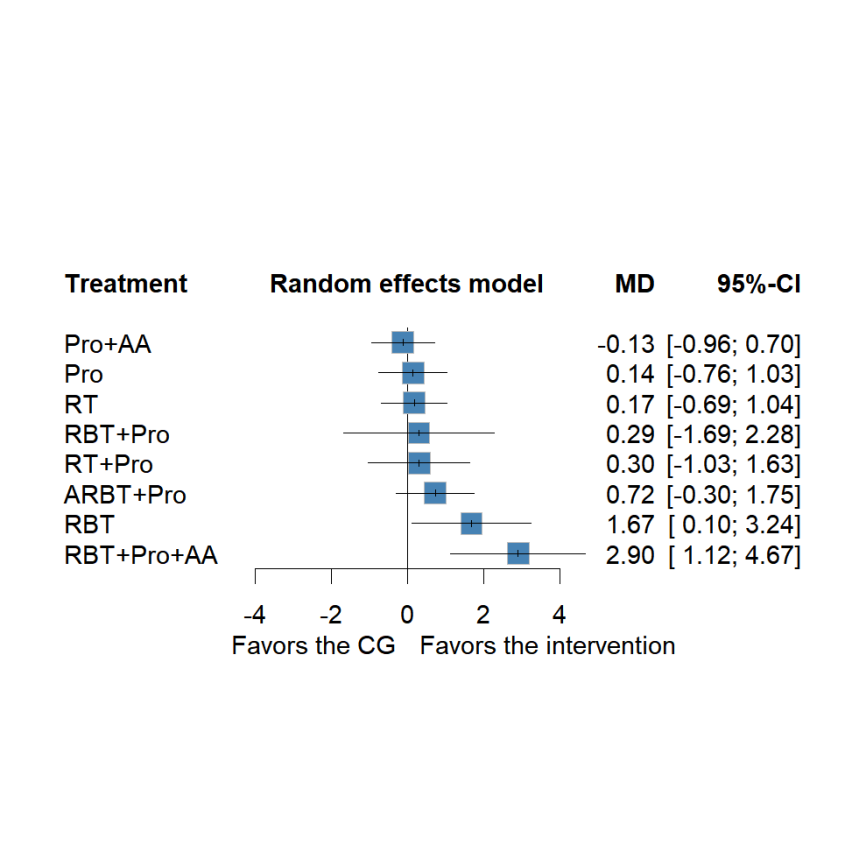


**Table S10.6** League table of SPPB


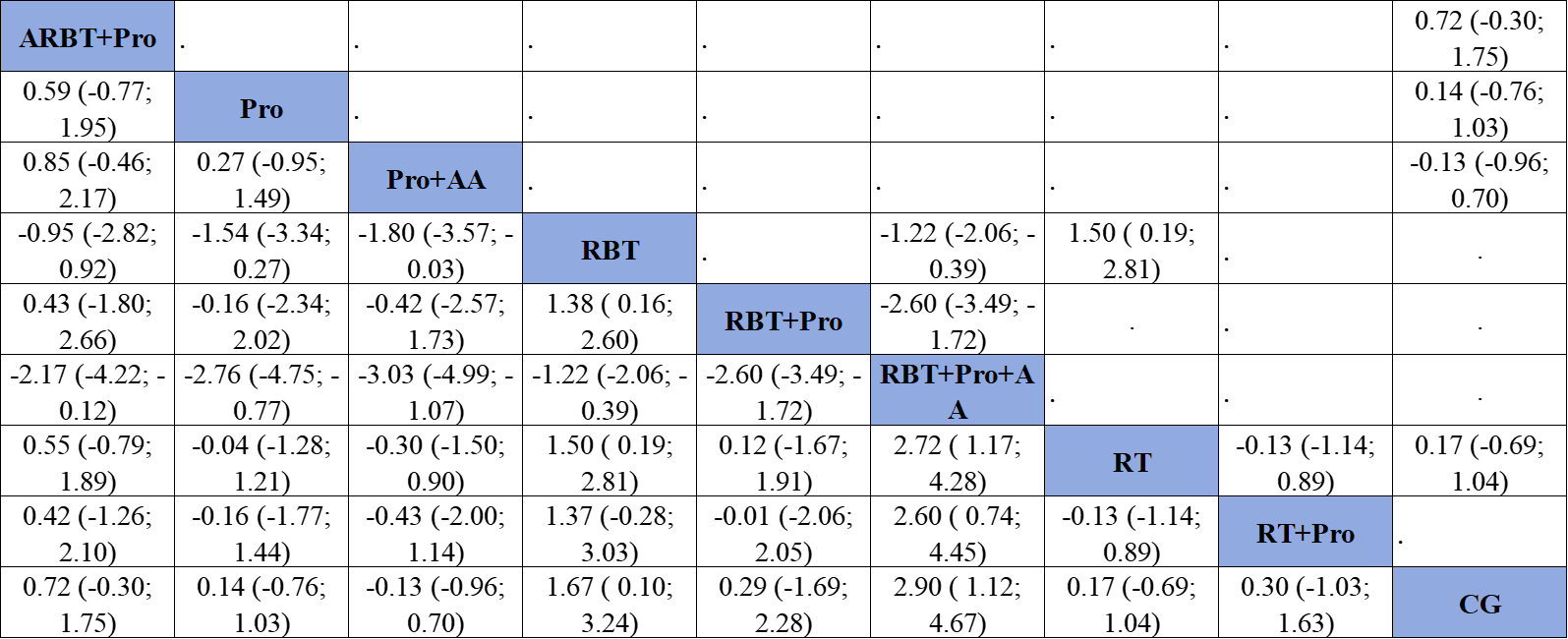


**Figure S10.13** Network map of Balance test


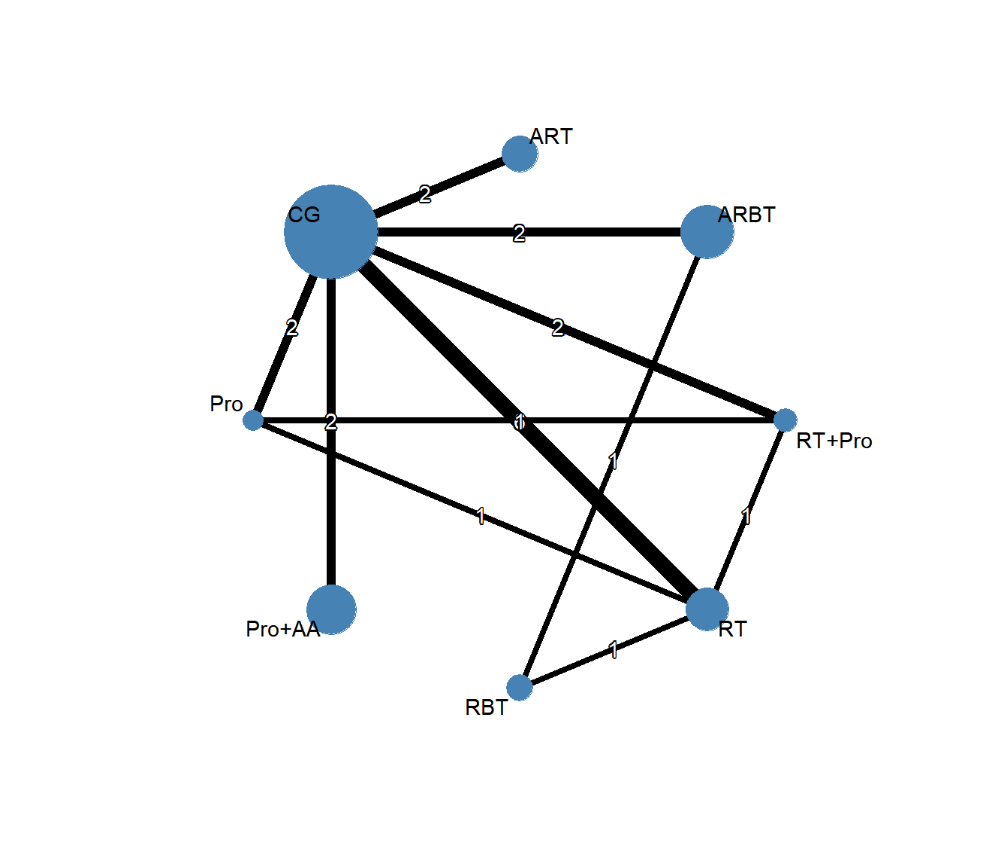


**Figure S10.14** Forest plot of Balance test


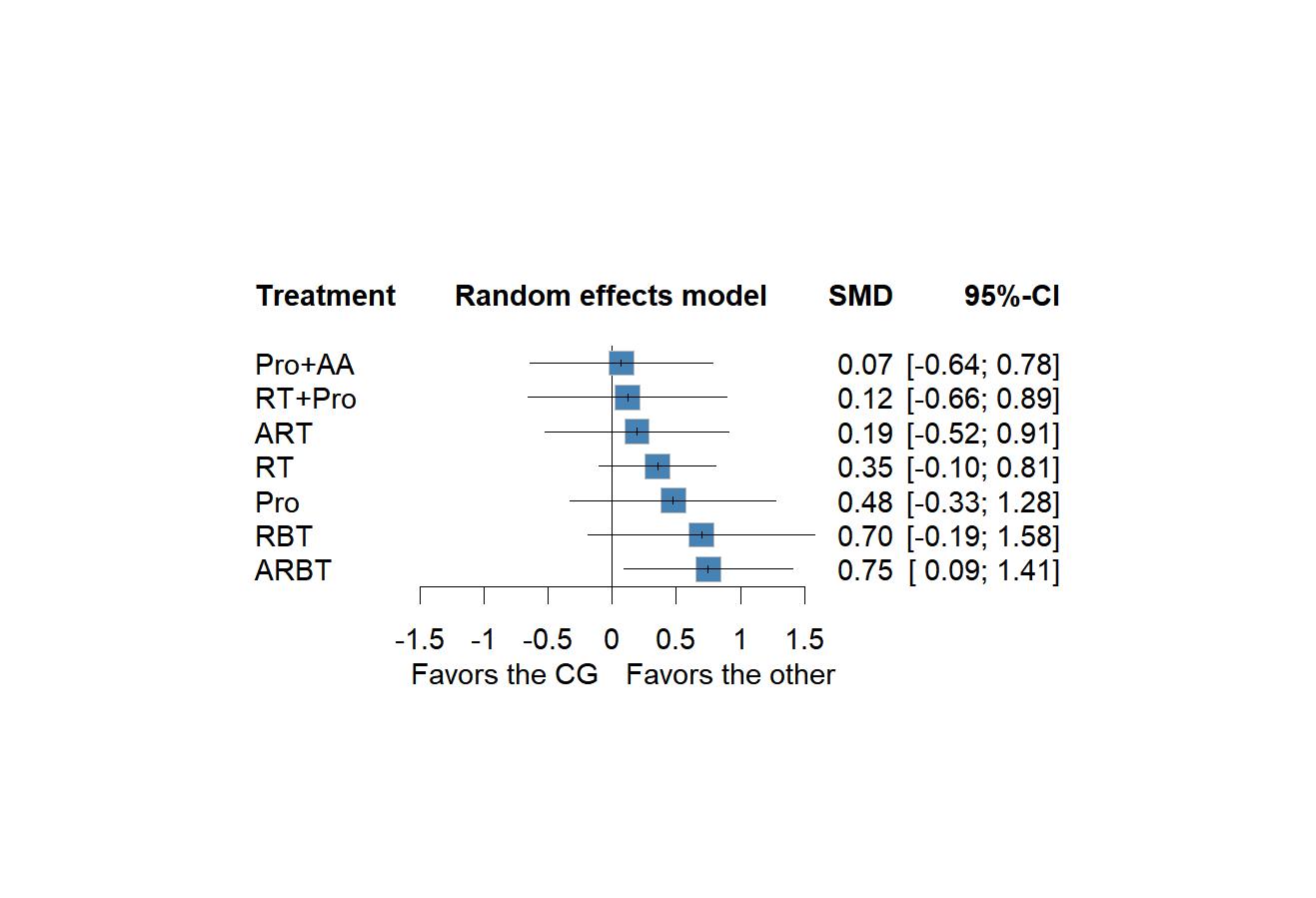


**Table S10.7** League table of Balance test

**Figure S10.15** Network map of ASMI


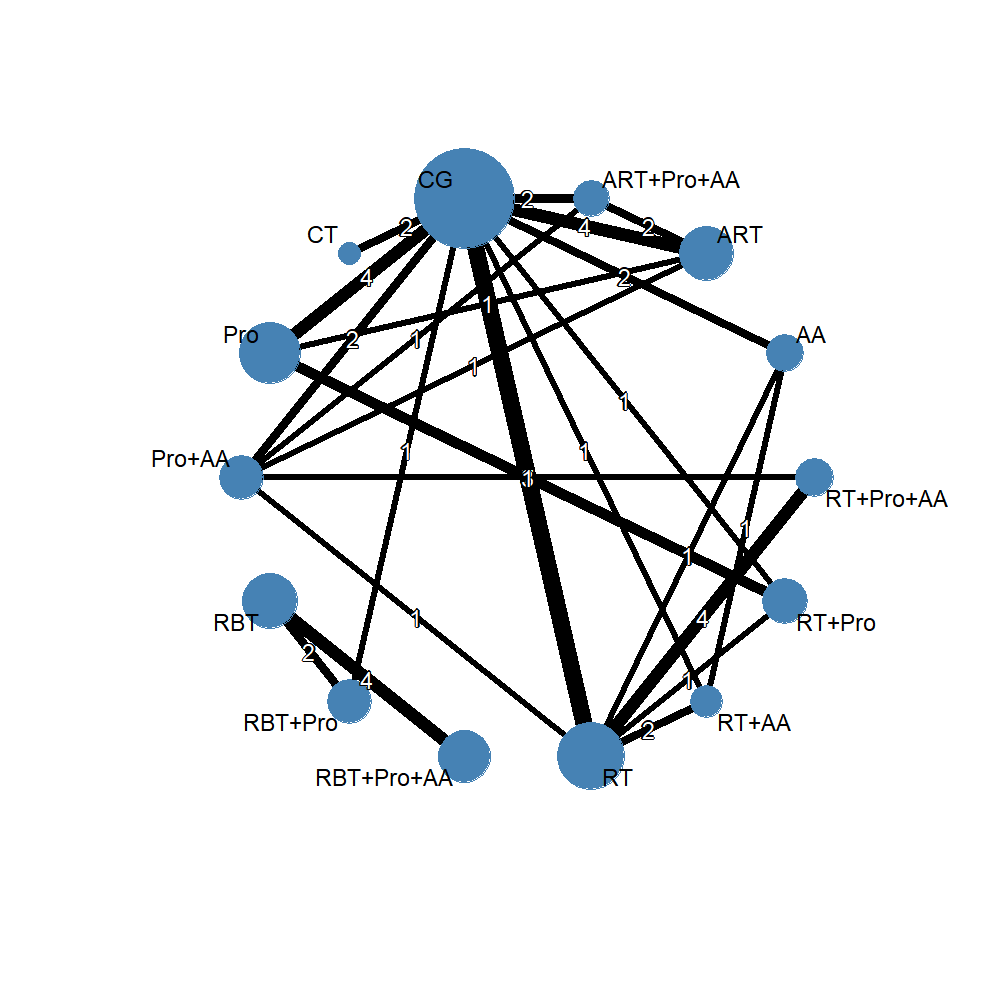


**Figure S10.16** Forest plot of ASMI


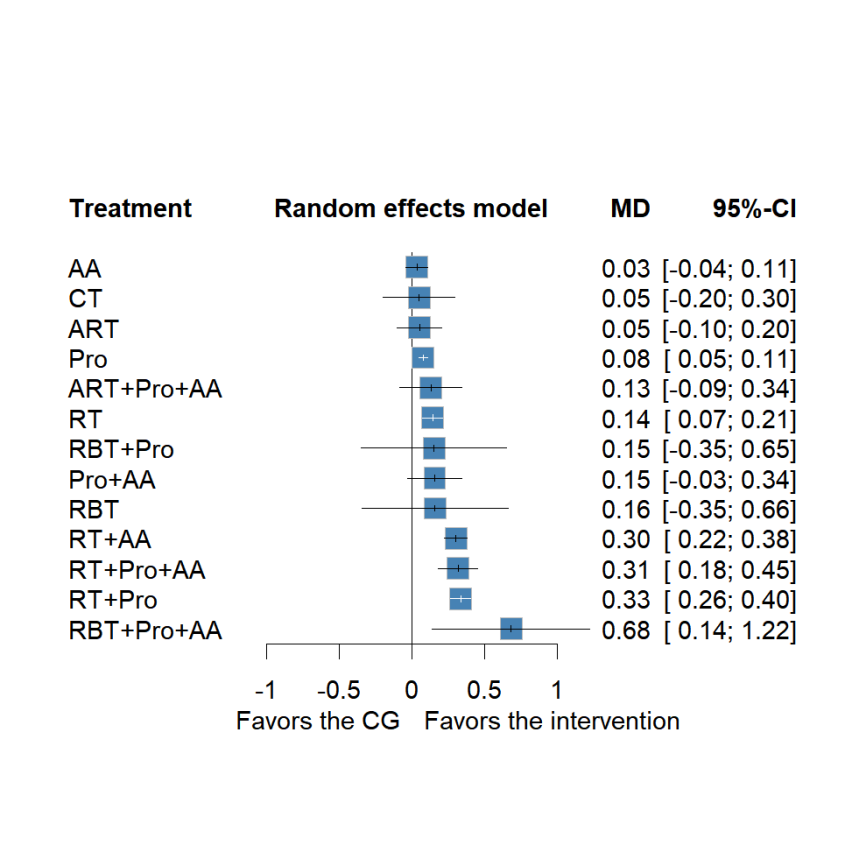


**Table S10.8** League table of ASMI


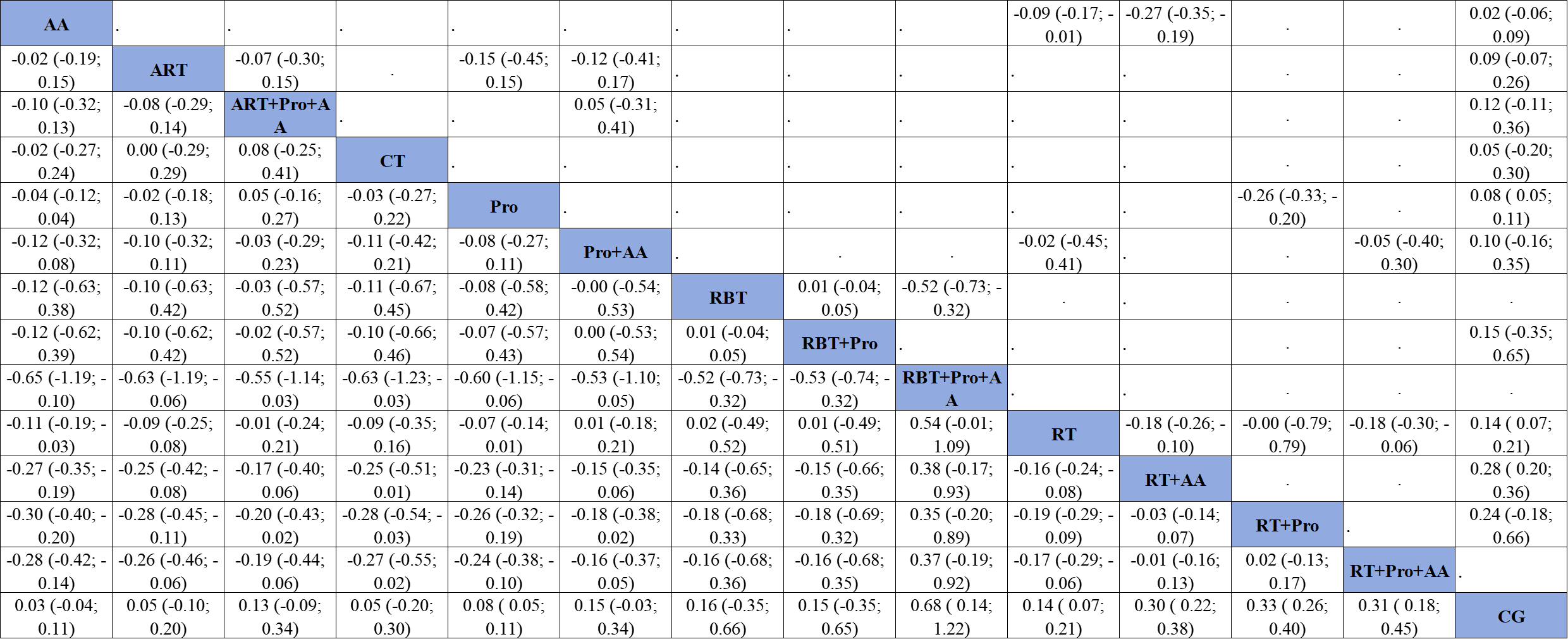


**Figure S10.17** Network map of SMI


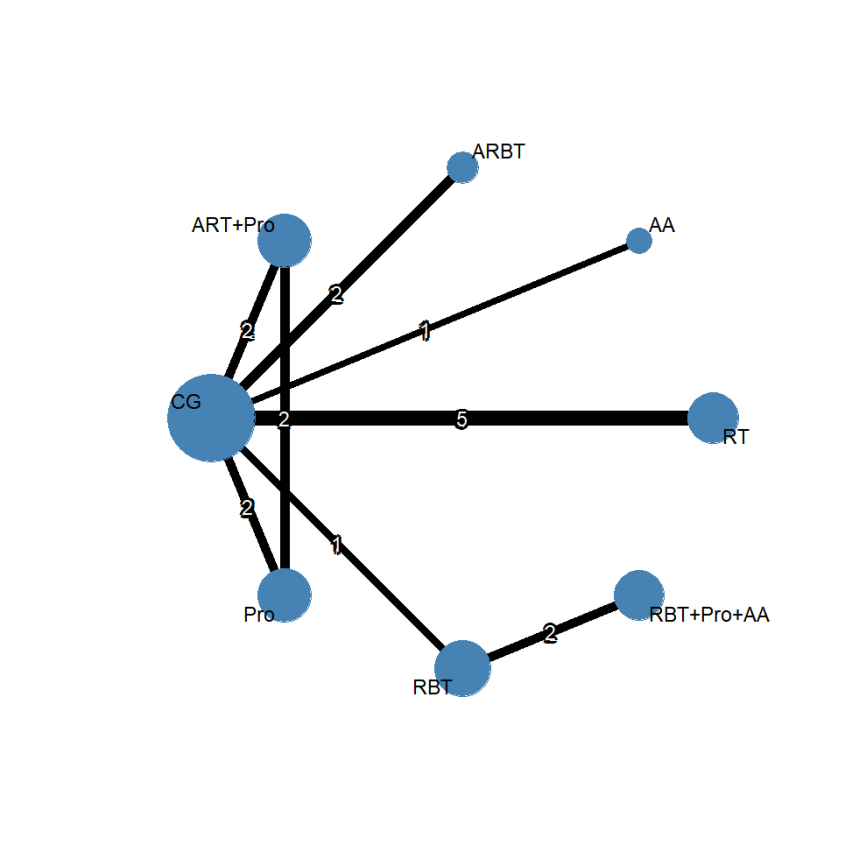


**Figure S10.18** Forest plot of SMI


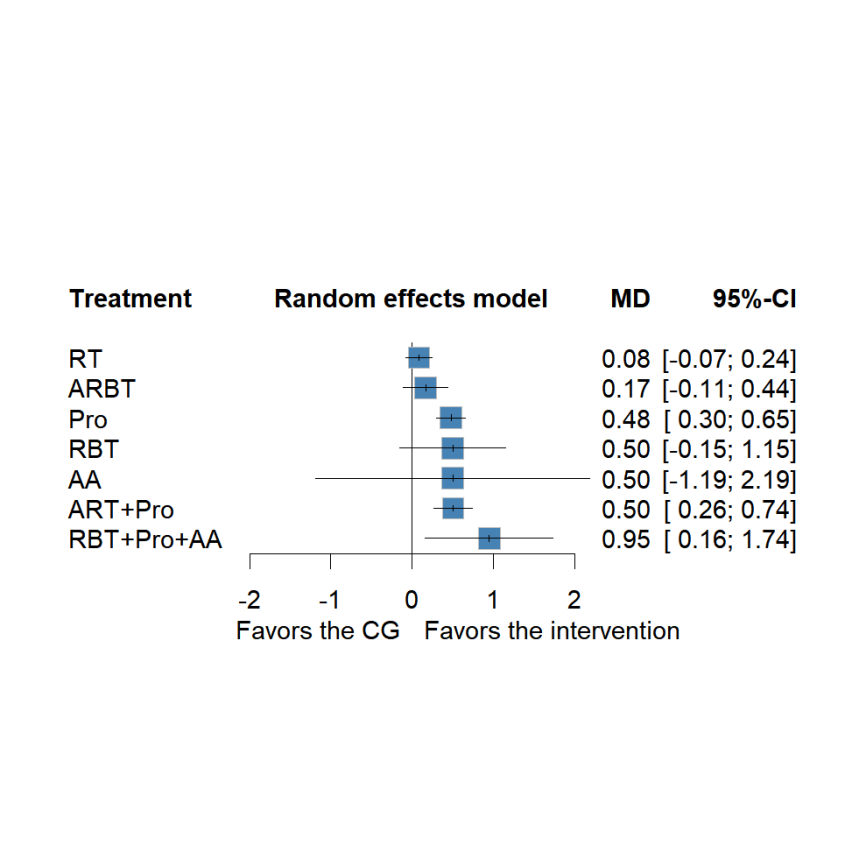


**Table S10.9** League table of SMI


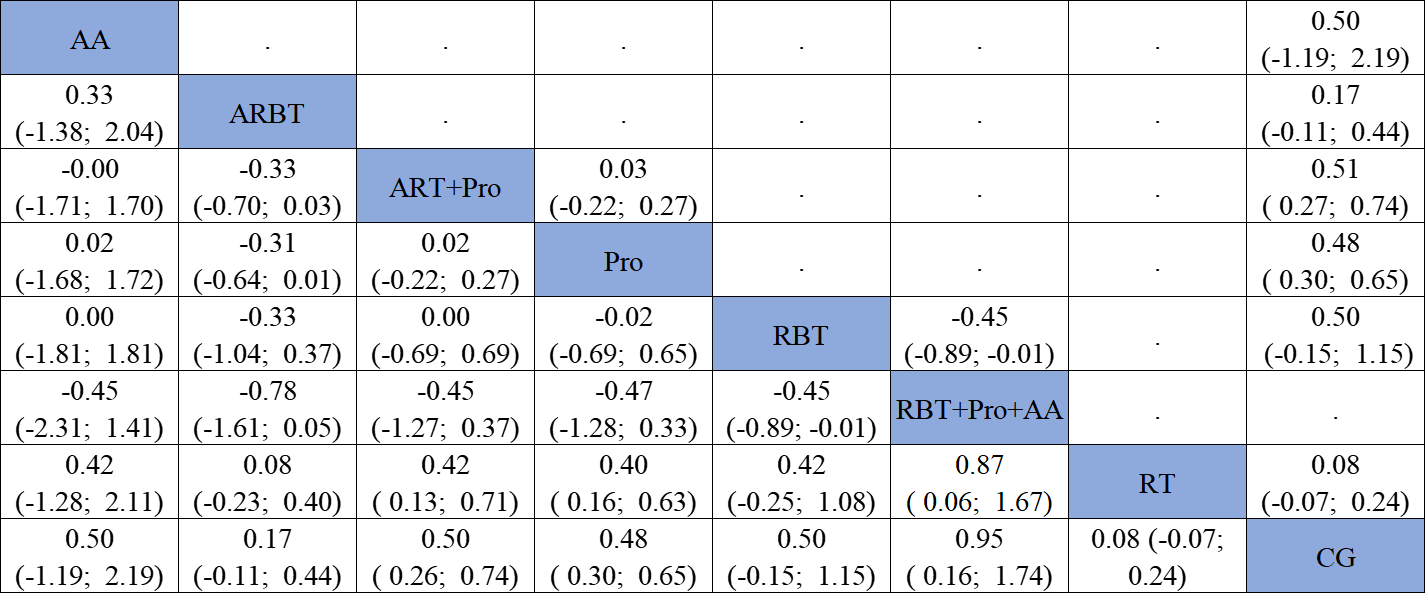


**Appendix 10.2 Secondary Subgroup Analysis**

**Table S10.1** Subgroups analysis of Muscle Strength

**Table S10.2** Subgroups analysis of Physical Function

**Table S10.3** Subgroups analysis of Muscle Mass

**Appendix11: Sensitivity analysis**

**11.1** Sensitivity Analysis Excluding Studies at High Risk of Bias

**Figure S11.1.1** Forest plot of Grip strength


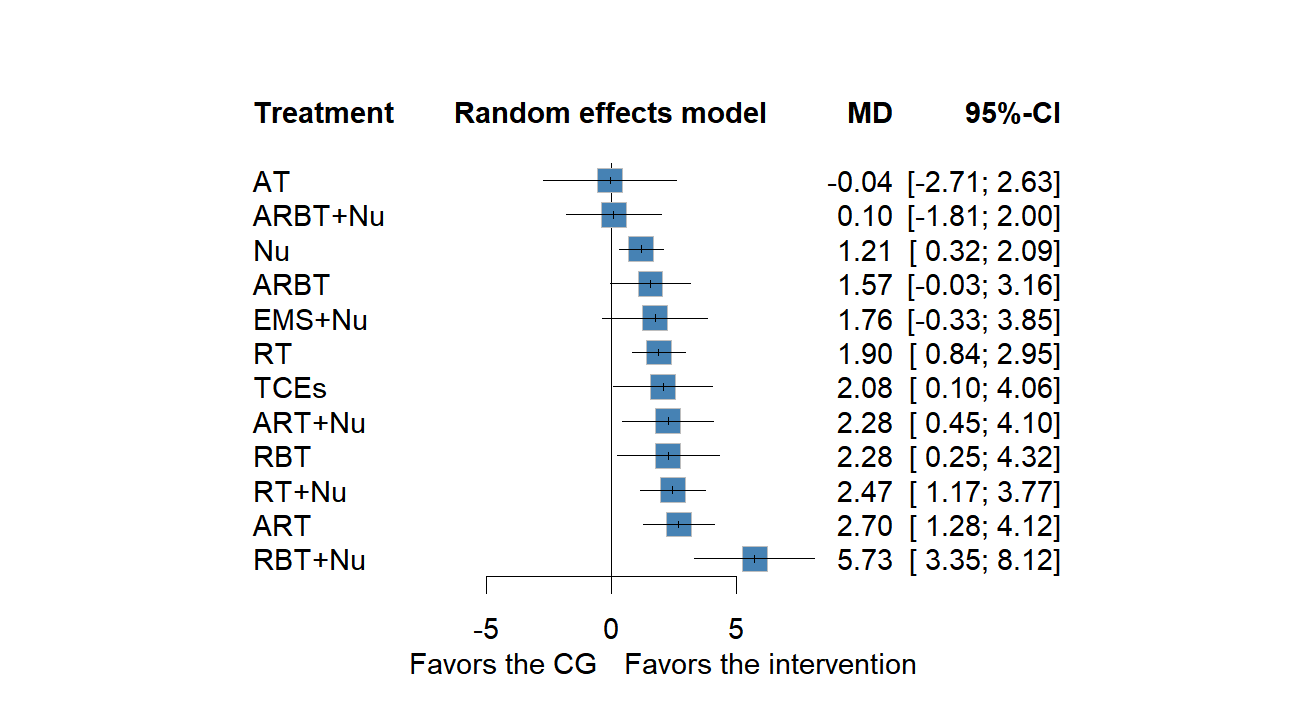


**Figure S11.1.2** Forest plot of Knee extension strength


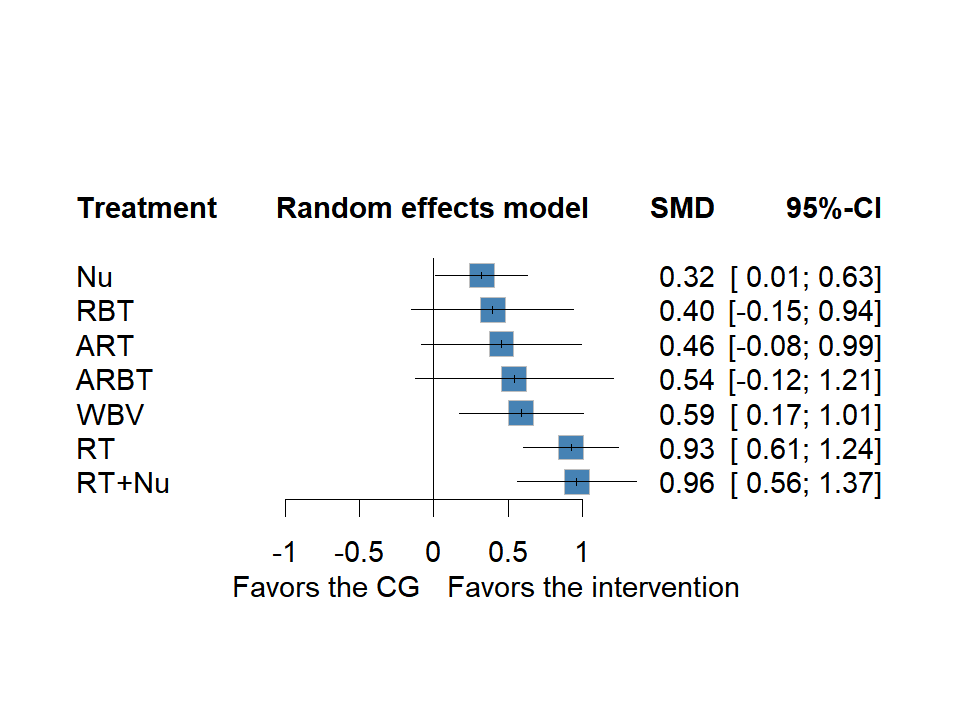


**Figure S11.1.3** Forest plot of Gait speed


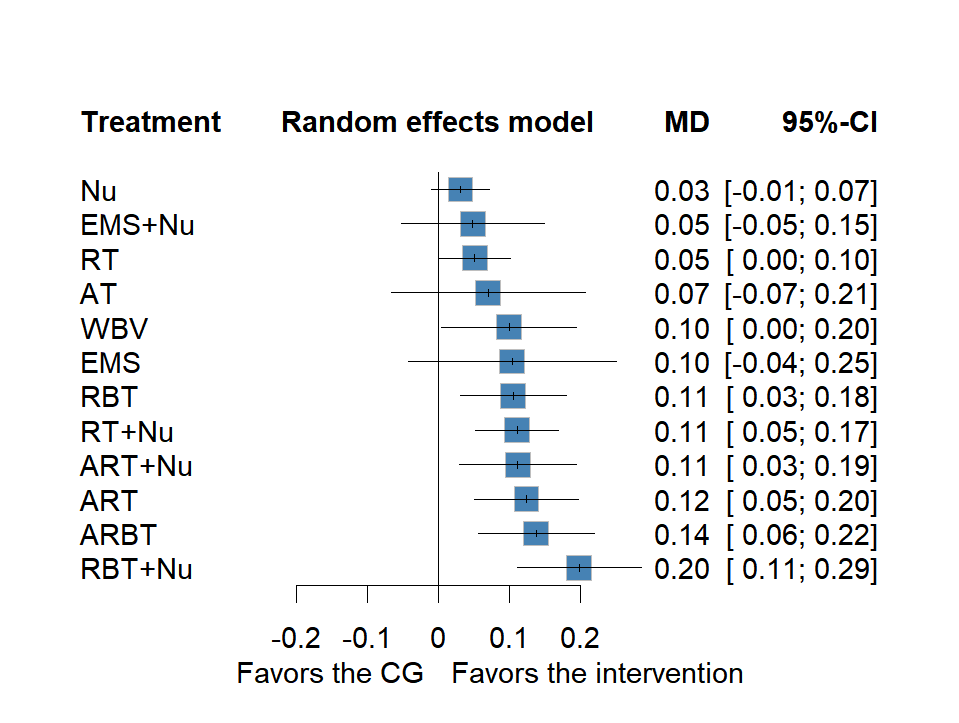


**Figure S11.1.4** Forest plot of Five-Times Sit-to-Stand test

**
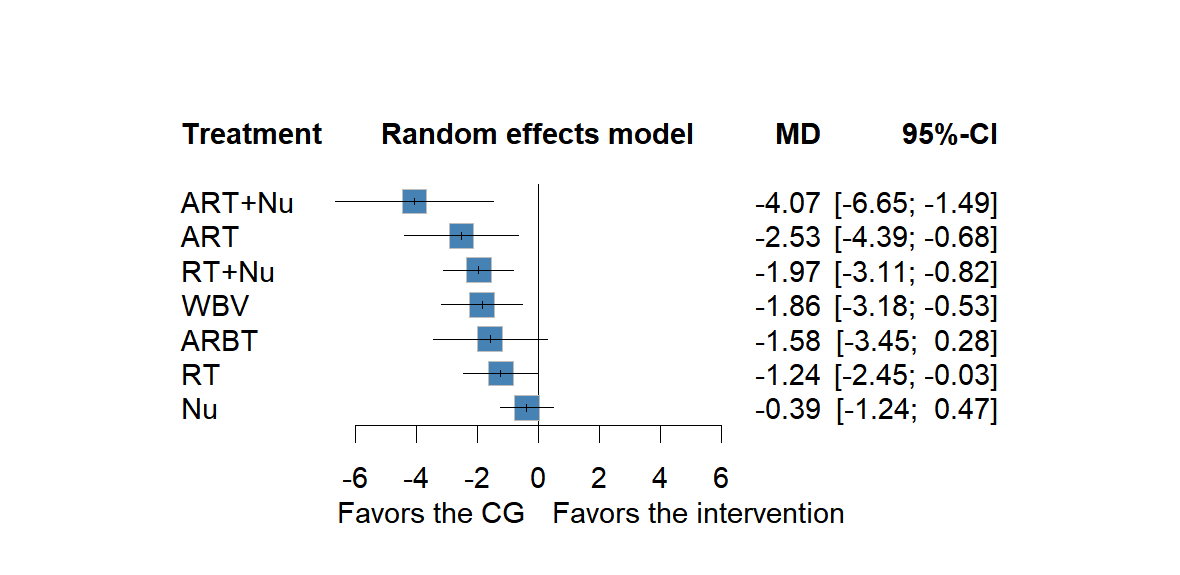
**

**Figure S11.1.5** Forest plot of Timed up and go


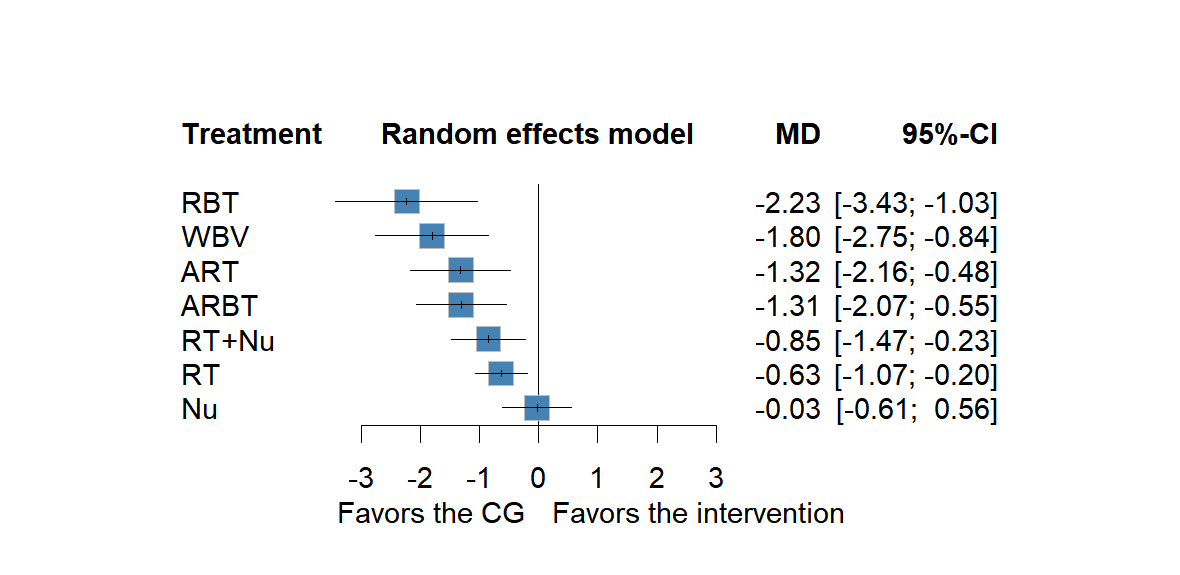


**Figure S11.1.6** Forest plot of SPPB

**
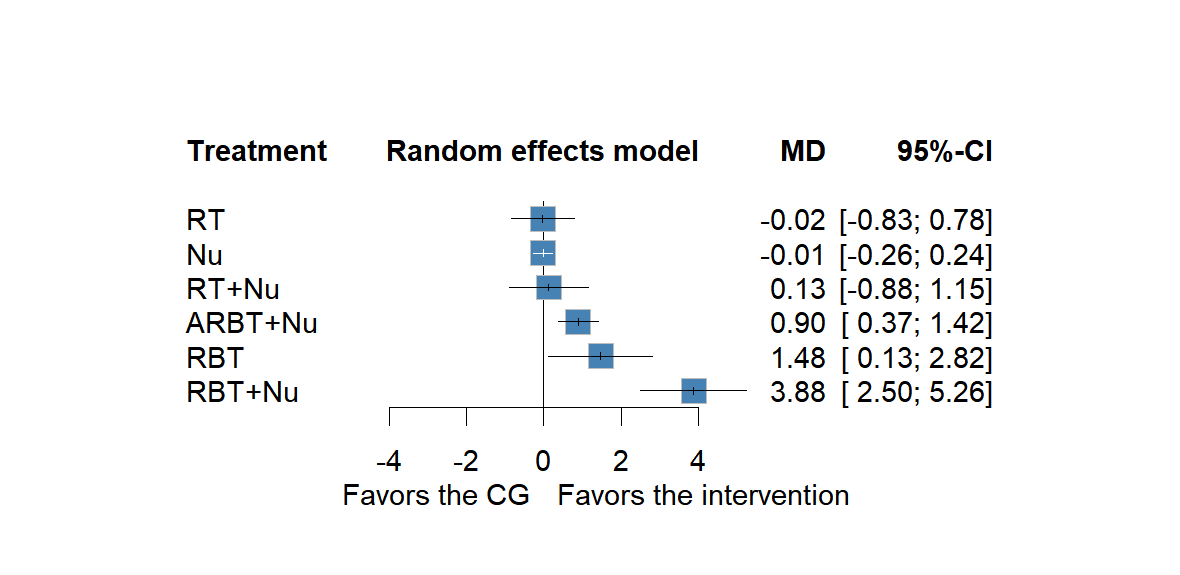
**

**Figure S11.1.7** Forest plot of Balance test


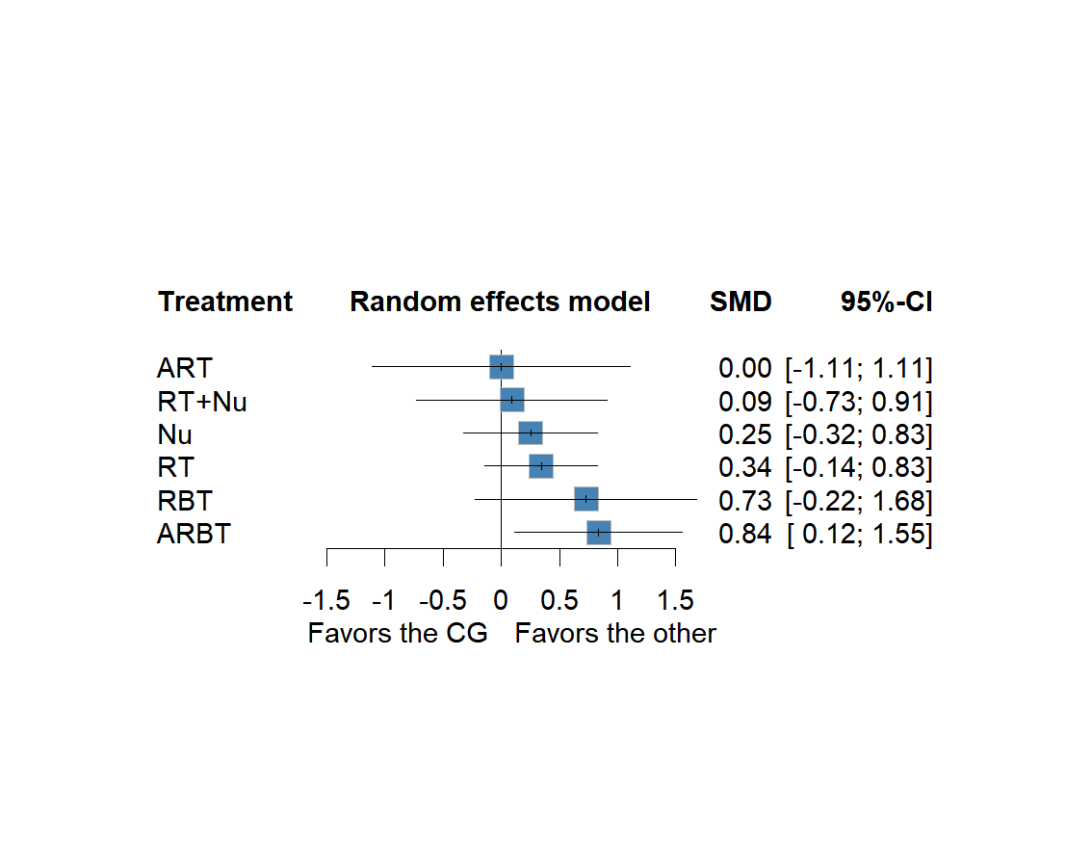


**Figure S11.1.8** Forest plot of ASMI

**
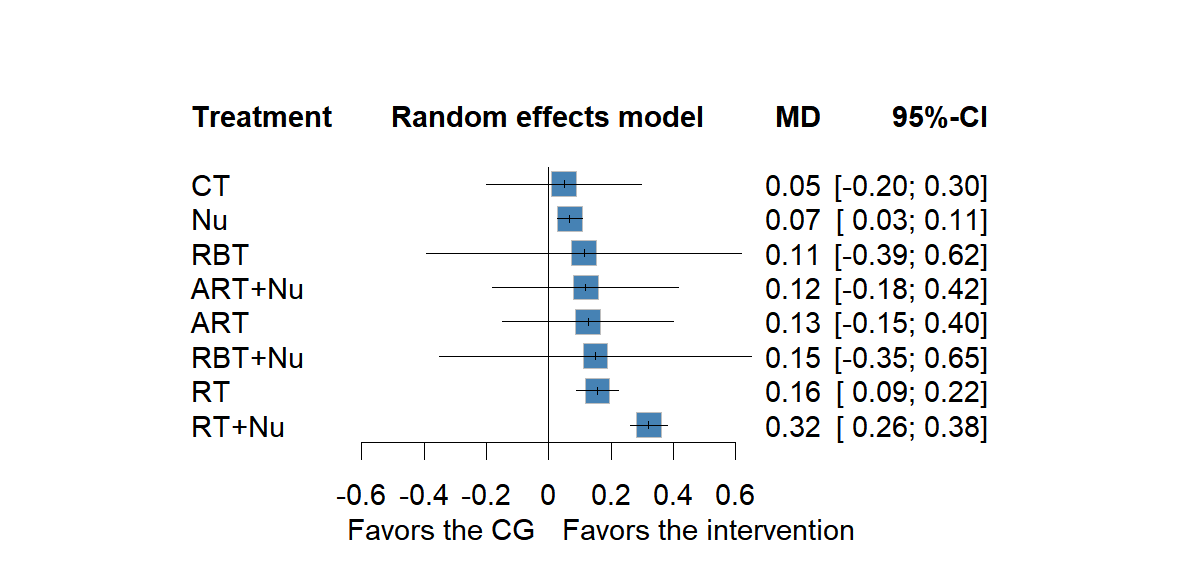
**

**Figure S11.1.9** Forest plot of SMI

**
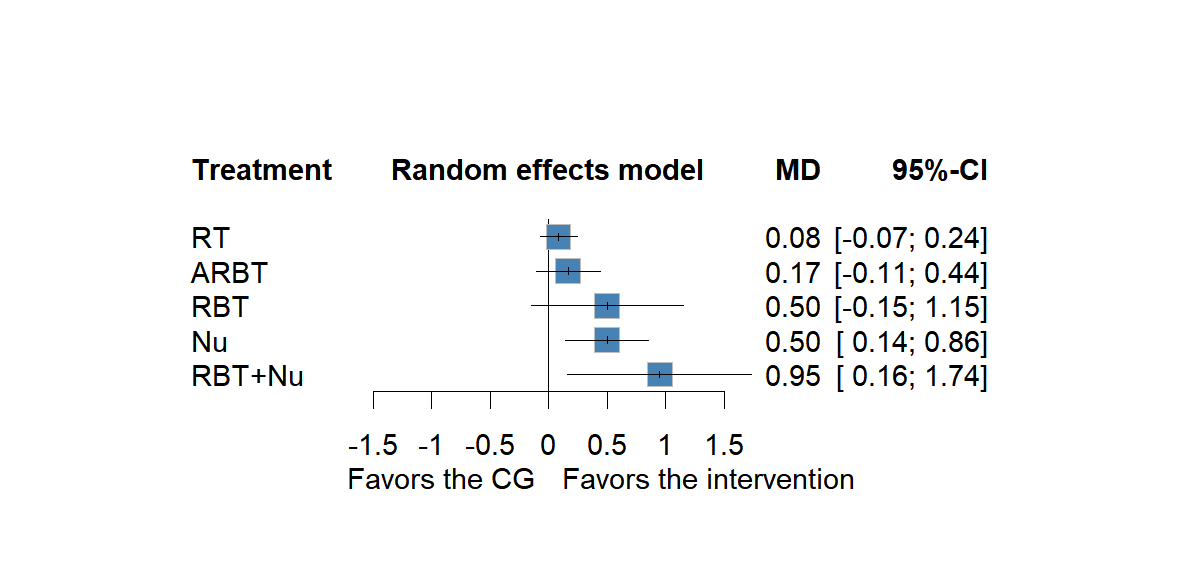
**

**11.2** Sensitivity Analysis Excluding Studies with Fewer Than 15

**Figure S11.2.1** Forest plot of Grip strength


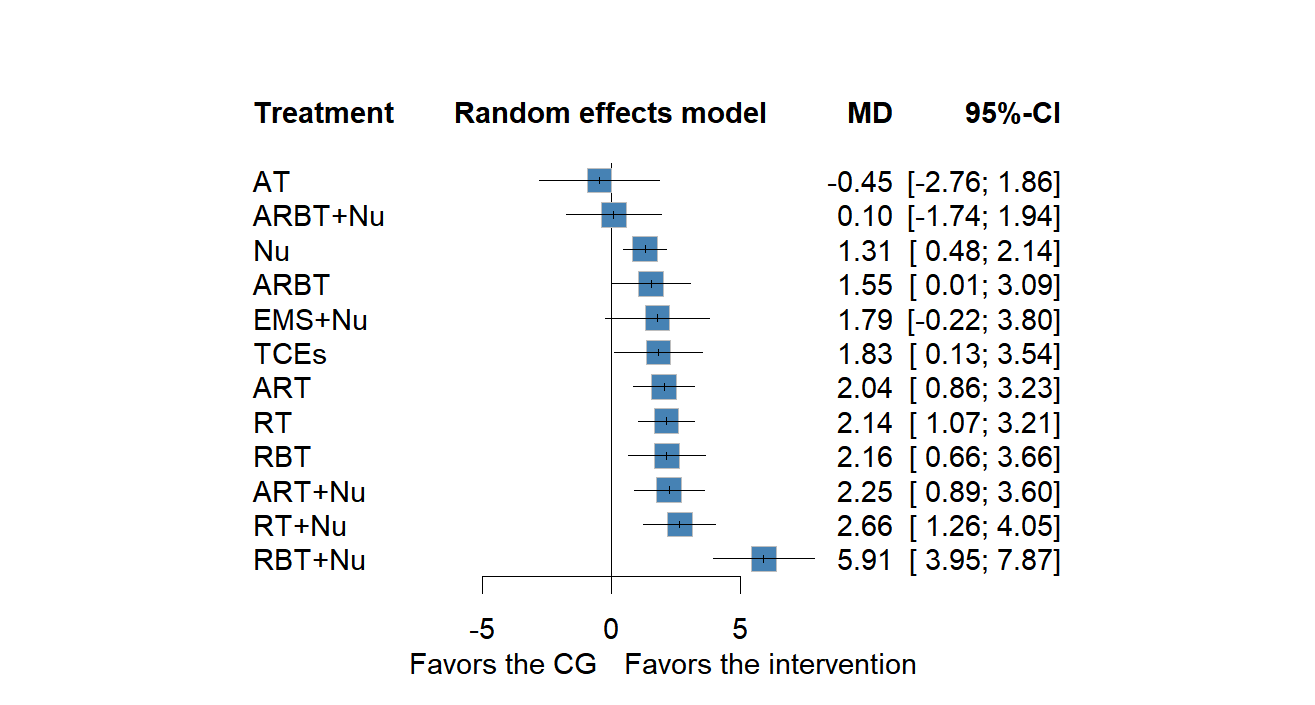


**Figure S11.2.2** Forest plot of Knee extension strength


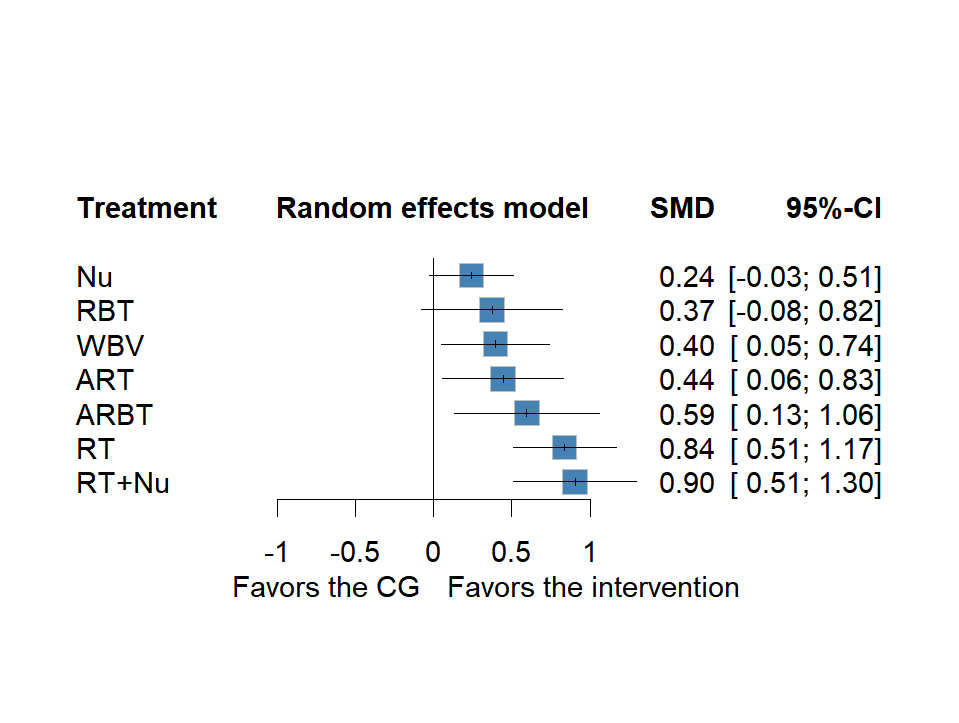


**Figure S11.2.3** Forest plot of Gait speed


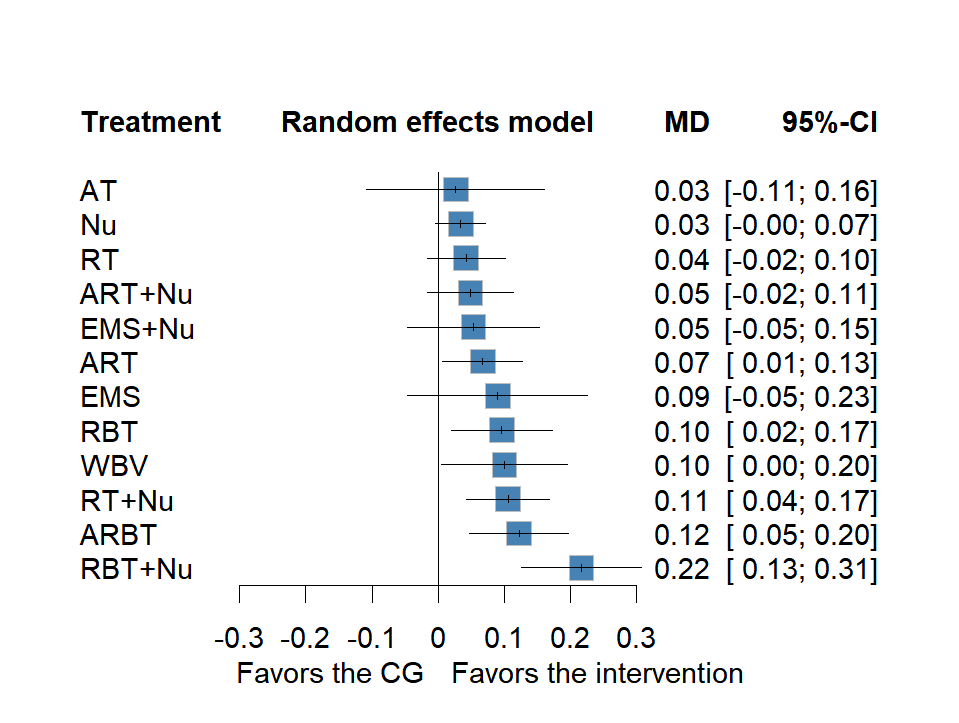


**Figure S11.2.4** Forest plot of Five-Times Sit-to-Stand test


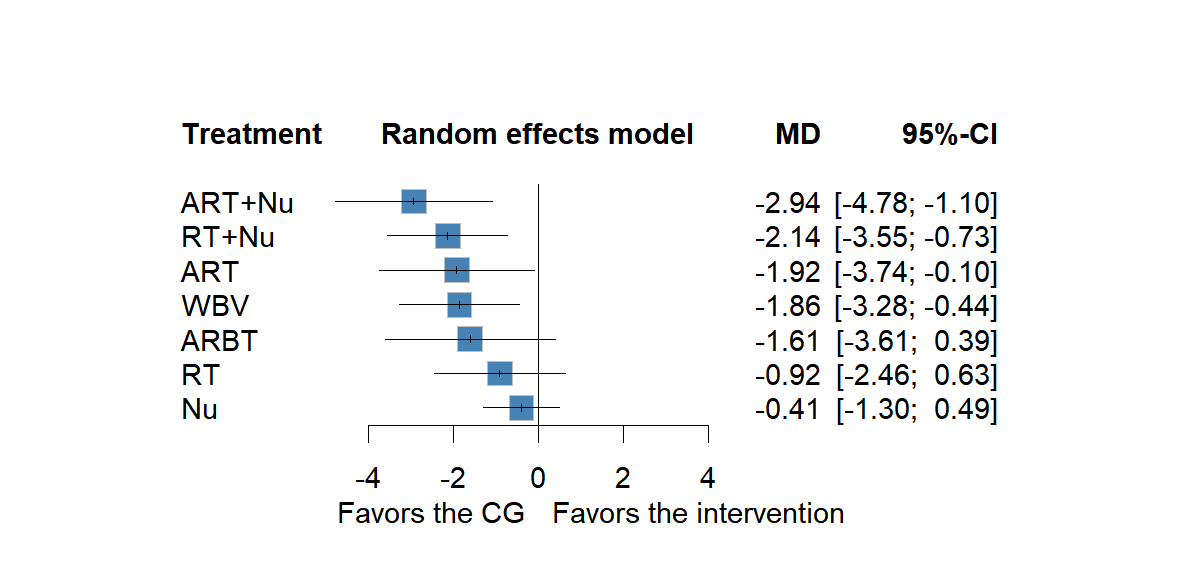


**Figure S11.2.5** Forest plot of Timed up and go


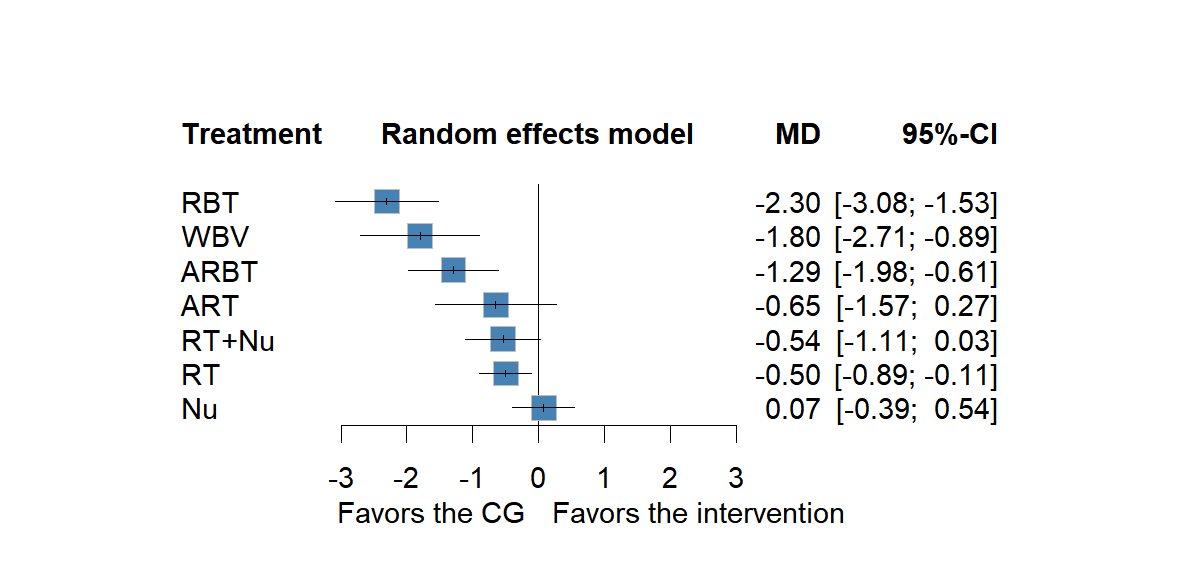


**Figure S11.2.6** Forest plot of SPPB


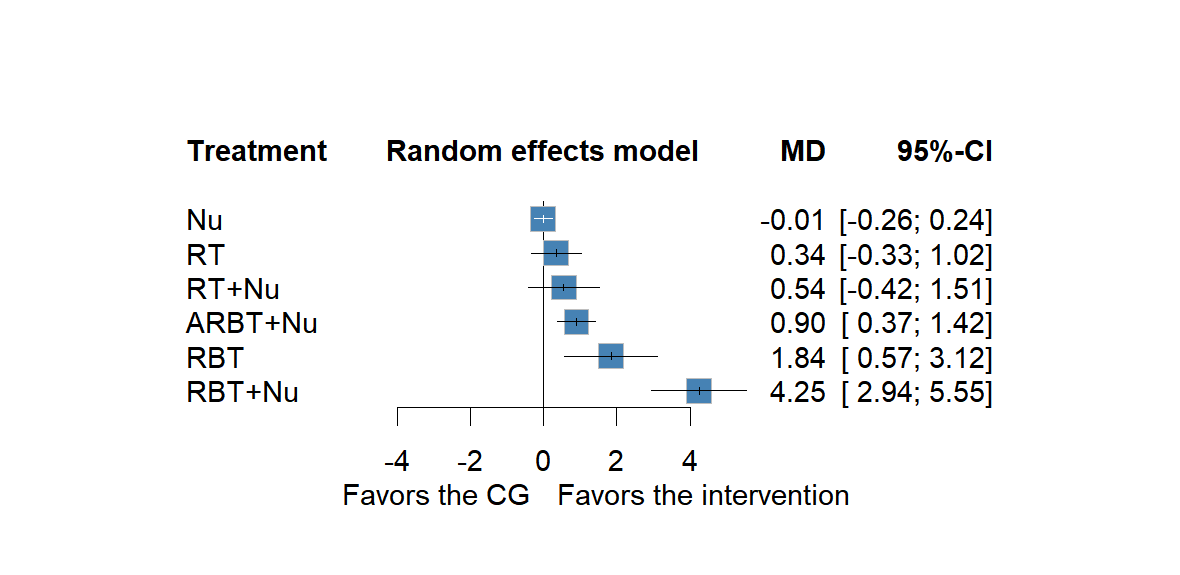


**Figure S11.2.7** Forest plot of Balance test


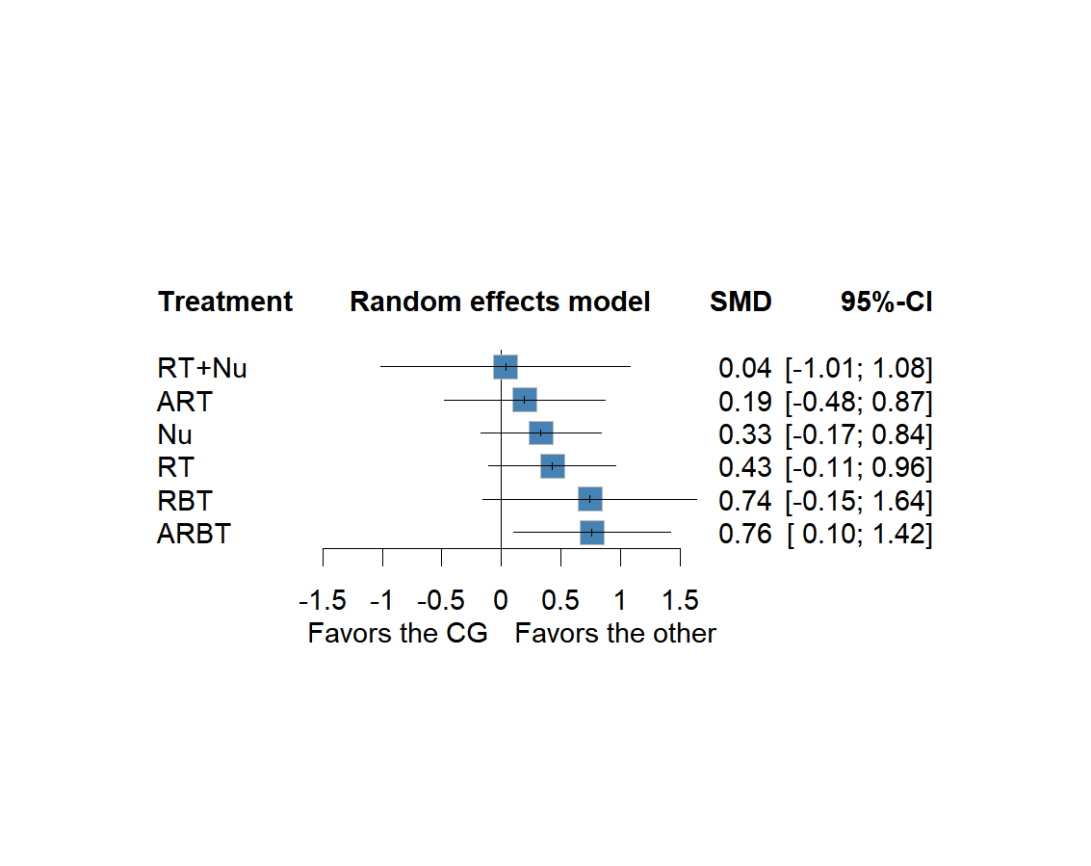


**Figure S11.2.8** Forest plot of ASMI

**
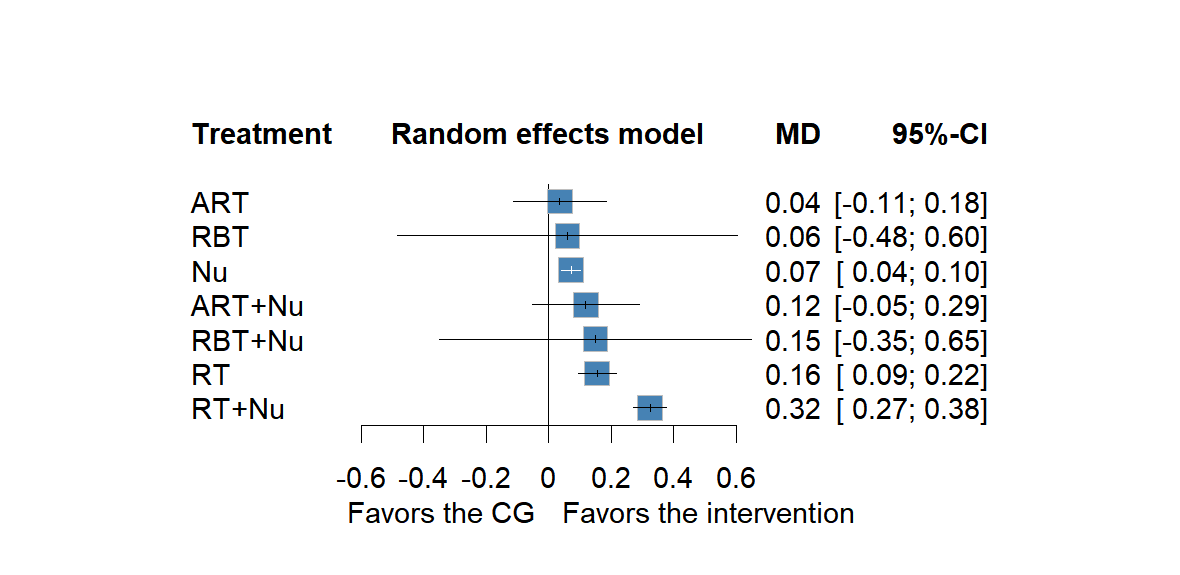
**

**Figure S11.2.9** Forest plot of SMI


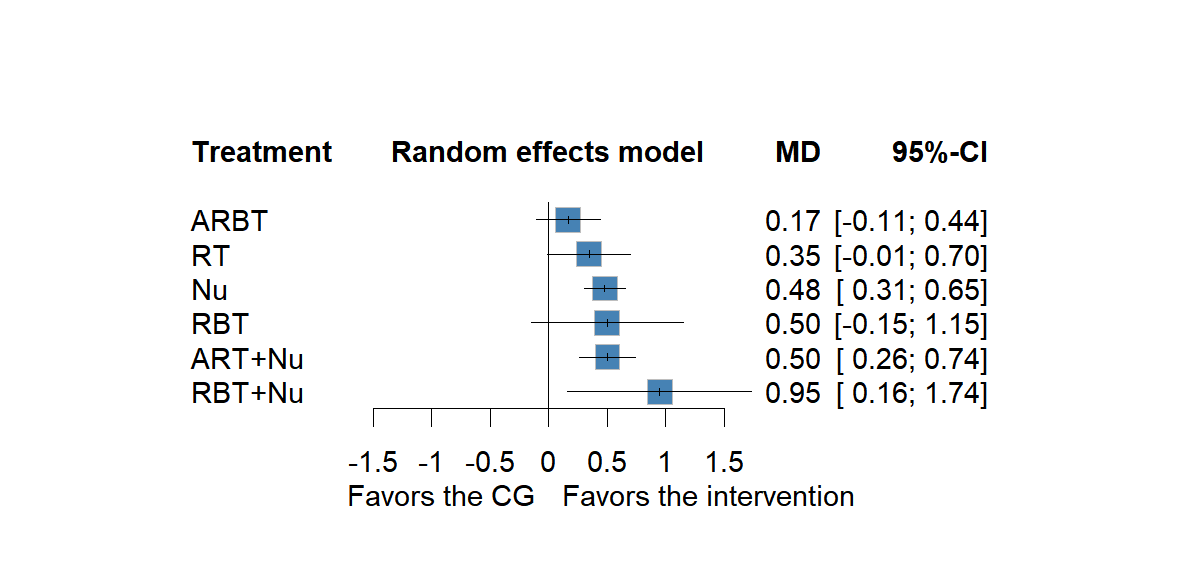


**11.3** Sensitivity Analysis Excluding Studies Including Participants with Probable Sarcopenia

**Figure S11.3.1** Forest plot of Grip strength


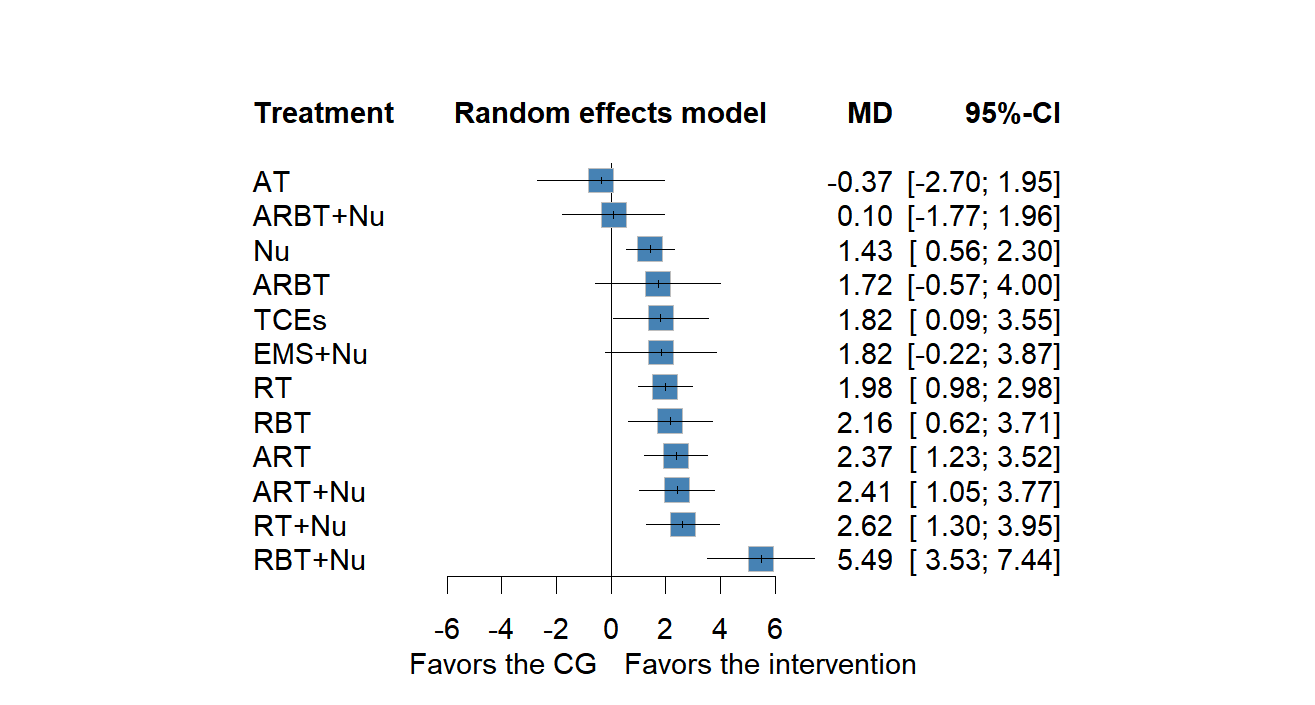


**Figure S11.3.2** Forest plot of Knee extension strength

**
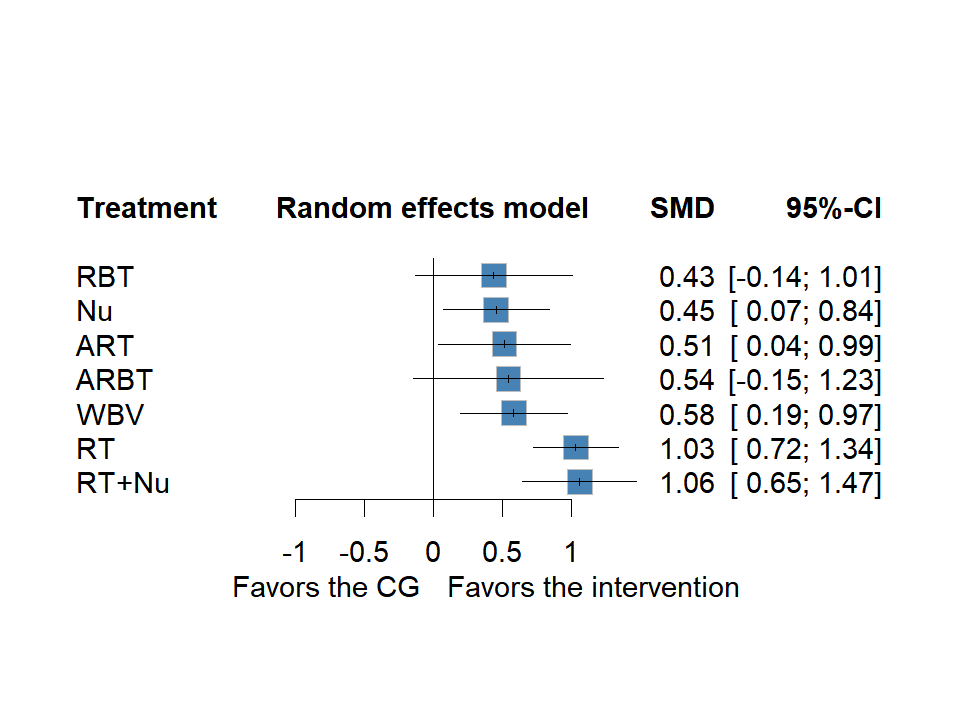
**

**Figure S11.3.3** Forest plot of Gait speed


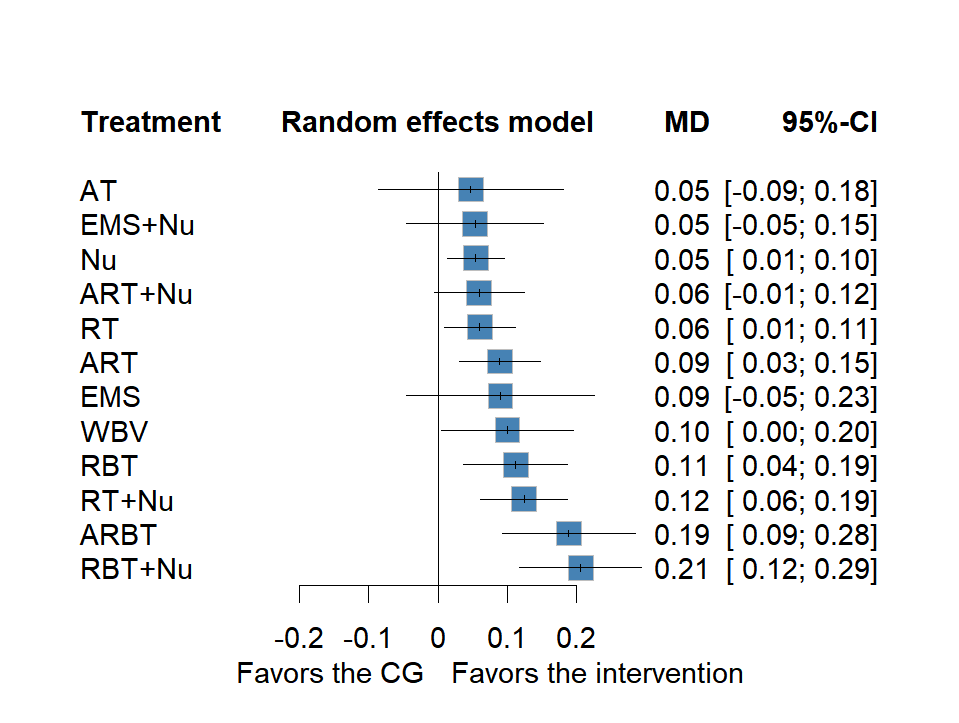


**Figure S11.3.4** Forest plot of Five-Times Sit-to-Stand test


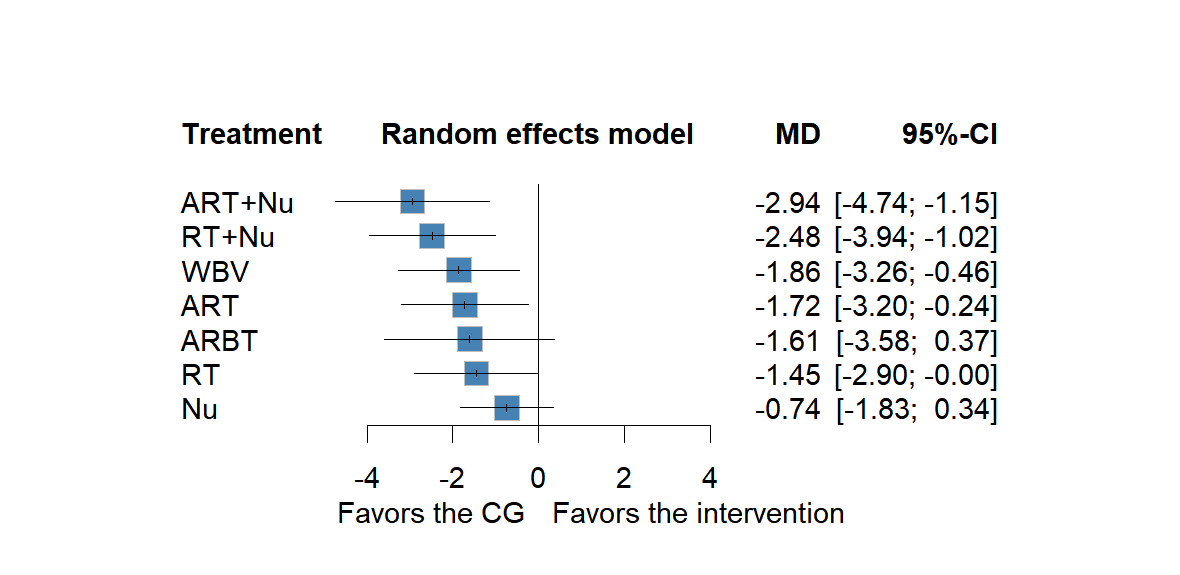


**Figure S11.3.5** Forest plot of Timed up and go


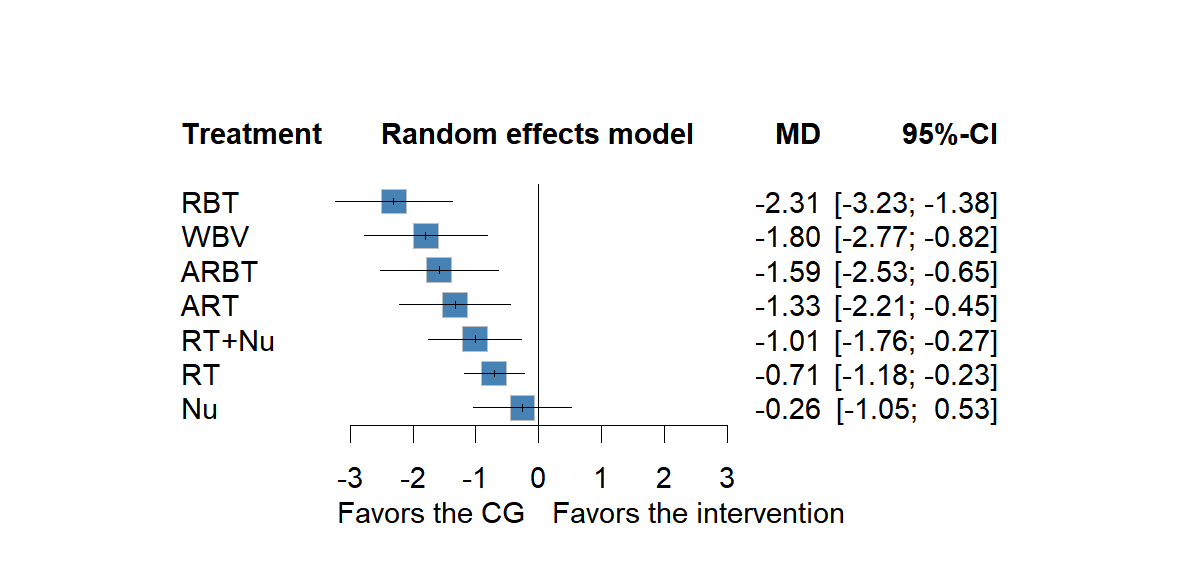


**Figure S11.3.6** Forest plot of SPPB


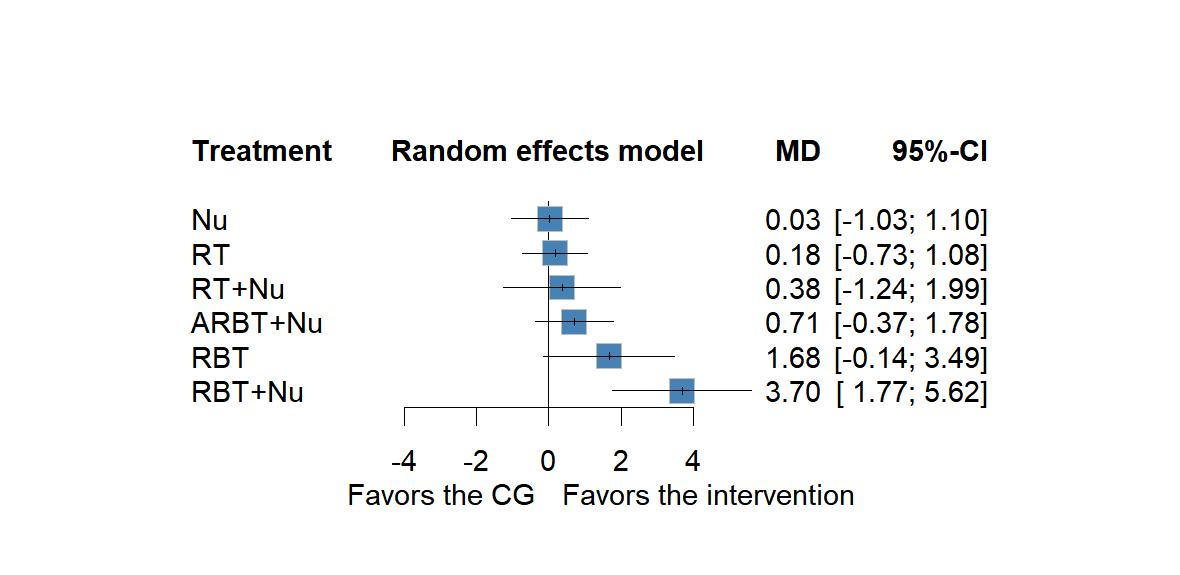


**Figure S11.3.7** Forest plot of Balance test


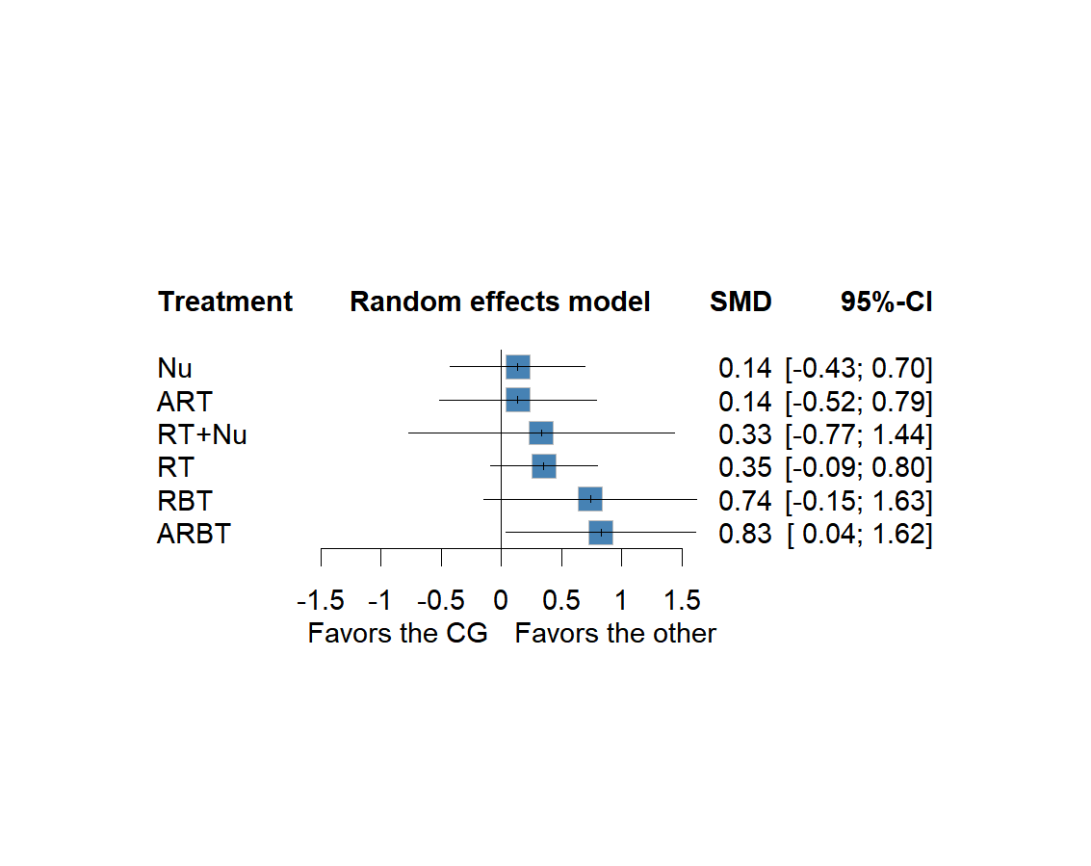


**Figure S11.3.8** Forest plot of ASMI


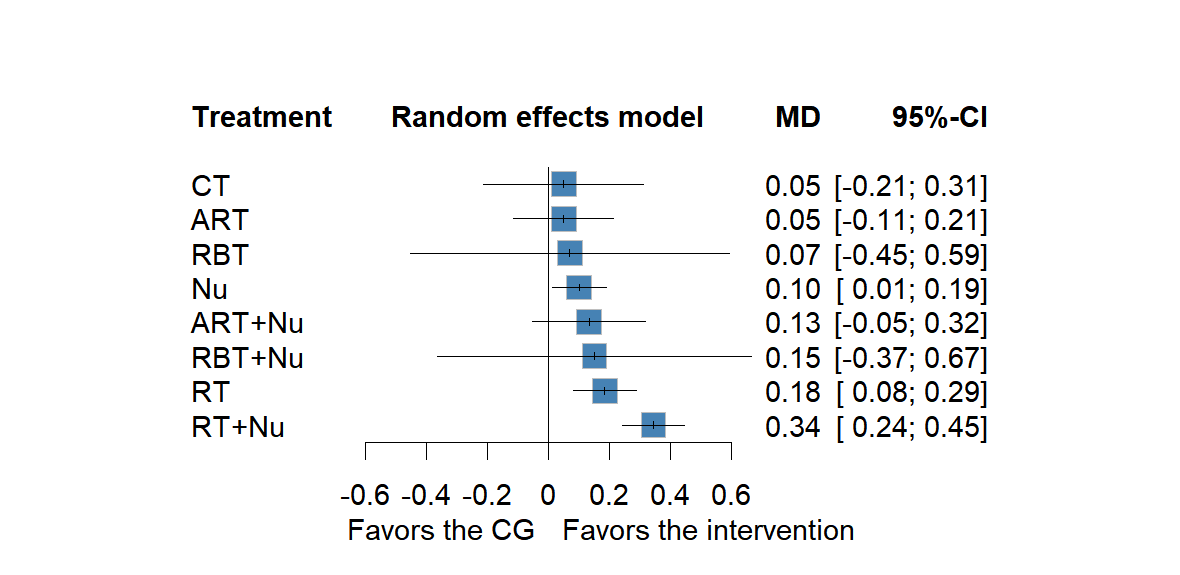


**11.4** Sensitivity Analysis Excluding Studies Not Using Internationally Recognized Consensus Criteria for Sarcopenia Diagnosis

**Figure S11.4.1** Forest plot of Grip strength


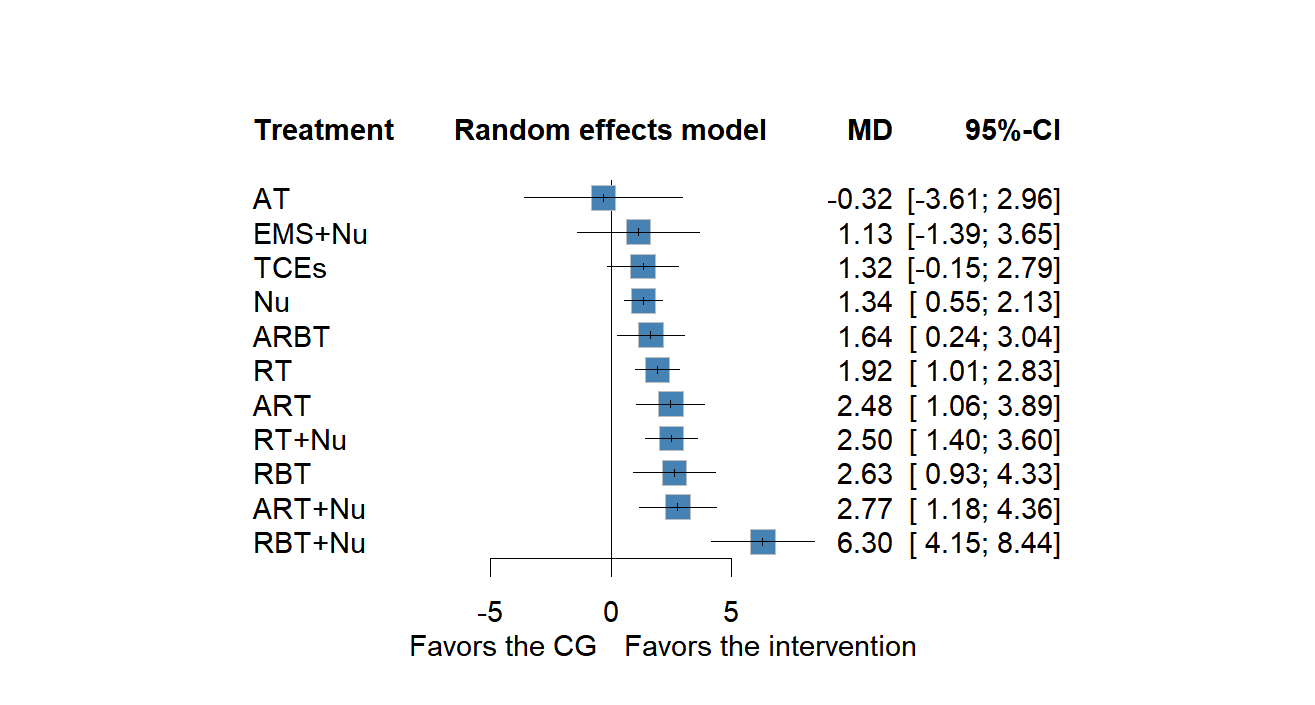


**Figure S11.4.2** Forest plot of Knee extension strength

**
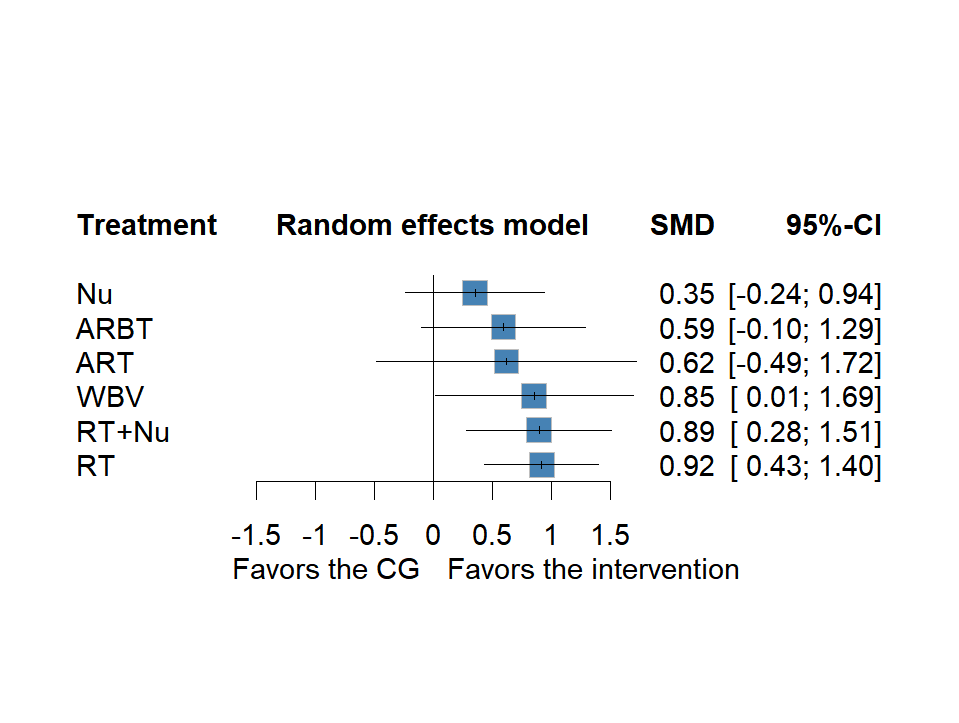
**

**Figure S11.4.3** Forest plot of Gait speed


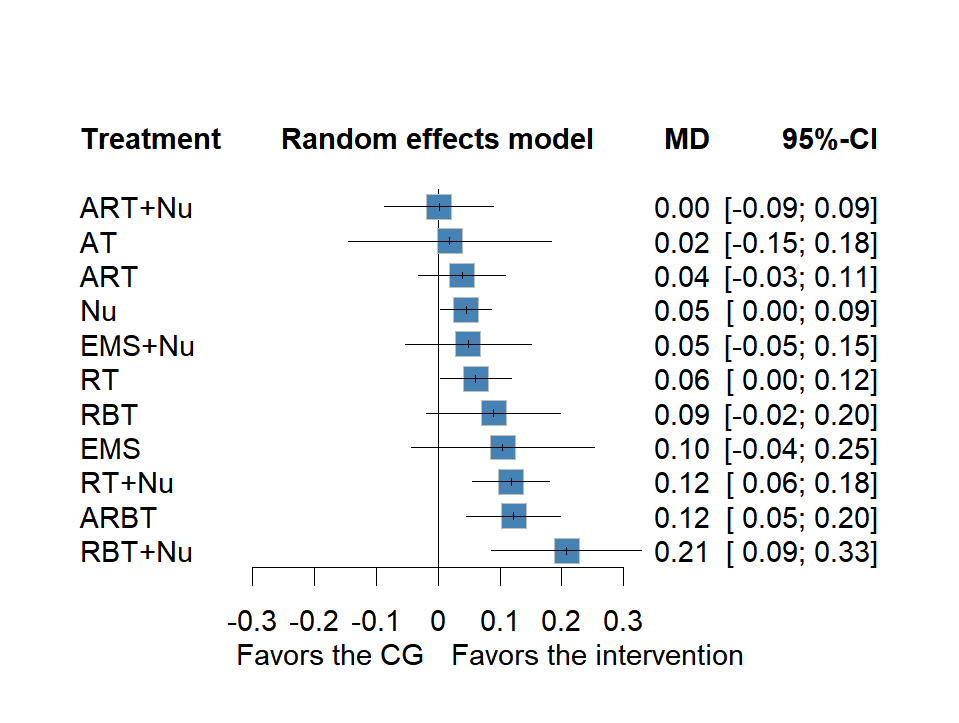


**Figure S11.4.4** Forest plot of Five-Times Sit-to-Stand test


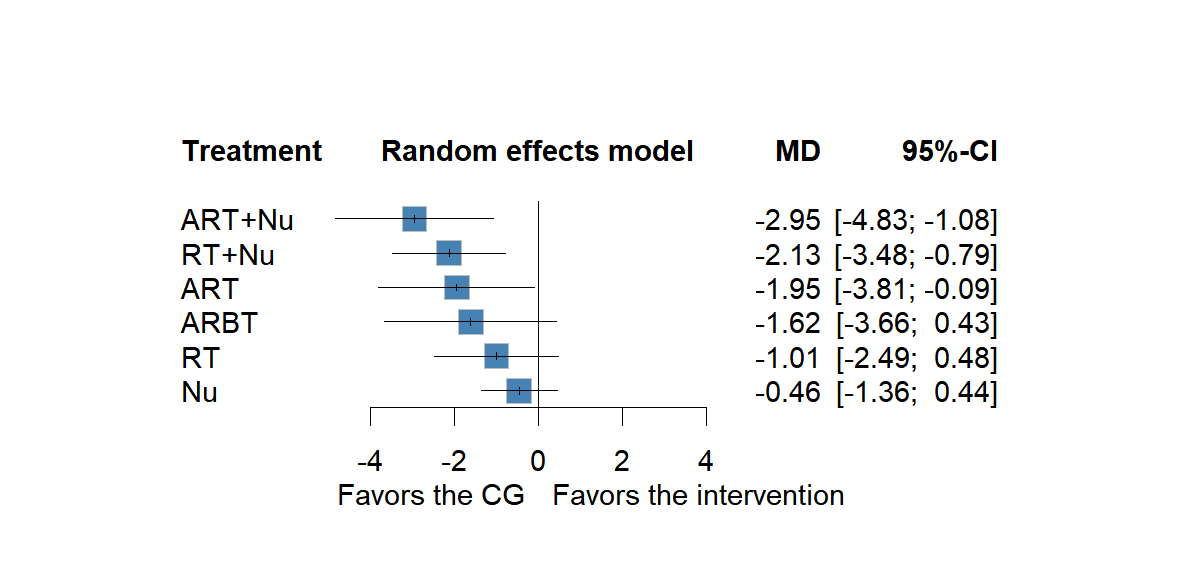


**Figure S11.4.5** Forest plot of Timed up and go


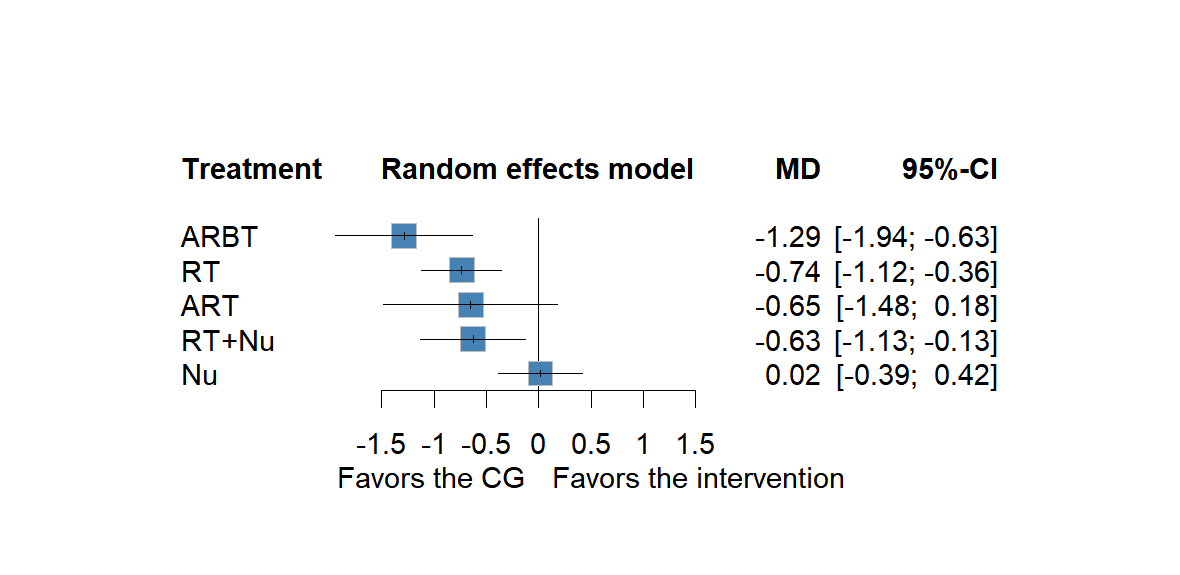


**Figure S11.4.6** Forest plot of SPPB


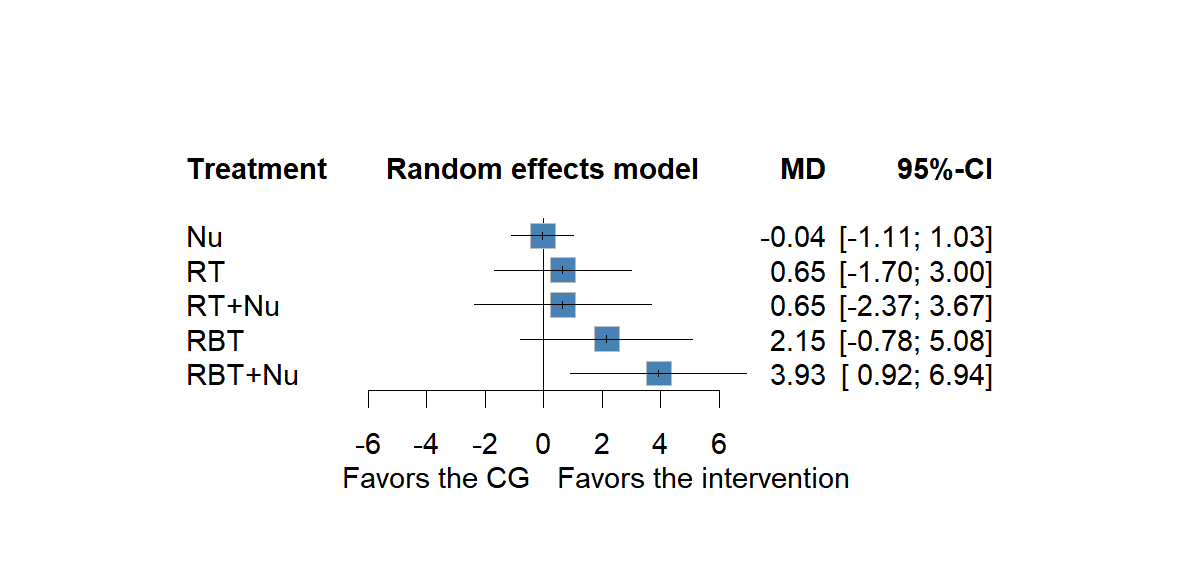


**Figure S11.4.7** Forest plot of Balance test


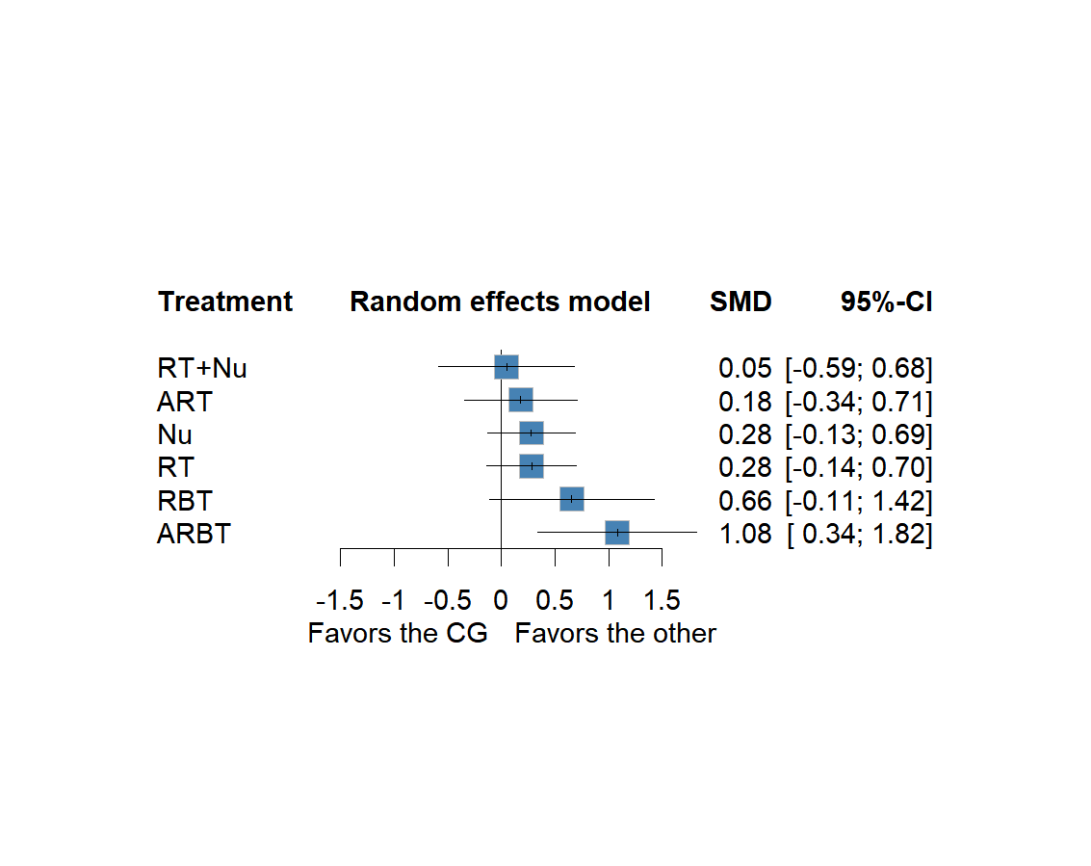


**Figure S11.4.8** Forest plot of ASMI


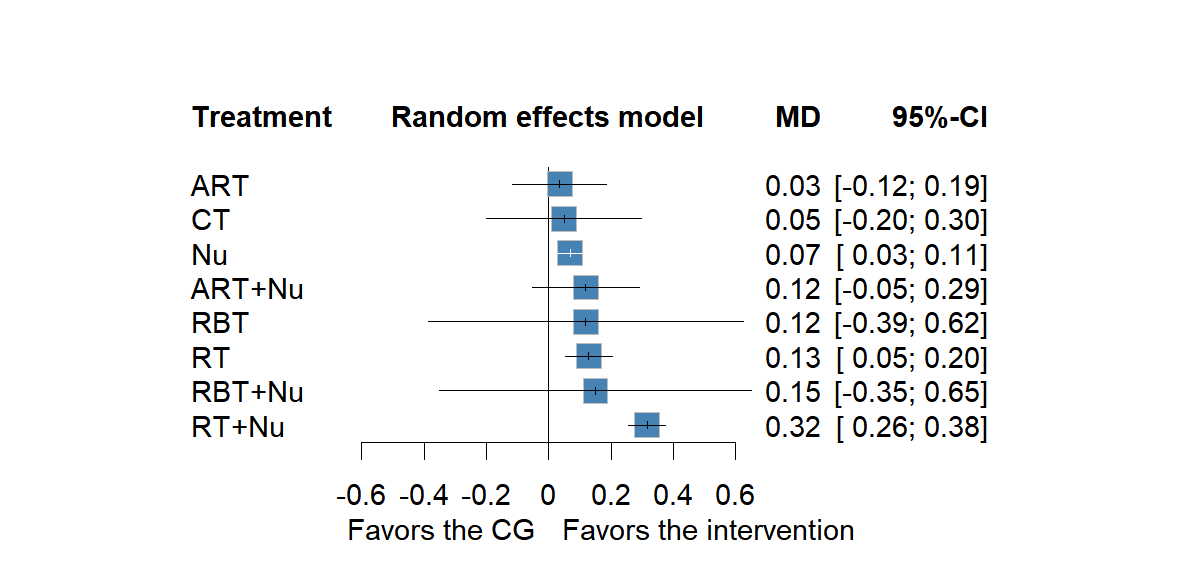


**Figure S11.4.9** Forest plot of SMI


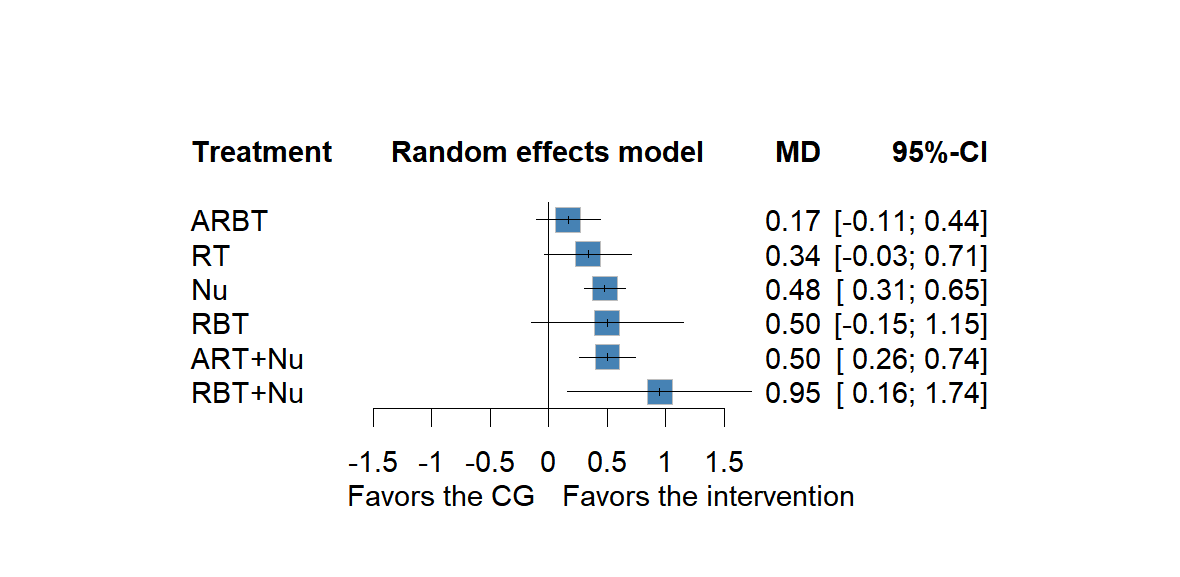

Supplement: Supplementary file 1 [file mmc1.zip › mmc1.docm]
